# The causal relationships between body composition and heart failure: A two-sample mendelian randomization study

**DOI:** 10.1038/s41598-025-00406-7

**Published:** 2025-05-02

**Authors:** Chenxi Lu, Zhuang Guo, Zhuoran Wang, Ke Xu, Guiyuan Han, Ke Peng, Xiaoying Liu, Yichong Li, Yu Shi

**Affiliations:** 1 School of Public Health, Shenzhen University, No. 12 Langshan Road, Nanshan District, Shenzhen, 518057 Guangdong People’s Republic of China; 2 National Clinical Research Center for Cardiovascular Diseases, Heart Failure Ward, Fuwai Hospital Chinese Academy of Medical Sciences, Shenzhen, Guangdong People’s Republic of China; 3 Department of Mathematics, School of Arts and Sciences, Washington University in St. Louis, St. Louis, MO USA

**Keywords:** Heart failure, Body mass index, Fat mass, Fat-free mass, Mendelian randomization, Heart failure, Epidemiology

## Abstract

The objective of this study was to assess the causal relationships between various body composition indicators, including body mass index (BMI), waist circumference (WC), waist-to-hip ratio (WHR), and specific measures of fat mass (right arm, right leg, trunk, and whole-body fat mass) and fat-free mass, and the risk of heart failure (HF) using a two-sample Mendelian randomization (MR) approach. We used genome-wide significant single nucleotide polymorphisms (SNPs) related to body composition from the UK Biobank, GIANT, and FinnGen as instrumental variables. To estimate causal associations, we applied multiple methods, including inverse variance weighted (IVW), IVW with multiplicative random effects (IVW_mre), MR-PRESSO, and maximum likelihood. The results demonstrated that each standard deviation (SD) increase in BMI (OR = 1.48; 95% CI: 1.37–1.60; *P* = 1.24E-23), WC (OR = 1.60; 95% CI: 1.45–1.77; *P* = 1.72E-20), and WHR (OR = 1.25; 95% CI: 1.01–1.54; *P* = 3.70E-02) was significantly associated with increased HF risk. Comparable associations were observed for fat mass in the right arm (OR = 1.42; *P* = 6.60E-17), right leg (OR = 1.57; *P* = 5.80E-18), trunk (OR = 1.31; *P* = 3.02E-11), and the whole body (OR = 1.34; *P* = 2.24E-12). Fat-free mass—both whole-body (OR = 1.34; *P* = 4.77E-10) and regional measurements (right arm, right leg, trunk)—also exhibited positive associations with HF risk. Leave-one-out analyses confirmed the stability of these findings and underscored the significance of multiple body composition indicators in HF risk assessment and prevention.

## Introduction

Heart failure (HF), also referred to as congestive heart failure (CHF) or simple cardiac failure, represents a severe and terminal stage of various cardiovascular diseases. It has been characterized as a complex clinical syndrome characterized by abnormalities in cardiac structure or function, leading to impaired ventricular filling or ejection capacity. Patients with HF had poor quality of life, high rates of hospital readmission, and elevated mortality risks^1,2^. Previous studies indicated that there have been approximately 64 million individuals with HF worldwide, and increasing incidence has been observed in many middle-income and low-income countries^3^. Therefore, identifying the causal risk factors is highly important for preventing HF^4^. Existing knowledge has suggested that obesity is one of the risk factors for the development of HF^5,6^. Traditional epidemiological studies have shown that excessive increases in fat mass are associated with increased incidence of heart failure^7^. It has been reported that each increase of 100 cm3 in visceral fat mass was associated with a 7% increased hazard for HF events^8^. However, given the methodological limitations of conventional observational research, it is difficult to identify the actual causality between body composition and HF^9^. Concerning the indicator of whole-body fat-free mass, the results of the association with the occurrence of heart failure were still inconsistent. Additionally, the relationship between location-specific fat-free mass and heart failure remains uncertain.

However, there is still considerable controversy over accurate anthropometric measurements of obesity. The common anthropometric measurements include body mass index (BMI), waist circumference (WC), and waist-to-hip ratio (WHR). Although BMI, WC, and WHR are widely used for obesity assessment due to their simplicity and noninvasiveness in measurement, these three indicators could not be applied to differentiate between fat content (composed of subcutaneous and visceral fat tissue) and fat-free mass (including muscle, bones, internal organs, ligaments, and tendons)^10,11^.

Mendelian randomization (MR) utilizes genetic variation as instrumental variables (IVs) to examine the causal relationships between exposures and outcomes. MR analyses can help reduce confounding bias inherent in conventional observational studies. However, while MR is less susceptible to confounding, potential selection bias in the included genome-wide association studies (GWAS) must be considered, as participation in biobank-based studies may be associated with both genetic predispositions and health-related behaviors. In this study, we conducted a series of two-sample MR analyses to explore the causal relationships between several body composition indicators, including BMI, WC, WHR, body fat mass (right arm fat mass, right leg fat mass, trunk fat mass, whole body fat mass), body fat-free mass (right arm fat-free mass, right leg fat-free mass, trunk fat-free fat mass, whole-body fat-free mass) and the risk of HF development.

## Methods

### Study overview

Based on the three key assumptions of MR studies, we used genetic variants as instrumental variables (IVs) to investigate the causal relationship between several body composition indicators, including BMI, WC, WHR, body fat mass (right arm fat mass, right leg fat mass, trunk fat mass, whole body fat mass), body fat-free mass (right arm fat-free mass, right leg fat-free mass, trunk fat-free fat mass, whole-body fat-free mass) and the development of HF (Supplementary study design).

### Genetic instrument of body composition indicators

Except WHR, the body composition datasets utilized in this study were obtained from the UK Biobank. In the UK Biobank study, researchers have employed bioelectrical impedance analysis to assess both fat mass and fat-free mass. We included 8 of these measurements, such as right leg fat mass (GWAS ID: ukb-b-18096), right arm fat mass (GWAS ID: ukb-b-6704), right leg fat-free mass (GWAS ID: ukb-b-12828), right arm fat-free mass (GWAS ID: ukb-b-19520), trunk fat mass (GWAS ID: ukb-b-20044), trunk fat-free mass (GWAS ID: ukb-b-17409), whole-body fat mass (GWAS ID: ukb-b-19393) and whole-body fat-free mass (GWAS ID: ukb-b-13354). We also included two measures of body size, BMI (GWAS ID: ukb-b-19953) and WC (GWAS ID: ukb-b-9405). The GWAS for WHR was derived from the Genetic Investigation of Anthropometric Traits (GIANT) (GWAS ID: ieu-a-73), which aims to identify genetic loci influencing individual characteristics and traits in humans. All the abovementioned GWASs were available in the IEU GWAS database (https://gwas.mrcieu.ac.uk/).

### Genetic instrument for heart failure

The GWAS data for HF (phenotype “All-cause Heart Failure”) were obtained from the fifth edition of the FinnGen biological database released in 2021 (https://r5.risteys.finngen.fi/phenocode/I9_HEARTFAIL_ALLCAUSE). In total, 23,397 patients with HF and 194,811 controls were included, with 16,380,447 SNPs. The patients included in these data included males and females. In this study, the phenotype of “All-cause Heart Failure” was defined according to the Tenth Edition of the International Classification of Diseases (ICD10), including congestive heart failure (I50), hypertensive heart disease with (congestive) heart failure (I11.0), hypertensive heart and renal disease with (congestive) heart failure (I13.0), and hypertensive heart and renal disease with both (congestive) heart failure and renal failure (I13.2). The FinnGen research project was launched in 2017 and combines genomic information with digital healthcare data from Finnish participants. In this study, to ensure similar genetic characteristics between the exposure and outcome samples, the European population database was uniformly selected for the study. No further informed consent was needed for this study because data was already available to the public. All studies were previously approved by the appropriate institutional review boards.

### Genetic instrument selection

Valid IVs should fulfill three assumptions^12^: the IVs are strongly correlated with exposure; the IVs are not interfered with by any confounding factors; and the IVs affect the outcome only through exposure. The criteria for selecting instrumental variables were as follows: (1) SNPs met the genome-wide significance criterion (*P* < 5 × 10^–8^), the parameter *R*^2^threshold was set to 0.001, and the kilobase pair (kb) was set to 10,000 to minimize interference from linkage disequilibrium (LD)^13^; (2) The strength of the genetic instruments was assessed by calculating the F statistic via the formula $F = {\kern 1pt} {\kern 1pt} \left( {\frac{{N{\kern 1pt} - {\kern 1pt} K{\kern 1pt} - 1}}{K}} \right)\,\left( {\frac{{R^{2} }}{{1{\kern 1pt} - R^{2} }}} \right)$, where *R*^2^denotes the proportion of exposure variation explained by SNPs, N denotes the sample size, and K indicates the number of SNPs, with all SNPs demonstrating F-statistics > 10 to avoid weak tool bias^14^. Ultimately, in this study, 368 BMI SNPs, 286 WC SNPs, 23 WHR SNPs, 338 right arm fat mass SNPs, 324 right leg fat mass SNPs, 326 trunk fat mass SNPs, 340 whole-body fat mass SNPs, 424 right arm fat-free mass SNPs, 419 right leg fat-free mass SNPs, 468 trunk fat-free mass SNPs and 463 whole-body fat-free mass SNPs, were used as genetic instruments. Table 1 provides a comprehensive summary of the instrument variants utilized in this MR study, and additional details are shown in Supplementary Tables S1-S11.

**Table 1:** Characteristics of the genetic variants used in the present MR study.

Variables	Sample size	SNPs (n)	Participate	Consortium	Cochran’s Q *P*	Source
BMI	461,460	9,851,867	European	Pan UKBB	0.9999848	https://pan-ukb-us-east-1.s3.amazonaws.com/sumstats_flat_files/continuous-23104-both_sexes-irnt.tsv.bgz
WC	462,166	9,851,867	European	Pan UKBB	0.9999989	https://pan-ukb-us-east-1.s3.amazonaws.com/sumstats_flat_files/continuous-48-both_sexes-irnt.tsv.bgz
WHR	212,244	2,560,782	European	GIANT	0.6866845	https://doi.org/10.1371/journal.pgen.1005378
Arm fat mass (right)	454,757	9,851,867	European	Pan UKBB	0.9999791	https://pan-ukb-us-east-1.s3.amazonaws.com/sumstats_flat_files/continuous-23120-both_sexes-irnt.tsv.bgz
Leg fat mass (right)	454,846	9,851,867	European	Pan UKBB	0.9999846	https://pan-ukb-us-east-1.s3.amazonaws.com/sumstats_flat_files/continuous-23112-both_sexes-irnt.tsv.bgz
Trunk fat mass	454,588	9,851,867	European	Pan UKBB	0.9987673	https://pan-ukb-us-east-1.s3.amazonaws.com/sumstats_flat_files/continuous-23128-both_sexes-irnt.tsv.bgz
Whole-body fat mass	454,137	9,851,867	European	Pan UKBB	0.9999772	https://pan-ukb-us-east-1.s3.amazonaws.com/sumstats_flat_files/continuous-23100-both_sexes-irnt.tsv.bgz
Arm fat-free mass (right)	454,753	9,851,867	European	Pan UKBB	0.9963929	https://pan-ukb-us-east-1.s3.amazonaws.com/sumstats_flat_files/continuous-23121-both_sexes-irnt.tsv.bgz
Leg fat-free mass (right)	454,835	9,851,867	European	Pan UKBB	0.9986165	https://pan-ukb-us-east-1.s3.amazonaws.com/sumstats_flat_files/continuous-23113-both_sexes-irnt.tsv.bgz
Trunk fat-free mass	454,508	9,851,867	European	Pan UKBB	0.9973235	https://pan-ukb-us-east-1.s3.amazonaws.com/sumstats_flat_files/continuous-23129-both_sexes-irnt.tsv.bgz
Whole-body fat-free mass	454,850	9,851,867	European	Pan UKBB	0.9987497	https://pan-ukb-us-east-1.s3.amazonaws.com/sumstats_flat_files/continuous-23101-both_sexes-irnt.tsv.bgz

BMI: body mass index; WC: waist circumference; WHR: waist-to-hip ratio; SNP: single nucleotide polymorphism; Pan UKBB: Pan-UKBiobank; GIANT: Genetic Investigation of Anthropometric Traits.

### Mendelian randomization analysis

The inverse variance weighted (IVW), the IVW with multiplicative random effects (IVW_mre), the maximum likelihood, the Mendelian randomization regression, and the Mendelian randomization pleiotropy residual sum and outlier (MR-PRESSO) methods were employed to evaluate the causal associations between body composition indicators and the risk of HF. The IVW method was used as the major analysis method in this study. The inverse variance weighting (IVW) method employs a meta-analysis approach that integrates Wald estimates from each SNP result to derive an unbiased overall estimate, while IVW_mre addresses potential heterogeneity more flexibly by introducing a random-effects model, allowing it to provide more robust and accurate causal estimates in the presence of heterogeneity^15^. We also used maximum likelihood as a sensitivity analysis to further validate the robustness of the main analysis through their respective strengths. The maximum likelihood method provides an estimate of the causal effect, which has the advantage of being able to addresses uncertainty in the model in a more flexible manner, providing us with unbiased and robust estimates^16^. The MR Egger regression, can be adapted to test for bias from pleiotropy, and the slope coefficient from Egger regression provides an estimate of the causal effect. In addition, the MR-Egger intercept test, if the p-value is below 0.05, it indicates the presence of horizontal pleiotropy^17–19^. The MR-PRESSO analysis detects and attempts to reduce horizontal pleiotropy by removing significant outliers. Odds ratio (OR) and 95% confidence interval (CI) were calculated. The leave-one-out analysis was employed to assess whether the results were caused by any single SNP associated with body composition indicators, and the symmetry in the resulting figure represents no pleiotropy. For testing the results, Cochran’s Q-test was conducted to evaluate the statistical heterogeneity between SNPs in the IVW method, and *P* < 0.05 was set as significantly heterogeneous. All statistical analyses were conducted using the“TwoSampleMR”package in R version 4.3.2 for MR analysis.

## Results

### Causal relationships between the three common anthropometries measurements and HF

**Fig. 1:**
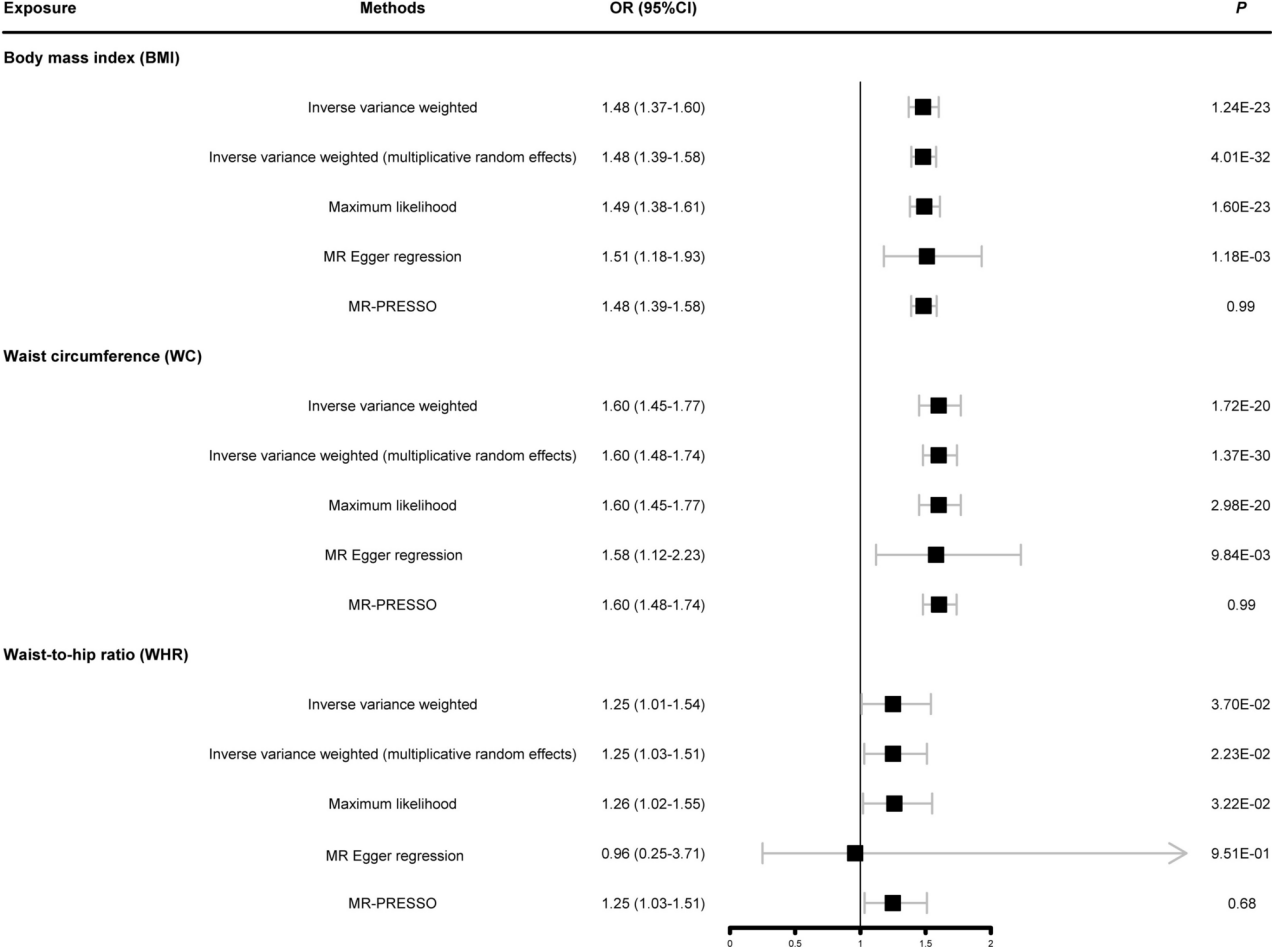
Summary Mendelian randomization estimates of the associations of body mass index/waist circumference/waist-to-hip ratio with heart failure incidence. OR: odds ratio; 95% CI: 95% confidence interval; MR-PRESSO: Mendelian randomization pleiotropy residual sum and outlier.

Figure 1 shows the results of a two-sample MR analysis identifying the causal relationships between the three common anthropometries measurements and HF. We found that BMI, WC and WHR were causal risk factors for HF. Specifically, as indicated by the IVW method, the odds ratio (OR) of HF increased per 1-SD increase in BMI (OR = 1.48, 95% CI: 1.37–1.60, P = 1.24E-23), WC (OR = 1.60 95% CI: 1.45–1.77, P = 1.72E-20), and WHR (OR = 1.25 95% CI: 1.01–1.54, P = 3.70E-02). Notably, WHR, the weighted median methods yielded negative results; however, the direction of effect remained consistent with an increased risk of HF (Supplementary Table S12). Furthermore, we conducted heterogeneity tests on the two-sample MR analysis, revealing Cochran’s Q test P-values of 1.000, 1.000, and 0.687 for BMI, WC, and WHR, which showed no significant heterogeneity (Table 1). In supplementary Figure S1, the positive associations between BMI, WC, WHR, and HF were shown in the inverse variance weighted, maximum likelihood, and MR-PRESSO, reinforcing the inference of causal relationships between these anthropometric measurements and HF.

### Causal relationships of body fat mass and HF

**Fig. 2:**
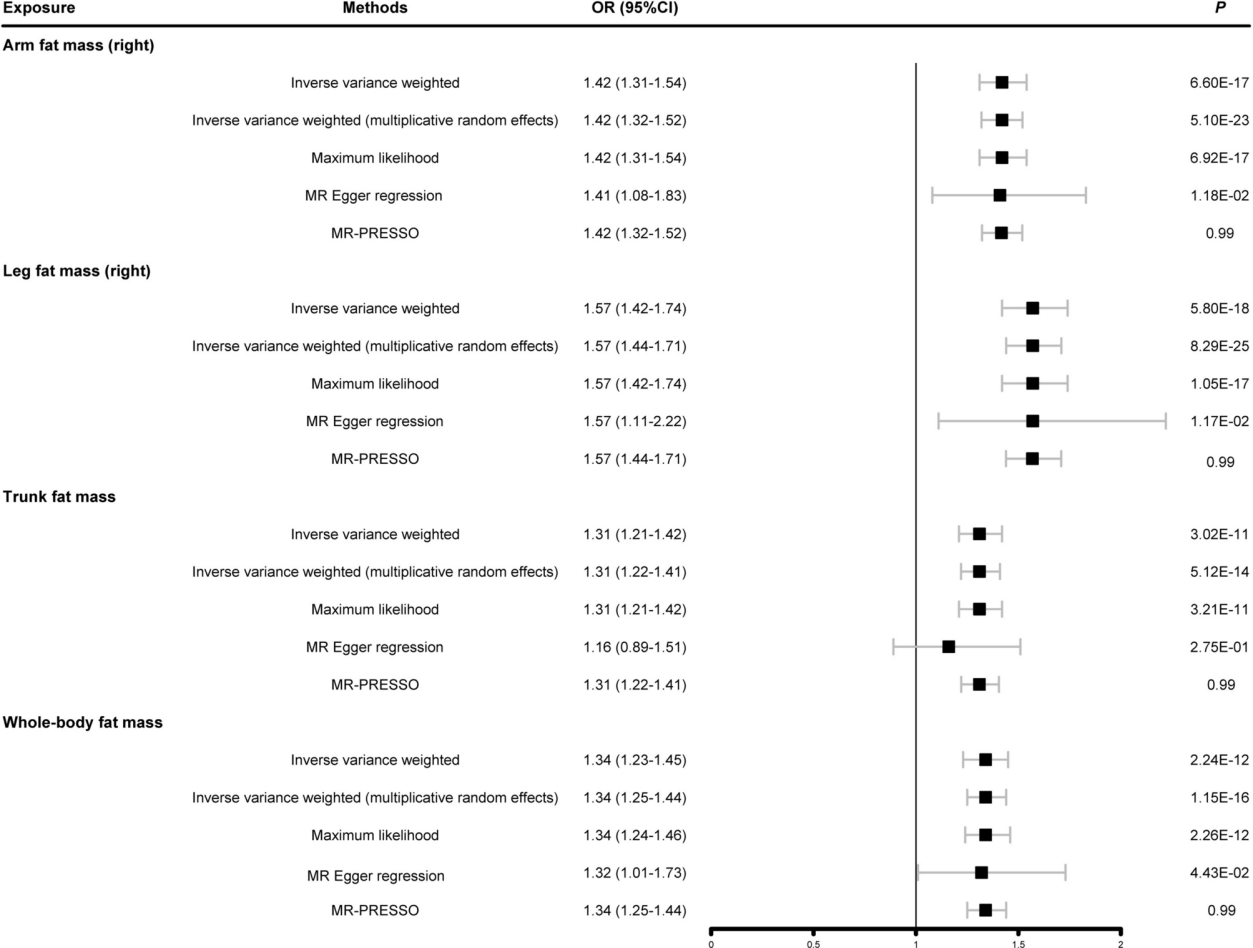
Summary Mendelian randomization estimates of the associations of body fat mass with HF. OR: odds ratio; 95% CI: 95% confidence interval; MR-PRESSO: Mendelian randomization pleiotropy residual sum and outlier.

The IVW analysis showed that right arm fat mass (OR = 1.42, 95% CI: 1.31–1.54, *P* = 6.60E-17), right leg fat mass (OR = 1.57, 95% CI: 1.42–1.74, *P* = 5.80E-18), trunk fat mass (OR = 1.31, 95% CI: 1.21–1.42, *P* = 3.02E-11) and whole-body fat mass (OR = 1.34, 95% CI: 1.23–1.45, *P* = 2.24E-12) were all shown as significant causal risk factors for HF (Fig. 2). The weighted median method yielded positive results, with the direction of effect consistent with an increased risk of HF (Table S12). Additionally, Cochran’s Q heterogeneity test results for the IVW method yielded p-values of 1.000, indicating a high degree of consistency in effect size estimates for each SNP (Table 1). The scatter plots depicted the positive associations of right arm fat mass, right leg fat mass, trunk fat mass, whole-body fat mass, with HF indifferent methods (Supplementary Figure S2).

### Causal relationships of body fat-free mass and HF

**Fig. 3:**
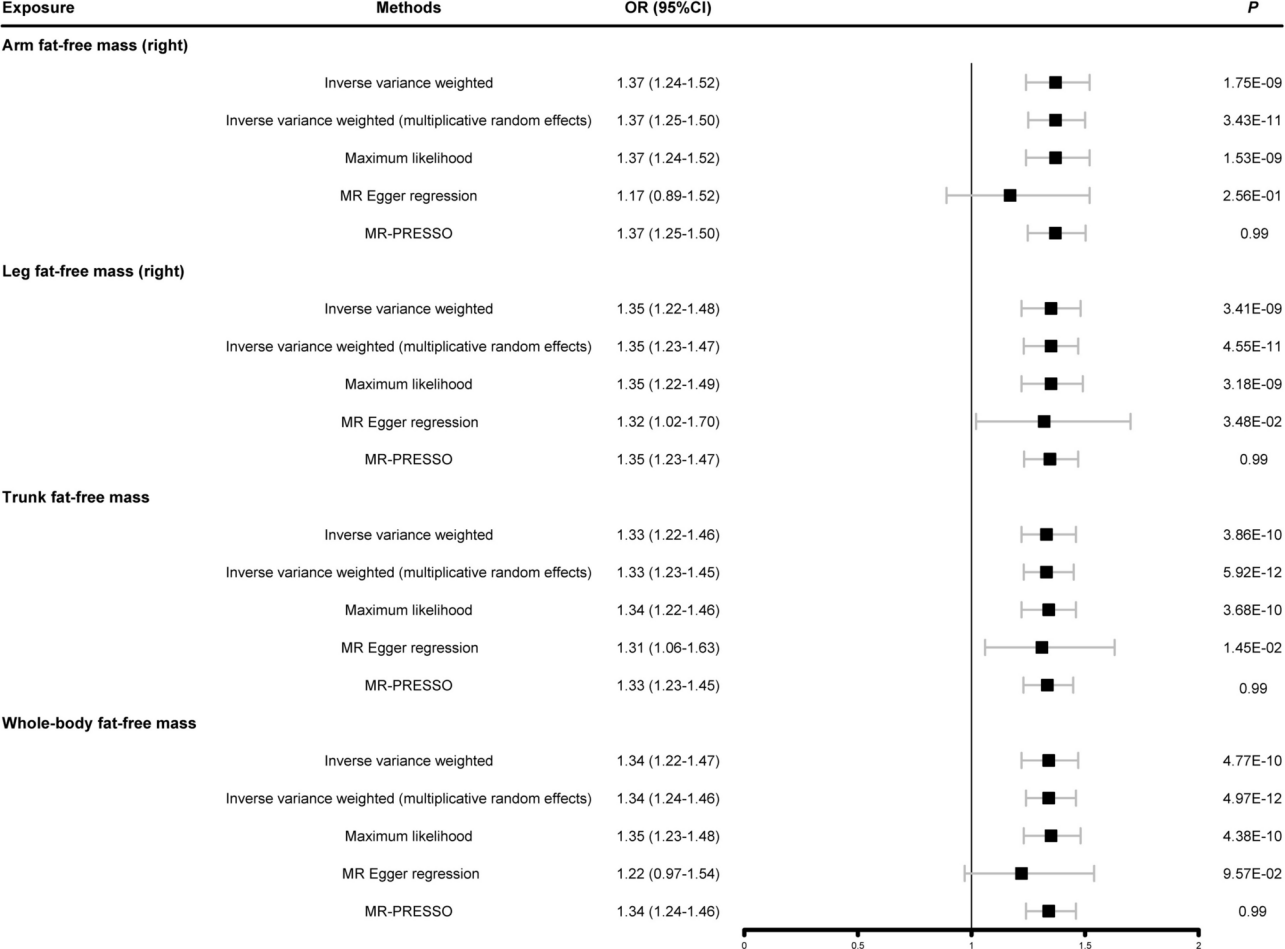
Summary Mendelian randomization estimates of the associations of body fat-free mass with HF. OR: odds ratio; 95%CI: 95% confidence interval; MR-PRESSO: Mendelian randomization pleiotropy residual sum and outlie.

Figure 3 showed the results of a two-sample MR analysis identifying the causal relationships between four fat-free masses and HF. We observed that each SD increase of right arm fat-free mass (OR = 1.37, 95% CI: 1.24–1.52, *P* = 1.75E-09), right leg fat-free mass (OR = 1.35, 95% CI: 1.22–1.48, *P* = 3.41E-09), trunk fat-free mass (OR = 1.33, 95% CI: 1.22–1.46, *P* = 3.86E-10) and whole body fat-free mass (OR = 1.34, 95% CI: 1.22–1.47, *P* = 4.77E-10) were positively associated with the risk of HF. The weighted median method yielded positive results, with the direction of effect consistent with an increased risk of HF (Supplementary Table S12). The scatter plots of the MR analysis results are shown in Supplementary Figure S3.

### Sensitivity analysis

The results of the leave-one-out analysis consistently demonstrated significant causal relationships between BMI, WC, WHR, right arm fat mass, right leg fat mass, trunk fat mass, whole-body fat mass, right arm fat-free mass, right leg fat-free mass, trunk fat-free fat mass, and whole-body fat-free fat mass and HF (shown in Supplementary Figures S4 to S6), indicating the robustness of the causal relationship between body composition and HF risk. Furthermore, the MR-PRESSO’s global test results indicated that all causal relationships in this study exhibit no horizontal pleiotropy (*P* > 0.05). Additionally, the MR-Egger intercept test did not reveal any horizontal pleiotropy (Supplementary Table S13).

## Discussion

Based on different large-scale population studies with GWAS involving the UK Biobank, GIANT, and FinnGen databases, the present study applied two-sample MR analysis to explore the causal relationship between body composition and the risk of HF. The results revealed that increases in BMI, WC, and WHR were associated with an increased risk of HF, consistent with prior observational studies^20–22^. Additionally, right arm fat mass, right leg fat mass, trunk fat mass, and whole-body fat mass were identified as causal risk factors for HF. Interestingly, our MR analysis suggests that indicators of fat-free mass, such as right arm fat-free mass, right leg fat-free mass, trunk fat-free mass, and whole-body fat-free mass, were also identified as causal risk factors for HF. These results provide evidence that both fat and fat-free mass in the whole body and specific body parts have adverse effects on the development of HF.

Consistent with previous MR studies, we observed a strong positive causal relationship between BMI and HF, with a 48% increase in the risk of developing HF for per standard deviation increase in BMI^23^. A MR study based on data from 19,384 individuals in the ENGAGE consortium showed that a genetically higher BMI is associated with an increased risk of HF^24^. Similarly, a meta-analysis of three prospective cohort studies showed that compared with normal weight, individuals with overweight and class 1 obesity have 38% and 56% increased risk of HF^25^. As indicators of central obesity, WC and WHR have also been shown to be causally associated with the risk of HF, in our study^26^. A recent meta-analysis revealed that the risk of HF increases by 28% with each 10 cm increase in WC, which is consistent with the results of our study^27^. These findings suggest that both overall and central obesity are causal risk factors for the development of HF.

In this study, we found causal relationships between overall, central, and localized fat mass and the risk of HF. Our study expands the understanding of how fat distribution in different body regions might contribute to HF. A previous meta-analysis had suggested that excessive accumulation of whole-body fat mass may increase the risk of HF^27^. Similarly, a recent MR study has also consistent with our findings, indicating that whole-body fat mass is a causal risk factor for heart failure^23^. The possible explanation is that whole-body fat mass leads to HF by increasing blood volume, raising blood pressure, elevating filling pressure, and activating the renin–angiotensin–aldosterone system within the cardiovascular system^28^. Trunk fat mass, as one of the indicators of central obesity, was found as a risk factor for HF in this study. Currently, trunk fat mass is considered to be associated with conditions such as hypertension, dyslipidemia, and diabetes, which may further lead to the occurrence of HF^29^. Moreover, trunk fat mass might involve in the formation of atherosclerotic plaques in the coronary arteries, followed by left ventricular remodeling, reduced systolic function, and ultimately leading to heart failure^30–33^. Inconsistent with our findings, a prospective cohort study indicated a negative correlation between leg fat mass and HF, while no association was found between arm fat mass and HF^34^. One possible reason for this discrepancy is that the study used Bioelectrical Impedance Analysis to measure leg and arm fat mass, and this method may be influenced by hydration status, food intake, and skin temperature fluctuations^35^. This could have led to inaccurate measurements of leg and arm fat mass, potentially explaining the negative correlation and no association observed in the cohort study. Therefore, more precise measurement techniques, such as MRI, are necessary for more comprehensive research. Further research is also needed to confirm the role and mechanisms of limbs fat mass in the development of heart failure. These findings underscore the complex role of overall, central, and localized obesity in heart failure, suggesting that interventions targeting heart failure should not only focus on overall fat mass but also consider the damage of central and localized fat mass.

We also observed causal relationships between several fat-free mass indicators and HF, including right arm fat-free mass, right leg fat-free mass, trunk fat-free mass and whole-body fat-free mass. A previous prospective study also suggested an association between higher whole-body fat-free mass and increased risk of HF^9^. All fat-free mass indicators were measured using dual energy X-ray absorptiometry (DEXA) method, but fat-free mass reflects not only skeletal muscle mass but also visceral organs, non-fat soft tissue, fibrous tissue, bone mineral mass and extracellular water^36^. As one part of nonfat tissue, increased fibrosis might increase the risk of HF development^37^. Additionally, compared to fat mass, muscle mass with the same quality imposes greater demands on the circulation of the heart, which might contribute to a higher burden of heart and then the failure of heart^37^. Moreover, increased extracellular water and subclinical oedema could potentially explain the remaining excess risk of HF associated with higher fat-free mass. Indeed, in population studies, there is an inverse association between skeletal muscle mass and cardiovascular outcomes while increased extracellular fluid is associated with higher risk of cardiovascular outcomes^38–40^. Further studies are required to explore the possible mechanisms of fat-free mass on the development of HF and to control the increasing prevalence of HF, ultimately reducing the heavy burden of HF.

### Strengths and limitations

This study investigated a more comprehensive set of anthropometric measurements than did previous studies, using two-sample MR for the first time to explore the associations between regional fat mass of human body and fat-free mass and the risk of HF. We conducted a MR analysis using validated SNPs to meet the core principles of MR. First, to fulfill the first assumption of MR analysis, we used genetic variants that had been previously linked to smoking exposure in large studies or identified through de novo analysis, ensuring they meet the genome-wide significance threshold (*p* < 5 × 10^−8^). Second, we employed methods such as MR-PRESSO and the MR-Egger intercept test, which detected no horizontal pleiotropy or outlier SNPs, meeting the second and third assumptions. Overall, our study results are unlikely to be affected by violations of MR assumptions. However, several limitations exist in this study. First, the GWAS summary data used in this study were obtained from European populations, and thus the results may not be generalizable to other ethnic groups and require validation in diverse populations. Second, the GWAS dataset used for heart failure outcomes in this study comprised 218,208 participants. Future studies should incorporate larger GWAS datasets to enhance statistical power and improve the robustness of findings. Third, the use of summary-level data does not permit detailed exploration of associations with specific heart failure subtypes, such as heart failure with preserved ejection fraction (HFpEF) and heart failure with reduced ejection fraction (HFrEF). This limitation arises because the UK biobank aggregate data lack detailed information on heart failure subtypes. Future MR studies should leverage individual-level data to thoroughly investigate these subtype-specific associations. Additionally, the summary-level data were not stratified by sex, age, or weight, limiting our ability to evaluate how these factors might influence the relationship between fat distribution and heart failure risk. Stratification by these variables could reveal important differences and nuances in how body composition affects heart failure in different demographic groups.

## Conclusion

Our study revealed a causal relationship between body composition and HF by two-sample MR. Considering the significant role of obesity in the pathogenesis of heart failure, our findings underscore the critical importance of incorporating body composition into heart failure prevention strategies. Therefore, for more effective prevention of heart failure, comprehensive management of fat and fat-free weight is necessary to reduce the global burden of HF.

## Supplementary Information

Supplementary Information.

## Data Availability

The data that support the findings of this study are available from the corresponding author upon reasonable request.

## Abbreviations

GIANT: Genetic Investigation of Anthropometric Traits
IVW: Inverse variance weighted
IVW_mre: Inverse variance weighted with multiplicative random effects
