# Supplementary material for: The causal relationships between body composition and heart failure: A two-sample mendelian randomization study
Source: Sci Rep. 2025 May 2;15:15434. doi: 10.1038/s41598-025-00406-7 (PMC12048550; doi:10.1038/s41598-025-00406-7)
Supplement: Supplementary file 1 — Supplementary Information. [file 41598_2025_406_MOESM1_ESM.docx]

## The Causal Relationships Between Adiposity Measurement and Heart failure : A Mendelian Randomization Study

**Table of contents**

**Study design** for this two-sample Mendelian randomization study on the association of body composition with heart failure.

**Figure S1.** Scatter plots visualizing the Mendelian randomization (MR) estimates of 3 anthropometries measurements with HF ((A): body mass index; (B): waist circumference; (C): waist-to-hip ratio).

**Figure S2.** Scatter plots visualizing the Mendelian randomization (MR) estimates of body fat mass with HF ((A): arm fat mass (right); (B): leg fat mass (right); (C): trunk fat mass; (D): whole-body fat mass).

**Figure S3.** Scatter plots visualizing the Mendelian randomization (MR) estimates of body fat-free mass with HF ((A): arm fat-free mass (right); (B): leg fat-free mass (right); (C): trunk fat-free mass; (D): whole-body fat-free mass).

**Figure S4.** Forest diagram for leave-one-out of the association between body mass index/ waist circumference / waist-to-hip ratio and heart failure.

**Figure S5.** Forest diagram for leave-one-out of the association between body fat mass and heart failure.

**Figure S6.** Forest diagram for leave-one-out of the association between body fat-free mass and heart failure.

**Table S1**. Genetic instruments used in the analyses for the association of BMI with Heart failure.

**Table S2**. Genetic instruments used in the analyses for the association of Waist circumference with Heart failure.

**Table S3**. Genetic instruments used in the analyses for the association of Waist to ratio with Heart failure.

**Table S4**. Genetic instruments used in the analyses for the association of Arm fat mass(right) with Heart failure.

**Table S5**. Genetic instruments used in the analyses for the association of Leg fat mass (right) with Heart failure.

**Table S6**. Genetic instruments used in the analyses for the association of Trunk fat mass with Heart failure.

**Table S7**. Genetic instruments used in the analyses for the association of Whole body fat mass with Heart failure.

**Table S8**. Genetic instruments used in the analyses for the association of Arm fat-free mass(right) with Heart failure.

**Table S9**. Genetic instruments used in the analyses for the association of Leg fat-free mass with Heart failure.

**Table S10**. Genetic instruments used in the analyses for the association of Trunk fat-free mass with Heart failure.

**Table S11**. Genetic instruments used in the analyses for the association of Whole fat-free mass with Heart failure.

**Table S12.** The results of the horizontal pleiotropy analysis.

**Table S13.** The results of the weighted median analysis.

**Table S14.** Multivariate MR of Whole body fat-free mass, Whole body fat mass and Heart Failure.

**Table S15.** Multivariate MR of Whole body fat-free mass, BMI and Heart Failure.


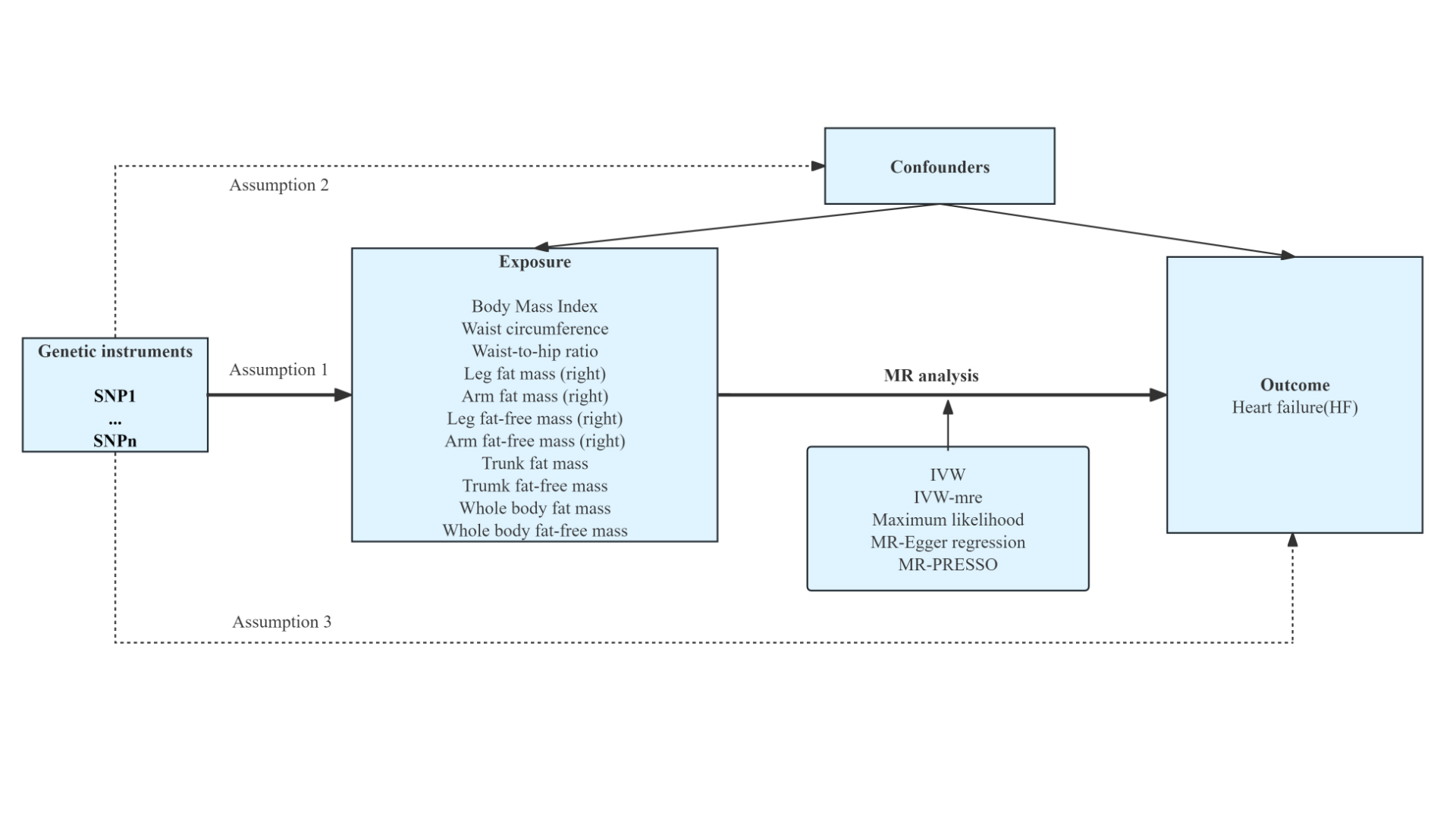
 **Study design** for this two-sample Mendelian randomization study on the association of body composition with heart failure. SNP: single-nucleotide polymorphism; MR: Mendelian randomization; IVW: inverse variance weighted; IVW_mre: inverse variance weighted with multiplicative random effects; MR-PRESSO: Mendelian randomization pleiotropy residual sum and outlier.


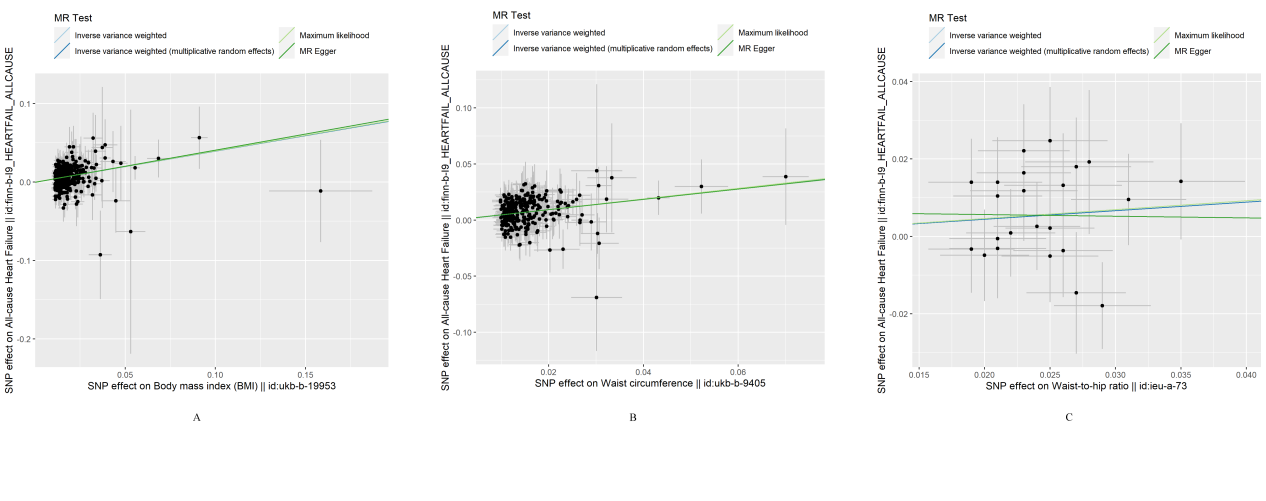
**Figure S1.** Scatter plots visualizing the Mendelian randomization (MR) estimates of 3 anthropometries measurements with HF ((A): body mass index; (B): waist circumference; (C): waist-to-hip ratio).


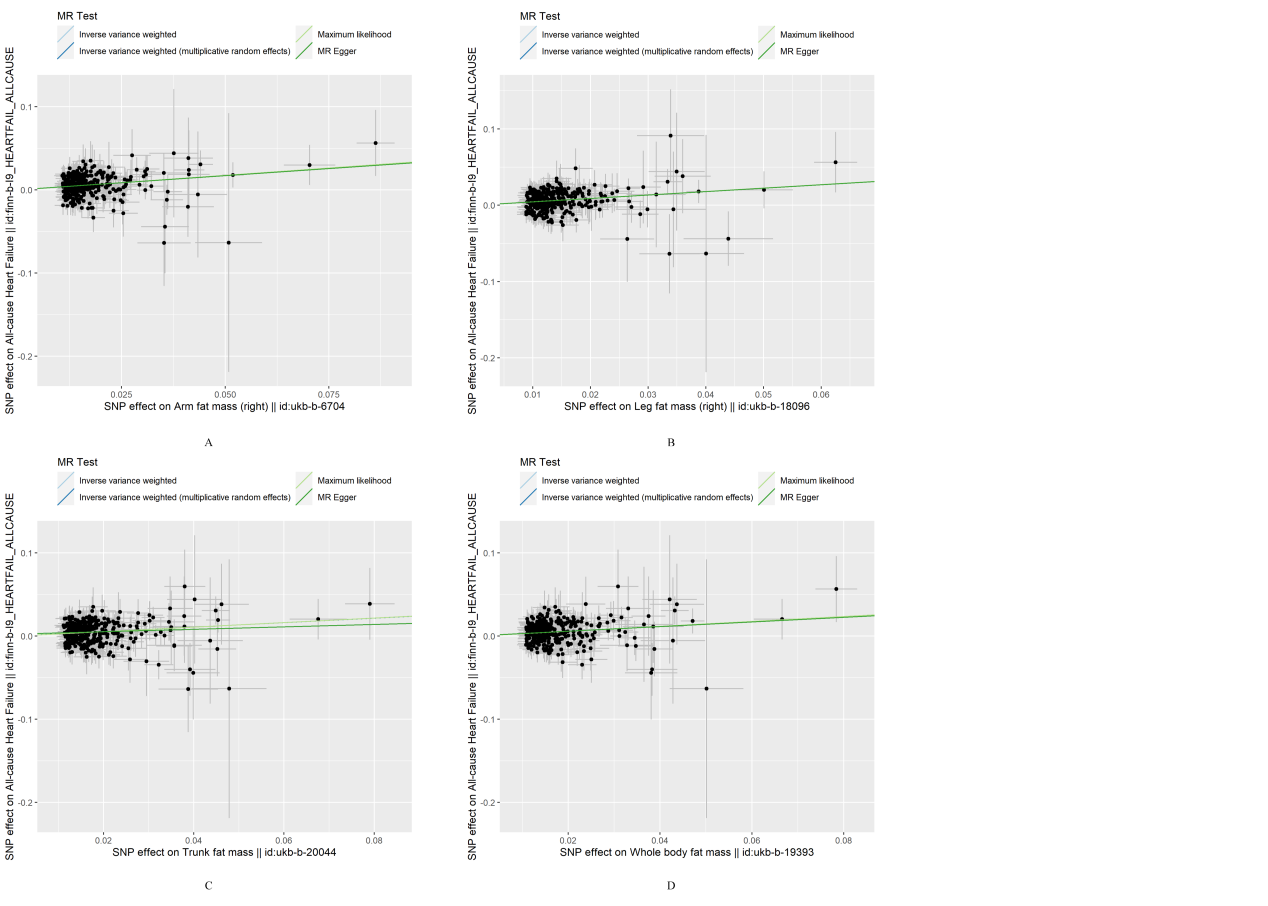


**Figure S2.** Scatter plots visualizing the Mendelian randomization (MR) estimates of body fat mass with HF ((A): arm fat mass (right); (B): leg fat mass (right); (C): trunk fat mass; (D): whole-body fat mass).


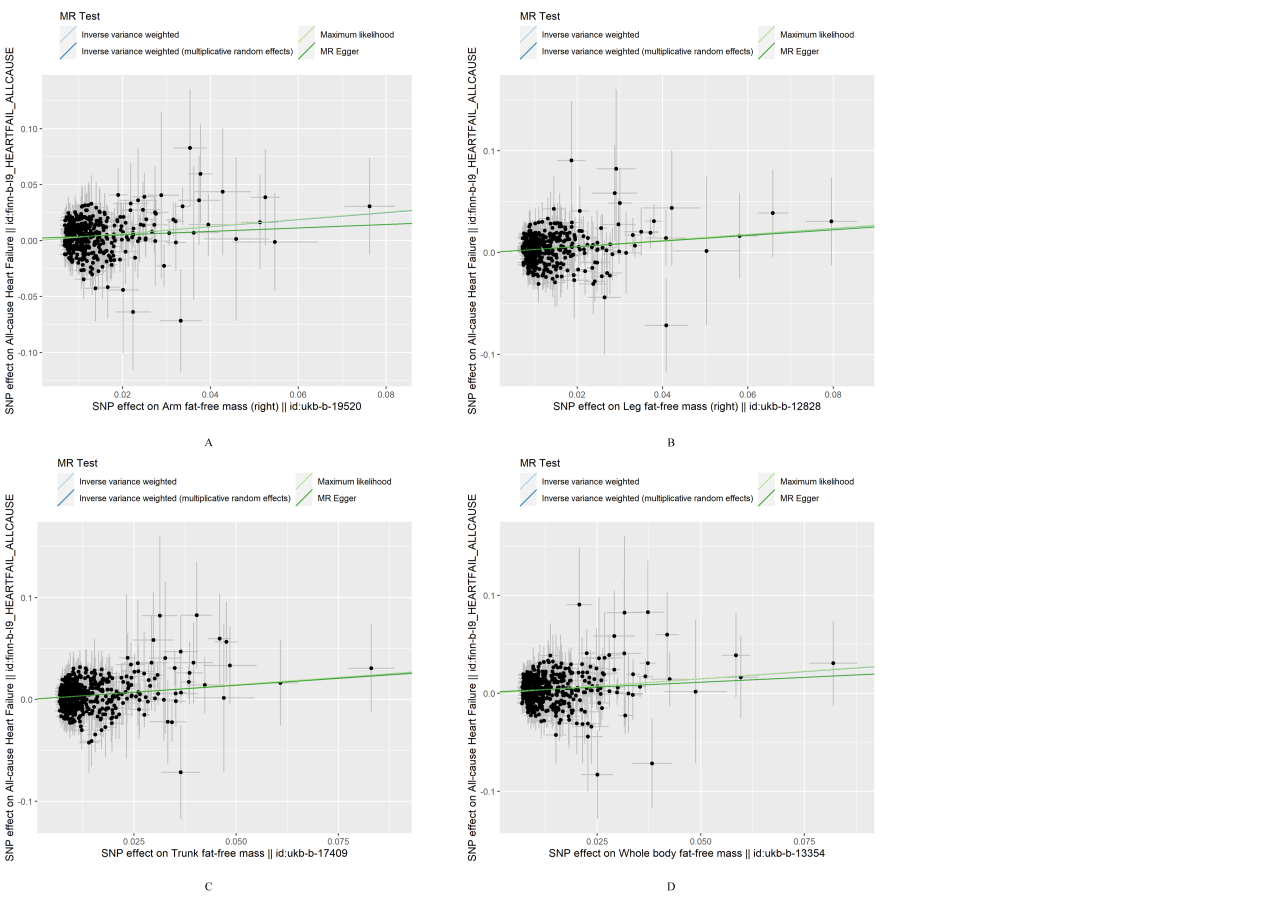


**Figure S3.** Scatter plots visualizing the Mendelian randomization (MR) estimates of body fat-free mass with HF ((A): arm fat-free mass (right); (B): leg fat-free mass (right); (C): trunk fat-free mass; (D): whole-body fat-free mass).


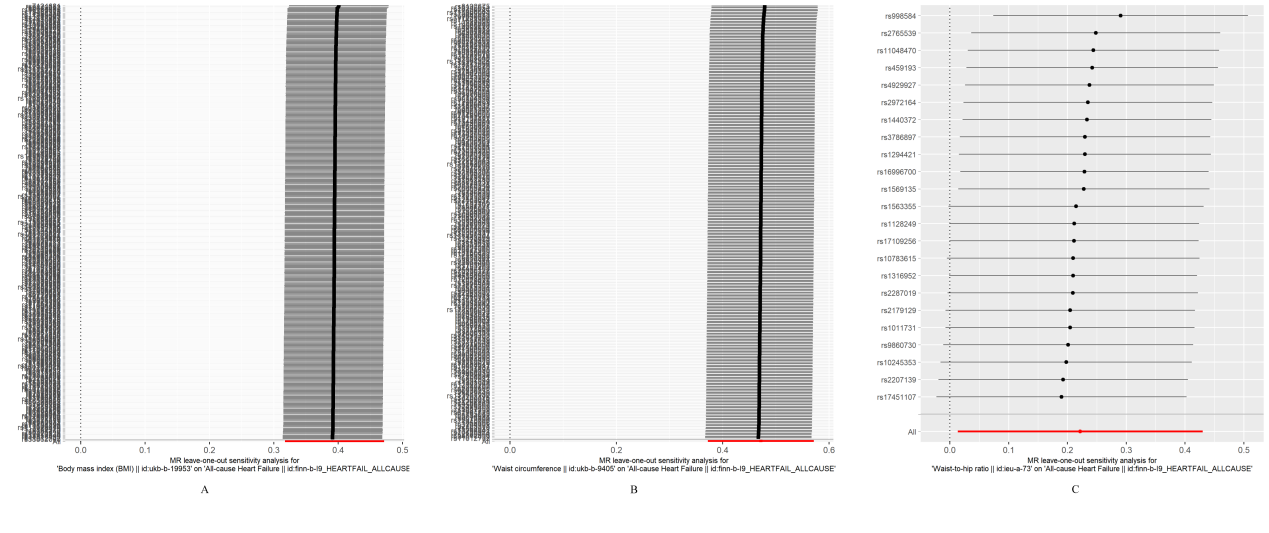


**Figure S4.** Forest diagram for leave-one-out of the association between body mass index/ waist circumference / waist-to-hip ratio and heart failure. ((A): body mass index; (B): waist circumference; (C): waist-to-hip ratio.


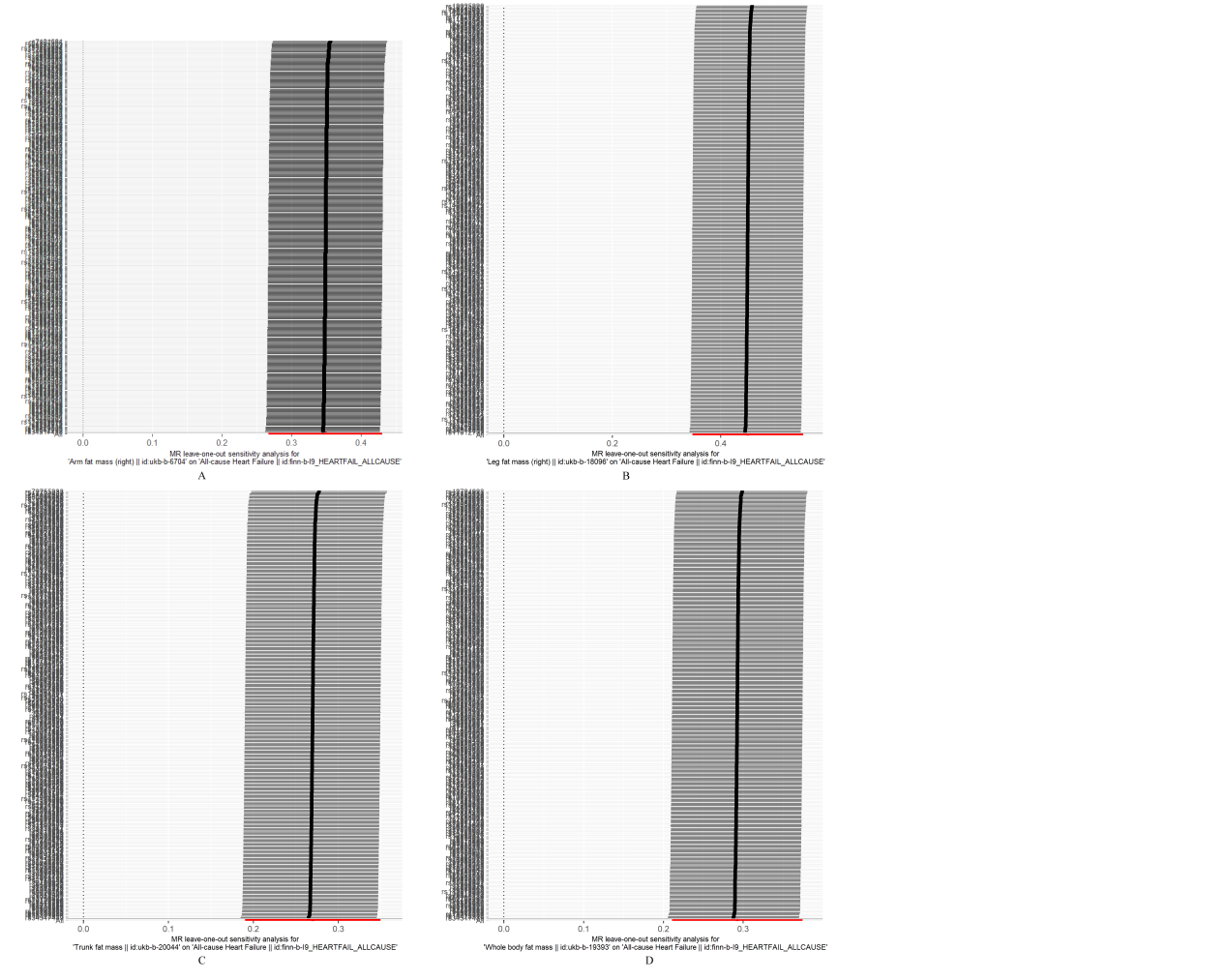


**Figure S5.** Forest diagram for leave-one-out of the association between body fat mass and heart failure. ((A): arm fat mass (right); (B): leg fat mass (right); (C): trunk fat mass; (D): whole-body fat mass).


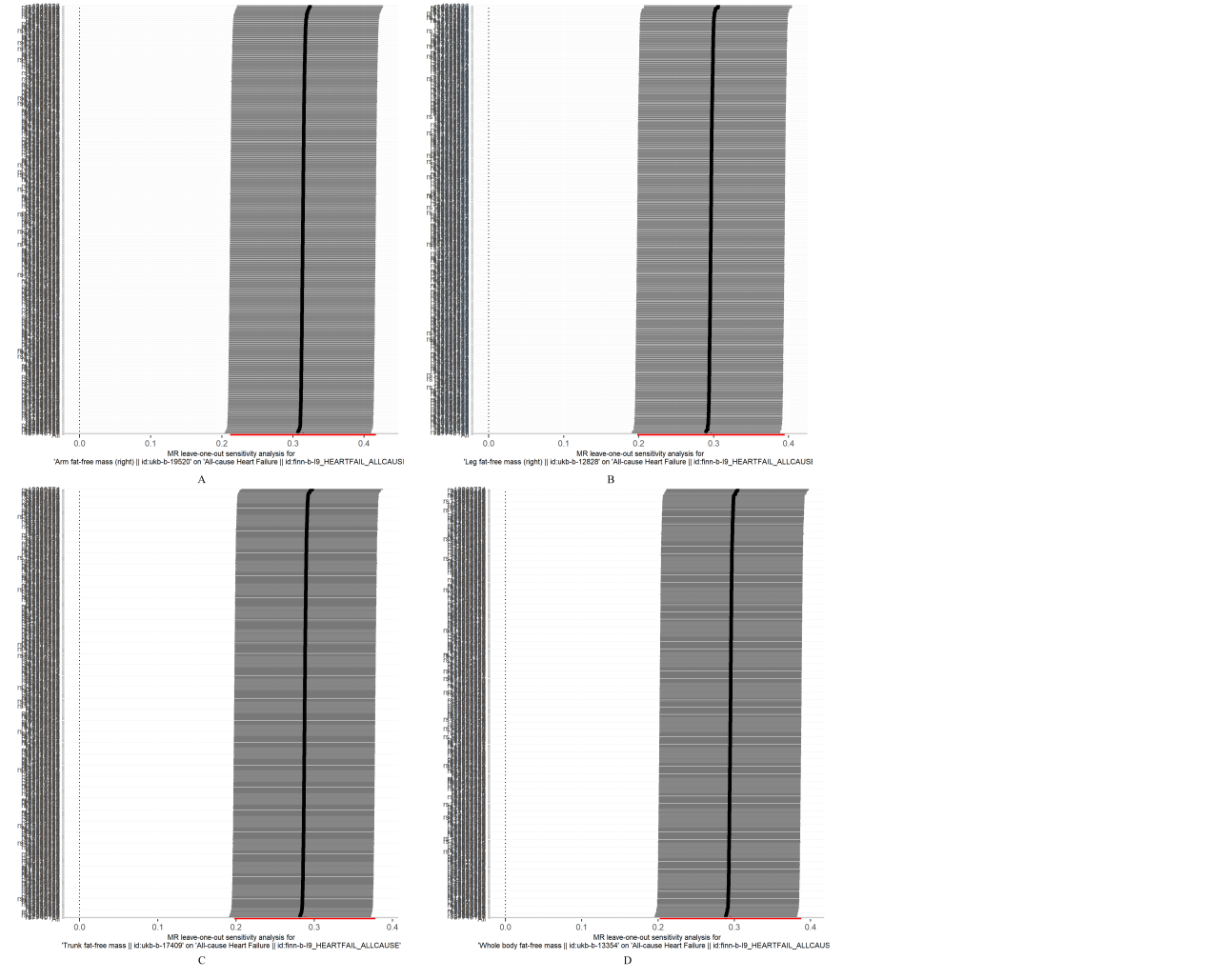


**Figure S6.** Forest diagram for leave-one-out of the association between body fat-free mass and heart failure.((A): arm fat-free mass (right); (B): leg fat-free mass (right); (C): trunk fat-free mass; (D): whole-body fat-free mass).

**Table S1**. Genetic instruments used in the analyses for the association of BMI with Heart failure.

| SNP | EA | OA | chr | SE | *P* value | F |
| --- | --- | --- | --- | --- | --- | --- |
| rs10063055 | T | C | 5 | 0.00226991 | 1.7E-09 | 36.30748534 |
| rs10160769 | C | G | 11 | 0.00242199 | 1.2E-10 | 41.41291473 |
| rs10182416 | G | A | 2 | 0.00197134 | 3.69999E-11 | 43.74068309 |
| rs10184537 | T | C | 2 | 0.00208355 | 5.10035E-15 | 61.22477578 |
| rs10402950 | C | T | 19 | 0.00218692 | 5.69994E-10 | 38.41503305 |
| rs10423928 | A | T | 19 | 0.00249869 | 3.40017E-42 | 185.3021434 |
| rs10505836 | C | A | 12 | 0.00287086 | 1.2E-10 | 41.45907232 |
| rs10510025 | T | C | 10 | 0.00229886 | 2.19989E-14 | 58.37631077 |
| rs1064213 | A | G | 2 | 0.00197196 | 3.69999E-14 | 57.32304216 |
| rs10742752 | C | T | 11 | 0.00202961 | 6.1E-09 | 33.78969203 |
| rs10760277 | T | C | 9 | 0.00203825 | 1E-11 | 46.32878537 |
| rs10771041 | T | C | 12 | 0.0030884 | 5.00035E-12 | 47.66962622 |
| rs10780248 | A | G | 9 | 0.00199415 | 1.2E-09 | 36.95888785 |
| rs1078141 | T | C | 8 | 0.00205862 | 4.90004E-12 | 47.74028477 |
| rs10824211 | T | C | 10 | 0.00286631 | 4.30031E-13 | 52.50207773 |
| rs10927006 | C | T | 1 | 0.00281467 | 2.30001E-09 | 35.69190823 |
| rs11001963 | T | C | 10 | 0.00201921 | 7.29995E-09 | 33.45631376 |
| rs11009685 | T | C | 10 | 0.00230766 | 1.40001E-08 | 32.1438279 |
| rs11012732 | G | A | 10 | 0.00210126 | 7.10068E-25 | 106.0853139 |
| rs11057072 | G | A | 12 | 0.00233829 | 0.00000004 | 30.16352643 |
| rs11079849 | T | C | 17 | 0.00211163 | 1.80011E-21 | 90.54280019 |
| rs11099020 | T | C | 4 | 0.00206202 | 5.60015E-12 | 47.44859427 |
| rs11115160 | A | G | 12 | 0.00233641 | 2.19999E-08 | 31.32599328 |
| rs11122450 | G | T | 1 | 0.00202425 | 9.09997E-09 | 33.01798924 |
| rs11134679 | G | A | 5 | 0.00213286 | 1.20005E-17 | 73.18227087 |
| rs111598585 | T | C | 4 | 0.00243798 | 5.39995E-09 | 34.04189612 |
| rs11161044 | C | G | 14 | 0.00251308 | 1.59993E-11 | 45.3554344 |
| rs11165643 | T | C | 1 | 0.00200337 | 4.90004E-22 | 93.11652234 |
| rs111689389 | C | G | 5 | 0.00219522 | 4.70002E-10 | 38.78100421 |
| rs11218510 | A | G | 11 | 0.00202121 | 8.4004E-13 | 51.19290957 |
| rs1126930 | C | G | 12 | 0.00535892 | 1.7E-09 | 36.26017744 |
| rs113079574 | T | C | 4 | 0.00251598 | 5.80003E-10 | 38.39331175 |
| rs113603865 | T | C | 1 | 0.00242689 | 1.80011E-14 | 58.74709688 |
| rs113624107 | A | G | 14 | 0.00236876 | 2.1E-10 | 40.34649072 |
| rs11525873 | C | T | 7 | 0.00333603 | 6.59933E-13 | 51.66181254 |
| rs11607476 | C | A | 11 | 0.00199179 | 3.10027E-15 | 62.22656754 |
| rs11608710 | G | T | 12 | 0.00415281 | 2.19999E-09 | 35.80388993 |
| rs11610621 | A | T | 12 | 0.00278318 | 3.09999E-09 | 35.09097359 |
| rs11630647 | A | G | 15 | 0.00228082 | 2.69998E-08 | 30.87773108 |
| rs116374395 | A | G | 5 | 0.00536236 | 2.80001E-09 | 35.34850152 |
| rs11642090 | C | T | 16 | 0.00205925 | 2.90001E-08 | 30.74626431 |
| rs11656076 | A | G | 17 | 0.00237054 | 7.59976E-11 | 42.35969592 |
| rs1167311 | A | G | 1 | 0.00213109 | 1.59993E-19 | 81.72029687 |
| rs11691869 | A | C | 2 | 0.00205562 | 5.60015E-21 | 88.295736 |
| rs11699828 | A | G | 20 | 0.00582677 | 8.60003E-09 | 33.13728928 |
| rs11709402 | G | A | 3 | 0.00220796 | 4.90004E-25 | 106.8116015 |
| rs117118217 | C | G | 10 | 0.00788004 | 1.29999E-08 | 32.34891573 |
| rs11757278 | C | T | 6 | 0.00214834 | 9.8992E-12 | 46.35521025 |
| rs11778219 | G | A | 8 | 0.0026874 | 4.20001E-09 | 34.54853406 |
| rs118136827 | T | G | 17 | 0.0022019 | 1.7E-09 | 36.2555042 |
| rs1191600 | A | C | 14 | 0.00202687 | 1.7E-09 | 36.34706088 |
| rs12072739 | G | A | 1 | 0.00236691 | 3.29989E-11 | 44.00277583 |
| rs12089815 | A | G | 1 | 0.00198606 | 5.69994E-10 | 38.43081009 |
| rs12101393 | G | C | 15 | 0.00241172 | 2.49977E-11 | 44.51284057 |
| rs12140153 | T | G | 1 | 0.0034589 | 1.20005E-21 | 91.43736306 |
| rs12213441 | T | C | 6 | 0.0024103 | 2.5E-09 | 35.52808872 |
| rs12259464 | A | G | 10 | 0.00198686 | 4.49987E-11 | 43.40218381 |
| rs12273545 | T | C | 11 | 0.00428551 | 6.80002E-09 | 33.59492531 |
| rs1229984 | C | T | 4 | 0.00599385 | 4.60002E-10 | 38.84474227 |
| rs12340969 | T | C | 9 | 0.0019941 | 1.10002E-25 | 109.7109434 |
| rs12364470 | G | T | 11 | 0.0026658 | 4.90004E-13 | 52.25535941 |
| rs12440603 | T | C | 15 | 0.0020011 | 3.40017E-12 | 48.44747119 |
| rs12462975 | A | G | 19 | 0.00212071 | 2.60016E-20 | 85.26295796 |
| rs12541408 | C | T | 8 | 0.00212723 | 1.59993E-11 | 45.35519707 |
| rs1263629 | G | A | 2 | 0.00282352 | 4.49997E-10 | 38.88296658 |
| rs1266874 | G | A | 6 | 0.0020712 | 1E-11 | 46.32172012 |
| rs12681792 | A | C | 8 | 0.00251693 | 3.50002E-09 | 34.90107251 |
| rs12692596 | T | C | 2 | 0.00203738 | 1.29999E-10 | 41.26191705 |
| rs12696039 | G | A | 3 | 0.0027705 | 3.50002E-08 | 30.41877835 |
| rs12712767 | A | G | 2 | 0.00204487 | 2.30001E-08 | 31.20042495 |
| rs12881629 | G | A | 14 | 0.00358929 | 7.69999E-10 | 37.82478404 |
| rs12921986 | G | A | 16 | 0.0036983 | 3.79997E-08 | 30.22635862 |
| rs1296328 | C | A | 4 | 0.001999 | 3.90032E-21 | 89.03277151 |
| rs12974458 | T | C | 19 | 0.00199717 | 2.29985E-14 | 58.27951724 |
| rs13012070 | A | G | 2 | 0.00234795 | 6.1E-09 | 33.79823159 |
| rs13033310 | A | G | 2 | 0.0022826 | 3.29997E-08 | 30.52385807 |
| rs13097918 | A | T | 3 | 0.0024161 | 1.7E-09 | 36.29612403 |
| rs13107325 | T | C | 4 | 0.00375479 | 8.49963E-37 | 160.5741918 |
| rs13176429 | C | T | 5 | 0.00213122 | 3.10027E-11 | 44.11576982 |
| rs13218383 | G | C | 6 | 0.00209236 | 5.79963E-12 | 47.38086116 |
| rs1322842 | G | A | 6 | 0.00203539 | 1.09999E-10 | 41.60842293 |
| rs13248187 | C | T | 8 | 0.00224155 | 1.99986E-12 | 49.45793632 |
| rs1327259 | G | A | 6 | 0.00203363 | 2.80027E-13 | 53.34530198 |
| rs13291723 | A | G | 9 | 0.00199876 | 3.40001E-08 | 30.46291511 |
| rs13427822 | G | A | 2 | 0.00224142 | 5.60015E-16 | 65.58107849 |
| rs1360201 | T | C | 9 | 0.00197747 | 4.79954E-11 | 43.26810371 |
| rs1411432 | C | A | 9 | 0.0025491 | 3.69999E-17 | 70.91871768 |
| rs1438945 | A | T | 5 | 0.00219791 | 1.09999E-09 | 37.07218671 |
| rs1441264 | A | G | 13 | 0.00205854 | 3.40017E-18 | 75.63930984 |
| rs1451963 | T | G | 14 | 0.00360585 | 7.39997E-10 | 37.907903 |
| rs1458156 | T | C | 12 | 0.00197951 | 1.20005E-12 | 50.55701298 |
| rs145981104 | G | A | 8 | 0.00404552 | 0.00000002 | 31.51547154 |
| rs146569428 | A | G | 11 | 0.00248571 | 0.00000002 | 31.5103295 |
| rs1471093 | A | G | 3 | 0.00203996 | 4.10015E-11 | 43.54812058 |
| rs147568678 | C | T | 10 | 0.00233034 | 1.09999E-08 | 32.7338912 |
| rs1477290 | C | T | 5 | 0.00289815 | 2.19989E-31 | 135.8330873 |
| rs1503526 | C | T | 5 | 0.00197681 | 5.90065E-15 | 60.93222345 |
| rs156201 | C | G | 6 | 0.00228932 | 8.30004E-09 | 33.19479578 |
| rs1582931 | A | G | 5 | 0.0019956 | 2.29985E-11 | 44.69802889 |
| rs1609010 | G | A | 8 | 0.0019963 | 7.89951E-26 | 110.4199547 |
| rs16965658 | G | A | 15 | 0.00418404 | 5.69994E-09 | 33.93498828 |
| rs17132130 | C | G | 7 | 0.00238564 | 7.50067E-14 | 55.93292324 |
| rs17399739 | G | A | 10 | 0.00391006 | 4.40048E-12 | 47.9348508 |
| rs17446299 | G | C | 13 | 0.00266515 | 8.9E-09 | 33.05769979 |
| rs17544384 | C | T | 1 | 0.00241434 | 5.30005E-09 | 34.07345775 |
| rs17668356 | G | C | 3 | 0.00279318 | 1.50003E-16 | 68.12492436 |
| rs17724992 | G | A | 19 | 0.00224218 | 1.69981E-14 | 58.83902183 |
| rs17770336 | T | C | 9 | 0.00211161 | 1.29987E-30 | 132.354422 |
| rs1778830 | A | G | 1 | 0.00205596 | 7.50067E-12 | 46.88368617 |
| rs1793636 | C | G | 11 | 0.0021408 | 4.70002E-10 | 38.7961162 |
| rs1834144 | A | C | 18 | 0.00205213 | 8.60003E-12 | 46.61663554 |
| rs1861410 | T | C | 2 | 0.00198918 | 1.20005E-26 | 114.1843916 |
| rs1884897 | G | A | 20 | 0.00205634 | 2.29985E-22 | 94.6048873 |
| rs1919243 | C | T | 5 | 0.00200175 | 5.49997E-09 | 34.02209549 |
| rs1928706 | A | G | 9 | 0.00198846 | 1.7E-09 | 36.32625409 |
| rs1934102 | A | G | 9 | 0.00210374 | 8.19974E-11 | 42.21225187 |
| rs1990662 | C | A | 12 | 0.00251798 | 8.9E-09 | 33.05845819 |
| rs2035936 | T | G | 3 | 0.00435778 | 1.69981E-17 | 72.42631872 |
| rs2051559 | C | T | 4 | 0.00291963 | 2.80027E-12 | 48.85811911 |
| rs2075466 | C | G | 16 | 0.00223627 | 2.80001E-09 | 35.3200324 |
| rs2102278 | G | A | 4 | 0.0021139 | 0.00000002 | 31.46849616 |
| rs2133561 | T | A | 5 | 0.00204744 | 5.79963E-12 | 47.40575112 |
| rs213518 | C | T | 7 | 0.00280491 | 1.79999E-08 | 31.68789516 |
| rs2153740 | G | A | 20 | 0.00199313 | 0.000000016 | 31.8880134 |
| rs215634 | G | A | 7 | 0.00203492 | 2.39994E-14 | 58.185861 |
| rs2192158 | G | A | 4 | 0.00198302 | 3.69999E-14 | 57.30901219 |
| rs2193101 | G | C | 16 | 0.00256649 | 2.59998E-08 | 30.97869331 |
| rs2216931 | A | C | 2 | 0.00208587 | 5.19996E-16 | 65.72929392 |
| rs2234458 | T | C | 11 | 0.00205579 | 3.59998E-23 | 98.31631312 |
| rs2249825 | C | G | 13 | 0.00223793 | 6.4998E-11 | 42.6678485 |
| rs2271189 | A | G | 12 | 0.00201869 | 6.4998E-16 | 65.26706729 |
| rs2275003 | G | A | 9 | 0.00197803 | 1.7E-09 | 36.3369983 |
| rs2289379 | T | C | 7 | 0.00202952 | 5.19996E-14 | 56.65797623 |
| rs2307111 | C | T | 5 | 0.00202203 | 1.29987E-43 | 191.8099587 |
| rs2342892 | G | T | 16 | 0.00197715 | 1.29999E-10 | 41.24171177 |
| rs2381404 | C | T | 2 | 0.00229974 | 1.29999E-09 | 36.88174613 |
| rs2398861 | G | A | 9 | 0.00226733 | 2.09991E-15 | 62.97777976 |
| rs2425816 | A | G | 20 | 0.00201106 | 1.2E-09 | 36.97338532 |
| rs2433733 | A | G | 2 | 0.00210907 | 3.80014E-16 | 66.3574379 |
| rs2439823 | G | A | 10 | 0.00199108 | 5.30029E-22 | 92.98566249 |
| rs2482356 | C | T | 9 | 0.00199506 | 1.29999E-08 | 32.37158594 |
| rs2512892 | C | T | 11 | 0.00199819 | 9.20026E-11 | 41.98085853 |
| rs2568958 | A | G | 1 | 0.00201204 | 1.59993E-28 | 122.7597566 |
| rs2569993 | C | T | 3 | 0.00212202 | 2.39999E-09 | 35.64956904 |
| rs2606228 | C | A | 3 | 0.00208379 | 2.70023E-11 | 44.36236704 |
| rs2616143 | A | G | 8 | 0.00212547 | 6.70039E-11 | 42.6069945 |
| rs2678204 | G | T | 1 | 0.00208158 | 3.80014E-31 | 134.7235747 |
| rs2725371 | G | A | 8 | 0.00215845 | 1.10002E-13 | 55.15545577 |
| rs2781668 | T | C | 6 | 0.00265988 | 0.000000021 | 31.36493515 |
| rs2791643 | T | C | 1 | 0.00231332 | 7.19996E-09 | 33.47194593 |
| rs28350 | G | A | 3 | 0.00258234 | 2.90001E-12 | 48.7678044 |
| rs28366156 | C | T | 6 | 0.00292978 | 1.59993E-19 | 81.70548679 |
| rs2837996 | C | T | 21 | 0.00207859 | 1.09999E-09 | 37.12377624 |
| rs28404639 | T | C | 5 | 0.00205533 | 0.000000012 | 32.47624046 |
| rs28489620 | A | G | 22 | 0.00220025 | 2.80027E-12 | 48.82168281 |
| rs28537413 | T | A | 4 | 0.00216552 | 3.29997E-09 | 35.02519802 |
| rs2861685 | C | T | 2 | 0.00199763 | 1E-17 | 73.51107571 |
| rs28670671 | C | T | 9 | 0.00226426 | 3.69999E-08 | 30.30039947 |
| rs2870111 | T | C | 15 | 0.00201912 | 7.10068E-15 | 60.57731732 |
| rs2875762 | C | G | 6 | 0.0023121 | 3.29989E-11 | 44.01298943 |
| rs2899644 | T | C | 15 | 0.0023609 | 2.30001E-10 | 40.18375282 |
| rs2920503 | T | C | 3 | 0.00219666 | 1.7E-10 | 40.79744766 |
| rs2941452 | G | A | 8 | 0.00203237 | 0.00000004 | 30.16435535 |
| rs2962334 | T | G | 5 | 0.00703547 | 7.79992E-10 | 37.79987971 |
| rs3213943 | A | C | 2 | 0.00288163 | 4.79999E-10 | 38.76122492 |
| rs32421 | T | A | 5 | 0.00237757 | 2.59998E-08 | 30.97073683 |
| rs329118 | T | C | 5 | 0.0020038 | 1.29987E-16 | 68.40919499 |
| rs329651 | T | G | 11 | 0.00250202 | 3.29997E-10 | 39.48416705 |
| rs34153025 | C | T | 15 | 0.00678155 | 9.59997E-09 | 32.9289866 |
| rs34234296 | A | G | 2 | 0.00204032 | 2.39994E-13 | 53.66751615 |
| rs34481751 | A | C | 20 | 0.00270404 | 7.8001E-12 | 46.82236864 |
| rs34517439 | A | C | 1 | 0.00304983 | 3.59998E-37 | 162.2505083 |
| rs34696181 | C | T | 7 | 0.00198283 | 8.10009E-09 | 33.25549338 |
| rs349071 | A | G | 11 | 0.00198231 | 1.9002E-11 | 45.05727784 |
| rs35154326 | G | A | 16 | 0.00223442 | 5.30005E-09 | 34.07260904 |
| rs35364449 | T | C | 15 | 0.00318335 | 9.20026E-12 | 46.49720308 |
| rs35697587 | A | G | 14 | 0.00198067 | 9.20026E-17 | 69.12603486 |
| rs35697691 | G | C | 15 | 0.00352223 | 6.20012E-11 | 42.75346417 |
| rs35809007 | A | G | 2 | 0.00205627 | 9.09913E-17 | 69.16361044 |
| rs35957544 | T | G | 8 | 0.00200438 | 1.10002E-22 | 96.03976531 |
| rs36007635 | A | G | 6 | 0.00286788 | 2.19989E-13 | 53.84878511 |
| rs36061954 | T | C | 8 | 0.00201928 | 2E-10 | 40.46143846 |
| rs3764625 | G | T | 19 | 0.00201523 | 5.19996E-09 | 34.11984077 |
| rs3766823 | A | G | 1 | 0.0026141 | 8.10009E-10 | 37.72509349 |
| rs3784710 | C | T | 15 | 0.00236042 | 2.49977E-36 | 158.3909083 |
| rs3803286 | G | A | 14 | 0.00209828 | 6.4003E-19 | 78.93037152 |
| rs3807566 | T | G | 7 | 0.00199583 | 1.5E-09 | 36.58994232 |
| rs3814883 | T | C | 16 | 0.00198424 | 1E-33 | 146.4270263 |
| rs3851998 | G | C | 3 | 0.00226874 | 0.000000002 | 35.97176079 |
| rs3897102 | T | C | 12 | 0.00202716 | 2.5E-09 | 35.53292842 |
| rs3901286 | A | C | 7 | 0.00275602 | 2.70023E-16 | 66.97804795 |
| rs3935190 | A | G | 17 | 0.00199701 | 4.00037E-13 | 52.62116592 |
| rs394608 | C | T | 21 | 0.00199484 | 9.49948E-21 | 87.26832519 |
| rs40071 | C | T | 5 | 0.00258163 | 3.80014E-24 | 102.732201 |
| rs4017425 | T | C | 3 | 0.00198035 | 2.1E-10 | 40.36405096 |
| rs4055791 | T | C | 13 | 0.00200801 | 7.39946E-19 | 78.65173484 |
| rs406388 | G | C | 22 | 0.00260181 | 8.30004E-10 | 37.68962585 |
| rs41279738 | G | T | 1 | 0.0062225 | 4.00037E-28 | 120.9250578 |
| rs41315816 | C | T | 6 | 0.00419129 | 1.29999E-09 | 36.84423294 |
| rs4261944 | G | T | 4 | 0.00205652 | 1.59993E-11 | 45.39060721 |
| rs4284600 | C | T | 15 | 0.00199506 | 0.000000002 | 35.98159689 |
| rs429343 | G | A | 2 | 0.00199729 | 3.29989E-18 | 75.7168086 |
| rs4307239 | G | A | 7 | 0.00198816 | 0.000000001 | 37.29303396 |
| rs4456769 | T | C | 20 | 0.00210138 | 3.59998E-12 | 48.34556468 |
| rs4477562 | T | C | 13 | 0.00298011 | 2.90001E-23 | 98.73361203 |
| rs4482463 | A | C | 2 | 0.00370692 | 2.99985E-17 | 71.37526689 |
| rs45486197 | A | G | 19 | 0.00403737 | 1.7E-10 | 40.72835802 |
| rs4562625 | G | C | 1 | 0.00202513 | 1.2E-10 | 41.53953448 |
| rs4605363 | C | A | 2 | 0.00207733 | 3.29989E-15 | 62.05222229 |
| rs4625888 | T | C | 2 | 0.00198303 | 3.09999E-09 | 35.11307859 |
| rs4648450 | A | C | 1 | 0.00198775 | 8.4004E-14 | 55.71455539 |
| rs4658403 | T | C | 1 | 0.00264997 | 1E-12 | 50.8014651 |
| rs4672338 | T | C | 2 | 0.00208669 | 8.00018E-11 | 42.25694669 |
| rs4722398 | T | C | 7 | 0.00287545 | 7.89951E-11 | 42.29013368 |
| rs4764949 | G | A | 12 | 0.00211197 | 3.10027E-18 | 75.84052264 |
| rs4790292 | A | C | 17 | 0.00275603 | 2.60016E-20 | 85.27827518 |
| rs4820410 | G | A | 22 | 0.00208527 | 1.69981E-17 | 72.42052687 |
| rs4832298 | T | C | 2 | 0.00212268 | 5.40008E-14 | 56.56773501 |
| rs4858940 | C | T | 3 | 0.00309976 | 1.39991E-13 | 54.71167299 |
| rs4876611 | G | A | 8 | 0.00220502 | 3.29989E-19 | 80.26470342 |
| rs4906350 | T | C | 14 | 0.00211827 | 1.69981E-12 | 49.79504336 |
| rs4929923 | C | T | 11 | 0.00206436 | 4.49987E-20 | 84.19748819 |
| rs5011579 | G | C | 16 | 0.00219322 | 1.6E-10 | 40.90277886 |
| rs512121 | C | T | 18 | 0.00252176 | 2.59998E-10 | 39.93426433 |
| rs529200 | G | A | 3 | 0.0019785 | 1.29987E-17 | 72.93254961 |
| rs55707359 | G | T | 11 | 0.00812381 | 6.20012E-11 | 42.77076695 |
| rs55714539 | C | A | 19 | 0.00210043 | 6.00067E-17 | 69.98758709 |
| rs557951 | G | T | 3 | 0.00213387 | 4.49987E-11 | 43.37374281 |
| rs558887 | G | A | 11 | 0.00214881 | 1.5E-09 | 36.587295 |
| rs56038322 | A | G | 3 | 0.00214833 | 8.99912E-11 | 42.02914783 |
| rs56133507 | G | T | 2 | 0.00247385 | 2.69998E-08 | 30.94774459 |
| rs56143236 | T | C | 3 | 0.00226533 | 2.30001E-08 | 31.26540197 |
| rs56161855 | T | A | 17 | 0.00291784 | 1.39991E-14 | 59.23792935 |
| rs56203622 | C | T | 9 | 0.00280183 | 1.40001E-10 | 41.16669891 |
| rs56352336 | C | T | 19 | 0.00275021 | 2.90001E-09 | 35.24366559 |
| rs56399737 | T | C | 13 | 0.00199646 | 6.4003E-16 | 65.29532997 |
| rs56858768 | A | G | 13 | 0.00217378 | 2.39994E-13 | 53.62773757 |
| rs56893062 | G | T | 8 | 0.00215383 | 6.19998E-09 | 33.75797588 |
| rs56930105 | T | C | 2 | 0.00286187 | 3.69999E-08 | 30.30927873 |
| rs58862095 | T | C | 7 | 0.00200786 | 2.39994E-30 | 131.0763036 |
| rs6023655 | G | A | 20 | 0.00234997 | 3.59998E-10 | 39.31443739 |
| rs61740466 | A | G | 1 | 0.00231885 | 5.60003E-09 | 33.97082296 |
| rs61871615 | T | C | 10 | 0.00359269 | 1E-13 | 55.29681636 |
| rs61903695 | G | A | 11 | 0.00227115 | 2.49977E-13 | 53.57390441 |
| rs62020775 | A | T | 15 | 0.0028635 | 7.19996E-09 | 33.47088185 |
| rs62107261 | C | T | 2 | 0.00460882 | 4.60045E-87 | 391.1920974 |
| rs62176243 | T | A | 2 | 0.00228878 | 6.09958E-11 | 42.77890844 |
| rs62190049 | C | G | 2 | 0.00203614 | 0.00000004 | 30.14760367 |
| rs62241847 | G | A | 3 | 0.00212897 | 5.30005E-09 | 34.06783892 |
| rs62379271 | G | T | 5 | 0.00200557 | 0.000000005 | 34.17068965 |
| rs6430068 | A | G | 2 | 0.0031882 | 5.30005E-09 | 34.05986544 |
| rs6444950 | A | G | 3 | 0.00232007 | 8.30042E-12 | 46.68671823 |
| rs6474856 | T | C | 9 | 0.00207048 | 3.79997E-09 | 34.74316954 |
| rs6545714 | A | G | 2 | 0.00201573 | 2.39994E-24 | 103.6502646 |
| rs6560906 | C | T | 12 | 0.00214136 | 0.000000012 | 32.44874363 |
| rs6561937 | A | T | 13 | 0.00230346 | 4.60045E-12 | 47.85069782 |
| rs6575340 | A | G | 14 | 0.00206189 | 8.69961E-24 | 101.1127399 |
| rs66679256 | T | C | 4 | 0.00198759 | 6.89922E-14 | 56.09520799 |
| rs6669189 | T | C | 1 | 0.00201682 | 1.9002E-17 | 72.19800174 |
| rs6669341 | G | A | 1 | 0.0019986 | 1.59993E-17 | 72.59575441 |
| rs6682438 | C | T | 1 | 0.00210047 | 3.69999E-10 | 39.24518714 |
| rs6696828 | C | G | 1 | 0.00214061 | 1.2E-10 | 41.44334975 |
| rs6707827 | G | A | 2 | 0.00217332 | 3.89996E-08 | 30.20163255 |
| rs6710091 | G | C | 2 | 0.00206707 | 0.000000016 | 31.94740283 |
| rs6713781 | C | G | 2 | 0.00202799 | 2.19989E-11 | 44.79798452 |
| rs6725931 | T | C | 2 | 0.00274403 | 3.29989E-12 | 48.47280902 |
| rs6744646 | G | A | 2 | 0.00261213 | 4.49987E-100 | 450.9222895 |
| rs6752979 | A | G | 2 | 0.00211721 | 3.29997E-09 | 34.98835844 |
| rs67609008 | C | T | 10 | 0.0022021 | 8.60003E-15 | 60.20353338 |
| rs6774894 | A | T | 3 | 0.00205674 | 9.70063E-11 | 41.87154717 |
| rs6831088 | A | G | 4 | 0.0020591 | 2.19999E-08 | 31.3123701 |
| rs6843852 | T | C | 4 | 0.00197607 | 3.50026E-11 | 43.87879945 |
| rs6909685 | T | C | 6 | 0.00211352 | 4.70002E-12 | 47.79765645 |
| rs6922607 | G | A | 6 | 0.00251479 | 3.2E-09 | 35.03342178 |
| rs6950388 | A | G | 7 | 0.00244806 | 2.30001E-10 | 40.2018137 |
| rs698147 | G | A | 5 | 0.00198514 | 9.60064E-11 | 41.90878635 |
| rs7024334 | G | T | 9 | 0.00238338 | 6.69993E-09 | 33.61127115 |
| rs7027304 | T | C | 9 | 0.00208703 | 3.10027E-12 | 48.59827387 |
| rs7034554 | G | A | 9 | 0.00204252 | 2.1E-10 | 40.40087001 |
| rs7038943 | C | T | 9 | 0.00208641 | 1.80011E-11 | 45.15533896 |
| rs704061 | C | T | 12 | 0.00198552 | 1.69981E-13 | 54.34747233 |
| rs705145 | A | C | 10 | 0.0020798 | 2.29985E-11 | 44.68046338 |
| rs7070670 | T | C | 10 | 0.00211928 | 5.99998E-09 | 33.83500906 |
| rs7081254 | C | T | 10 | 0.00245216 | 6.19998E-09 | 33.77766633 |
| rs7124681 | A | C | 11 | 0.0020063 | 1.50003E-37 | 164.0577546 |
| rs7132908 | A | G | 12 | 0.00203363 | 1.39991E-48 | 214.5896588 |
| rs71495038 | A | G | 10 | 0.00370969 | 6.70039E-14 | 56.15754377 |
| rs7218014 | C | T | 17 | 0.00249216 | 2.90001E-14 | 57.83311506 |
| rs723672 | T | C | 12 | 0.00200698 | 2.99999E-08 | 30.73926683 |
| rs7259070 | C | T | 19 | 0.00203628 | 6.59933E-27 | 115.340773 |
| rs72634826 | A | G | 1 | 0.00228001 | 1E-20 | 87.08578753 |
| rs72649373 | C | T | 4 | 0.00287805 | 6.1E-10 | 38.27769917 |
| rs72976986 | A | G | 19 | 0.00254712 | 7.59976E-20 | 83.14551205 |
| rs73052033 | C | T | 3 | 0.00254634 | 7.70016E-33 | 142.4660875 |
| rs7306534 | A | G | 12 | 0.00205437 | 4.09996E-08 | 30.12358018 |
| rs73124396 | C | T | 7 | 0.0024548 | 3.09999E-10 | 39.59745828 |
| rs73142879 | T | C | 20 | 0.00252257 | 3.59998E-26 | 111.9767462 |
| rs73193736 | G | A | 12 | 0.00232002 | 2.19989E-14 | 58.31394186 |
| rs73213484 | T | A | 4 | 0.0028364 | 1.69981E-15 | 63.35517918 |
| rs7331420 | A | G | 13 | 0.00220081 | 6.59933E-11 | 42.63443614 |
| rs7357754 | G | A | 9 | 0.00198362 | 1.10002E-12 | 50.63142995 |
| rs73601548 | T | C | 10 | 0.00311639 | 1.29999E-08 | 32.38757052 |
| rs73985439 | C | A | 2 | 0.00214059 | 1.7E-10 | 40.73203857 |
| rs7442885 | G | C | 5 | 0.00241251 | 3.19963E-21 | 89.44420178 |
| rs745249 | T | C | 2 | 0.00219724 | 1.10002E-15 | 64.24694726 |
| rs74750282 | C | T | 7 | 0.00352561 | 0.000000025 | 31.02664515 |
| rs7498665 | G | A | 16 | 0.00201997 | 2.29985E-40 | 176.8768186 |
| rs7516554 | T | C | 1 | 0.00201565 | 2.59998E-09 | 35.48214276 |
| rs7519259 | A | G | 1 | 0.00198381 | 1.69981E-12 | 49.82297101 |
| rs754635 | G | C | 3 | 0.0031093 | 1.50003E-12 | 50.02924485 |
| rs75499503 | T | C | 6 | 0.00241845 | 8.9002E-14 | 55.59894565 |
| rs7571496 | G | A | 2 | 0.00225454 | 1.80011E-12 | 49.68432115 |
| rs76183894 | C | T | 3 | 0.00364437 | 1.7E-09 | 36.30416549 |
| rs7619139 | A | T | 3 | 0.00201061 | 2.19989E-11 | 44.77419662 |
| rs76267866 | T | A | 3 | 0.00243965 | 6.69993E-09 | 33.6106694 |
| rs76702514 | G | C | 1 | 0.00243278 | 1.20005E-11 | 45.92355021 |
| rs7683836 | A | G | 4 | 0.00199427 | 8.19993E-10 | 37.71242253 |
| rs7708584 | G | A | 5 | 0.0019949 | 1.39991E-15 | 63.78283029 |
| rs7761673 | A | T | 6 | 0.0023901 | 1.29999E-08 | 32.33721656 |
| rs7762794 | G | A | 6 | 0.00218606 | 9.09913E-12 | 46.50473068 |
| rs7776021 | A | G | 6 | 0.00218251 | 1.40001E-08 | 32.12431949 |
| rs7802342 | G | T | 7 | 0.00218145 | 1.79999E-08 | 31.66199843 |
| rs7805441 | T | C | 7 | 0.00198884 | 1.80011E-11 | 45.22394109 |
| rs78605811 | C | A | 3 | 0.00444673 | 1.80011E-13 | 54.19961929 |
| rs78886584 | G | A | 1 | 0.0019948 | 0.000000005 | 34.20209197 |
| rs7893571 | T | G | 10 | 0.00210152 | 2.29985E-11 | 44.73147144 |
| rs7924036 | T | G | 10 | 0.0019778 | 5.10035E-13 | 52.1567611 |
| rs7925100 | A | G | 11 | 0.0020221 | 3.29989E-13 | 53.02801232 |
| rs7942037 | C | G | 11 | 0.00205793 | 3.59998E-15 | 61.93147659 |
| rs7944782 | G | T | 11 | 0.00198748 | 2.19989E-15 | 62.88397114 |
| rs7947143 | A | G | 11 | 0.00267522 | 8.30042E-12 | 46.68182702 |
| rs7975187 | G | A | 12 | 0.00241251 | 3.50002E-10 | 39.36313977 |
| rs79780963 | T | C | 10 | 0.00369591 | 1.5E-10 | 41.05174532 |
| rs7996639 | A | G | 13 | 0.00200245 | 3.59998E-13 | 52.83902131 |
| rs80135274 | T | A | 17 | 0.00388113 | 3.79997E-08 | 30.22955451 |
| rs8015400 | A | C | 14 | 0.00211709 | 6.70039E-24 | 101.6248059 |
| rs8020365 | A | T | 14 | 0.00239517 | 1E-25 | 109.8778212 |
| rs8024137 | T | A | 15 | 0.00276778 | 0.000000016 | 31.90185715 |
| rs8025516 | G | T | 15 | 0.00207578 | 1.69981E-12 | 49.8489957 |
| rs8076669 | C | T | 17 | 0.00199601 | 1.80011E-12 | 49.67586841 |
| rs8089514 | A | T | 18 | 0.00207588 | 3.89996E-10 | 39.14534009 |
| rs8132491 | A | G | 21 | 0.00219317 | 2.39994E-12 | 49.14571476 |
| rs815163 | C | T | 1 | 0.00198582 | 1E-16 | 68.91252171 |
| rs852042 | G | A | 20 | 0.00231309 | 1.40001E-08 | 32.17577002 |
| rs862320 | T | C | 16 | 0.00201345 | 1.20005E-30 | 132.4285474 |
| rs879620 | T | C | 16 | 0.00203595 | 2.29985E-32 | 140.2781044 |
| rs909892 | A | G | 20 | 0.00291255 | 2.59998E-10 | 39.95621924 |
| rs9294260 | A | G | 6 | 0.00198818 | 1E-13 | 55.27834021 |
| rs9349235 | T | C | 6 | 0.00200924 | 2.59998E-08 | 30.96634125 |
| rs935166 | A | G | 2 | 0.00197292 | 3.19963E-16 | 66.64825696 |
| rs9366863 | C | T | 6 | 0.00210043 | 5.79963E-42 | 184.2155196 |
| rs9461887 | T | C | 6 | 0.00220206 | 1.39991E-11 | 45.71770606 |
| rs9463175 | T | C | 6 | 0.00210115 | 4.39997E-08 | 29.95584437 |
| rs9515446 | G | A | 13 | 0.00199052 | 3.19963E-14 | 57.58562946 |
| rs9522180 | T | C | 13 | 0.00199325 | 1.50003E-12 | 50.10715007 |
| rs9571687 | A | C | 13 | 0.0021094 | 1.40001E-10 | 41.19480601 |
| rs9638713 | G | A | 7 | 0.00635399 | 0.000000012 | 32.41066998 |
| rs9673839 | G | A | 16 | 0.00198783 | 5.50047E-11 | 42.99094185 |
| rs9674487 | G | C | 17 | 0.0286171 | 3.09999E-08 | 30.6553187 |
| rs9830592 | A | C | 3 | 0.00200164 | 1E-14 | 59.84734212 |
| rs9839081 | A | G | 3 | 0.00214045 | 4.60002E-08 | 29.88379744 |
| rs9843653 | C | T | 3 | 0.00197538 | 2.90001E-50 | 222.2776709 |
| rs9876664 | T | G | 3 | 0.00204174 | 9.60064E-19 | 78.13536853 |
| rs9888533 | T | C | 13 | 0.0020182 | 2.69998E-09 | 35.37603037 |
| rs9926784 | C | T | 16 | 0.00254677 | 8.60003E-21 | 87.4643617 |
| rs9931586 | G | C | 16 | 0.00224111 | 4.70002E-08 | 29.83226042 |
| rs9951619 | G | T | 18 | 0.00235777 | 9.49992E-10 | 37.42091085 |
| rs9991259 | A | G | 4 | 0.00204963 | 3.89996E-08 | 30.2110226 |

**Table S2**. Genetic instruments used in the analyses for the association of Waist circumference with Heart failure.

| SNP | EA | OA | chr | SE | *P* value | F |
| --- | --- | --- | --- | --- | --- | --- |
| rs10111287 | T | C | 8 | 0.00198784 | 4.60002E-08 | 29.87147293 |
| rs1013402 | G | A | 11 | 0.00191723 | 3.90032E-39 | 171.2669886 |
| rs10150482 | A | G | 14 | 0.00216957 | 4.49987E-24 | 102.4270095 |
| rs10184230 | T | C | 2 | 0.00186862 | 8.9002E-11 | 42.04411535 |
| rs10236214 | T | C | 7 | 0.00187561 | 1.9002E-13 | 54.12652556 |
| rs10247983 | A | G | 7 | 0.00338732 | 2.19999E-09 | 35.78415362 |
| rs10248298 | A | C | 7 | 0.00185427 | 8.80035E-13 | 51.10144474 |
| rs1025065 | G | T | 16 | 0.00186623 | 3.59998E-08 | 30.36643457 |
| rs10269774 | A | G | 7 | 0.00190774 | 3.59998E-10 | 39.30087324 |
| rs1037702 | A | G | 4 | 0.00184913 | 3.59998E-08 | 30.35052791 |
| rs10423928 | A | T | 19 | 0.00225944 | 6.79986E-32 | 138.1319861 |
| rs10471636 | A | G | 5 | 0.00182385 | 2.99999E-08 | 30.73335594 |
| rs10490869 | T | A | 3 | 0.00220375 | 1.20005E-13 | 54.96939776 |
| rs10499014 | G | C | 6 | 0.00202893 | 5.90065E-11 | 42.85866117 |
| rs10505836 | C | A | 12 | 0.00259774 | 4.09996E-09 | 34.55533848 |
| rs1051613 | A | G | 4 | 0.00179742 | 3.09999E-08 | 30.65636952 |
| rs10772985 | A | G | 12 | 0.00195421 | 0.000000015 | 31.99200451 |
| rs10787738 | T | C | 10 | 0.00208556 | 2.09991E-13 | 53.86567283 |
| rs10824211 | T | C | 10 | 0.00259306 | 6.29999E-09 | 33.75126676 |
| rs10827380 | T | C | 10 | 0.0019302 | 1.79999E-09 | 36.18176445 |
| rs10835676 | G | C | 11 | 0.00209843 | 0.000000004 | 34.64410414 |
| rs10842240 | C | G | 12 | 0.00278463 | 2.99999E-08 | 30.72961027 |
| rs10947793 | G | A | 6 | 0.0018605 | 8.10028E-12 | 46.75163594 |
| rs10957087 | A | T | 8 | 0.00244363 | 0.000000012 | 32.56005757 |
| rs10962550 | C | G | 9 | 0.00231818 | 8.40001E-10 | 37.674044 |
| rs10992854 | C | T | 9 | 0.00192897 | 5.39995E-09 | 34.04093374 |
| rs11012732 | G | A | 10 | 0.00190117 | 2.39994E-24 | 103.6290738 |
| rs1108548 | G | A | 1 | 0.00200003 | 1.2E-09 | 37.035242 |
| rs11099020 | T | C | 4 | 0.00186604 | 1.7E-09 | 36.31359933 |
| rs1111817 | G | C | 9 | 0.00188105 | 0.00000002 | 31.51392383 |
| rs111258054 | T | C | 11 | 0.00234105 | 9.49948E-12 | 46.43574976 |
| rs11160600 | G | A | 14 | 0.00311766 | 7.10003E-09 | 33.51128161 |
| rs11162968 | C | T | 1 | 0.00192412 | 2.1E-10 | 40.38944282 |
| rs11165493 | A | G | 1 | 0.00189335 | 1.29999E-08 | 32.28263499 |
| rs1117619 | G | C | 3 | 0.00206049 | 0.000000005 | 34.20792829 |
| rs11215381 | C | T | 11 | 0.00179363 | 4.60002E-10 | 38.82391216 |
| rs11218510 | A | G | 11 | 0.00182872 | 2.90001E-10 | 39.76833732 |
| rs11223204 | G | A | 11 | 0.00180899 | 1.50003E-11 | 45.59771222 |
| rs112266013 | A | G | 6 | 0.00255067 | 5.19996E-09 | 34.1293573 |
| rs113132247 | A | G | 9 | 0.00249349 | 8.40001E-10 | 37.66647011 |
| rs113866544 | C | T | 17 | 0.003552 | 1.39991E-17 | 72.88409918 |
| rs114964326 | A | G | 2 | 0.00537007 | 0.000000021 | 31.44495945 |
| rs11603984 | T | G | 11 | 0.00261937 | 8.9002E-11 | 42.04178528 |
| rs11639596 | C | A | 16 | 0.00207809 | 1.09999E-08 | 32.63766428 |
| rs11653367 | G | A | 17 | 0.00191685 | 2.49977E-15 | 62.60372968 |
| rs11675464 | G | A | 2 | 0.00179869 | 3.69999E-10 | 39.27884136 |
| rs11704728 | T | C | 22 | 0.00226433 | 3.89996E-09 | 34.65983324 |
| rs11757278 | C | T | 6 | 0.00194427 | 4.90004E-11 | 43.23349783 |
| rs11767811 | A | G | 7 | 0.00231875 | 8.9002E-11 | 42.04071803 |
| rs11773362 | T | C | 7 | 0.0018937 | 0.000000032 | 30.61033695 |
| rs1183668 | G | C | 13 | 0.00186172 | 9.30037E-11 | 41.95528339 |
| rs11842871 | T | G | 13 | 0.00204439 | 4E-10 | 39.12588506 |
| rs1188209 | G | A | 14 | 0.00181136 | 0.000000016 | 31.9575445 |
| rs11898037 | C | T | 2 | 0.00185324 | 0.000000012 | 32.52650028 |
| rs1191600 | A | C | 14 | 0.00183345 | 3.79997E-09 | 34.70160651 |
| rs12001437 | C | T | 9 | 0.00185543 | 3.79997E-09 | 34.72142112 |
| rs12042959 | G | A | 1 | 0.00255686 | 5.19996E-09 | 34.11445173 |
| rs12072739 | G | A | 1 | 0.00214325 | 3.90032E-14 | 57.20744009 |
| rs12107172 | G | A | 3 | 0.00260901 | 1.79999E-09 | 36.14475179 |
| rs12140153 | T | G | 1 | 0.00313243 | 2.09991E-17 | 72.07613914 |
| rs1218824 | A | G | 13 | 0.00189332 | 4.19952E-11 | 43.52576086 |
| rs12245654 | C | A | 10 | 0.00347158 | 3.19963E-11 | 44.0731862 |
| rs12273545 | T | C | 11 | 0.00387781 | 9.40005E-09 | 32.962909 |
| rs12287076 | C | G | 11 | 0.00197238 | 1.29987E-26 | 114.0492057 |
| rs1229984 | C | T | 4 | 0.00542443 | 2.80001E-08 | 30.86824884 |
| rs12375196 | A | C | 7 | 0.00182044 | 3.80014E-13 | 52.72599129 |
| rs12462975 | A | G | 19 | 0.00191777 | 5.90065E-20 | 83.6500369 |
| rs12463617 | C | A | 2 | 0.00236577 | 1.80011E-74 | 333.3186218 |
| rs12478299 | C | T | 2 | 0.00206169 | 1.89998E-08 | 31.5457158 |
| rs12549000 | A | T | 8 | 0.00292178 | 1.89998E-08 | 31.54861947 |
| rs12877270 | A | G | 13 | 0.00181553 | 1.09999E-10 | 41.68945464 |
| rs12880641 | G | T | 14 | 0.00188965 | 2.39994E-13 | 53.68054332 |
| rs1296328 | C | A | 4 | 0.00180908 | 3.10027E-13 | 53.1683356 |
| rs12983532 | T | C | 19 | 0.00209191 | 7.59976E-13 | 51.39111492 |
| rs13033310 | A | G | 2 | 0.00206781 | 1.29999E-09 | 36.84251233 |
| rs13163306 | A | G | 5 | 0.00179351 | 3.50002E-08 | 30.40527734 |
| rs1321519 | G | A | 6 | 0.00187835 | 5.19996E-14 | 56.66021359 |
| rs1327259 | G | A | 6 | 0.0018403 | 3.09999E-10 | 39.63873471 |
| rs13273726 | A | C | 8 | 0.00216798 | 7.10003E-09 | 33.50057963 |
| rs13288841 | A | G | 9 | 0.00191229 | 2.19989E-23 | 99.2400585 |
| rs13322435 | G | A | 3 | 0.00182964 | 1.9002E-20 | 85.88565769 |
| rs13333747 | C | T | 16 | 0.00232636 | 1.9002E-22 | 95.02889037 |
| rs13410783 | G | A | 2 | 0.0018498 | 2.39994E-14 | 58.14948778 |
| rs13420048 | A | C | 2 | 0.00185957 | 7.00003E-13 | 51.54226432 |
| rs13427822 | G | A | 2 | 0.00203048 | 3.19963E-12 | 48.56067947 |
| rs1346841 | A | G | 4 | 0.00182614 | 6.4E-09 | 33.71746511 |
| rs1357079 | C | T | 3 | 0.00180784 | 6.19998E-10 | 38.26455501 |
| rs1360201 | T | C | 9 | 0.00178924 | 4.39997E-08 | 29.96412678 |
| rs1436348 | G | A | 3 | 0.00181185 | 5.40008E-12 | 47.54250345 |
| rs1441264 | A | G | 13 | 0.00186203 | 7.59976E-16 | 64.97691722 |
| rs145350287 | A | T | 12 | 0.00458244 | 1.99986E-12 | 49.47628732 |
| rs1458156 | T | C | 12 | 0.00179105 | 3.50026E-16 | 66.48205537 |
| rs1502317 | T | C | 11 | 0.0020032 | 4.30031E-18 | 75.17717551 |
| rs1507331 | G | C | 1 | 0.00263908 | 1.5E-09 | 36.53085428 |
| rs1554654 | T | C | 3 | 0.00179351 | 1.89998E-08 | 31.58924962 |
| rs1559900 | T | C | 8 | 0.00197882 | 1.09999E-10 | 41.66778767 |
| rs1570298 | T | A | 6 | 0.00204452 | 3.79997E-09 | 34.70867967 |
| rs1582931 | A | G | 5 | 0.00180588 | 1.69981E-14 | 58.86033715 |
| rs1609010 | G | A | 8 | 0.00180594 | 1.29987E-16 | 68.37986051 |
| rs1609303 | A | T | 2 | 0.00185779 | 2.09991E-16 | 67.52563139 |
| rs1625623 | T | C | 5 | 0.00187307 | 9.80009E-09 | 32.88191912 |
| rs1657930 | A | G | 15 | 0.00224816 | 2.30001E-10 | 40.16608253 |
| rs17296856 | C | A | 15 | 0.00199629 | 5.00035E-15 | 61.24497808 |
| rs1731246 | T | G | 2 | 0.0020818 | 1.29999E-08 | 32.32778392 |
| rs17446091 | C | T | 8 | 0.00222979 | 2.29985E-11 | 44.73692965 |
| rs1752169 | A | C | 9 | 0.00206705 | 4.49987E-12 | 47.91461913 |
| rs17681738 | T | C | 17 | 0.00190864 | 3.29997E-08 | 30.50747194 |
| rs1799923 | G | A | 3 | 0.00281552 | 1.2E-09 | 36.96829344 |
| rs1801282 | G | C | 3 | 0.00274708 | 2.60016E-12 | 48.96810223 |
| rs1834144 | A | C | 18 | 0.00185609 | 3.40017E-15 | 62.00282304 |
| rs1861410 | T | C | 2 | 0.00180199 | 6.4003E-19 | 78.92664751 |
| rs1902066 | C | T | 6 | 0.00181038 | 1.40001E-09 | 36.60116996 |
| rs1942826 | A | G | 18 | 0.00270011 | 4.90004E-10 | 38.69999171 |
| rs2074881 | T | C | 19 | 0.00240193 | 8E-10 | 37.76788248 |
| rs2133561 | T | A | 5 | 0.00185284 | 3.90032E-11 | 43.66946788 |
| rs215669 | A | G | 7 | 0.00184246 | 1.10002E-11 | 46.09817867 |
| rs2161097 | T | C | 5 | 0.00180004 | 2.19989E-15 | 62.91118394 |
| rs2180454 | C | T | 14 | 0.00213539 | 3.69999E-17 | 70.91466707 |
| rs2183947 | A | G | 6 | 0.00213628 | 4.90004E-25 | 106.8107369 |
| rs2225909 | C | T | 11 | 0.00213642 | 1.39991E-13 | 54.6733854 |
| rs2237403 | T | C | 7 | 0.00188895 | 6.59994E-09 | 33.64976486 |
| rs2297600 | G | T | 1 | 0.00237197 | 3.09999E-08 | 30.67285351 |
| rs2302209 | T | C | 19 | 0.00197713 | 1.10002E-23 | 100.6240962 |
| rs2307111 | C | T | 5 | 0.0018299 | 3.29989E-39 | 171.6173021 |
| rs2376885 | A | G | 16 | 0.00191252 | 2.80001E-08 | 30.84786806 |
| rs2439823 | G | A | 10 | 0.00180145 | 6.79986E-18 | 74.27474532 |
| rs245767 | G | A | 5 | 0.00201635 | 4.49987E-13 | 52.41204183 |
| rs2470549 | C | T | 3 | 0.00182105 | 5.19996E-11 | 43.09134289 |
| rs2470946 | T | G | 7 | 0.00182473 | 1.5E-10 | 41.0212475 |
| rs2482704 | T | G | 9 | 0.00180618 | 1.5E-10 | 40.99294597 |
| rs2568958 | A | G | 1 | 0.00182184 | 1.59993E-20 | 86.26031736 |
| rs2584205 | A | G | 18 | 0.00202662 | 3.69999E-08 | 30.31946084 |
| rs2678204 | G | T | 1 | 0.00188488 | 7.89951E-17 | 69.44503393 |
| rs2725371 | G | A | 8 | 0.00195255 | 2.60016E-15 | 62.5723902 |
| rs28350 | G | A | 3 | 0.0023374 | 2.5E-09 | 35.55575569 |
| rs28366156 | C | T | 6 | 0.00265145 | 1.50003E-12 | 50.09240831 |
| rs28375268 | T | G | 16 | 0.00187766 | 3.29989E-12 | 48.51863 |
| rs28489620 | A | G | 22 | 0.00198899 | 4.39997E-10 | 38.94891005 |
| rs2861692 | C | T | 2 | 0.00199772 | 7.70016E-17 | 69.47809799 |
| rs2903738 | T | A | 19 | 0.00215663 | 6.80002E-10 | 38.06714283 |
| rs3087523 | A | G | 2 | 0.00271786 | 0.000000001 | 37.24167091 |
| rs308911 | G | A | 2 | 0.00198055 | 6.90001E-09 | 33.56805648 |
| rs3113509 | T | C | 4 | 0.00201975 | 0.000000001 | 37.26272073 |
| rs34140906 | C | T | 13 | 0.00238486 | 5.40008E-14 | 56.5816389 |
| rs34234296 | A | G | 2 | 0.00184833 | 1.29987E-12 | 50.3966101 |
| rs34483452 | A | C | 5 | 0.0026269 | 7.29962E-25 | 106.0212373 |
| rs34517439 | A | C | 1 | 0.00276165 | 1.9002E-28 | 122.3656626 |
| rs34994596 | C | T | 15 | 0.00195866 | 1.10002E-13 | 55.25161586 |
| rs35023999 | C | A | 11 | 0.00178977 | 2.39999E-10 | 40.15139082 |
| rs35681682 | C | T | 2 | 0.00177268 | 0.00000001 | 32.80668147 |
| rs35882248 | T | C | 2 | 0.00191956 | 2.80027E-16 | 66.97036068 |
| rs36007635 | A | G | 6 | 0.00259543 | 1.7E-10 | 40.75188891 |
| rs36061954 | T | C | 8 | 0.00182669 | 3.59998E-10 | 39.34073307 |
| rs36140 | C | A | 5 | 0.001867 | 2.1E-09 | 35.83436343 |
| rs36165342 | C | T | 12 | 0.00178943 | 2.1E-09 | 35.83401595 |
| rs3764002 | T | C | 12 | 0.00203505 | 2.80027E-15 | 62.41236802 |
| rs3768321 | T | G | 1 | 0.00224849 | 3.80014E-15 | 61.77588485 |
| rs3784692 | T | C | 15 | 0.00182662 | 2.60016E-24 | 103.4920981 |
| rs3806114 | A | G | 6 | 0.00192005 | 1.79999E-08 | 31.72340397 |
| rs3807566 | T | G | 7 | 0.00180583 | 1.69981E-11 | 45.26133264 |
| rs3814883 | T | C | 16 | 0.00179535 | 1.10002E-40 | 178.4259664 |
| rs3826408 | T | C | 17 | 0.00179579 | 4.09996E-10 | 39.07094033 |
| rs3845344 | T | C | 1 | 0.00182845 | 4.30002E-09 | 34.48322042 |
| rs3935190 | A | G | 17 | 0.00180625 | 2.80027E-12 | 48.81222177 |
| rs3936510 | T | G | 5 | 0.0022268 | 7.29995E-10 | 37.93238929 |
| rs40067 | A | G | 5 | 0.00238663 | 6.09958E-11 | 42.79319492 |
| rs4072917 | A | G | 8 | 0.0017994 | 7.39946E-11 | 42.41488213 |
| rs4075353 | A | G | 10 | 0.00189213 | 2.19999E-08 | 31.31604719 |
| rs41279738 | G | T | 1 | 0.00563338 | 1.69981E-20 | 86.05691178 |
| rs4290163 | T | G | 10 | 0.00183305 | 5.39995E-10 | 38.52656897 |
| rs429343 | G | A | 2 | 0.00180931 | 5.90065E-12 | 47.35605574 |
| rs4456769 | T | C | 20 | 0.0019003 | 1.80011E-12 | 49.72465081 |
| rs4469245 | T | A | 5 | 0.00189126 | 1.09999E-09 | 37.1095269 |
| rs4482463 | A | C | 2 | 0.00335821 | 1.29987E-14 | 59.43951249 |
| rs4525978 | T | C | 4 | 0.00203018 | 0.000000021 | 31.39803298 |
| rs4527444 | G | A | 4 | 0.00179433 | 4.49997E-09 | 34.40185033 |
| rs4552632 | A | G | 5 | 0.00184128 | 3.29997E-08 | 30.50419226 |
| rs4562625 | G | C | 1 | 0.00183376 | 6.1E-09 | 33.78686792 |
| rs4689465 | C | T | 4 | 0.00178976 | 1.29999E-09 | 36.87920842 |
| rs4718964 | T | G | 7 | 0.00182202 | 1.80011E-11 | 45.19031311 |
| rs4722398 | T | C | 7 | 0.00260196 | 1E-10 | 41.74023245 |
| rs4740627 | C | T | 9 | 0.00180496 | 3.40017E-16 | 66.53792596 |
| rs484455 | A | G | 13 | 0.00179638 | 1.40001E-10 | 41.22240715 |
| rs4876611 | G | A | 8 | 0.0019947 | 5.30029E-14 | 56.61342163 |
| rs4900590 | T | C | 14 | 0.00191504 | 6.29941E-11 | 42.72133399 |
| rs4900715 | A | G | 14 | 0.00179331 | 1.89998E-10 | 40.60898385 |
| rs4908672 | T | C | 1 | 0.00183009 | 4.70002E-10 | 38.77981342 |
| rs520478 | T | G | 6 | 0.00197262 | 2.30001E-10 | 40.18717462 |
| rs55794894 | A | G | 7 | 0.00261481 | 2.80001E-08 | 30.85687973 |
| rs557951 | G | T | 3 | 0.00193163 | 4E-10 | 39.10271279 |
| rs577721086 | C | T | 6 | 0.0041713 | 1.99986E-13 | 53.96840128 |
| rs587271 | T | C | 1 | 0.0020079 | 4.09996E-09 | 34.56823885 |
| rs58862095 | T | C | 7 | 0.00181678 | 7.10068E-20 | 83.29659235 |
| rs588660 | A | G | 1 | 0.00181174 | 1E-17 | 73.45424918 |
| rs59104534 | T | C | 8 | 0.00196197 | 4.70002E-08 | 29.84803283 |
| rs6001877 | A | G | 22 | 0.00189657 | 2.39999E-08 | 31.11368719 |
| rs6030803 | C | T | 20 | 0.00270327 | 6.09958E-11 | 42.778443 |
| rs6069037 | A | C | 20 | 0.00202001 | 3.59998E-08 | 30.331438 |
| rs61903695 | G | A | 11 | 0.00205497 | 5.90065E-11 | 42.8473535 |
| rs61969511 | A | G | 13 | 0.0020127 | 3.79997E-09 | 34.70495987 |
| rs62072003 | T | C | 17 | 0.00256295 | 1.79999E-08 | 31.71048357 |
| rs62174721 | G | A | 2 | 0.0020945 | 4.90004E-08 | 29.76068435 |
| rs62243489 | G | T | 3 | 0.00204876 | 2.99985E-14 | 57.7171551 |
| rs62261725 | G | A | 3 | 0.00190872 | 6.70039E-15 | 60.69121141 |
| rs6493498 | C | T | 15 | 0.001805 | 4.19952E-13 | 52.52836427 |
| rs649458 | A | T | 1 | 0.00256951 | 1.50003E-12 | 50.05406519 |
| rs6536575 | C | T | 4 | 0.00178995 | 1.40001E-09 | 36.69721761 |
| rs6551304 | G | A | 3 | 0.00239416 | 2.09991E-12 | 49.39773639 |
| rs6575340 | A | G | 14 | 0.00186502 | 4.60045E-18 | 75.06365769 |
| rs6669341 | G | A | 1 | 0.0018097 | 4.49987E-12 | 47.87321631 |
| rs6693294 | G | A | 1 | 0.00192843 | 1.10002E-18 | 77.82759745 |
| rs6739755 | G | A | 2 | 0.00182885 | 3.10027E-18 | 75.81473462 |
| rs67609008 | C | T | 10 | 0.00199233 | 0.000000025 | 31.05130785 |
| rs67632512 | A | C | 5 | 0.00281561 | 3.79997E-09 | 34.72874779 |
| rs6791983 | A | C | 3 | 0.00205988 | 2.30001E-09 | 35.74134439 |
| rs6846041 | G | C | 4 | 0.00191467 | 1.29999E-10 | 41.31784944 |
| rs6849518 | T | C | 4 | 0.00270973 | 7.10068E-16 | 65.1015468 |
| rs7034554 | G | A | 9 | 0.00184813 | 1.09999E-09 | 37.14933929 |
| rs703983 | G | A | 10 | 0.00181827 | 3.09999E-10 | 39.58127191 |
| rs704061 | C | T | 12 | 0.00179659 | 4.00037E-16 | 66.24199808 |
| rs7070670 | T | C | 10 | 0.00191737 | 3.50002E-10 | 39.37760992 |
| rs7094644 | A | G | 10 | 0.00194957 | 2.70023E-11 | 44.38063562 |
| rs7115013 | T | C | 11 | 0.00180461 | 3.69999E-09 | 34.75902251 |
| rs7132908 | A | G | 12 | 0.00183998 | 1.39991E-31 | 136.7010065 |
| rs71495038 | A | G | 10 | 0.00335663 | 2.60016E-11 | 44.44550371 |
| rs7154982 | A | G | 14 | 0.00201799 | 1.80011E-16 | 67.83279273 |
| rs7169847 | T | G | 15 | 0.00186771 | 3.79997E-08 | 30.23760374 |
| rs7171864 | A | G | 15 | 0.00189898 | 1.10002E-11 | 46.07067988 |
| rs7218014 | C | T | 17 | 0.00225393 | 2.70023E-23 | 98.84625708 |
| rs7259070 | C | T | 19 | 0.00184133 | 6.59933E-17 | 69.7768779 |
| rs72618637 | A | T | 2 | 0.00231667 | 0.000000021 | 31.39746909 |
| rs72634826 | A | G | 1 | 0.00206456 | 5.40008E-13 | 52.05552327 |
| rs72976986 | A | G | 19 | 0.00230334 | 7.8001E-12 | 46.80849486 |
| rs73052033 | C | T | 3 | 0.00230513 | 7.89951E-20 | 83.07009789 |
| rs73068448 | T | C | 7 | 0.00241309 | 2E-10 | 40.43034958 |
| rs73142879 | T | C | 20 | 0.00228107 | 2.80027E-26 | 112.498131 |
| rs7324067 | C | T | 13 | 0.00210064 | 7.69999E-09 | 33.34240001 |
| rs735033 | G | A | 12 | 0.00184312 | 0.000000025 | 31.02261296 |
| rs7372674 | A | C | 3 | 0.00186339 | 1.7E-10 | 40.74735895 |
| rs7377083 | A | C | 4 | 0.00181913 | 3.19963E-15 | 62.13647353 |
| rs73985439 | C | A | 2 | 0.00193905 | 1.6E-10 | 40.9248893 |
| rs74395133 | C | T | 3 | 0.00263949 | 6.59994E-10 | 38.13366286 |
| rs7442885 | G | C | 5 | 0.00218339 | 6.79986E-21 | 87.9174095 |
| rs746839 | G | C | 8 | 0.00187751 | 2.39999E-10 | 40.13272539 |
| rs7498044 | A | G | 15 | 0.00219298 | 4.90004E-12 | 47.72483738 |
| rs7498665 | G | A | 16 | 0.00182769 | 4.90004E-48 | 212.0706192 |
| rs75035127 | G | A | 1 | 0.0052036 | 1.5E-10 | 41.05983168 |
| rs7519259 | A | G | 1 | 0.00179634 | 1.69981E-12 | 49.75041578 |
| rs7537581 | A | C | 1 | 0.00180248 | 2.59998E-09 | 35.47809377 |
| rs76286777 | C | T | 2 | 0.0021623 | 1.80011E-27 | 117.87253 |
| rs7630382 | T | C | 3 | 0.00179336 | 9.30037E-14 | 55.51469744 |
| rs76653871 | C | T | 12 | 0.00370775 | 1.89998E-08 | 31.55180476 |
| rs7708584 | G | A | 5 | 0.00180534 | 1.39991E-11 | 45.64894713 |
| rs77165542 | T | C | 2 | 0.00488088 | 9.8992E-47 | 206.0734813 |
| rs7752202 | T | C | 6 | 0.00253183 | 2.70023E-12 | 48.87836914 |
| rs7792176 | C | T | 7 | 0.00245777 | 2.19999E-10 | 40.25101906 |
| rs7845090 | A | G | 8 | 0.00197942 | 6.29941E-23 | 97.18193339 |
| rs7925100 | A | G | 11 | 0.00182953 | 1.69981E-14 | 58.89771547 |
| rs7952436 | T | C | 11 | 0.00326001 | 5.90065E-19 | 79.10307809 |
| rs8024137 | T | A | 15 | 0.00250296 | 4.60002E-08 | 29.89471924 |
| rs80243702 | A | G | 15 | 0.00245648 | 6.69993E-10 | 38.1182382 |
| rs815163 | C | T | 1 | 0.00179812 | 2.70023E-13 | 53.38767468 |
| rs8192675 | C | T | 3 | 0.00196858 | 4.49987E-16 | 65.99571208 |
| rs852042 | G | A | 20 | 0.00209164 | 2.99999E-08 | 30.73088871 |
| rs852983 | A | G | 5 | 0.00179325 | 4.20001E-08 | 30.05790943 |
| rs862227 | G | A | 16 | 0.00179141 | 8.79995E-10 | 37.56633875 |
| rs862320 | T | C | 16 | 0.0018218 | 4.40048E-23 | 97.90764667 |
| rs876605 | G | A | 5 | 0.00203598 | 4.20001E-08 | 30.06394428 |
| rs879620 | T | C | 16 | 0.00184223 | 4.90004E-26 | 111.3892601 |
| rs883403 | C | T | 7 | 0.00247511 | 9.49948E-13 | 50.95257145 |
| rs900448 | G | C | 12 | 0.0019396 | 5.79963E-12 | 47.40999807 |
| rs9289630 | C | G | 3 | 0.00183898 | 3.50026E-15 | 61.96281451 |
| rs9294260 | A | G | 6 | 0.00179925 | 2.39994E-13 | 53.64417553 |
| rs9316661 | C | T | 13 | 0.00224739 | 3.80014E-12 | 48.24524123 |
| rs9370243 | T | G | 6 | 0.00325673 | 1.89998E-09 | 36.04467214 |
| rs9392348 | A | G | 6 | 0.00216983 | 0.000000032 | 30.55969994 |
| rs9568867 | A | G | 13 | 0.00268971 | 4.19952E-17 | 70.68770153 |
| rs9584870 | C | T | 13 | 0.00189234 | 1.09999E-08 | 32.65625355 |
| rs9654453 | C | T | 5 | 0.00266903 | 1.7E-10 | 40.76221325 |
| rs9673839 | G | A | 16 | 0.00179863 | 1.29999E-09 | 36.76810215 |
| rs9814758 | G | T | 3 | 0.00187521 | 2.69998E-09 | 35.38790898 |
| rs9843653 | C | T | 3 | 0.00178819 | 6.70039E-28 | 119.8855936 |
| rs9888533 | T | C | 13 | 0.00182556 | 2.99999E-09 | 35.2099309 |
| rs9902846 | T | C | 17 | 0.00192994 | 4.19952E-12 | 48.03291353 |
| rs9916444 | G | C | 17 | 0.0018902 | 1.29999E-09 | 36.84673689 |
| rs9926784 | C | T | 16 | 0.00230452 | 8.70001E-09 | 33.10098558 |

**Table S3**. Genetic instruments used in the analyses for the association of Waist to ratio with Heart failure.

| SNP | EA | OA | chr | SE | *P* value | F |
| --- | --- | --- | --- | --- | --- | --- |
| rs1011731 | A | G | 1 | 0.0033 | 1.09999E-08 | 33.1496786 |
| rs10245353 | A | C | 7 | 0.0042 | 1.6E-10 | 41.32653061 |
| rs10783615 | A | G | 12 | 0.0049 | 7.00003E-13 | 51.02040816 |
| rs11048470 | T | G | 12 | 0.0037 | 6.29941E-12 | 45.65376187 |
| rs1128249 | T | G | 2 | 0.0034 | 1.6E-09 | 38.14878893 |
| rs1294421 | G | T | 6 | 0.0034 | 6.89922E-14 | 54.06574394 |
| rs1316952 | C | T | 12 | 0.0049 | 7.29995E-09 | 32.65306122 |
| rs1440372 | C | T | 15 | 0.0037 | 7.59994E-09 | 32.21329438 |
| rs1563355 | C | T | 1 | 0.0044 | 1.69981E-12 | 49.63842975 |
| rs1569135 | G | A | 2 | 0.0033 | 1E-12 | 52.89256198 |
| rs16996700 | C | T | 20 | 0.0037 | 0.000000016 | 32.21329438 |
| rs17109256 | A | G | 14 | 0.0041 | 2.99999E-08 | 31.46936347 |
| rs17451107 | C | T | 3 | 0.0035 | 3.50026E-11 | 43.18367347 |
| rs2179129 | G | A | 22 | 0.0034 | 1.2E-09 | 38.14878893 |
| rs2207139 | G | A | 6 | 0.0044 | 1.40001E-08 | 32.28305785 |
| rs2287019 | T | C | 19 | 0.0045 | 4.30002E-09 | 33.38271605 |
| rs2765539 | T | C | 1 | 0.0038 | 1.10002E-12 | 50.48476454 |
| rs2972164 | C | T | 3 | 0.0033 | 2.39999E-08 | 33.1496786 |
| rs3786897 | G | A | 19 | 0.0034 | 4.00037E-11 | 41.86851211 |
| rs459193 | G | A | 5 | 0.0038 | 6.00067E-12 | 46.81440443 |
| rs4929927 | G | A | 11 | 0.0034 | 7.59994E-09 | 34.60207612 |
| rs9860730 | G | A | 3 | 0.0036 | 2.80001E-10 | 40.81790123 |
| rs998584 | A | C | 6 | 0.0037 | 5.00035E-15 | 61.43170197 |

**Table S4**. Genetic instruments used in the analyses for the association of Arm fat mass(right) with Heart failure.

| SNP | EA | OA | chr | SE | *P* value | F |
| --- | --- | --- | --- | --- | --- | --- |
| rs10100245 | A | G | 8 | 0.00198462 | 6.4003E-25 | 106.2718701 |
| rs10116857 | A | C | 9 | 0.00408775 | 6.29999E-10 | 38.21630584 |
| rs1013402 | G | A | 11 | 0.00210689 | 5.40008E-47 | 207.2777728 |
| rs10146997 | G | A | 14 | 0.00236745 | 8.4004E-29 | 124.0074555 |
| rs10160660 | C | T | 11 | 0.00241532 | 3.69999E-08 | 30.2820705 |
| rs1017529 | A | C | 17 | 0.00263796 | 9.49948E-11 | 41.93057842 |
| rs10184537 | T | C | 2 | 0.00207237 | 3.19963E-13 | 53.08306905 |
| rs10248298 | A | C | 7 | 0.00203796 | 1E-14 | 59.81033388 |
| rs10251591 | C | T | 7 | 0.00278062 | 0.000000032 | 30.55294992 |
| rs1031881 | G | T | 6 | 0.00261815 | 1.79999E-09 | 36.21390273 |
| rs10402950 | C | T | 19 | 0.00217396 | 7.29962E-15 | 60.52189549 |
| rs10423928 | A | T | 19 | 0.00248325 | 4.30031E-35 | 152.7589487 |
| rs10457469 | A | G | 6 | 0.00196586 | 2.19989E-11 | 44.76084131 |
| rs10505836 | C | A | 12 | 0.00285362 | 9.20005E-09 | 33.01087612 |
| rs10510025 | T | C | 10 | 0.0022854 | 3.80014E-15 | 61.82401523 |
| rs10515237 | G | A | 5 | 0.0021841 | 1.9002E-11 | 45.09533569 |
| rs1057042 | C | T | 16 | 0.00209973 | 1.09999E-08 | 32.57333293 |
| rs1062557 | A | C | 18 | 0.00224755 | 1.59993E-11 | 45.34854352 |
| rs10755151 | T | G | 4 | 0.00197069 | 2.19999E-08 | 31.26990511 |
| rs10756798 | T | C | 9 | 0.00205131 | 6.4003E-14 | 56.23226883 |
| rs10820852 | A | C | 9 | 0.00220125 | 8.19974E-11 | 42.21265448 |
| rs10842240 | C | G | 12 | 0.00306032 | 2.69998E-09 | 35.41026184 |
| rs10878349 | G | A | 12 | 0.00197064 | 1.9002E-11 | 45.06296335 |
| rs10915840 | A | G | 1 | 0.00221019 | 4.30002E-10 | 38.96874369 |
| rs11012732 | G | A | 10 | 0.00208889 | 1.80011E-21 | 90.60458118 |
| rs11017772 | T | C | 10 | 0.00243709 | 4.39997E-10 | 38.91639433 |
| rs11041371 | A | G | 11 | 0.00201738 | 4.49997E-08 | 29.92486642 |
| rs11042030 | C | T | 11 | 0.00220152 | 2.60016E-20 | 85.24968005 |
| rs11057418 | C | G | 12 | 0.00250232 | 1.29999E-09 | 36.84255967 |
| rs11099020 | T | C | 4 | 0.0020499 | 5.79963E-12 | 47.38400511 |
| rs11105842 | A | G | 12 | 0.00204833 | 4.30031E-11 | 43.45966894 |
| rs11142756 | T | C | 9 | 0.00197211 | 1.89998E-08 | 31.57722932 |
| rs11150461 | G | C | 16 | 0.00221619 | 1.09999E-09 | 37.20417049 |
| rs11165643 | T | C | 1 | 0.00199213 | 2.09991E-25 | 108.4833721 |
| rs11218510 | A | G | 11 | 0.00200949 | 6.4003E-11 | 42.68725356 |
| rs112551143 | G | A | 19 | 0.00230103 | 4.49987E-22 | 93.31182341 |
| rs113079574 | T | C | 4 | 0.00250163 | 1.29999E-10 | 41.37739065 |
| rs113230003 | A | G | 19 | 0.00226079 | 1.39991E-17 | 72.83306779 |
| rs113603865 | T | C | 1 | 0.00241334 | 1.80011E-15 | 63.25618692 |
| rs113852095 | T | C | 7 | 0.00257502 | 1.09999E-10 | 41.6256529 |
| rs113866544 | C | T | 17 | 0.00390496 | 3.50026E-20 | 84.71148604 |
| rs114263339 | T | C | 5 | 0.00620863 | 3.80014E-11 | 43.70712495 |
| rs11525873 | C | T | 7 | 0.00331732 | 3.79997E-08 | 30.23819644 |
| rs11538 | G | A | 22 | 0.00260892 | 8.50002E-10 | 37.63952212 |
| rs11603042 | T | G | 11 | 0.0020477 | 3.59998E-08 | 30.34704015 |
| rs11664848 | G | C | 18 | 0.00208513 | 4.49987E-11 | 43.3775048 |
| rs11667638 | G | A | 19 | 0.00197249 | 4.09996E-10 | 39.08618875 |
| rs1167311 | A | G | 1 | 0.00211921 | 1.20005E-18 | 77.70226045 |
| rs11691869 | A | C | 2 | 0.00204493 | 1.69981E-19 | 81.57165541 |
| rs11709402 | G | A | 3 | 0.0021957 | 1.39991E-23 | 100.1786104 |
| rs117176448 | G | C | 8 | 0.00333132 | 2E-10 | 40.47023981 |
| rs117342986 | T | C | 16 | 0.00642676 | 4.09996E-08 | 30.09960142 |
| rs11757278 | C | T | 6 | 0.00213589 | 1.7E-10 | 40.75173931 |
| rs11775287 | T | C | 8 | 0.00197487 | 3.69999E-11 | 43.78660027 |
| rs11781222 | C | T | 8 | 0.00286342 | 6.70039E-13 | 51.64142625 |
| rs11782074 | T | G | 8 | 0.00205449 | 1.9002E-12 | 49.59854882 |
| rs1191600 | A | C | 14 | 0.00201512 | 3.40017E-11 | 43.90397386 |
| rs12001437 | C | T | 9 | 0.00203869 | 1.09999E-08 | 32.72509592 |
| rs1205593 | C | T | 1 | 0.0022968 | 6.29999E-10 | 38.23964177 |
| rs12072739 | G | A | 1 | 0.00235343 | 8.60003E-14 | 55.65991924 |
| rs12140153 | T | G | 1 | 0.00343936 | 7.39946E-26 | 110.5562344 |
| rs12144626 | C | T | 1 | 0.00199273 | 5.79963E-17 | 70.03715929 |
| rs12259464 | A | G | 10 | 0.00197556 | 1.40001E-09 | 36.71443738 |
| rs1229984 | C | T | 4 | 0.00595902 | 2.69998E-10 | 39.8851358 |
| rs12364470 | G | T | 11 | 0.0026505 | 1.39991E-12 | 50.13473447 |
| rs12427676 | G | A | 13 | 0.00197844 | 3.69999E-09 | 34.77067062 |
| rs12462975 | A | G | 19 | 0.0021081 | 4.49987E-23 | 97.86268952 |
| rs12477385 | T | G | 2 | 0.00235178 | 8.10009E-09 | 33.25232918 |
| rs12506689 | A | G | 4 | 0.00204034 | 6.4003E-15 | 60.76087658 |
| rs12616219 | A | C | 2 | 0.00196148 | 3.29997E-09 | 35.02490456 |
| rs12659431 | A | G | 5 | 0.00196667 | 2.1E-10 | 40.34023133 |
| rs12679106 | T | G | 8 | 0.00217471 | 2.99985E-23 | 98.6617994 |
| rs12881629 | G | A | 14 | 0.00356842 | 1.5E-09 | 36.48106919 |
| rs12883788 | T | C | 14 | 0.00198113 | 2.80027E-19 | 80.59555542 |
| rs12885458 | G | T | 14 | 0.00197036 | 2.29985E-13 | 53.71373186 |
| rs12956148 | A | C | 18 | 0.00219918 | 2.60016E-11 | 44.43231954 |
| rs1296328 | C | A | 4 | 0.0019874 | 7.39946E-18 | 74.10858618 |
| rs12993643 | A | G | 2 | 0.00214709 | 5.1E-09 | 34.14746977 |
| rs13033310 | A | G | 2 | 0.00227038 | 3.29997E-08 | 30.51711073 |
| rs13097918 | A | T | 3 | 0.00240232 | 2.39999E-10 | 40.07688114 |
| rs13107325 | T | C | 4 | 0.00373396 | 2.09991E-28 | 122.1601663 |
| rs13156484 | A | G | 5 | 0.00198672 | 1.20005E-13 | 55.08282762 |
| rs13292699 | C | A | 9 | 0.00198705 | 5.40008E-27 | 115.7325727 |
| rs1329733 | G | A | 9 | 0.00197319 | 6.4998E-12 | 47.18704529 |
| rs1330199 | T | G | 9 | 0.00197504 | 4.90004E-08 | 29.75347211 |
| rs13375176 | C | T | 1 | 0.00245339 | 1.09999E-08 | 32.69513236 |
| rs13389219 | T | C | 2 | 0.00200532 | 1.69981E-12 | 49.81854186 |
| rs13427822 | G | A | 2 | 0.00222965 | 6.20012E-11 | 42.76273634 |
| rs1357079 | C | T | 3 | 0.00198598 | 1E-15 | 64.42172388 |
| rs139911 | T | C | 22 | 0.00200144 | 2.1E-09 | 35.87096881 |
| rs141622900 | A | G | 19 | 0.00452439 | 1.09999E-09 | 37.14644648 |
| rs1436348 | G | A | 3 | 0.0019897 | 1.10002E-12 | 50.58879121 |
| rs1441264 | A | G | 13 | 0.00204644 | 1.69981E-17 | 72.4208951 |
| rs1446585 | G | A | 2 | 0.00223536 | 1.89998E-10 | 40.61883948 |
| rs145350287 | A | T | 12 | 0.00503435 | 2.49977E-16 | 67.13752177 |
| rs1458156 | T | C | 12 | 0.00196766 | 5.19996E-13 | 52.12249107 |
| rs1459190 | A | G | 5 | 0.00198397 | 9.09913E-12 | 46.51875694 |
| rs1470705 | C | T | 14 | 0.00226916 | 4.90004E-09 | 34.24835753 |
| rs1503526 | C | T | 5 | 0.0019654 | 1.10002E-13 | 55.26372358 |
| rs1522569 | G | T | 4 | 0.002538 | 2.30001E-08 | 31.19297319 |
| rs1554654 | T | C | 3 | 0.00196964 | 6.49995E-10 | 38.16996799 |
| rs1657930 | A | G | 15 | 0.00247162 | 2.1E-09 | 35.89171538 |
| rs16824376 | T | C | 2 | 0.0024861 | 2.19999E-08 | 31.27959145 |
| rs16868443 | C | G | 7 | 0.00204736 | 2E-10 | 40.49677198 |
| rs16916303 | G | A | 9 | 0.00306116 | 6.19998E-09 | 33.78328597 |
| rs17399739 | G | A | 10 | 0.00388675 | 2E-10 | 40.41939901 |
| rs17668356 | G | C | 3 | 0.00277686 | 5.00035E-15 | 61.25525901 |
| rs17770336 | T | C | 9 | 0.00209912 | 4.70002E-28 | 120.607088 |
| rs1778830 | A | G | 1 | 0.00204475 | 2.5E-09 | 35.55069548 |
| rs1782508 | G | C | 11 | 0.00206534 | 1.20005E-11 | 45.94473309 |
| rs1801282 | G | C | 3 | 0.00301695 | 5.00035E-18 | 74.87286968 |
| rs1813039 | A | G | 8 | 0.00217721 | 7.29995E-09 | 33.44306015 |
| rs1840126 | C | A | 2 | 0.00197701 | 7.00003E-09 | 33.54643245 |
| rs1883711 | C | G | 20 | 0.0057531 | 9.09997E-10 | 37.51831327 |
| rs1884389 | T | C | 20 | 0.00199215 | 2.30001E-08 | 31.2541697 |
| rs194656 | A | G | 1 | 0.00243289 | 1.29999E-08 | 32.34222412 |
| rs2051559 | C | T | 4 | 0.00290173 | 2.69998E-08 | 30.90203245 |
| rs2052607 | A | G | 18 | 0.00207715 | 5.60015E-17 | 70.09602846 |
| rs2075466 | C | G | 16 | 0.00222323 | 2.59998E-09 | 35.4988127 |
| rs2095484 | C | T | 9 | 0.0020217 | 0.000000025 | 31.06971973 |
| rs2118793 | A | C | 5 | 0.00199842 | 4.70002E-08 | 29.82376918 |
| rs2126320 | A | G | 8 | 0.0021605 | 1.9002E-11 | 45.0616565 |
| rs2133561 | T | A | 5 | 0.00203581 | 2.80027E-17 | 71.5105042 |
| rs213518 | C | T | 7 | 0.0027889 | 6.59933E-11 | 42.63145521 |
| rs2135877 | G | A | 15 | 0.00209894 | 4.39997E-10 | 38.9257948 |
| rs215634 | G | A | 7 | 0.00202374 | 1.29987E-13 | 54.92631107 |
| rs2216931 | A | C | 2 | 0.00207503 | 2.49977E-15 | 62.64674858 |
| rs2237025 | C | T | 4 | 0.00199127 | 4.79999E-10 | 38.75340263 |
| rs2267373 | T | C | 22 | 0.00200032 | 5.70033E-14 | 56.45640601 |
| rs2272168 | C | G | 7 | 0.0027402 | 1.69981E-12 | 49.76781924 |
| rs2289379 | T | C | 7 | 0.00201793 | 1.2E-10 | 41.41637134 |
| rs2291127 | T | C | 3 | 0.00270174 | 0.000000012 | 32.4393098 |
| rs2307111 | C | T | 5 | 0.00201004 | 1.69981E-41 | 182.069511 |
| rs2312193 | C | T | 3 | 0.00201803 | 5.40008E-22 | 92.9295121 |
| rs2439823 | G | A | 10 | 0.00197961 | 5.30029E-21 | 88.4071675 |
| rs2494196 | A | C | 1 | 0.00216942 | 5.50047E-17 | 70.16461993 |
| rs2499468 | A | C | 6 | 0.00206224 | 4.00037E-13 | 52.63114681 |
| rs2507909 | G | A | 11 | 0.0019869 | 2.80001E-09 | 35.29085908 |
| rs2568958 | A | G | 1 | 0.00200115 | 2.19989E-24 | 103.8084162 |
| rs2678204 | G | T | 1 | 0.00206992 | 1.69981E-27 | 118.0284812 |
| rs271590 | T | C | 5 | 0.00212865 | 6.19998E-10 | 38.24318307 |
| rs273505 | C | T | 19 | 0.00199378 | 7.39946E-20 | 83.19934421 |
| rs28350 | G | A | 3 | 0.0025669 | 1.9002E-12 | 49.63251628 |
| rs28366156 | C | T | 6 | 0.00291252 | 5.79963E-19 | 79.15055804 |
| rs2846139 | T | C | 11 | 0.00238756 | 1.80011E-12 | 49.71078001 |
| rs2861685 | C | T | 2 | 0.00198719 | 2.70023E-16 | 67.04077521 |
| rs286535 | C | G | 11 | 0.00287249 | 1.09999E-09 | 37.10606334 |
| rs28670671 | C | T | 9 | 0.00225136 | 0.00000002 | 31.45102467 |
| rs28687152 | C | G | 15 | 0.00223264 | 7.00003E-10 | 38.00874042 |
| rs28696937 | T | C | 9 | 0.00324942 | 9.29994E-09 | 32.97703312 |
| rs28826668 | T | C | 15 | 0.00218273 | 9.40005E-09 | 32.95880257 |
| rs2903738 | T | A | 19 | 0.00237077 | 2.90001E-08 | 30.79372885 |
| rs2917677 | T | C | 16 | 0.00200069 | 4.49987E-24 | 102.4155857 |
| rs2930227 | T | G | 16 | 0.00241249 | 8.30004E-09 | 33.1922021 |
| rs2954021 | G | A | 8 | 0.00196588 | 2.60016E-15 | 62.5551381 |
| rs3107610 | G | C | 11 | 0.0021838 | 1.40001E-09 | 36.61086709 |
| rs3113509 | T | C | 4 | 0.00221793 | 5.40008E-12 | 47.53152916 |
| rs329118 | T | C | 5 | 0.00199211 | 3.90032E-13 | 52.6987183 |
| rs329651 | T | G | 11 | 0.00248825 | 2.39999E-08 | 31.14072825 |
| rs34215484 | C | T | 8 | 0.00398923 | 1.40001E-08 | 32.17552952 |
| rs34234296 | A | G | 2 | 0.00202923 | 5.30029E-13 | 52.10320814 |
| rs34483452 | A | C | 5 | 0.0028861 | 6.59933E-29 | 124.498615 |
| rs34517439 | A | C | 1 | 0.00303478 | 1.10002E-47 | 210.3712816 |
| rs34571768 | A | G | 8 | 0.00299067 | 4.09996E-08 | 30.11301726 |
| rs34769775 | T | C | 15 | 0.00215372 | 2.49977E-12 | 49.00754073 |
| rs34882821 | T | G | 9 | 0.00208392 | 2.30001E-08 | 31.25876202 |
| rs35154326 | G | A | 16 | 0.00222133 | 4.30031E-12 | 47.97676243 |
| rs35537311 | T | C | 17 | 0.00202239 | 0.000000005 | 34.19310865 |
| rs35697691 | G | C | 15 | 0.00349974 | 3.59998E-13 | 52.87321875 |
| rs35882248 | T | C | 2 | 0.00210794 | 6.79986E-17 | 69.73122286 |
| rs36007635 | A | G | 6 | 0.00285098 | 9.20026E-11 | 41.99108006 |
| rs3729793 | G | C | 7 | 0.00337552 | 0.000000015 | 32.0046099 |
| rs3730071 | A | C | 12 | 0.00574077 | 6.29999E-10 | 38.22328358 |
| rs3739514 | A | G | 9 | 0.00208321 | 1.09999E-09 | 37.09483049 |
| rs3759094 | T | C | 12 | 0.00208136 | 1.39991E-12 | 50.12756472 |
| rs3766823 | A | G | 1 | 0.00259905 | 1.20005E-11 | 45.98739812 |
| rs3770821 | C | T | 2 | 0.0020926 | 4.00037E-15 | 61.72074853 |
| rs3784710 | C | T | 15 | 0.00234641 | 2.70023E-34 | 149.1240036 |
| rs3803286 | G | A | 14 | 0.00208612 | 3.19963E-15 | 62.16750865 |
| rs3807566 | T | G | 7 | 0.00198445 | 3.29989E-11 | 43.99029271 |
| rs3814883 | T | C | 16 | 0.00197279 | 3.90032E-38 | 166.6730879 |
| rs3826408 | T | C | 17 | 0.00197404 | 4.79954E-11 | 43.27451521 |
| rs3848453 | C | T | 17 | 0.00200096 | 4.90004E-08 | 29.74646576 |
| rs3892367 | A | G | 12 | 0.00196584 | 3.80014E-14 | 57.2878901 |
| rs3935190 | A | G | 17 | 0.00198523 | 6.4003E-15 | 60.77484294 |
| rs394608 | C | T | 21 | 0.00198273 | 1.50003E-18 | 77.28895763 |
| rs396354 | C | T | 2 | 0.00217888 | 4.60045E-11 | 43.35059154 |
| rs40071 | C | T | 5 | 0.00256655 | 6.70039E-16 | 65.21889497 |
| rs4055791 | T | C | 13 | 0.00199675 | 6.59933E-15 | 60.71623178 |
| rs4123668 | C | T | 3 | 0.0020033 | 1.7E-10 | 40.76841305 |
| rs41279738 | G | T | 1 | 0.00619384 | 7.10068E-30 | 128.9185978 |
| rs4261944 | G | T | 4 | 0.00204473 | 0.000000015 | 31.99037539 |
| rs429343 | G | A | 2 | 0.00198687 | 1.50003E-14 | 59.07937109 |
| rs4307239 | G | A | 7 | 0.00197659 | 0.000000017 | 31.7709904 |
| rs4377779 | C | T | 6 | 0.00206375 | 2.60016E-13 | 53.45868325 |
| rs4397962 | C | T | 13 | 0.00238114 | 1.40001E-08 | 32.19909744 |
| rs4411908 | C | A | 3 | 0.00202703 | 2.69998E-09 | 35.39411426 |
| rs4430672 | C | T | 14 | 0.0024681 | 0.00000002 | 31.48055159 |
| rs4456769 | T | C | 20 | 0.00208896 | 6.29941E-14 | 56.28519047 |
| rs4482463 | A | C | 2 | 0.00368829 | 5.60015E-17 | 70.11234061 |
| rs4562625 | G | C | 1 | 0.00201412 | 1E-10 | 41.80594624 |
| rs4605363 | C | A | 2 | 0.00206624 | 7.00003E-13 | 51.54612311 |
| rs4658403 | T | C | 1 | 0.00263545 | 1E-10 | 41.80268056 |
| rs4672338 | T | C | 2 | 0.00207592 | 0.00000004 | 30.13745122 |
| rs4718964 | T | G | 7 | 0.00200232 | 8.30042E-12 | 46.70386399 |
| rs4722398 | T | C | 7 | 0.00285942 | 4.60045E-12 | 47.83372277 |
| rs4725984 | C | T | 7 | 0.0020617 | 6.79986E-14 | 56.11293949 |
| rs4800191 | C | G | 18 | 0.0020625 | 4.60002E-08 | 29.87808357 |
| rs4844809 | C | G | 1 | 0.0028961 | 3.50002E-08 | 30.4049751 |
| rs4858940 | C | T | 3 | 0.0030812 | 4.49987E-14 | 56.95908817 |
| rs4876611 | G | A | 8 | 0.00219163 | 8.69961E-26 | 110.227819 |
| rs4900590 | T | C | 14 | 0.00210486 | 3.79997E-10 | 39.21006577 |
| rs4908672 | T | C | 1 | 0.00201035 | 0.00000001 | 32.77019312 |
| rs4936175 | C | T | 11 | 0.00197777 | 2.69998E-09 | 35.39388736 |
| rs4953424 | A | G | 2 | 0.00197281 | 6.49995E-10 | 38.16254726 |
| rs4982753 | T | C | 14 | 0.0022709 | 5.19996E-09 | 34.09554038 |
| rs55707359 | G | T | 11 | 0.00807223 | 2.99999E-10 | 39.67447206 |
| rs55938344 | C | A | 12 | 0.00231459 | 1.80011E-11 | 45.19530992 |
| rs56102300 | T | G | 2 | 0.00197058 | 3.59998E-08 | 30.35788662 |
| rs56187939 | G | A | 19 | 0.00213848 | 0.000000012 | 32.43918453 |
| rs56356382 | C | T | 19 | 0.00250724 | 2.90001E-12 | 48.7419211 |
| rs56374036 | G | A | 20 | 0.00242296 | 1.6E-09 | 36.46475847 |
| rs585066 | T | C | 19 | 0.0019752 | 9.90011E-10 | 37.34712286 |
| rs587271 | T | C | 1 | 0.0022057 | 1.2E-09 | 36.90657521 |
| rs58862095 | T | C | 7 | 0.00199656 | 1.9002E-22 | 94.99844059 |
| rs59237168 | C | T | 17 | 0.00239517 | 1.40001E-10 | 41.11465399 |
| rs6058209 | A | G | 20 | 0.00210889 | 2.99985E-12 | 48.71542087 |
| rs60661769 | A | T | 4 | 0.00205573 | 4.70002E-11 | 43.31430462 |
| rs6069037 | A | C | 20 | 0.00222035 | 1.89998E-08 | 31.61646746 |
| rs61754230 | T | C | 12 | 0.00706767 | 8.10009E-10 | 37.73471623 |
| rs61791109 | C | T | 3 | 0.00219112 | 5.19996E-16 | 65.72707617 |
| rs61871615 | T | C | 10 | 0.00357256 | 2.19989E-14 | 58.33129081 |
| rs61903695 | G | A | 11 | 0.00225822 | 4.90004E-11 | 43.20444508 |
| rs61969510 | C | T | 13 | 0.00221224 | 5.00035E-13 | 52.21020112 |
| rs61983990 | A | G | 14 | 0.00359061 | 9.20005E-10 | 37.49281715 |
| rs62107261 | C | T | 2 | 0.00458452 | 5.50047E-79 | 354.0884135 |
| rs62407565 | G | A | 6 | 0.00219494 | 1.5E-10 | 40.9785699 |
| rs62489440 | A | G | 8 | 0.00264918 | 3.59998E-09 | 34.81106011 |
| rs6429425 | T | G | 1 | 0.00282858 | 2.90001E-09 | 35.2711473 |
| rs6443750 | C | T | 3 | 0.00248929 | 0.000000025 | 31.07573504 |
| rs6469351 | T | C | 8 | 0.00209751 | 1.40001E-08 | 32.2555936 |
| rs6480794 | A | C | 10 | 0.00197276 | 1.89998E-08 | 31.54446561 |
| rs6536575 | C | T | 4 | 0.00196612 | 2.1E-09 | 35.86816559 |
| rs6545714 | A | G | 2 | 0.00200519 | 5.00035E-24 | 102.2174354 |
| rs6561937 | A | T | 13 | 0.00229031 | 0.000000002 | 35.94102779 |
| rs6575340 | A | G | 14 | 0.00204973 | 2.39994E-21 | 89.99124188 |
| rs66679256 | T | C | 4 | 0.00197621 | 2.99985E-19 | 80.41911312 |
| rs6669189 | T | C | 1 | 0.00200613 | 1.50003E-15 | 63.69378682 |
| rs6696828 | C | G | 1 | 0.00212857 | 3.79997E-09 | 34.71547334 |
| rs6744646 | G | A | 2 | 0.00259812 | 1.20005E-88 | 398.405054 |
| rs67560975 | C | T | 17 | 0.00308128 | 2.39999E-09 | 35.6341516 |
| rs67605820 | G | A | 3 | 0.00212658 | 1.7E-10 | 40.84143833 |
| rs67609008 | C | T | 10 | 0.0021894 | 1.80011E-13 | 54.24234365 |
| rs6774894 | A | T | 3 | 0.00204479 | 2.49977E-11 | 44.55188245 |
| rs67807996 | A | G | 1 | 0.00206841 | 2.49977E-16 | 67.18481105 |
| rs6790206 | G | A | 3 | 0.00196193 | 2.19999E-09 | 35.76868256 |
| rs6840236 | C | T | 4 | 0.00197017 | 1.6E-09 | 36.36135849 |
| rs6954290 | T | G | 7 | 0.00210473 | 2.19999E-10 | 40.26076235 |
| rs6973656 | G | A | 7 | 0.00200724 | 0.000000012 | 32.45270814 |
| rs6973700 | G | A | 7 | 0.00243164 | 1E-10 | 41.74969689 |
| rs698147 | G | A | 5 | 0.0019733 | 4.79999E-10 | 38.73742406 |
| rs7027304 | T | C | 9 | 0.00207474 | 2.49977E-12 | 49.01363157 |
| rs7034554 | G | A | 9 | 0.00203042 | 1E-10 | 41.79708279 |
| rs7038943 | C | T | 9 | 0.00207422 | 2.09991E-12 | 49.43197254 |
| rs704061 | C | T | 12 | 0.00197373 | 3.29989E-19 | 80.24419056 |
| rs7070670 | T | C | 10 | 0.00210691 | 1.2E-09 | 36.96690328 |
| rs7124681 | A | C | 11 | 0.00199472 | 1.69981E-35 | 154.5962716 |
| rs7132908 | A | G | 12 | 0.00202166 | 8.99912E-48 | 210.8346634 |
| rs7138383 | A | G | 12 | 0.00225861 | 3.40017E-13 | 52.96822979 |
| rs7171864 | A | G | 15 | 0.00208714 | 2.39994E-14 | 58.17977696 |
| rs719802 | C | T | 11 | 0.00201736 | 1.29987E-11 | 45.8649394 |
| rs7218014 | C | T | 17 | 0.0024773 | 2.19989E-21 | 90.19670387 |
| rs725959 | T | G | 9 | 0.0020065 | 0.000000012 | 32.42100602 |
| rs72634826 | A | G | 1 | 0.00226749 | 1.20005E-15 | 64.12199026 |
| rs72665129 | A | T | 13 | 0.00271504 | 1.7E-10 | 40.78125266 |
| rs72757416 | C | A | 15 | 0.00241958 | 3.50002E-09 | 34.87966691 |
| rs72817602 | A | G | 17 | 0.00218619 | 1.29999E-09 | 36.7795514 |
| rs72868406 | G | C | 2 | 0.00322693 | 0.000000025 | 31.03422129 |
| rs73052033 | C | T | 3 | 0.00253072 | 5.90065E-22 | 92.76784985 |
| rs73142879 | T | C | 20 | 0.00250742 | 8.4004E-25 | 105.7425508 |
| rs73213484 | T | A | 4 | 0.00282011 | 5.10035E-17 | 70.28074053 |
| rs73985439 | C | A | 2 | 0.00212882 | 3.69999E-10 | 39.28756631 |
| rs7442885 | G | C | 5 | 0.00239789 | 8.99912E-20 | 82.82139299 |
| rs745249 | T | C | 2 | 0.00218597 | 2.19989E-12 | 49.31759876 |
| rs7498665 | G | A | 16 | 0.0020085 | 7.10068E-53 | 234.2324713 |
| rs7519259 | A | G | 1 | 0.00197307 | 1.10002E-12 | 50.57280291 |
| rs7537581 | A | C | 1 | 0.00197982 | 1.7E-10 | 40.76983302 |
| rs754635 | G | C | 3 | 0.00309099 | 7.19946E-13 | 51.47573538 |
| rs7561278 | C | T | 2 | 0.0023877 | 1.29987E-11 | 45.84691344 |
| rs7571496 | G | A | 2 | 0.00224242 | 5.39995E-09 | 34.05284212 |
| rs7619139 | A | T | 3 | 0.00199883 | 1.29987E-13 | 54.77746081 |
| rs763750 | T | C | 12 | 0.00200566 | 4.70002E-08 | 29.84643596 |
| rs765876 | G | A | 6 | 0.00196387 | 9.49948E-12 | 46.42428015 |
| rs76702514 | G | C | 1 | 0.0024193 | 1.79999E-10 | 40.63673838 |
| rs7683836 | A | G | 4 | 0.00198275 | 1.79999E-09 | 36.16905439 |
| rs7730898 | A | G | 5 | 0.00220993 | 1.29987E-17 | 73.0081177 |
| rs7761673 | A | T | 6 | 0.00237552 | 1.89998E-08 | 31.63446875 |
| rs7773530 | T | C | 6 | 0.00205407 | 0.000000017 | 31.81973379 |
| rs7776021 | A | G | 6 | 0.00216976 | 0.000000002 | 35.96428141 |
| rs77927866 | A | G | 12 | 0.00213365 | 8.19993E-09 | 33.22986321 |
| rs7817485 | C | T | 8 | 0.00202098 | 0.000000032 | 30.59173998 |
| rs7819514 | A | G | 8 | 0.00208796 | 3.09999E-08 | 30.62054103 |
| rs7828631 | T | C | 8 | 0.00314797 | 2.39999E-08 | 31.12976172 |
| rs7893571 | T | G | 10 | 0.00208934 | 1.80011E-11 | 45.17921332 |
| rs7944782 | G | T | 11 | 0.00197551 | 1.80011E-15 | 63.26123187 |
| rs7946756 | A | T | 11 | 0.00205074 | 8.30004E-09 | 33.20983871 |
| rs79518326 | A | C | 1 | 0.00594801 | 4.79954E-12 | 47.78540101 |
| rs79780963 | T | C | 10 | 0.00367505 | 3.40001E-10 | 39.42844162 |
| rs801738 | G | C | 11 | 0.0020506 | 1.50003E-15 | 63.63302538 |
| rs8076669 | C | T | 17 | 0.00198452 | 1E-10 | 41.75178986 |
| rs8093356 | T | C | 18 | 0.00235301 | 4E-10 | 39.12035956 |
| rs8096564 | T | G | 18 | 0.00216327 | 3.40017E-11 | 43.92515374 |
| rs8133137 | G | A | 21 | 0.00208688 | 5.30029E-11 | 43.05351416 |
| rs815163 | C | T | 1 | 0.00197513 | 1.39991E-14 | 59.18322204 |
| rs845084 | A | G | 10 | 0.00225479 | 6.4003E-12 | 47.2005118 |
| rs854917 | T | C | 6 | 0.0022375 | 4.20001E-10 | 39.03024409 |
| rs879620 | T | C | 16 | 0.00202429 | 5.70033E-33 | 143.0757706 |
| rs889495 | C | T | 16 | 0.00201919 | 5.69994E-10 | 38.43767871 |
| rs9294260 | A | G | 6 | 0.00197635 | 1.39991E-12 | 50.15894745 |
| rs9340799 | G | A | 6 | 0.00205374 | 0.000000015 | 32.10967635 |
| rs935166 | A | G | 2 | 0.0019624 | 4.10015E-16 | 66.16559032 |
| rs9405542 | T | C | 6 | 0.00245337 | 2.69998E-08 | 30.88760137 |
| rs946185 | G | A | 10 | 0.00201524 | 5.30029E-11 | 43.06893002 |
| rs947088 | T | G | 20 | 0.00219449 | 1.5E-09 | 36.56906914 |
| rs9522183 | T | G | 13 | 0.00199603 | 1.39991E-12 | 50.13780509 |
| rs9532583 | G | T | 13 | 0.00217163 | 6.90001E-10 | 38.03732906 |
| rs9556582 | A | G | 13 | 0.00199231 | 9.30037E-11 | 41.9541827 |
| rs9568867 | A | G | 13 | 0.0029568 | 4.70002E-26 | 111.4549791 |
| rs9654453 | C | T | 5 | 0.00293151 | 0.000000025 | 31.09367903 |
| rs9672778 | T | C | 15 | 0.00230424 | 2.90001E-08 | 30.78741436 |
| rs9673839 | G | A | 16 | 0.00197664 | 5.19996E-10 | 38.61793978 |
| rs9788550 | C | G | 14 | 0.00228834 | 2.29985E-21 | 90.03732053 |
| rs9839081 | A | G | 3 | 0.00212802 | 2.69998E-09 | 35.39568362 |
| rs9843653 | C | T | 3 | 0.00196353 | 1.29987E-38 | 168.9074549 |
| rs9876664 | T | G | 3 | 0.00202954 | 9.8992E-16 | 64.45948728 |
| rs9916444 | G | C | 17 | 0.00207764 | 8.10028E-11 | 42.23085545 |
| rs9926784 | C | T | 16 | 0.00253236 | 1.29987E-14 | 59.3151062 |
| rs9968060 | T | C | 3 | 0.00208526 | 1.20005E-12 | 50.4607723 |

**Table S5**. Genetic instruments used in the analyses for the association of Leg fat mass (right) with Heart failure.

| SNP | EA | OA | chr | SE | *P* value | F |
| --- | --- | --- | --- | --- | --- | --- |
| rs10100245 | A | G | 8 | 0.001618 | 6.29941E-21 | 88.08019714 |
| rs10160769 | C | G | 11 | 0.00196385 | 0.000000021 | 31.43669627 |
| rs10184537 | T | C | 2 | 0.00168983 | 1.50003E-12 | 50.05606671 |
| rs10209821 | T | C | 2 | 0.0016825 | 8.80035E-18 | 73.76180903 |
| rs1022185 | A | G | 20 | 0.00188683 | 3.59998E-10 | 39.29878435 |
| rs1025065 | G | T | 16 | 0.00167218 | 0.000000032 | 30.56691009 |
| rs10423928 | A | T | 19 | 0.00202485 | 2.19989E-39 | 172.3751065 |
| rs10499014 | G | C | 6 | 0.00181735 | 2.70023E-12 | 48.91337367 |
| rs10505836 | C | A | 12 | 0.00232698 | 8.80035E-11 | 42.06823904 |
| rs1057042 | C | T | 16 | 0.00171223 | 3.29997E-10 | 39.50881958 |
| rs1062557 | A | C | 18 | 0.00183247 | 1.40001E-08 | 32.18353386 |
| rs10756798 | T | C | 9 | 0.00167255 | 2.39994E-13 | 53.62209391 |
| rs10760277 | T | C | 9 | 0.00165256 | 1.09999E-10 | 41.71332624 |
| rs10766451 | T | C | 11 | 0.00172601 | 0.000000002 | 35.99332596 |
| rs10771041 | T | C | 12 | 0.00250438 | 2.90001E-08 | 30.7718853 |
| rs10779795 | G | A | 1 | 0.00169316 | 7.70016E-13 | 51.3686174 |
| rs10820852 | A | C | 9 | 0.00179481 | 3.59998E-10 | 39.30550341 |
| rs10846458 | A | T | 12 | 0.00175067 | 1.89998E-09 | 36.07530195 |
| rs10915840 | A | G | 1 | 0.00180238 | 8.70001E-10 | 37.60282462 |
| rs10947793 | G | A | 6 | 0.00166628 | 7.10068E-15 | 60.56988351 |
| rs10951992 | C | T | 7 | 0.0016351 | 4.79999E-09 | 34.27335889 |
| rs10992854 | C | T | 9 | 0.00172844 | 4.10015E-12 | 48.0875536 |
| rs11001963 | T | C | 10 | 0.00163703 | 2.19999E-10 | 40.28340552 |
| rs11012732 | G | A | 10 | 0.00170311 | 1.69981E-28 | 122.5640403 |
| rs1106761 | A | G | 8 | 0.00166876 | 1.99986E-13 | 54.04817229 |
| rs11071646 | A | G | 15 | 0.00546787 | 4.70002E-08 | 29.82843771 |
| rs11075252 | G | A | 16 | 0.00178115 | 0.00000002 | 31.48097319 |
| rs11099020 | T | C | 4 | 0.00167193 | 2.49977E-11 | 44.52981593 |
| rs11105842 | A | G | 12 | 0.00167038 | 2.99985E-11 | 44.16895409 |
| rs111258054 | T | C | 11 | 0.00209829 | 1E-10 | 41.82923081 |
| rs11135450 | G | A | 5 | 0.00170899 | 5.30029E-12 | 47.55485603 |
| rs11165643 | T | C | 1 | 0.00162454 | 3.80014E-22 | 93.6434524 |
| rs111909661 | C | G | 11 | 0.00195732 | 1.5E-09 | 36.56241503 |
| rs11245344 | T | C | 10 | 0.00162237 | 3.09999E-09 | 35.12715905 |
| rs113079574 | T | C | 4 | 0.00204038 | 5.49997E-10 | 38.50532223 |
| rs113230003 | A | G | 19 | 0.00184351 | 3.50026E-13 | 52.91982216 |
| rs113624107 | A | G | 14 | 0.00191976 | 4.39997E-09 | 34.45022726 |
| rs113866544 | C | T | 17 | 0.00318404 | 2.39994E-19 | 80.86968977 |
| rs11603984 | T | G | 11 | 0.00234737 | 6.1E-10 | 38.29489992 |
| rs11619722 | C | T | 13 | 0.00175109 | 2.09991E-14 | 58.46887767 |
| rs11656076 | A | G | 17 | 0.00192184 | 0.000000002 | 35.93733737 |
| rs11656758 | G | A | 17 | 0.00169119 | 1.40001E-10 | 41.11745755 |
| rs11664848 | G | C | 18 | 0.00170001 | 3.2E-09 | 35.05782855 |
| rs1167311 | A | G | 1 | 0.00172819 | 1.10002E-20 | 87.04228454 |
| rs11691869 | A | C | 2 | 0.00166743 | 4.40048E-23 | 97.88705375 |
| rs11698185 | C | T | 20 | 0.00161014 | 1.99986E-14 | 58.57980326 |
| rs11704728 | T | C | 22 | 0.00202984 | 9.8992E-12 | 46.34986536 |
| rs117176448 | G | C | 8 | 0.00271595 | 7.19946E-13 | 51.49150319 |
| rs117342986 | T | C | 16 | 0.00524068 | 1.29999E-10 | 41.34791244 |
| rs11757278 | C | T | 6 | 0.00174151 | 4.30002E-10 | 38.98546769 |
| rs11767811 | A | G | 7 | 0.00207722 | 8.69961E-12 | 46.60871161 |
| rs11779446 | G | A | 8 | 0.00218663 | 5.00035E-14 | 56.74826773 |
| rs11841757 | G | A | 13 | 0.00187987 | 1.29987E-11 | 45.77155489 |
| rs11914525 | G | A | 3 | 0.00167349 | 6.20012E-21 | 88.11183551 |
| rs11926300 | G | A | 3 | 0.00181324 | 0.00000002 | 31.46482107 |
| rs12025220 | A | G | 1 | 0.00162508 | 2.99985E-16 | 66.77400339 |
| rs12031634 | A | G | 1 | 0.00175802 | 9.69996E-10 | 37.38145312 |
| rs12042959 | G | A | 1 | 0.00228994 | 1.5E-09 | 36.58328468 |
| rs12072739 | G | A | 1 | 0.00191922 | 2.19989E-12 | 49.3248559 |
| rs12140153 | T | G | 1 | 0.00280473 | 4.49987E-22 | 93.31427882 |
| rs12144626 | C | T | 1 | 0.00162508 | 6.29941E-14 | 56.27400138 |
| rs12173616 | G | A | 6 | 0.00360227 | 2.19999E-08 | 31.27358791 |
| rs12254441 | T | C | 10 | 0.00171647 | 9.59997E-10 | 37.41379047 |
| rs12286929 | G | A | 11 | 0.00160765 | 5.39995E-09 | 34.05754996 |
| rs1229984 | C | T | 4 | 0.0048603 | 6.4003E-13 | 51.70806257 |
| rs12316080 | T | C | 12 | 0.00169592 | 2.99985E-11 | 44.19373893 |
| rs12427047 | T | C | 12 | 0.00186881 | 2.80027E-16 | 66.91007314 |
| rs12475388 | A | G | 2 | 0.00160757 | 1.2E-10 | 41.48120543 |
| rs12477385 | T | G | 2 | 0.00191769 | 5.90065E-12 | 47.36305387 |
| rs12541408 | C | T | 8 | 0.00172381 | 4.90004E-10 | 38.71439702 |
| rs1263629 | G | A | 2 | 0.00229065 | 7.19996E-10 | 37.9708611 |
| rs12679106 | T | G | 8 | 0.00177299 | 1.10002E-25 | 109.7945213 |
| rs12724928 | C | T | 1 | 0.00198006 | 1.59993E-11 | 45.42076243 |
| rs12877270 | A | G | 13 | 0.00162728 | 2.29985E-11 | 44.6845682 |
| rs12878084 | T | C | 14 | 0.002264 | 6.1E-09 | 33.8067255 |
| rs12881629 | G | A | 14 | 0.0029096 | 4.70002E-11 | 43.3101147 |
| rs12889080 | C | T | 14 | 0.00160603 | 1.50003E-14 | 59.14595741 |
| rs12920259 | A | G | 16 | 0.00164618 | 4.49997E-09 | 34.38571574 |
| rs1296328 | C | A | 4 | 0.00162094 | 8.80035E-19 | 78.31137333 |
| rs13033310 | A | G | 2 | 0.00185133 | 0.000000012 | 32.5357003 |
| rs13041173 | G | A | 20 | 0.00169514 | 3.40001E-09 | 34.92546849 |
| rs13043475 | G | A | 20 | 0.00205169 | 1.59993E-26 | 113.5544013 |
| rs13044335 | C | A | 20 | 0.00185026 | 2.5E-09 | 35.51548262 |
| rs13097918 | A | T | 3 | 0.00195923 | 5.1E-10 | 38.63120895 |
| rs13107325 | T | C | 4 | 0.00304555 | 1E-21 | 91.67596418 |
| rs1321519 | G | A | 6 | 0.00168255 | 5.89997E-09 | 33.86506032 |
| rs13218383 | G | C | 6 | 0.00169607 | 8.10028E-13 | 51.25664063 |
| rs1327259 | G | A | 6 | 0.00164877 | 2.99999E-10 | 39.69343136 |
| rs13292699 | C | A | 9 | 0.00162014 | 5.10035E-29 | 125.0094424 |
| rs1330199 | T | G | 9 | 0.00161037 | 5.1E-09 | 34.13925638 |
| rs13333747 | C | T | 16 | 0.00208418 | 5.00035E-16 | 65.77832765 |
| rs13389219 | T | C | 2 | 0.00163515 | 3.10027E-13 | 53.12677198 |
| rs13410783 | G | A | 2 | 0.00165646 | 1E-15 | 64.3706739 |
| rs13427822 | G | A | 2 | 0.00181808 | 3.19963E-13 | 53.09516697 |
| rs1360201 | T | C | 9 | 0.00160319 | 2.99999E-09 | 35.21398862 |
| rs1363695 | T | C | 5 | 0.00193202 | 2.59998E-09 | 35.48743297 |
| rs140733155 | G | A | 21 | 0.00775662 | 0.000000015 | 32.01988537 |
| rs1412239 | G | C | 9 | 0.00171146 | 2.19989E-26 | 112.9668429 |
| rs143121872 | C | T | 14 | 0.00582797 | 6.1E-09 | 33.81233813 |
| rs1436348 | G | A | 3 | 0.00162275 | 1E-15 | 64.36928792 |
| rs1441264 | A | G | 13 | 0.0016685 | 9.20026E-16 | 64.58532984 |
| rs1446585 | G | A | 2 | 0.00182273 | 1.20005E-11 | 45.99065093 |
| rs1458156 | T | C | 12 | 0.00160455 | 2.19989E-15 | 62.90188747 |
| rs1477290 | C | T | 5 | 0.00235032 | 6.09958E-30 | 129.1962362 |
| rs148636479 | T | A | 4 | 0.00512718 | 9.09997E-10 | 37.50015037 |
| rs1502317 | T | C | 11 | 0.00179514 | 2.99985E-17 | 71.35418454 |
| rs1554654 | T | C | 3 | 0.00160636 | 8.60003E-15 | 60.19293068 |
| rs1568488 | C | G | 3 | 0.00164328 | 2.49977E-16 | 67.19413956 |
| rs16916303 | G | A | 9 | 0.00249596 | 1.7E-09 | 36.28875441 |
| rs16996657 | C | T | 20 | 0.0024109 | 4.49987E-12 | 47.89772112 |
| rs17024393 | C | T | 1 | 0.00504928 | 3.50026E-23 | 98.33545955 |
| rs17115183 | T | C | 14 | 0.00164132 | 1.7E-10 | 40.77656438 |
| rs17270216 | G | A | 15 | 0.00198766 | 3.69999E-08 | 30.31350995 |
| rs17399739 | G | A | 10 | 0.00316892 | 4.40048E-11 | 43.44023391 |
| rs1743767 | C | A | 6 | 0.00183438 | 2.90001E-09 | 35.24528883 |
| rs17513752 | G | A | 19 | 0.00171058 | 1.50003E-19 | 81.80677865 |
| rs17668356 | G | C | 3 | 0.00226469 | 1.59993E-16 | 67.99554208 |
| rs17744603 | G | C | 17 | 0.00215505 | 4.39997E-09 | 34.42834107 |
| rs1778830 | A | G | 1 | 0.00166747 | 9.8992E-11 | 41.84591706 |
| rs17814208 | G | A | 2 | 0.00186496 | 0.000000008 | 33.27112745 |
| rs1782508 | G | C | 11 | 0.0016844 | 1.80011E-13 | 54.20295322 |
| rs1801282 | G | C | 3 | 0.00246057 | 1.10002E-22 | 96.10544086 |
| rs1814592 | A | G | 12 | 0.00176696 | 1.69981E-11 | 45.28344857 |
| rs1861410 | T | C | 2 | 0.00161349 | 5.40008E-22 | 92.94580867 |
| rs1871329 | G | A | 6 | 0.00189246 | 5.60003E-10 | 38.46718382 |
| rs1889490 | T | C | 10 | 0.00164253 | 2.80001E-09 | 35.30943865 |
| rs1899689 | T | C | 7 | 0.00164239 | 2.99985E-13 | 53.19539569 |
| rs1928496 | T | C | 13 | 0.00183574 | 1E-11 | 46.31906719 |
| rs1945160 | A | G | 18 | 0.00166315 | 9.20005E-10 | 37.48842346 |
| rs197374 | T | C | 1 | 0.0016462 | 5.80003E-10 | 38.39225879 |
| rs2051559 | C | T | 4 | 0.00236659 | 0.000000001 | 37.26931238 |
| rs2052607 | A | G | 18 | 0.00169353 | 3.69999E-14 | 57.34046757 |
| rs2072413 | T | C | 7 | 0.00181266 | 1.40001E-09 | 36.6158985 |
| rs208477 | T | C | 6 | 0.00204684 | 2.19999E-09 | 35.74752752 |
| rs2120450 | A | T | 15 | 0.00229449 | 4.30031E-11 | 43.47527782 |
| rs2133561 | T | A | 5 | 0.00166043 | 4.90004E-13 | 52.25801895 |
| rs2143175 | G | A | 22 | 0.00169662 | 7.59994E-10 | 37.85141011 |
| rs215634 | G | A | 7 | 0.00165007 | 1.20005E-16 | 68.58670828 |
| rs2156675 | A | G | 11 | 0.00192144 | 7.19996E-10 | 37.96951743 |
| rs2162826 | C | A | 5 | 0.00198132 | 4.79999E-09 | 34.28558428 |
| rs2169935 | T | C | 15 | 0.00161099 | 1.89998E-09 | 36.0258521 |
| rs2209514 | A | G | 13 | 0.00217082 | 2.59998E-09 | 35.46733 |
| rs2216931 | A | C | 2 | 0.00169204 | 1.10002E-17 | 73.24969927 |
| rs2283093 | T | C | 7 | 0.00199201 | 0.00000004 | 30.15809998 |
| rs2289379 | T | C | 7 | 0.00164536 | 9.09913E-14 | 55.54931008 |
| rs2291127 | T | C | 3 | 0.00220338 | 1.09999E-09 | 37.09482109 |
| rs2292238 | C | A | 12 | 0.00163292 | 4.19952E-11 | 43.53921126 |
| rs2307111 | C | T | 5 | 0.00163942 | 6.59933E-43 | 188.5424378 |
| rs2425847 | G | A | 20 | 0.00163744 | 2.19999E-08 | 31.3402767 |
| rs2433733 | A | G | 2 | 0.00171086 | 2.70023E-18 | 76.09346412 |
| rs245767 | G | A | 5 | 0.00180641 | 2.80027E-14 | 57.84572484 |
| rs255801 | G | A | 5 | 0.00160735 | 3.69999E-09 | 34.75536565 |
| rs2568958 | A | G | 1 | 0.00163193 | 6.20012E-29 | 124.6175681 |
| rs2606227 | C | T | 3 | 0.00167482 | 0.000000001 | 37.29963704 |
| rs264948 | T | C | 2 | 0.00159947 | 7.90005E-10 | 37.77337272 |
| rs2660241 | C | T | 16 | 0.00166708 | 9.79941E-14 | 55.40738914 |
| rs2678204 | G | T | 1 | 0.00168803 | 5.70033E-26 | 111.0686708 |
| rs2726036 | C | A | 16 | 0.00164054 | 1.50003E-40 | 177.7799451 |
| rs28350 | G | A | 3 | 0.00209348 | 1E-12 | 50.82659862 |
| rs28366156 | C | T | 6 | 0.0023746 | 1.20005E-18 | 77.65375465 |
| rs284532 | G | T | 2 | 0.00160141 | 0.000000032 | 30.56993843 |
| rs28457808 | G | C | 7 | 0.00217393 | 5.30029E-12 | 47.57879224 |
| rs2846139 | T | C | 11 | 0.00194722 | 4.60045E-16 | 65.96754437 |
| rs2861685 | C | T | 2 | 0.00162036 | 6.4998E-15 | 60.75523061 |
| rs2920939 | A | G | 8 | 0.0016396 | 0.000000016 | 31.94531635 |
| rs2954021 | G | A | 8 | 0.00160274 | 6.4998E-13 | 51.6844315 |
| rs2964023 | C | T | 5 | 0.00173694 | 2.19989E-11 | 44.74201503 |
| rs3113509 | T | C | 4 | 0.00180886 | 1.9002E-12 | 49.62564799 |
| rs329118 | T | C | 5 | 0.00162479 | 1.9002E-11 | 45.08820932 |
| rs34234296 | A | G | 2 | 0.00165465 | 5.50047E-14 | 56.54614618 |
| rs34517439 | A | C | 1 | 0.00247484 | 1.80011E-41 | 181.9540284 |
| rs34769775 | T | C | 15 | 0.0017562 | 2.29985E-14 | 58.21684786 |
| rs34801745 | C | G | 3 | 0.00166766 | 1E-11 | 46.23357397 |
| rs35184536 | G | A | 11 | 0.00337637 | 6.29941E-11 | 42.71211709 |
| rs35513882 | C | G | 17 | 0.00223518 | 9.09913E-11 | 42.01495081 |
| rs35519679 | A | G | 10 | 0.00186475 | 2.99999E-09 | 35.20582325 |
| rs35589149 | C | G | 7 | 0.00236615 | 2.69998E-10 | 39.87689109 |
| rs35792595 | A | T | 8 | 0.00176235 | 5.70033E-11 | 42.91519322 |
| rs35867081 | G | A | 17 | 0.00161307 | 3.80014E-13 | 52.74461874 |
| rs36007635 | A | G | 6 | 0.00232453 | 3.40001E-10 | 39.40248526 |
| rs372519 | A | G | 21 | 0.00161655 | 6.4998E-17 | 69.80628084 |
| rs3730071 | A | C | 12 | 0.00468151 | 1.79999E-08 | 31.75127785 |
| rs3737992 | A | G | 1 | 0.00213216 | 7.00003E-15 | 60.60136458 |
| rs3748916 | A | G | 2 | 0.00159801 | 2.99999E-08 | 30.72755859 |
| rs3764002 | T | C | 12 | 0.00182314 | 1E-16 | 68.9632068 |
| rs3764625 | G | T | 19 | 0.00163343 | 0.000000015 | 32.02344696 |
| rs3766823 | A | G | 1 | 0.00211956 | 2.29985E-14 | 58.24035292 |
| rs3803286 | G | A | 14 | 0.00170094 | 4.90004E-18 | 74.93817703 |
| rs383701 | A | G | 1 | 0.00237244 | 2.39999E-08 | 31.12692118 |
| rs396354 | C | T | 2 | 0.00177669 | 1.29999E-10 | 41.33681752 |
| rs40071 | C | T | 5 | 0.00209333 | 1.9002E-17 | 72.23453026 |
| rs4055791 | T | C | 13 | 0.001628 | 2.49977E-14 | 58.06945515 |
| rs41350349 | A | G | 15 | 0.00202914 | 3.69999E-08 | 30.29951412 |
| rs4240326 | G | A | 4 | 0.00160926 | 6.70039E-14 | 56.14984329 |
| rs4266606 | T | C | 8 | 0.0023143 | 2.80001E-08 | 30.86025011 |
| rs4267103 | C | T | 12 | 0.00206137 | 1.40001E-09 | 36.73711135 |
| rs4290163 | T | G | 10 | 0.00164237 | 5.50047E-11 | 42.98051552 |
| rs429343 | G | A | 2 | 0.00162012 | 7.39946E-15 | 60.49062671 |
| rs4307239 | G | A | 7 | 0.00161165 | 3.89996E-08 | 30.20936897 |
| rs4430672 | C | T | 14 | 0.00201238 | 4.20001E-09 | 34.52419406 |
| rs4482463 | A | C | 2 | 0.00300754 | 2.80027E-16 | 66.96040165 |
| rs4561038 | C | T | 1 | 0.00258643 | 0.00000002 | 31.46094229 |
| rs4562625 | G | C | 1 | 0.00164252 | 2.19999E-10 | 40.27456026 |
| rs4672338 | T | C | 2 | 0.00169273 | 0.00000001 | 32.7587217 |
| rs4718964 | T | G | 7 | 0.00163261 | 1.20005E-12 | 50.45844929 |
| rs4757945 | G | A | 11 | 0.00163139 | 5.49997E-09 | 34.00338407 |
| rs4776970 | T | A | 15 | 0.0016714 | 3.19963E-32 | 139.6565657 |
| rs4790292 | A | C | 17 | 0.00223349 | 1.80011E-22 | 95.08758068 |
| rs4808762 | C | T | 19 | 0.00176708 | 9.79941E-23 | 96.32100385 |
| rs4820323 | G | C | 22 | 0.00163093 | 1E-16 | 68.87555783 |
| rs4842920 | T | G | 15 | 0.00179154 | 9.20026E-24 | 100.9982703 |
| rs4876611 | G | A | 8 | 0.00178677 | 9.30037E-22 | 91.85194375 |
| rs4881171 | A | G | 10 | 0.00267945 | 4.09996E-08 | 30.092254 |
| rs4924136 | A | G | 15 | 0.0017252 | 4.20001E-08 | 30.04853388 |
| rs4936175 | C | T | 11 | 0.00161299 | 1.2E-09 | 37.0065233 |
| rs494048 | C | T | 11 | 0.00163692 | 7.49998E-10 | 37.88144862 |
| rs4947584 | T | A | 7 | 0.00168677 | 5.30029E-11 | 43.07474707 |
| rs529200 | G | A | 3 | 0.00160411 | 3.80014E-16 | 66.3524404 |
| rs530255 | C | T | 4 | 0.00162925 | 0.000000032 | 30.5876718 |
| rs55707359 | G | T | 11 | 0.00658334 | 1.2E-09 | 37.01923225 |
| rs56203622 | C | T | 9 | 0.00227126 | 5.00035E-11 | 43.19569389 |
| rs56356382 | C | T | 19 | 0.00204444 | 1.20005E-16 | 68.60082473 |
| rs56399737 | T | C | 13 | 0.00161828 | 2.09991E-11 | 44.89433903 |
| rs577525 | C | T | 10 | 0.0016157 | 2.29985E-20 | 85.53359083 |
| rs57803 | A | G | 6 | 0.00210878 | 3.40001E-08 | 30.47267424 |
| rs58862095 | T | C | 7 | 0.00162795 | 1.9002E-21 | 90.43654244 |
| rs60661769 | A | T | 4 | 0.00167657 | 2.80001E-10 | 39.81341507 |
| rs61023343 | C | A | 5 | 0.0016738 | 1.7E-09 | 36.26647289 |
| rs6103254 | C | T | 20 | 0.00242144 | 5.10035E-11 | 43.13255964 |
| rs61754230 | T | C | 12 | 0.00576291 | 2.5E-09 | 35.55688821 |
| rs61871585 | A | C | 10 | 0.0017484 | 8.40001E-09 | 33.18429464 |
| rs61903695 | G | A | 11 | 0.00184173 | 2.80001E-10 | 39.82352613 |
| rs61969510 | C | T | 13 | 0.00180368 | 1.10002E-11 | 46.16746216 |
| rs62072665 | C | G | 17 | 0.00297292 | 4.49997E-09 | 34.38411771 |
| rs62107261 | C | T | 2 | 0.00373835 | 9.20026E-63 | 279.6021255 |
| rs6444950 | A | G | 3 | 0.00188089 | 5.30029E-11 | 43.07379897 |
| rs6469351 | T | C | 8 | 0.00171004 | 9.09997E-09 | 33.0169875 |
| rs6495017 | A | T | 15 | 0.00188715 | 7.59976E-16 | 64.97702015 |
| rs6551304 | G | A | 3 | 0.00214411 | 1.29987E-13 | 54.88836744 |
| rs6561937 | A | T | 13 | 0.00186739 | 4.70002E-12 | 47.80684338 |
| rs6569648 | T | C | 6 | 0.00187434 | 4.90004E-15 | 61.29317534 |
| rs6575340 | A | G | 14 | 0.00167128 | 1.69981E-20 | 86.16639918 |
| rs6594682 | T | C | 5 | 0.00162687 | 4.00037E-12 | 48.10238048 |
| rs66679256 | T | C | 4 | 0.00161172 | 2.09991E-17 | 72.06637523 |
| rs6669189 | T | C | 1 | 0.00163599 | 5.00035E-11 | 43.17008631 |
| rs66954327 | G | A | 8 | 0.00170672 | 1.5E-09 | 36.51141256 |
| rs6744646 | G | A | 2 | 0.0021185 | 8.49963E-75 | 334.8355816 |
| rs67609008 | C | T | 10 | 0.00178505 | 6.29941E-13 | 51.73711617 |
| rs6861649 | C | T | 5 | 0.00164456 | 9.3994E-12 | 46.45436667 |
| rs6935484 | A | G | 6 | 0.00172646 | 3.09999E-09 | 35.12089509 |
| rs6954290 | T | G | 7 | 0.00171612 | 4.30002E-09 | 34.47931207 |
| rs6973656 | G | A | 7 | 0.00163662 | 5.39995E-09 | 34.05073546 |
| rs698147 | G | A | 5 | 0.00160946 | 2.80001E-10 | 39.78401582 |
| rs7027304 | T | C | 9 | 0.00169169 | 1.69981E-13 | 54.29986445 |
| rs7034554 | G | A | 9 | 0.00165554 | 1.89998E-09 | 36.05728505 |
| rs7038943 | C | T | 9 | 0.00169123 | 3.59998E-10 | 39.29205183 |
| rs7070670 | T | C | 10 | 0.0017178 | 8.9E-09 | 33.07756459 |
| rs7097348 | T | C | 10 | 0.00178343 | 6.49995E-09 | 33.66736273 |
| rs7124681 | A | C | 11 | 0.00162682 | 2.09991E-25 | 108.5204981 |
| rs7132908 | A | G | 12 | 0.00164858 | 4.10015E-44 | 194.0935834 |
| rs71495038 | A | G | 10 | 0.00300748 | 5.79963E-12 | 47.38399781 |
| rs719802 | C | T | 11 | 0.00164528 | 1.50003E-13 | 54.55085844 |
| rs7205054 | G | C | 16 | 0.0020651 | 3.69999E-11 | 43.79259284 |
| rs7218014 | C | T | 17 | 0.00201993 | 1.50003E-22 | 95.43049628 |
| rs7258937 | T | C | 19 | 0.0016076 | 4.90004E-12 | 47.74452385 |
| rs7259070 | C | T | 19 | 0.0016507 | 3.80014E-18 | 75.43838538 |
| rs72634826 | A | G | 1 | 0.00184907 | 6.20012E-16 | 65.36957785 |
| rs72866851 | T | A | 2 | 0.00263785 | 0.00000002 | 31.46257934 |
| rs72913721 | G | A | 2 | 0.0019064 | 1.39991E-12 | 50.11962625 |
| rs73052033 | C | T | 3 | 0.00206397 | 4.79954E-19 | 79.49456769 |
| rs73213484 | T | A | 4 | 0.00229995 | 6.59933E-16 | 65.2477895 |
| rs73985439 | C | A | 2 | 0.00173588 | 1.79999E-10 | 40.63228888 |
| rs7442885 | G | C | 5 | 0.00195574 | 2.39994E-24 | 103.7018156 |
| rs745249 | T | C | 2 | 0.00178249 | 1.99986E-12 | 49.51398326 |
| rs7460093 | A | G | 8 | 0.00161985 | 3.29997E-08 | 30.54089881 |
| rs7537581 | A | C | 1 | 0.00161453 | 5.30029E-12 | 47.58643779 |
| rs754635 | G | C | 3 | 0.00252089 | 4.30031E-11 | 43.47325769 |
| rs75557510 | G | A | 3 | 0.00339045 | 2.39994E-12 | 49.15352153 |
| rs7575523 | G | T | 2 | 0.00163956 | 7.50067E-26 | 110.5424795 |
| rs7619139 | A | T | 3 | 0.00163012 | 9.80009E-10 | 37.37234241 |
| rs76702514 | G | C | 1 | 0.00197286 | 1.79999E-09 | 36.19331866 |
| rs7683836 | A | G | 4 | 0.00161717 | 6.59994E-09 | 33.63479259 |
| rs77149883 | A | G | 2 | 0.00346602 | 9.49992E-09 | 32.93450239 |
| rs7765393 | T | A | 6 | 0.00178423 | 3.19963E-12 | 48.5326041 |
| rs7828631 | T | C | 8 | 0.00256648 | 3.59998E-08 | 30.37983416 |
| rs7893571 | T | G | 10 | 0.00170349 | 3.59998E-12 | 48.34367047 |
| rs7916385 | T | C | 10 | 0.00243913 | 6.09958E-13 | 51.81676547 |
| rs79197301 | A | T | 14 | 0.00291585 | 5.99998E-10 | 38.31138098 |
| rs79246548 | A | T | 12 | 0.00361626 | 4.90004E-08 | 29.74526527 |
| rs7944782 | G | T | 11 | 0.00161115 | 2.39994E-13 | 53.66131957 |
| rs79518326 | A | C | 1 | 0.00485052 | 1.20005E-13 | 55.01431469 |
| rs801738 | G | C | 11 | 0.00167238 | 7.39946E-21 | 87.74535514 |
| rs8020365 | A | T | 14 | 0.00194179 | 4.49987E-31 | 134.3764975 |
| rs803973 | A | G | 16 | 0.00164034 | 5.60003E-09 | 33.95761512 |
| rs8059619 | T | C | 16 | 0.00160778 | 2.09991E-24 | 103.920054 |
| rs8089514 | A | T | 18 | 0.00168254 | 2.80001E-10 | 39.77765089 |
| rs8093356 | T | C | 18 | 0.00191841 | 3.69999E-08 | 30.2766399 |
| rs8096564 | T | G | 18 | 0.00176376 | 1.40001E-09 | 36.69415662 |
| rs8132491 | A | G | 21 | 0.00177756 | 1.80011E-11 | 45.22493645 |
| rs815163 | C | T | 1 | 0.00161067 | 1.50003E-12 | 50.00037937 |
| rs825680 | T | A | 16 | 0.00163671 | 3.50002E-08 | 30.43628404 |
| rs854917 | T | C | 6 | 0.00182431 | 0.000000012 | 32.41214119 |
| rs862320 | T | C | 16 | 0.00163219 | 2.60016E-18 | 76.13997588 |
| rs879620 | T | C | 16 | 0.00165072 | 2.80027E-31 | 135.3150506 |
| rs900448 | G | C | 12 | 0.00173746 | 8.00018E-11 | 42.247082 |
| rs922654 | C | T | 14 | 0.00162147 | 1.89998E-08 | 31.57122091 |
| rs9289630 | C | G | 3 | 0.00164708 | 4.30031E-21 | 88.84974978 |
| rs9294260 | A | G | 6 | 0.00161138 | 4.70002E-13 | 52.30810606 |
| rs9421249 | T | C | 10 | 0.00182832 | 2.19989E-17 | 71.97113903 |
| rs9449999 | G | A | 6 | 0.00163104 | 2.69998E-08 | 30.92087904 |
| rs9480184 | T | C | 6 | 0.0019676 | 1.2E-09 | 36.99849414 |
| rs9522183 | T | G | 13 | 0.00162741 | 4.10015E-15 | 61.66911941 |
| rs9522279 | T | C | 13 | 0.00162746 | 5.30029E-15 | 61.13378479 |
| rs9568867 | A | G | 13 | 0.0024107 | 6.79986E-21 | 87.91230702 |
| rs9571687 | A | C | 13 | 0.00170991 | 2.69998E-09 | 35.36239373 |
| rs9615937 | C | G | 22 | 0.00278069 | 4.60002E-08 | 29.87538084 |
| rs9673839 | G | A | 16 | 0.00161186 | 1.29999E-09 | 36.76623456 |
| rs9788550 | C | G | 14 | 0.00186581 | 9.30037E-22 | 91.85590131 |
| rs9835574 | C | A | 3 | 0.0017907 | 3.29989E-22 | 93.89520173 |
| rs9839081 | A | G | 3 | 0.00173556 | 1.10002E-11 | 46.09664819 |
| rs9843653 | C | T | 3 | 0.00160139 | 1.80011E-41 | 182.0129363 |
| rs9866090 | A | G | 3 | 0.001911 | 5.1E-09 | 34.13775661 |
| rs9917946 | G | T | 4 | 0.0016353 | 3.50026E-11 | 43.84961573 |
| rs9968060 | T | C | 3 | 0.00170069 | 4.79999E-10 | 38.74407155 |

**Table S6**. Genetic instruments used in the analyses for the association of Trunk fat mass with Heart failure.

| SNP | EA | OA | chr | SE | *P* value | F |
| --- | --- | --- | --- | --- | --- | --- |
| rs10050620 | T | C | 5 | 0.00215065 | 2.39999E-09 | 35.58440221 |
| rs10071339 | G | C | 5 | 0.00208636 | 1.7E-09 | 36.27452971 |
| rs10071662 | T | C | 5 | 0.00235657 | 1.89998E-09 | 36.05389507 |
| rs10100245 | A | G | 8 | 0.00203394 | 2.99985E-24 | 103.2053164 |
| rs10116857 | A | C | 9 | 0.0041896 | 1.09999E-09 | 37.07367007 |
| rs1013293 | A | G | 1 | 0.00203614 | 6.70039E-24 | 101.6342121 |
| rs1013402 | G | A | 11 | 0.00215953 | 1.39991E-37 | 164.1924556 |
| rs10141106 | G | A | 14 | 0.00221412 | 1.39991E-11 | 45.69179191 |
| rs10146997 | G | A | 14 | 0.00242675 | 1.59993E-29 | 127.3142698 |
| rs10172196 | A | G | 2 | 0.00218675 | 3.80014E-22 | 93.63300282 |
| rs10187101 | T | C | 2 | 0.00208883 | 2.30001E-10 | 40.20359995 |
| rs1020048 | C | A | 9 | 0.00259232 | 3.69999E-08 | 30.29686421 |
| rs10423928 | A | T | 19 | 0.00254536 | 4.00037E-34 | 148.3324484 |
| rs1049193 | C | G | 12 | 0.00267907 | 0.000000021 | 31.35836124 |
| rs10499014 | G | C | 6 | 0.00228401 | 1.29999E-08 | 32.40541897 |
| rs10510025 | T | C | 10 | 0.00234254 | 2.19989E-13 | 53.78449814 |
| rs10513935 | A | G | 18 | 0.00220101 | 4.49997E-08 | 29.92972468 |
| rs1062557 | A | C | 18 | 0.00230403 | 8.40001E-10 | 37.65514027 |
| rs10745785 | T | C | 12 | 0.0021303 | 2.59998E-09 | 35.45119932 |
| rs10755150 | G | A | 4 | 0.00201982 | 8.9E-09 | 33.06497675 |
| rs10756798 | T | C | 9 | 0.0021025 | 1.29987E-14 | 59.32333167 |
| rs10770294 | A | G | 12 | 0.00237647 | 7.59994E-10 | 37.86344862 |
| rs10788797 | A | G | 1 | 0.00309429 | 5.99998E-10 | 38.32588247 |
| rs10795422 | G | A | 10 | 0.00217967 | 1.39991E-11 | 45.73997973 |
| rs10829612 | T | C | 10 | 0.00212375 | 0.000000012 | 32.53362853 |
| rs10915846 | A | G | 1 | 0.00225759 | 4.79954E-11 | 43.26176851 |
| rs10999456 | T | C | 10 | 0.00228264 | 1.10002E-15 | 64.20540058 |
| rs11023199 | G | A | 11 | 0.00210702 | 2.99999E-09 | 35.20471478 |
| rs11042030 | C | T | 11 | 0.0022565 | 1.10002E-19 | 82.35451903 |
| rs11075263 | T | C | 16 | 0.00249944 | 4.49997E-08 | 29.92730474 |
| rs11105842 | A | G | 12 | 0.00209924 | 2.39994E-11 | 44.64262011 |
| rs111258054 | T | C | 11 | 0.0026373 | 1.29999E-10 | 41.2448084 |
| rs11135450 | G | A | 5 | 0.00214766 | 2.70023E-13 | 53.41231348 |
| rs11165643 | T | C | 1 | 0.00204156 | 5.30029E-24 | 102.1034641 |
| rs1118151 | G | T | 2 | 0.00224748 | 2.19999E-08 | 31.31970754 |
| rs112108364 | G | T | 13 | 0.00225397 | 4.19952E-12 | 48.0106836 |
| rs113603865 | T | C | 1 | 0.00247321 | 2.39994E-12 | 49.15312708 |
| rs113866544 | C | T | 17 | 0.00400267 | 5.10035E-19 | 79.3832487 |
| rs1147346 | G | A | 9 | 0.00210536 | 1.7E-09 | 36.26975908 |
| rs11538 | G | A | 22 | 0.00267423 | 6.69993E-10 | 38.09382606 |
| rs11583602 | A | G | 1 | 0.00203123 | 0.000000012 | 32.548333 |
| rs1159974 | C | T | 6 | 0.00201199 | 8.9002E-12 | 46.54920699 |
| rs11603984 | T | G | 11 | 0.0029501 | 8.9002E-11 | 42.03940582 |
| rs1160543 | T | C | 2 | 0.00204938 | 5.70033E-19 | 79.15718327 |
| rs11619393 | C | T | 13 | 0.00286626 | 1.40001E-08 | 32.16118888 |
| rs11630647 | A | G | 15 | 0.0023231 | 2.19989E-13 | 53.81019146 |
| rs11664848 | G | C | 18 | 0.00213748 | 7.39946E-11 | 42.41364038 |
| rs11709402 | G | A | 3 | 0.00225058 | 5.30029E-20 | 83.86480015 |
| rs117113213 | A | G | 20 | 0.00574091 | 1.79999E-09 | 36.15283453 |
| rs117176448 | G | C | 8 | 0.00341437 | 1.10002E-11 | 46.14240028 |
| rs117342986 | T | C | 16 | 0.00658806 | 3.89996E-09 | 34.66908511 |
| rs117755721 | A | G | 17 | 0.00608795 | 4.60002E-09 | 34.37062637 |
| rs11779446 | G | A | 8 | 0.00274875 | 3.90032E-15 | 61.72542471 |
| rs11782074 | T | G | 8 | 0.00210564 | 7.59976E-13 | 51.38207916 |
| rs11856579 | A | G | 15 | 0.00228124 | 3.2E-09 | 35.08304795 |
| rs12072739 | G | A | 1 | 0.00241174 | 4.90004E-13 | 52.25982905 |
| rs12119170 | T | C | 1 | 0.00203095 | 8.10009E-09 | 33.24075051 |
| rs12144626 | C | T | 1 | 0.00204219 | 6.79986E-14 | 56.11108069 |
| rs1218822 | A | G | 13 | 0.00213351 | 4.40048E-14 | 56.96711426 |
| rs12218858 | T | C | 10 | 0.00203776 | 9.3994E-13 | 50.96834346 |
| rs1229984 | C | T | 4 | 0.0061083 | 4.60045E-11 | 43.32761492 |
| rs12316080 | T | C | 12 | 0.00213132 | 2.39994E-19 | 80.89379082 |
| rs12451882 | T | C | 17 | 0.00217224 | 1.39991E-13 | 54.66550024 |
| rs12459965 | T | C | 19 | 0.00228622 | 4.90004E-13 | 52.24083413 |
| rs12461902 | A | G | 19 | 0.00216399 | 5.19996E-20 | 83.91873612 |
| rs12475388 | A | G | 2 | 0.00202114 | 4.30031E-13 | 52.48028033 |
| rs12613191 | G | A | 2 | 0.00209528 | 2.69998E-08 | 30.91117573 |
| rs12724928 | C | T | 1 | 0.00248818 | 1.69981E-17 | 72.48980753 |
| rs12881629 | G | A | 14 | 0.0036579 | 1.40001E-09 | 36.64751793 |
| rs12921077 | A | G | 16 | 0.00214534 | 0.000000005 | 34.19502442 |
| rs13008033 | G | A | 2 | 0.00258291 | 7.49998E-09 | 33.39622845 |
| rs13084608 | A | G | 3 | 0.00299323 | 1.29987E-13 | 54.80598353 |
| rs13106087 | C | T | 4 | 0.00267798 | 2.49977E-22 | 94.49099078 |
| rs13107325 | T | C | 4 | 0.00382747 | 4.30031E-23 | 97.93990111 |
| rs13156484 | A | G | 5 | 0.00203637 | 1.29987E-11 | 45.77955728 |
| rs13218383 | G | C | 6 | 0.00213163 | 5.90065E-13 | 51.86407902 |
| rs13292699 | C | A | 9 | 0.00203663 | 2.49977E-29 | 126.408018 |
| rs1329733 | G | A | 9 | 0.00202243 | 6.20012E-11 | 42.75742319 |
| rs13333747 | C | T | 16 | 0.0026201 | 4.70002E-18 | 74.98260445 |
| rs13389219 | T | C | 2 | 0.0020557 | 7.89951E-21 | 87.62187584 |
| rs143281807 | C | T | 4 | 0.00481383 | 3.50026E-13 | 52.87965231 |
| rs1436348 | G | A | 3 | 0.00203943 | 8.69961E-11 | 42.08827894 |
| rs1440286 | A | G | 11 | 0.00205095 | 1.79999E-10 | 40.65737278 |
| rs1441264 | A | G | 13 | 0.00209777 | 1.9002E-15 | 63.13527655 |
| rs1446585 | G | A | 2 | 0.00229163 | 1.29987E-11 | 45.77776055 |
| rs1458156 | T | C | 12 | 0.00201656 | 2.80027E-12 | 48.81259275 |
| rs1480474 | G | A | 12 | 0.00204561 | 6.59933E-18 | 74.33678146 |
| rs149457 | T | C | 5 | 0.00268724 | 3.2E-10 | 39.53068386 |
| rs1503526 | C | T | 5 | 0.00201442 | 1.9002E-12 | 49.54540781 |
| rs1506285 | A | G | 3 | 0.00201916 | 1.29987E-11 | 45.75304776 |
| rs1568488 | C | G | 3 | 0.00206529 | 3.90032E-18 | 75.35402371 |
| rs16916303 | G | A | 9 | 0.00313763 | 3.09999E-09 | 35.10200792 |
| rs16996657 | C | T | 20 | 0.00303048 | 1.9002E-11 | 45.04305175 |
| rs17024393 | C | T | 1 | 0.0063465 | 1.80011E-26 | 113.406307 |
| rs17047234 | C | A | 2 | 0.00240808 | 7.8001E-13 | 51.33726547 |
| rs17055384 | T | C | 13 | 0.00261964 | 9.30037E-12 | 46.46375601 |
| rs17055653 | A | C | 5 | 0.0021804 | 1.59993E-14 | 58.9368163 |
| rs17115183 | T | C | 14 | 0.00206336 | 2.09991E-12 | 49.43097997 |
| rs17115481 | A | G | 5 | 0.00227237 | 1.5E-10 | 41.06558316 |
| rs1724557 | A | C | 4 | 0.00205467 | 1.29987E-14 | 59.37203247 |
| rs17411781 | A | T | 7 | 0.00213793 | 4.90004E-17 | 70.38007079 |
| rs17668356 | G | C | 3 | 0.0028463 | 1.10002E-12 | 50.66410644 |
| rs17704028 | T | C | 7 | 0.00284766 | 2.5E-10 | 39.9956328 |
| rs17770336 | T | C | 9 | 0.00215153 | 1.69981E-22 | 95.2446012 |
| rs1782508 | G | C | 11 | 0.00211696 | 2.09991E-12 | 49.41738798 |
| rs1799507 | A | G | 12 | 0.00287436 | 3.79997E-08 | 30.26730024 |
| rs1801282 | G | C | 3 | 0.00309239 | 1.9002E-29 | 126.9659983 |
| rs1808629 | A | G | 8 | 0.00217831 | 9.8992E-26 | 109.9719988 |
| rs1861410 | T | C | 2 | 0.00202848 | 3.69999E-22 | 93.65863209 |
| rs197374 | T | C | 1 | 0.0020687 | 3.59998E-08 | 30.37268905 |
| rs2002023 | T | C | 10 | 0.00205191 | 1.2E-10 | 41.44974802 |
| rs2035831 | C | G | 3 | 0.0021422 | 0.00000002 | 31.52322931 |
| rs2058526 | G | A | 16 | 0.00227035 | 3.40001E-08 | 30.43718718 |
| rs2119263 | T | C | 15 | 0.00242097 | 1.29999E-08 | 32.35439018 |
| rs215634 | G | A | 7 | 0.00207436 | 1.99986E-14 | 58.49407724 |
| rs2164840 | G | A | 2 | 0.00211243 | 5.39995E-09 | 34.02773175 |
| rs2192527 | G | A | 4 | 0.00201901 | 5.40008E-23 | 97.48604885 |
| rs2208947 | G | A | 10 | 0.00228887 | 4.20001E-09 | 34.51813406 |
| rs2216931 | A | C | 2 | 0.00212732 | 5.60015E-15 | 61.03745156 |
| rs2237025 | C | T | 4 | 0.00204108 | 0.000000017 | 31.78257459 |
| rs2261747 | A | G | 20 | 0.00203046 | 7.00003E-18 | 74.22660153 |
| rs2267373 | T | C | 22 | 0.00205044 | 4.30031E-27 | 116.1806089 |
| rs2269487 | G | A | 4 | 0.00296196 | 4.70002E-09 | 34.30365915 |
| rs2276190 | A | G | 18 | 0.00225649 | 1.5E-09 | 36.57856388 |
| rs2289379 | T | C | 7 | 0.00206837 | 0.000000002 | 36.00742652 |
| rs2302209 | T | C | 19 | 0.00222789 | 6.4003E-26 | 110.8423195 |
| rs2307111 | C | T | 5 | 0.00206025 | 2.90001E-36 | 158.1606105 |
| rs2442439 | T | C | 12 | 0.00222042 | 3.40017E-13 | 52.96982968 |
| rs2447832 | T | C | 5 | 0.00202741 | 6.70039E-13 | 51.63031472 |
| rs247008 | G | A | 5 | 0.0021548 | 1.29999E-08 | 32.2963392 |
| rs2482356 | C | T | 9 | 0.00203311 | 3.09999E-09 | 35.13957407 |
| rs2494196 | A | C | 1 | 0.00222319 | 2.80027E-33 | 144.4864144 |
| rs2499468 | A | C | 6 | 0.0021132 | 1.69981E-12 | 49.85902281 |
| rs2606227 | C | T | 3 | 0.00210494 | 2E-10 | 40.43377875 |
| rs2610245 | G | A | 5 | 0.00204845 | 4.09996E-10 | 39.08622581 |
| rs2678204 | G | T | 1 | 0.00212122 | 8.00018E-22 | 92.15717599 |
| rs2737263 | T | G | 8 | 0.00224378 | 1.29987E-26 | 114.0008124 |
| rs2802774 | A | C | 1 | 0.00204991 | 2E-10 | 40.46059876 |
| rs2815752 | A | G | 1 | 0.00205204 | 4.60045E-20 | 84.15767163 |
| rs28366156 | C | T | 6 | 0.00298439 | 1.99986E-18 | 76.73492071 |
| rs28375268 | T | G | 16 | 0.00211517 | 6.59933E-15 | 60.69975857 |
| rs28687152 | C | G | 15 | 0.00228777 | 5.60003E-10 | 38.45206615 |
| rs28714450 | T | C | 8 | 0.00215656 | 1.5E-09 | 36.51845382 |
| rs28742003 | T | C | 15 | 0.00249604 | 1.29987E-28 | 123.1704778 |
| rs2943653 | T | C | 2 | 0.00214165 | 1.99986E-14 | 58.5652112 |
| rs2954021 | G | A | 8 | 0.00201475 | 3.10027E-17 | 71.29485788 |
| rs2966859 | G | A | 16 | 0.00247901 | 1E-10 | 41.80822401 |
| rs2980237 | T | G | 8 | 0.00233626 | 4.39997E-09 | 34.44159416 |
| rs3113509 | T | C | 4 | 0.00227335 | 5.90065E-14 | 56.40235631 |
| rs3114140 | A | G | 4 | 0.00218565 | 2.39994E-14 | 58.19379568 |
| rs314279 | A | C | 6 | 0.00315443 | 1.9002E-19 | 81.36692773 |
| rs33503 | A | G | 3 | 0.0025409 | 8.00018E-14 | 55.80135275 |
| rs34234296 | A | G | 2 | 0.00208027 | 5.00035E-13 | 52.19679944 |
| rs34483452 | A | C | 5 | 0.00295824 | 2.19989E-28 | 122.1028586 |
| rs34517439 | A | C | 1 | 0.00310998 | 2.99985E-47 | 208.4466405 |
| rs34571768 | A | G | 8 | 0.00306499 | 6.90001E-09 | 33.57023917 |
| rs34580448 | C | T | 5 | 0.00507052 | 5.89997E-09 | 33.86683013 |
| rs34675417 | G | A | 17 | 0.00232514 | 4.40048E-14 | 56.99076553 |
| rs35099456 | C | G | 11 | 0.00419884 | 4.79954E-27 | 115.9667037 |
| rs35142762 | C | T | 2 | 0.00265919 | 2.39994E-65 | 291.4314632 |
| rs35307904 | A | G | 9 | 0.00309184 | 1.9002E-14 | 58.67661875 |
| rs35502096 | T | C | 3 | 0.00205164 | 8.60003E-09 | 33.13860729 |
| rs35537311 | T | C | 17 | 0.00207294 | 3.79997E-09 | 34.73805115 |
| rs35697691 | G | C | 15 | 0.00358613 | 5.10035E-13 | 52.15957637 |
| rs35867081 | G | A | 17 | 0.00202779 | 3.69999E-12 | 48.29468247 |
| rs35937770 | A | G | 17 | 0.00214798 | 2.90001E-12 | 48.76070033 |
| rs3729793 | G | C | 7 | 0.00345988 | 8.50002E-09 | 33.1466127 |
| rs3730071 | A | C | 12 | 0.00588375 | 1.20005E-11 | 45.96235049 |
| rs3766823 | A | G | 1 | 0.00266343 | 8.10028E-12 | 46.74369156 |
| rs3803286 | G | A | 14 | 0.00213834 | 2.80027E-13 | 53.34954074 |
| rs3814883 | T | C | 16 | 0.00202247 | 2.29985E-28 | 121.9911387 |
| rs3817428 | G | C | 15 | 0.00228541 | 1.99986E-22 | 94.89408137 |
| rs3822742 | A | C | 5 | 0.0020891 | 4.49987E-15 | 61.44650717 |
| rs3826408 | T | C | 17 | 0.0020234 | 0.000000001 | 37.27007761 |
| rs3845344 | T | C | 1 | 0.0020584 | 2.59998E-09 | 35.45640118 |
| rs394608 | C | T | 21 | 0.00203242 | 1.99986E-15 | 63.03332743 |
| rs396354 | C | T | 2 | 0.00223367 | 2.69998E-08 | 30.89366391 |
| rs4017425 | T | C | 3 | 0.00201802 | 5.90065E-11 | 42.84065171 |
| rs41271299 | T | C | 6 | 0.00456062 | 7.59976E-17 | 69.50012542 |
| rs4253755 | A | G | 22 | 0.00303696 | 6.4003E-12 | 47.20678512 |
| rs429343 | G | A | 2 | 0.00203682 | 1.39991E-12 | 50.13206188 |
| rs4308382 | A | G | 4 | 0.00208017 | 6.59933E-11 | 42.64209226 |
| rs4377779 | C | T | 6 | 0.00211473 | 1E-14 | 59.85775914 |
| rs4398538 | C | T | 4 | 0.00210481 | 7.10068E-11 | 42.48028858 |
| rs441792 | G | A | 2 | 0.00201531 | 1.69981E-14 | 58.88655244 |
| rs4430672 | C | T | 14 | 0.00252996 | 3.59998E-11 | 43.84515138 |
| rs4453725 | T | A | 2 | 0.00203859 | 3.90032E-18 | 75.39042592 |
| rs4482463 | A | C | 2 | 0.00378118 | 1.20005E-15 | 63.99348368 |
| rs4658403 | T | C | 1 | 0.00270075 | 2.30001E-08 | 31.24564734 |
| rs467176 | T | C | 5 | 0.00202565 | 5.39995E-09 | 34.0543245 |
| rs4709745 | C | T | 6 | 0.002183 | 2.29985E-12 | 49.16752728 |
| rs4718964 | T | G | 7 | 0.00205234 | 1.39991E-14 | 59.26660599 |
| rs4722398 | T | C | 7 | 0.00293106 | 6.09958E-12 | 47.30392415 |
| rs4776880 | A | G | 15 | 0.00213938 | 2.80027E-11 | 44.28483248 |
| rs477895 | T | C | 11 | 0.00272044 | 4.30031E-13 | 52.52418414 |
| rs4804312 | C | T | 19 | 0.0020357 | 5.19996E-11 | 43.09748033 |
| rs4898556 | C | A | 14 | 0.00201858 | 4.40048E-11 | 43.44088409 |
| rs4908676 | G | A | 1 | 0.00202268 | 1.7E-10 | 40.82562268 |
| rs4936175 | C | T | 11 | 0.0020272 | 0.000000012 | 32.51058534 |
| rs4942099 | A | G | 13 | 0.00225067 | 0.00000002 | 31.45087318 |
| rs530255 | C | T | 4 | 0.00204744 | 7.10003E-09 | 33.49934555 |
| rs55707359 | G | T | 11 | 0.00827474 | 7.19996E-09 | 33.47535481 |
| rs56186137 | G | A | 16 | 0.0020537 | 7.29962E-60 | 266.306101 |
| rs56374036 | G | A | 20 | 0.00248364 | 1.39991E-11 | 45.72914362 |
| rs577525 | C | T | 10 | 0.00203113 | 1.29987E-19 | 82.06096149 |
| rs578603 | A | T | 8 | 0.00239741 | 5.60003E-09 | 33.97236437 |
| rs584170 | A | G | 6 | 0.00206097 | 3.09999E-08 | 30.63102074 |
| rs587271 | T | C | 1 | 0.00226033 | 4.49997E-09 | 34.37469227 |
| rs58862095 | T | C | 7 | 0.00204646 | 4.40048E-17 | 70.58985216 |
| rs59499656 | T | A | 18 | 0.00213057 | 8.99912E-20 | 82.81267377 |
| rs595127 | C | T | 11 | 0.00203654 | 9.40005E-09 | 32.96428992 |
| rs6058209 | A | G | 20 | 0.00216165 | 1.20005E-14 | 59.61455786 |
| rs6059298 | A | G | 20 | 0.00269721 | 4.20001E-10 | 39.03904178 |
| rs6069037 | A | C | 20 | 0.00227587 | 0.000000016 | 31.91075606 |
| rs60814640 | G | A | 7 | 0.00212567 | 2.60016E-17 | 71.63236082 |
| rs61754230 | T | C | 12 | 0.00724264 | 1.7E-09 | 36.29628938 |
| rs617948 | G | A | 11 | 0.0020171 | 9.09997E-09 | 33.02530978 |
| rs61871585 | A | C | 10 | 0.00219802 | 8.79995E-10 | 37.56818472 |
| rs61903695 | G | A | 11 | 0.00231465 | 1.39991E-11 | 45.73771349 |
| rs62025831 | C | T | 15 | 0.00224762 | 8.10028E-45 | 197.3129825 |
| rs62068776 | C | T | 17 | 0.00224407 | 0.000000012 | 32.5662506 |
| rs62190394 | T | C | 2 | 0.00216112 | 2.09991E-18 | 76.57626663 |
| rs62379271 | G | T | 5 | 0.00204348 | 6.29999E-09 | 33.73609374 |
| rs62425398 | A | C | 6 | 0.00328245 | 1.89998E-08 | 31.58313646 |
| rs62473743 | G | A | 7 | 0.00277226 | 0.000000017 | 31.78378249 |
| rs6429425 | T | G | 1 | 0.00289878 | 0.000000025 | 31.08601603 |
| rs6569648 | T | C | 6 | 0.00235564 | 1.29987E-13 | 54.8899046 |
| rs6575340 | A | G | 14 | 0.00210104 | 5.00035E-18 | 74.89171468 |
| rs6684205 | G | A | 1 | 0.00221981 | 1.50003E-14 | 59.15938974 |
| rs6693294 | G | A | 1 | 0.00217032 | 2.60016E-18 | 76.14056459 |
| rs67180261 | G | A | 1 | 0.00246336 | 7.19996E-09 | 33.49282565 |
| rs6750646 | T | C | 2 | 0.00252025 | 5.60003E-09 | 33.95394403 |
| rs6752378 | A | C | 2 | 0.00201013 | 6.4003E-29 | 124.5572503 |
| rs67689854 | A | C | 16 | 0.00303182 | 2.19989E-14 | 58.30508322 |
| rs6790206 | G | A | 3 | 0.00201099 | 0.000000015 | 32.04627698 |
| rs6799532 | C | T | 3 | 0.00218073 | 1.79999E-09 | 36.12843836 |
| rs6840236 | C | T | 4 | 0.00201942 | 2.09991E-15 | 62.97681761 |
| rs6875133 | C | G | 5 | 0.00239797 | 4.49997E-08 | 29.91361964 |
| rs6934060 | G | A | 6 | 0.00303587 | 8.30004E-09 | 33.2041252 |
| rs6946091 | G | A | 7 | 0.00208766 | 1.5E-09 | 36.55821486 |
| rs6948959 | A | G | 7 | 0.00232556 | 1.20005E-11 | 45.97133413 |
| rs6950442 | G | A | 7 | 0.00251681 | 1E-10 | 41.75063404 |
| rs6973656 | G | A | 7 | 0.00205737 | 9.8992E-11 | 41.83521791 |
| rs6973700 | G | A | 7 | 0.00249242 | 1.29999E-08 | 32.40500915 |
| rs698147 | G | A | 5 | 0.0020226 | 4.09996E-09 | 34.56516751 |
| rs7027304 | T | C | 9 | 0.0021265 | 4.40048E-11 | 43.42044583 |
| rs7038943 | C | T | 9 | 0.00212598 | 1.29987E-11 | 45.78206157 |
| rs704061 | C | T | 12 | 0.00202271 | 6.4998E-19 | 78.90564313 |
| rs7070670 | T | C | 10 | 0.00215958 | 6.29999E-09 | 33.7283425 |
| rs7081254 | C | T | 10 | 0.00249947 | 3.29997E-10 | 39.46467743 |
| rs7124681 | A | C | 11 | 0.00204451 | 3.50026E-32 | 139.456887 |
| rs7132908 | A | G | 12 | 0.00207192 | 8.9002E-46 | 201.6966965 |
| rs7167767 | A | G | 15 | 0.00211541 | 5.19996E-09 | 34.12330166 |
| rs7171864 | A | G | 15 | 0.00213869 | 6.00067E-18 | 74.52779474 |
| rs718947 | G | A | 10 | 0.00215487 | 1.79999E-09 | 36.17886814 |
| rs719802 | C | T | 11 | 0.00206781 | 2.29985E-13 | 53.76189097 |
| rs7218014 | C | T | 17 | 0.00253919 | 1.39991E-30 | 132.1079633 |
| rs724016 | G | A | 3 | 0.00202261 | 3.10027E-42 | 185.4552702 |
| rs7259070 | C | T | 19 | 0.00207499 | 5.30029E-13 | 52.10106975 |
| rs72634814 | A | G | 1 | 0.00220826 | 4.40048E-13 | 52.47202313 |
| rs72755233 | A | G | 15 | 0.00320085 | 6.70039E-24 | 101.6129 |
| rs72767253 | A | C | 5 | 0.00501433 | 4.00037E-12 | 48.10230628 |
| rs72915955 | A | G | 11 | 0.0027579 | 0.000000017 | 31.86133585 |
| rs72976986 | A | G | 19 | 0.00259479 | 2.60016E-12 | 48.98796894 |
| rs73052033 | C | T | 3 | 0.00259403 | 1.29987E-13 | 54.91631406 |
| rs73142879 | T | C | 20 | 0.00257015 | 1.59993E-21 | 90.76655322 |
| rs73175572 | G | A | 3 | 0.00322573 | 9.20026E-11 | 41.98465329 |
| rs73213484 | T | A | 4 | 0.00289049 | 6.00067E-16 | 65.43150901 |
| rs7372674 | A | C | 3 | 0.00209749 | 8.99912E-13 | 51.06079483 |
| rs743572 | G | A | 10 | 0.00207974 | 8.60003E-10 | 37.61872936 |
| rs7442885 | G | C | 5 | 0.00245774 | 6.89922E-16 | 65.16193137 |
| rs7460093 | A | G | 8 | 0.00203628 | 0.00000001 | 32.75930347 |
| rs75281888 | C | T | 5 | 0.00568802 | 5.79963E-12 | 47.40971874 |
| rs75412871 | T | C | 12 | 0.00454484 | 2.39999E-09 | 35.60913217 |
| rs7575523 | G | T | 2 | 0.0020613 | 6.59933E-25 | 106.2070584 |
| rs7582359 | A | G | 2 | 0.00214349 | 7.29995E-09 | 33.45494656 |
| rs7638516 | C | A | 3 | 0.00207593 | 2.19999E-09 | 35.7560135 |
| rs764729 | C | A | 2 | 0.00226217 | 3.69999E-08 | 30.30285508 |
| rs769658 | T | C | 4 | 0.00208637 | 7.70016E-12 | 46.85138299 |
| rs7697556 | C | T | 4 | 0.00201359 | 7.29962E-13 | 51.44992651 |
| rs77165542 | T | C | 2 | 0.00549479 | 6.4003E-47 | 206.9279415 |
| rs77927866 | A | G | 12 | 0.00218666 | 1.29999E-09 | 36.88529408 |
| rs7821664 | G | C | 8 | 0.00227119 | 3.2E-10 | 39.52209769 |
| rs7825990 | A | G | 8 | 0.00205056 | 3.2E-09 | 35.08789755 |
| rs7828631 | T | C | 8 | 0.00322637 | 4.09996E-08 | 30.09461189 |
| rs7916385 | T | C | 10 | 0.00306655 | 2.39994E-12 | 49.16243405 |
| rs79197301 | A | T | 14 | 0.00366567 | 2E-10 | 40.42093843 |
| rs7934638 | A | G | 11 | 0.00235033 | 4.60045E-12 | 47.87001008 |
| rs7944782 | G | T | 11 | 0.00202492 | 1.69981E-13 | 54.27206586 |
| rs79518326 | A | C | 1 | 0.00609592 | 3.69999E-14 | 57.31703466 |
| rs7966251 | A | G | 12 | 0.00231427 | 1.7E-09 | 36.30126674 |
| rs7975187 | G | A | 12 | 0.00245786 | 0.000000001 | 37.31308726 |
| rs8026411 | T | C | 15 | 0.00688025 | 3.40001E-08 | 30.45166124 |
| rs8074454 | C | G | 17 | 0.00215142 | 3.40017E-11 | 43.9438679 |
| rs8096564 | T | G | 18 | 0.00221764 | 4.10015E-12 | 48.07900676 |
| rs812949 | C | T | 5 | 0.00226492 | 2.60016E-16 | 67.05299706 |
| rs8132491 | A | G | 21 | 0.00223484 | 0.000000017 | 31.80254623 |
| rs815341 | G | A | 1 | 0.00209277 | 7.10068E-11 | 42.50254684 |
| rs8180470 | T | C | 5 | 0.00202669 | 8.10009E-10 | 37.74398657 |
| rs8192675 | C | T | 3 | 0.00221609 | 4.40048E-13 | 52.47647769 |
| rs852042 | G | A | 20 | 0.00235691 | 2.39999E-09 | 35.6381001 |
| rs862320 | T | C | 16 | 0.002052 | 8.00018E-19 | 78.50979019 |
| rs879620 | T | C | 16 | 0.00207523 | 2.29985E-31 | 135.7075732 |
| rs922656 | T | C | 14 | 0.00203791 | 2.80001E-08 | 30.83355161 |
| rs9294260 | A | G | 6 | 0.00202514 | 1.99986E-11 | 44.94203656 |
| rs9316661 | C | T | 13 | 0.00253251 | 2.19989E-14 | 58.31485002 |
| rs9322332 | A | C | 6 | 0.00201977 | 2.19989E-11 | 44.8087055 |
| rs9405542 | T | C | 6 | 0.00251396 | 1.40001E-10 | 41.23262704 |
| rs9461887 | T | C | 6 | 0.00224334 | 3.79997E-10 | 39.22439612 |
| rs9515455 | A | G | 13 | 0.00205872 | 5.89997E-10 | 38.3691762 |
| rs9522183 | T | G | 13 | 0.00204608 | 6.70039E-12 | 47.11990985 |
| rs9549263 | T | G | 13 | 0.0022185 | 5.89997E-10 | 38.34783084 |
| rs9568867 | A | G | 13 | 0.00303108 | 2.19989E-23 | 99.2386901 |
| rs9654453 | C | T | 5 | 0.00300471 | 5.40008E-11 | 43.04321727 |
| rs972283 | G | A | 7 | 0.00201287 | 3.59998E-14 | 57.37317206 |
| rs9788550 | C | G | 14 | 0.00234566 | 3.50026E-21 | 89.21802168 |
| rs9814633 | A | G | 3 | 0.00211701 | 5.79963E-14 | 56.43384064 |
| rs982457 | T | C | 7 | 0.00203025 | 3.89996E-08 | 30.18298876 |
| rs9833368 | C | T | 3 | 0.00309759 | 1.40001E-08 | 32.1769659 |
| rs9839081 | A | G | 3 | 0.00218124 | 7.8001E-13 | 51.32878596 |
| rs9843653 | C | T | 3 | 0.00201264 | 2.19989E-30 | 131.2729081 |
| rs9851777 | C | T | 3 | 0.00314687 | 5.10035E-13 | 52.16694567 |
| rs9866090 | A | G | 3 | 0.00240172 | 2.1E-10 | 40.34988528 |
| rs9876664 | T | G | 3 | 0.00208025 | 2.70023E-14 | 57.90915518 |
| rs9948776 | A | G | 18 | 0.00241937 | 2.60016E-11 | 44.4464652 |
| rs9968060 | T | C | 3 | 0.00213746 | 1.59993E-12 | 49.91187891 |

**Table S7**. Genetic instruments used in the analyses for the association of Whole body fat mass with Heart failure.

| SNP | EA | OA | chr | SE | *P* value | F |
| --- | --- | --- | --- | --- | --- | --- |
| rs10050620 | T | C | 5 | 0.00208579 | 3.69999E-09 | 34.77967767 |
| rs10071662 | T | C | 5 | 0.00228563 | 1.2E-09 | 36.92741112 |
| rs1008982 | C | T | 10 | 0.00202513 | 3.69999E-08 | 30.29012677 |
| rs10100245 | A | G | 8 | 0.00197253 | 7.39946E-23 | 96.87354359 |
| rs10116857 | A | C | 9 | 0.00406359 | 9.09997E-10 | 37.50088894 |
| rs10132514 | T | C | 14 | 0.00221021 | 2.30001E-08 | 31.26340893 |
| rs1013402 | G | A | 11 | 0.00209435 | 3.50026E-42 | 185.2372533 |
| rs10141106 | G | A | 14 | 0.00214721 | 2.1E-10 | 40.36105204 |
| rs10146997 | G | A | 14 | 0.00235343 | 8.00018E-30 | 128.6727442 |
| rs10187101 | T | C | 2 | 0.00202539 | 1.50003E-11 | 45.4999835 |
| rs1019315 | T | C | 12 | 0.00207306 | 8.50002E-09 | 33.15412248 |
| rs10206023 | C | G | 2 | 0.00203355 | 0.000000004 | 34.62661697 |
| rs10209821 | T | C | 2 | 0.00205116 | 5.50047E-14 | 56.53703229 |
| rs10248298 | A | C | 7 | 0.00202571 | 1.69981E-14 | 58.8122739 |
| rs1031881 | G | T | 6 | 0.00260229 | 1.09999E-08 | 32.65513165 |
| rs10421787 | T | C | 19 | 0.00235732 | 1.79999E-08 | 31.70974274 |
| rs10423928 | A | T | 19 | 0.00246865 | 1.39991E-37 | 164.120941 |
| rs10478110 | C | A | 5 | 0.00197516 | 1.69981E-11 | 45.24366536 |
| rs10499014 | G | C | 6 | 0.00221512 | 3.89996E-10 | 39.14436686 |
| rs10505836 | C | A | 12 | 0.00283692 | 5.1E-09 | 34.16279952 |
| rs10510025 | T | C | 10 | 0.00227166 | 2.60016E-15 | 62.58111931 |
| rs1062557 | A | C | 18 | 0.00223443 | 2.39999E-10 | 40.11456919 |
| rs10756798 | T | C | 9 | 0.00203905 | 1.9002E-14 | 58.61083067 |
| rs10820852 | A | C | 9 | 0.00218808 | 1.89998E-10 | 40.5293468 |
| rs10878349 | G | A | 12 | 0.00195894 | 6.89922E-14 | 56.10651907 |
| rs10915840 | A | G | 1 | 0.00219669 | 1.80011E-11 | 45.13052445 |
| rs10927006 | C | T | 1 | 0.00278232 | 0.000000012 | 32.43706807 |
| rs10959841 | C | T | 9 | 0.00201081 | 0.00000002 | 31.52265076 |
| rs10999456 | T | C | 10 | 0.00221376 | 9.8992E-13 | 50.86132406 |
| rs11012732 | G | A | 10 | 0.00207656 | 2.99985E-24 | 103.2251146 |
| rs1106761 | A | G | 8 | 0.00203434 | 3.10027E-13 | 53.17034429 |
| rs11075263 | T | C | 16 | 0.00242396 | 4.49997E-08 | 29.90618312 |
| rs11099020 | T | C | 4 | 0.00203762 | 1.20005E-11 | 45.99459978 |
| rs11105842 | A | G | 12 | 0.00203605 | 4.60045E-12 | 47.83022855 |
| rs11135450 | G | A | 5 | 0.00208292 | 1.99986E-12 | 49.51661197 |
| rs11165643 | T | C | 1 | 0.00197995 | 8.60003E-24 | 101.1274308 |
| rs11245344 | T | C | 10 | 0.00197795 | 2E-10 | 40.46978268 |
| rs113079574 | T | C | 4 | 0.00248678 | 2.39999E-08 | 31.10260579 |
| rs113866544 | C | T | 17 | 0.0038815 | 3.50026E-19 | 80.15496576 |
| rs1147346 | G | A | 9 | 0.00204186 | 3.69999E-08 | 30.27839744 |
| rs11538 | G | A | 22 | 0.00259365 | 3.19963E-11 | 44.02697152 |
| rs1159974 | C | T | 6 | 0.00195149 | 1.39991E-11 | 45.6306138 |
| rs11603984 | T | G | 11 | 0.00286103 | 3.80014E-11 | 43.70962212 |
| rs1160543 | T | C | 2 | 0.00198725 | 1.20005E-20 | 86.87206146 |
| rs11656076 | A | G | 17 | 0.00234319 | 6.1E-10 | 38.28152217 |
| rs11656758 | G | A | 17 | 0.00206174 | 3.10027E-12 | 48.63414072 |
| rs11664848 | G | C | 18 | 0.00207285 | 8.80035E-11 | 42.07504822 |
| rs1167309 | T | C | 1 | 0.00209079 | 1.99986E-19 | 81.19287435 |
| rs11698185 | C | T | 20 | 0.00196301 | 2.29985E-16 | 67.31619806 |
| rs11709402 | G | A | 3 | 0.00218265 | 3.29989E-21 | 89.33157563 |
| rs117176448 | G | C | 8 | 0.00331076 | 1E-11 | 46.27094526 |
| rs11731255 | G | A | 4 | 0.00195256 | 1.29999E-08 | 32.39844702 |
| rs117755721 | A | G | 17 | 0.00590379 | 2.69998E-08 | 30.92005634 |
| rs11779446 | G | A | 8 | 0.00266577 | 1E-14 | 59.80799529 |
| rs11782341 | G | A | 8 | 0.00251611 | 5.19996E-09 | 34.0968623 |
| rs11839227 | C | T | 13 | 0.00253518 | 4.40048E-12 | 47.94895203 |
| rs11926024 | A | T | 3 | 0.00221031 | 0.00000002 | 31.49271736 |
| rs12031634 | A | G | 1 | 0.00214264 | 2.90001E-10 | 39.71686711 |
| rs12072739 | G | A | 1 | 0.00233926 | 9.3994E-14 | 55.49430271 |
| rs12107172 | G | A | 3 | 0.00284839 | 4.90004E-08 | 29.77247261 |
| rs12140153 | T | G | 1 | 0.00341853 | 6.4998E-24 | 101.6938022 |
| rs12144626 | C | T | 1 | 0.00198063 | 1.29987E-14 | 59.36660369 |
| rs1218822 | A | G | 13 | 0.00206908 | 5.70033E-14 | 56.46261378 |
| rs12254441 | T | C | 10 | 0.00209269 | 6.70039E-11 | 42.59743974 |
| rs1229984 | C | T | 4 | 0.00592358 | 1.20005E-12 | 50.50229121 |
| rs12316080 | T | C | 12 | 0.00206722 | 5.60015E-15 | 61.03246358 |
| rs12364470 | G | T | 11 | 0.00263466 | 2.70023E-11 | 44.41813248 |
| rs12459965 | T | C | 19 | 0.00221757 | 4.70002E-13 | 52.33205186 |
| rs12462975 | A | G | 19 | 0.00209576 | 1.20005E-21 | 91.3738439 |
| rs12475388 | A | G | 2 | 0.00195977 | 5.00035E-12 | 47.67471355 |
| rs12578258 | G | A | 12 | 0.00214522 | 1.20005E-12 | 50.53874937 |
| rs1263629 | G | A | 2 | 0.00279234 | 1.79999E-08 | 31.73209621 |
| rs12724928 | C | T | 1 | 0.00241337 | 1.50003E-13 | 54.52269673 |
| rs12762744 | T | C | 10 | 0.00226633 | 0.000000012 | 32.46796661 |
| rs12881629 | G | A | 14 | 0.00354724 | 1.79999E-10 | 40.70623026 |
| rs12885458 | G | T | 14 | 0.00195882 | 8.80035E-12 | 46.56845534 |
| rs1296328 | C | A | 4 | 0.00197551 | 2.90001E-17 | 71.42081262 |
| rs12974458 | T | C | 19 | 0.00197346 | 6.59994E-09 | 33.65370889 |
| rs12987931 | T | C | 2 | 0.00201321 | 6.59933E-13 | 51.66053558 |
| rs13008033 | G | A | 2 | 0.00250456 | 5.80003E-09 | 33.89081041 |
| rs13107325 | T | C | 4 | 0.00371154 | 5.19996E-24 | 102.1251192 |
| rs13218383 | G | C | 6 | 0.00206744 | 5.40008E-13 | 52.0584613 |
| rs13292699 | C | A | 9 | 0.00197517 | 1.39991E-29 | 127.5547793 |
| rs13333747 | C | T | 16 | 0.00254086 | 2.09991E-17 | 72.01528229 |
| rs13389219 | T | C | 2 | 0.00199334 | 1.29987E-16 | 68.51807056 |
| rs13408397 | T | C | 2 | 0.00197847 | 1.50003E-15 | 63.60305767 |
| rs13409967 | T | G | 2 | 0.00320833 | 0.000000012 | 32.52916506 |
| rs13427822 | G | A | 2 | 0.00221652 | 1.2E-09 | 36.9776934 |
| rs1350506 | C | T | 4 | 0.0019543 | 5.89997E-10 | 38.35844682 |
| rs1363695 | T | C | 5 | 0.0023545 | 1E-11 | 46.25328615 |
| rs14057 | A | G | 1 | 0.0020398 | 0.00000002 | 31.52009399 |
| rs1436348 | G | A | 3 | 0.00197796 | 3.50026E-13 | 52.89282939 |
| rs1440286 | A | G | 11 | 0.00198918 | 1.29999E-09 | 36.8307265 |
| rs1441264 | A | G | 13 | 0.00203425 | 1.29987E-16 | 68.38844848 |
| rs1446585 | G | A | 2 | 0.0022222 | 2.70023E-11 | 44.37626993 |
| rs1456 | C | T | 8 | 0.00199377 | 2.69998E-09 | 35.41126542 |
| rs1458156 | T | C | 12 | 0.00195592 | 2.29985E-13 | 53.75143591 |
| rs1471093 | A | G | 3 | 0.00201593 | 2.69998E-09 | 35.41204281 |
| rs148636479 | T | A | 4 | 0.00624845 | 5.19996E-09 | 34.12383149 |
| rs1503526 | C | T | 5 | 0.00195362 | 8.80035E-13 | 51.08822738 |
| rs1554654 | T | C | 3 | 0.00195785 | 1.59993E-12 | 49.91775964 |
| rs1568488 | C | G | 3 | 0.002003 | 1.10002E-17 | 73.37466248 |
| rs16916303 | G | A | 9 | 0.00304317 | 3.69999E-09 | 34.76502349 |
| rs16996657 | C | T | 20 | 0.00293913 | 2.70023E-12 | 48.9052083 |
| rs17024393 | C | T | 1 | 0.00615325 | 2.70023E-27 | 117.1305582 |
| rs17115183 | T | C | 14 | 0.00200109 | 3.29989E-12 | 48.49457627 |
| rs17218712 | T | C | 9 | 0.002137 | 0.000000008 | 33.26326009 |
| rs17399739 | G | A | 10 | 0.00386381 | 2.39999E-09 | 35.59318546 |
| rs17668356 | G | C | 3 | 0.00276035 | 5.70033E-15 | 61.00892612 |
| rs17704028 | T | C | 7 | 0.00276169 | 4.60002E-10 | 38.82089204 |
| rs17744603 | G | C | 17 | 0.00262729 | 3.59998E-09 | 34.80321395 |
| rs17770336 | T | C | 9 | 0.00208654 | 2.09991E-24 | 103.9636756 |
| rs17781552 | A | G | 1 | 0.0019837 | 2.59998E-08 | 30.95805785 |
| rs1778830 | A | G | 1 | 0.00203229 | 0.000000012 | 32.48072826 |
| rs1782508 | G | C | 11 | 0.00205311 | 5.79963E-13 | 51.91021737 |
| rs1801282 | G | C | 3 | 0.00299903 | 3.19963E-25 | 107.6832756 |
| rs1813039 | A | G | 8 | 0.00216401 | 1.7E-09 | 36.33092184 |
| rs1840126 | C | A | 2 | 0.00196514 | 0.000000032 | 30.5790754 |
| rs1857883 | A | G | 3 | 0.00196588 | 2.69998E-10 | 39.86745471 |
| rs1861410 | T | C | 2 | 0.00196687 | 5.70033E-23 | 97.39776281 |
| rs1874832 | A | G | 15 | 0.00271039 | 3.50002E-09 | 34.90191756 |
| rs1881505 | C | T | 11 | 0.00424847 | 0.000000021 | 31.40263726 |
| rs1915792 | T | C | 8 | 0.00196086 | 5.49997E-10 | 38.49166979 |
| rs1945160 | A | G | 18 | 0.00202787 | 1.40001E-08 | 32.13591148 |
| rs1964675 | T | C | 3 | 0.00215496 | 1.09999E-09 | 37.11689501 |
| rs197374 | T | C | 1 | 0.00200643 | 6.59994E-10 | 38.12936165 |
| rs2052607 | A | G | 18 | 0.002065 | 1.9002E-17 | 72.28375697 |
| rs2081880 | G | A | 19 | 0.00221726 | 5.49997E-09 | 34.02085016 |
| rs215634 | G | A | 7 | 0.0020116 | 6.89922E-16 | 65.16492719 |
| rs2162826 | C | A | 5 | 0.00241478 | 8.19993E-10 | 37.71918521 |
| rs2192527 | G | A | 4 | 0.00195803 | 1.39991E-21 | 91.06319268 |
| rs2208947 | G | A | 10 | 0.00221984 | 9.80009E-09 | 32.88153255 |
| rs2216931 | A | C | 2 | 0.00206274 | 5.60015E-16 | 65.57078483 |
| rs2237025 | C | T | 4 | 0.00197949 | 2.19999E-09 | 35.77278588 |
| rs2249825 | C | G | 13 | 0.00221203 | 9.20005E-09 | 33.0122333 |
| rs2269487 | G | A | 4 | 0.00287282 | 3.79997E-08 | 30.25379082 |
| rs2271189 | A | G | 12 | 0.00199503 | 4.09996E-09 | 34.58868775 |
| rs2276190 | A | G | 18 | 0.00218811 | 5.60003E-10 | 38.47127682 |
| rs2289379 | T | C | 7 | 0.00200597 | 1.2E-10 | 41.48357344 |
| rs2302209 | T | C | 19 | 0.00216059 | 1.10002E-23 | 100.7093936 |
| rs2307111 | C | T | 5 | 0.00199805 | 2.49977E-40 | 176.7320768 |
| rs2494196 | A | C | 1 | 0.00215631 | 4.70002E-26 | 111.4662623 |
| rs2499468 | A | C | 6 | 0.00204964 | 3.80014E-12 | 48.21371441 |
| rs253440 | T | G | 5 | 0.00292777 | 1.29999E-08 | 32.38732911 |
| rs2568958 | A | G | 1 | 0.001989 | 1E-24 | 105.3280648 |
| rs2606227 | C | T | 3 | 0.0020414 | 5.70033E-11 | 42.90590116 |
| rs2610245 | G | A | 5 | 0.00198674 | 2.30001E-10 | 40.20497234 |
| rs2678204 | G | T | 1 | 0.00205731 | 8.80035E-25 | 105.644956 |
| rs2791643 | T | C | 1 | 0.00228666 | 1.09999E-10 | 41.71282323 |
| rs28350 | G | A | 3 | 0.00255183 | 1.80011E-13 | 54.17093366 |
| rs28366156 | C | T | 6 | 0.00289453 | 4.60045E-19 | 79.57868033 |
| rs28375268 | T | G | 16 | 0.00205102 | 1.69981E-14 | 58.87884626 |
| rs28587941 | C | T | 15 | 0.00233792 | 4.40048E-30 | 129.8460406 |
| rs28687152 | C | G | 15 | 0.0022192 | 2.19999E-10 | 40.27032511 |
| rs2920939 | A | G | 8 | 0.00199878 | 2.19999E-08 | 31.3338487 |
| rs2943653 | T | C | 2 | 0.0020766 | 2.19999E-10 | 40.32305042 |
| rs2954021 | G | A | 8 | 0.00195392 | 4.30031E-16 | 66.1154621 |
| rs2964023 | C | T | 5 | 0.00211678 | 3.10027E-11 | 44.13152316 |
| rs2966859 | G | A | 16 | 0.0024038 | 5.49997E-10 | 38.48200165 |
| rs3113509 | T | C | 4 | 0.00220477 | 1.80011E-13 | 54.2523713 |
| rs314279 | A | C | 6 | 0.00305945 | 5.50047E-15 | 61.08443401 |
| rs329651 | T | G | 11 | 0.00247375 | 2.80001E-08 | 30.87472875 |
| rs33836 | T | C | 19 | 0.0019872 | 9.70063E-18 | 73.56221505 |
| rs34483452 | A | C | 5 | 0.00286872 | 1.29987E-29 | 127.7188669 |
| rs34517439 | A | C | 1 | 0.00301618 | 1.50003E-46 | 205.1858823 |
| rs34571768 | A | G | 8 | 0.00297266 | 4.90004E-08 | 29.7472622 |
| rs35099456 | C | G | 11 | 0.00407213 | 1.69981E-21 | 90.69223433 |
| rs35307904 | A | G | 9 | 0.0029988 | 7.00003E-09 | 33.5285332 |
| rs35537311 | T | C | 17 | 0.0020105 | 3.50002E-09 | 34.8919233 |
| rs35589149 | C | G | 7 | 0.00288457 | 3.90032E-12 | 48.17747922 |
| rs35697691 | G | C | 15 | 0.00347887 | 6.20012E-13 | 51.79752319 |
| rs35722851 | C | T | 7 | 0.00195649 | 2E-10 | 40.51023952 |
| rs35867081 | G | A | 17 | 0.00196663 | 4.00037E-13 | 52.66010874 |
| rs35882248 | T | C | 2 | 0.00209543 | 4.49987E-19 | 79.62655601 |
| rs35937770 | A | G | 17 | 0.00208312 | 8.10009E-09 | 33.26021011 |
| rs3730071 | A | C | 12 | 0.00570662 | 2.49977E-11 | 44.51729418 |
| rs3737992 | A | G | 1 | 0.00259868 | 7.19946E-14 | 56.00840641 |
| rs3739514 | A | G | 9 | 0.00207075 | 0.000000012 | 32.469221 |
| rs3754963 | T | A | 2 | 0.00223515 | 1.2E-09 | 37.03001416 |
| rs3764625 | G | T | 19 | 0.0019914 | 4.70002E-09 | 34.29897986 |
| rs3766823 | A | G | 1 | 0.00258351 | 1.59993E-13 | 54.45980786 |
| rs3772882 | A | C | 3 | 0.00201566 | 6.90001E-09 | 33.55759376 |
| rs3803286 | G | A | 14 | 0.00207372 | 2.90001E-15 | 62.3346845 |
| rs3807566 | T | G | 7 | 0.00197257 | 1.29999E-10 | 41.2691459 |
| rs3814883 | T | C | 16 | 0.00196129 | 1.50003E-30 | 131.9636066 |
| rs3817428 | G | C | 15 | 0.00221707 | 2.39994E-13 | 53.60979139 |
| rs3822742 | A | C | 5 | 0.00202598 | 1.69981E-15 | 63.39931737 |
| rs3826408 | T | C | 17 | 0.00196225 | 1.09999E-09 | 37.08311892 |
| rs394608 | C | T | 21 | 0.00197101 | 8.4004E-16 | 64.78264822 |
| rs396354 | C | T | 2 | 0.00216591 | 6.69993E-10 | 38.10158316 |
| rs40071 | C | T | 5 | 0.00255126 | 7.59976E-15 | 60.43754273 |
| rs4123668 | C | T | 3 | 0.00199148 | 4.20001E-08 | 30.07658945 |
| rs41271299 | T | C | 6 | 0.00442333 | 3.10027E-12 | 48.6142795 |
| rs4240326 | G | A | 4 | 0.00196127 | 8.00018E-17 | 69.4145634 |
| rs429343 | G | A | 2 | 0.00197504 | 1.69981E-14 | 58.9053763 |
| rs4377779 | C | T | 6 | 0.00205102 | 2.90001E-15 | 62.31175764 |
| rs441792 | G | A | 2 | 0.00195402 | 2.60016E-13 | 53.49457893 |
| rs4430672 | C | T | 14 | 0.00245344 | 1.5E-09 | 36.55412467 |
| rs4477562 | T | C | 13 | 0.00294554 | 2.70023E-23 | 98.83959264 |
| rs4482463 | A | C | 2 | 0.00366606 | 3.40017E-16 | 66.57781163 |
| rs4562625 | G | C | 1 | 0.00200188 | 6.29999E-10 | 38.22316357 |
| rs4589116 | T | C | 1 | 0.00286467 | 1.5E-09 | 36.58200231 |
| rs4658403 | T | C | 1 | 0.00261946 | 3.2E-09 | 35.03915389 |
| rs467176 | T | C | 5 | 0.00196444 | 1.09999E-08 | 32.57008676 |
| rs4709745 | C | T | 6 | 0.00211712 | 6.29941E-11 | 42.72672836 |
| rs4718964 | T | G | 7 | 0.0019903 | 1.69981E-13 | 54.29164135 |
| rs477895 | T | C | 11 | 0.00263844 | 7.19946E-13 | 51.50125651 |
| rs4820323 | G | C | 22 | 0.00198862 | 2.29985E-22 | 94.6467486 |
| rs4876611 | G | A | 8 | 0.00217833 | 2.19989E-25 | 108.4100121 |
| rs4908672 | T | C | 1 | 0.00199809 | 0.000000015 | 32.03962597 |
| rs4919478 | G | A | 10 | 0.00212837 | 3.50002E-09 | 34.90996059 |
| rs4936175 | C | T | 11 | 0.00196606 | 1.7E-09 | 36.23561615 |
| rs497905 | T | C | 8 | 0.00235117 | 4.30002E-08 | 30.015897 |
| rs530255 | C | T | 4 | 0.00198568 | 1.29999E-08 | 32.3691131 |
| rs553634 | A | C | 11 | 0.00198315 | 0.000000032 | 30.59919804 |
| rs55707359 | G | T | 11 | 0.00802602 | 4.20001E-10 | 39.03770179 |
| rs56374036 | G | A | 20 | 0.0024087 | 6.4998E-11 | 42.67120901 |
| rs577525 | C | T | 10 | 0.00196979 | 2.90001E-21 | 89.60676934 |
| rs587271 | T | C | 1 | 0.0021922 | 2.39999E-09 | 35.58626993 |
| rs59322078 | T | C | 16 | 0.00349334 | 4.79999E-08 | 29.79867123 |
| rs6008749 | A | T | 22 | 0.00318766 | 3.59998E-09 | 34.81224331 |
| rs6029180 | G | A | 20 | 0.00210805 | 1.5E-09 | 36.56289105 |
| rs6058209 | A | G | 20 | 0.00209635 | 2.80027E-12 | 48.82285222 |
| rs60661769 | A | T | 4 | 0.00204372 | 3.19963E-12 | 48.53910782 |
| rs6069037 | A | C | 20 | 0.00220742 | 6.4E-10 | 38.20720822 |
| rs60814640 | G | A | 7 | 0.00206143 | 5.19996E-14 | 56.64416497 |
| rs61754230 | T | C | 12 | 0.00702405 | 1.09999E-09 | 37.12458542 |
| rs61779305 | C | G | 1 | 0.00266375 | 1.6E-09 | 36.43694432 |
| rs617948 | G | A | 11 | 0.00195633 | 7.79992E-09 | 33.32461891 |
| rs61903695 | G | A | 11 | 0.00224486 | 3.10027E-12 | 48.6232957 |
| rs61969510 | C | T | 13 | 0.00219909 | 4.10015E-12 | 48.06969866 |
| rs62025831 | C | T | 15 | 0.00218025 | 4.00037E-31 | 134.6302396 |
| rs62107261 | C | T | 2 | 0.00455712 | 2.49977E-66 | 295.952757 |
| rs62124717 | A | G | 2 | 0.00334767 | 3.50002E-08 | 30.39084799 |
| rs62302286 | C | T | 4 | 0.00225241 | 5.89997E-10 | 38.35833503 |
| rs62379271 | G | T | 5 | 0.00198188 | 0.000000021 | 31.42769266 |
| rs62407565 | G | A | 6 | 0.00218148 | 8.80035E-11 | 42.06237299 |
| rs62499697 | C | T | 8 | 0.00217517 | 3.59998E-11 | 43.83276743 |
| rs6469351 | T | C | 8 | 0.00208476 | 2.90001E-09 | 35.24956311 |
| rs6561937 | A | T | 13 | 0.00227678 | 3.59998E-09 | 34.81361766 |
| rs6569648 | T | C | 6 | 0.00228474 | 4.70002E-15 | 61.36776267 |
| rs6575340 | A | G | 14 | 0.00203768 | 1.80011E-19 | 81.47842413 |
| rs6669189 | T | C | 1 | 0.00199384 | 3.90032E-11 | 43.68160865 |
| rs6684205 | G | A | 1 | 0.00215289 | 3.69999E-11 | 43.78178068 |
| rs6744646 | G | A | 2 | 0.00258261 | 2.29985E-74 | 332.8189978 |
| rs6752378 | A | C | 2 | 0.00194906 | 4.10015E-35 | 152.8751208 |
| rs67609008 | C | T | 10 | 0.00217618 | 3.50026E-12 | 48.4012284 |
| rs67689854 | A | C | 16 | 0.00294036 | 1.59993E-11 | 45.39676824 |
| rs6790206 | G | A | 3 | 0.0019503 | 9.09997E-09 | 33.02226844 |
| rs6840236 | C | T | 4 | 0.00195841 | 4.49987E-13 | 52.39476762 |
| rs693573 | C | T | 6 | 0.00197039 | 3.09999E-08 | 30.63055849 |
| rs6946091 | G | A | 7 | 0.00202448 | 5.79963E-11 | 42.88733379 |
| rs6973656 | G | A | 7 | 0.00199534 | 2.39999E-10 | 40.13299396 |
| rs6973700 | G | A | 7 | 0.00241705 | 6.29999E-09 | 33.74571417 |
| rs6980476 | G | T | 8 | 0.00219168 | 3.59998E-08 | 30.3502618 |
| rs698147 | G | A | 5 | 0.00196155 | 3.89996E-10 | 39.18438956 |
| rs7027304 | T | C | 9 | 0.00206238 | 8.9002E-12 | 46.56114529 |
| rs7034554 | G | A | 9 | 0.00201834 | 7.00003E-09 | 33.54711891 |
| rs7038943 | C | T | 9 | 0.00206176 | 3.69999E-12 | 48.25847252 |
| rs7070670 | T | C | 10 | 0.00209427 | 5.30005E-09 | 34.09368385 |
| rs7124681 | A | C | 11 | 0.00198294 | 3.29989E-32 | 139.5818784 |
| rs7132908 | A | G | 12 | 0.00200949 | 2.49977E-46 | 204.2323739 |
| rs7167767 | A | G | 15 | 0.00205198 | 8.40001E-10 | 37.6585745 |
| rs7171864 | A | G | 15 | 0.00207457 | 2.60016E-17 | 71.66165158 |
| rs719802 | C | T | 11 | 0.00200551 | 1E-13 | 55.28614413 |
| rs7218014 | C | T | 17 | 0.0024626 | 8.60003E-28 | 119.3866372 |
| rs72634826 | A | G | 1 | 0.00225365 | 2.70023E-14 | 57.9223894 |
| rs72755233 | A | G | 15 | 0.00310531 | 1.20005E-13 | 55.01674123 |
| rs72767253 | A | C | 5 | 0.00486322 | 1E-11 | 46.2637845 |
| rs72915955 | A | G | 11 | 0.00267467 | 0.000000012 | 32.52573138 |
| rs72976986 | A | G | 19 | 0.00251664 | 4.00037E-14 | 57.19326936 |
| rs73052033 | C | T | 3 | 0.00251576 | 4.00037E-17 | 70.78332522 |
| rs73142879 | T | C | 20 | 0.0024928 | 9.20026E-24 | 101.0021775 |
| rs73175572 | G | A | 3 | 0.00312853 | 8.10009E-09 | 33.26001252 |
| rs73213484 | T | A | 4 | 0.00280342 | 1.9002E-16 | 67.74671846 |
| rs73371569 | C | A | 7 | 0.00398289 | 4.09996E-09 | 34.56379142 |
| rs7357754 | G | A | 9 | 0.00196037 | 2.80027E-11 | 44.27909688 |
| rs7372674 | A | C | 3 | 0.00203437 | 5.79963E-13 | 51.92097971 |
| rs73985439 | C | A | 2 | 0.00211613 | 2.19999E-09 | 35.7476427 |
| rs743572 | G | A | 10 | 0.00201698 | 4.30002E-10 | 38.99048863 |
| rs7442885 | G | C | 5 | 0.00238351 | 7.50067E-19 | 78.6163741 |
| rs7460093 | A | G | 8 | 0.00197487 | 6.69993E-09 | 33.63033241 |
| rs74618095 | C | T | 21 | 0.00270269 | 3.89996E-08 | 30.20282653 |
| rs7498665 | G | A | 16 | 0.00199676 | 7.89951E-55 | 243.2091454 |
| rs75281888 | C | T | 5 | 0.00551616 | 4.00037E-12 | 48.13152224 |
| rs7537581 | A | C | 1 | 0.00196786 | 1.79999E-10 | 40.6898326 |
| rs75412871 | T | C | 12 | 0.00440858 | 6.49995E-09 | 33.66430645 |
| rs754635 | G | C | 3 | 0.00307284 | 3.90032E-12 | 48.16942891 |
| rs75524125 | A | T | 12 | 0.00292272 | 2.90001E-10 | 39.73900592 |
| rs7575523 | G | T | 2 | 0.0019987 | 5.30029E-26 | 111.2066116 |
| rs7619139 | A | T | 3 | 0.00198694 | 4.49987E-12 | 47.90921607 |
| rs7632381 | C | T | 3 | 0.00196048 | 9.8992E-31 | 132.8181945 |
| rs7663885 | C | T | 4 | 0.00194867 | 0.000000021 | 31.36889778 |
| rs76702514 | G | C | 1 | 0.00240452 | 1.5E-10 | 40.99073189 |
| rs7683836 | A | G | 4 | 0.00197091 | 4.30002E-08 | 30.00100792 |
| rs770082 | A | G | 12 | 0.00197814 | 5.70033E-18 | 74.61440366 |
| rs7828631 | T | C | 8 | 0.00312884 | 4.20001E-08 | 30.0772057 |
| rs7845090 | A | G | 8 | 0.00216201 | 4.19952E-26 | 111.6980543 |
| rs7864914 | G | A | 9 | 0.00321643 | 1.5E-09 | 36.47732912 |
| rs7893571 | T | G | 10 | 0.00207681 | 1.9002E-12 | 49.55385526 |
| rs7916385 | T | C | 10 | 0.00297359 | 1.59993E-13 | 54.43365821 |
| rs79197301 | A | T | 14 | 0.00355518 | 8.69961E-11 | 42.09703534 |
| rs7934638 | A | G | 11 | 0.00227947 | 1.29987E-11 | 45.75386172 |
| rs7944782 | G | T | 11 | 0.00196395 | 2.19989E-14 | 58.31687745 |
| rs79518326 | A | C | 1 | 0.00591223 | 1.50003E-13 | 54.55591739 |
| rs7960609 | G | A | 12 | 0.00208948 | 5.60003E-09 | 33.96341157 |
| rs7975187 | G | A | 12 | 0.00238412 | 1.2E-10 | 41.4626187 |
| rs79847714 | C | T | 2 | 0.00232419 | 7.69999E-10 | 37.835369 |
| rs8026411 | T | C | 15 | 0.00667383 | 7.79992E-09 | 33.3283584 |
| rs8074454 | C | G | 17 | 0.00208646 | 8.49963E-11 | 42.13736273 |
| rs8076669 | C | T | 17 | 0.00197274 | 1.29999E-08 | 32.31791726 |
| rs8093356 | T | C | 18 | 0.00233922 | 2.1E-10 | 40.35374001 |
| rs8096564 | T | G | 18 | 0.00215052 | 2.29985E-11 | 44.69279244 |
| rs812949 | C | T | 5 | 0.00219661 | 7.8001E-16 | 64.92488301 |
| rs8132491 | A | G | 21 | 0.00216742 | 6.69993E-10 | 38.10260444 |
| rs815163 | C | T | 1 | 0.00196314 | 1.9002E-12 | 49.62325978 |
| rs8192675 | C | T | 3 | 0.00214912 | 8.99912E-14 | 55.57640338 |
| rs825680 | T | A | 16 | 0.00199539 | 0.000000012 | 32.53044467 |
| rs854917 | T | C | 6 | 0.00222371 | 2.59998E-09 | 35.4743819 |
| rs862320 | T | C | 16 | 0.00198982 | 3.59998E-20 | 84.64884043 |
| rs879620 | T | C | 16 | 0.00201244 | 3.69999E-33 | 143.9382309 |
| rs885114 | A | G | 11 | 0.00218994 | 9.70063E-20 | 82.67248132 |
| rs900448 | G | C | 12 | 0.00211789 | 1.09999E-09 | 37.18218798 |
| rs9294260 | A | G | 6 | 0.00196418 | 2.90001E-12 | 48.73620506 |
| rs9304665 | A | T | 19 | 0.00230841 | 9.3994E-15 | 60.01968541 |
| rs9316661 | C | T | 13 | 0.00245572 | 1.80011E-13 | 54.26075448 |
| rs9322332 | A | C | 6 | 0.00195898 | 0.000000004 | 34.60440772 |
| rs9378684 | T | C | 6 | 0.00245891 | 5.30005E-10 | 38.54828638 |
| rs9515455 | A | G | 13 | 0.00199645 | 1.80011E-11 | 45.13252609 |
| rs9522183 | T | G | 13 | 0.00198423 | 4.10015E-14 | 57.11490567 |
| rs9533031 | T | G | 13 | 0.00197792 | 7.49998E-10 | 37.89773334 |
| rs9654453 | C | T | 5 | 0.00291413 | 8E-10 | 37.76174681 |
| rs9673839 | G | A | 16 | 0.00196494 | 0.000000017 | 31.77022226 |
| rs9788550 | C | G | 14 | 0.00227487 | 2.39994E-22 | 94.5644185 |
| rs9839081 | A | G | 3 | 0.00211554 | 7.59976E-12 | 46.86974283 |
| rs9843653 | C | T | 3 | 0.00195189 | 4.30031E-37 | 161.9256217 |
| rs9851777 | C | T | 3 | 0.00305205 | 1.69981E-13 | 54.27388456 |
| rs9876664 | T | G | 3 | 0.00201752 | 6.79986E-16 | 65.18502207 |
| rs9926784 | C | T | 16 | 0.00251742 | 1.5E-09 | 36.52711993 |
| rs9968060 | T | C | 3 | 0.00207288 | 1.59993E-12 | 49.92992351 |

**Table S8**. Genetic instruments used in the analyses for the association of Arm fat-free mass(right) with Heart failure.

| SNP | EA | OA | chr | SE | *P* value | F |
| --- | --- | --- | --- | --- | --- | --- |
| rs10020631 | A | G | 4 | 0.00143536 | 4.70002E-08 | 29.85448106 |
| rs10058393 | T | C | 5 | 0.00184091 | 5.30005E-09 | 34.06998768 |
| rs10062079 | A | G | 5 | 0.00125161 | 0.000000001 | 37.29854967 |
| rs10107388 | C | T | 8 | 0.00128644 | 6.09958E-21 | 88.14915861 |
| rs10119967 | C | A | 9 | 0.00153644 | 5.60015E-19 | 79.20865612 |
| rs10141392 | T | A | 14 | 0.00209349 | 1.40001E-10 | 41.12012792 |
| rs10170082 | C | T | 2 | 0.00183152 | 1.80011E-16 | 67.78584779 |
| rs10222924 | G | A | 4 | 0.00135057 | 1.10002E-12 | 50.60557245 |
| rs1022523 | A | G | 1 | 0.00138435 | 1.39991E-29 | 127.5634283 |
| rs10242866 | T | C | 7 | 0.00126193 | 2.19999E-10 | 40.31006695 |
| rs10248298 | A | C | 7 | 0.00128049 | 1.9002E-14 | 58.63560581 |
| rs10260993 | G | T | 7 | 0.00155878 | 4.79999E-08 | 29.79878976 |
| rs10269774 | A | G | 7 | 0.00131732 | 1.29987E-67 | 301.8870638 |
| rs10283100 | G | A | 8 | 0.00270075 | 2.39994E-20 | 85.43898865 |
| rs1037426 | T | C | 2 | 0.00126645 | 3.79997E-08 | 30.23041693 |
| rs1044977 | C | T | 17 | 0.00142343 | 2.99985E-20 | 84.98605992 |
| rs10453255 | G | A | 9 | 0.00148932 | 1.29999E-10 | 41.23610739 |
| rs1047891 | A | C | 2 | 0.00132354 | 3.40017E-32 | 139.5366512 |
| rs10515237 | G | A | 5 | 0.00137242 | 1.99986E-19 | 81.20368073 |
| rs1055250 | G | C | 11 | 0.00172156 | 3.2E-10 | 39.51944731 |
| rs1064213 | A | G | 2 | 0.00123249 | 1.80011E-23 | 99.65465692 |
| rs10775348 | G | A | 16 | 0.00135768 | 3.40017E-18 | 75.63341521 |
| rs10820852 | A | C | 9 | 0.00138393 | 1.80011E-16 | 67.83351136 |
| rs10832961 | G | C | 11 | 0.00144505 | 7.69999E-10 | 37.83529008 |
| rs10843397 | T | C | 12 | 0.00143975 | 0.000000017 | 31.86007543 |
| rs10861678 | A | G | 12 | 0.00139805 | 1.29987E-12 | 50.26572157 |
| rs10870597 | G | A | 13 | 0.00145908 | 6.70039E-11 | 42.60678262 |
| rs10878984 | T | C | 12 | 0.0013045 | 1.69981E-14 | 58.89224084 |
| rs10920678 | G | A | 1 | 0.00124207 | 1.20005E-11 | 45.89523328 |
| rs10931008 | C | T | 2 | 0.00129928 | 3.29989E-17 | 71.14238513 |
| rs10943915 | T | A | 6 | 0.00135533 | 6.59994E-09 | 33.6496379 |
| rs10986292 | G | T | 9 | 0.00130717 | 9.49948E-16 | 64.53773443 |
| rs11014285 | A | G | 10 | 0.00168465 | 5.70033E-25 | 106.5037722 |
| rs11042725 | A | C | 11 | 0.00123483 | 6.09958E-18 | 74.48133237 |
| rs11049684 | T | C | 12 | 0.00134837 | 3.59998E-14 | 57.39632528 |
| rs11065015 | T | C | 12 | 0.00386771 | 1.79999E-10 | 40.69915524 |
| rs11071182 | G | A | 15 | 0.00185032 | 5.60003E-10 | 38.47228141 |
| rs11097755 | C | T | 4 | 0.00124156 | 0.000000002 | 35.96772527 |
| rs11098675 | G | A | 4 | 0.00155963 | 3.29989E-21 | 89.35457369 |
| rs11109785 | T | A | 12 | 0.00145924 | 2.99999E-08 | 30.71590543 |
| rs111365325 | T | C | 5 | 0.00146591 | 7.50067E-19 | 78.62339902 |
| rs111391498 | G | A | 4 | 0.00289672 | 5.00035E-16 | 65.78274384 |
| rs11142700 | C | T | 9 | 0.00126021 | 1E-10 | 41.79819857 |
| rs11153171 | T | C | 6 | 0.00129201 | 6.79986E-20 | 83.37172195 |
| rs11160601 | T | C | 14 | 0.00215399 | 3.40017E-14 | 57.46875816 |
| rs11217843 | G | A | 11 | 0.00167438 | 1E-10 | 41.74321537 |
| rs11243202 | C | T | 6 | 0.00123801 | 2.49977E-47 | 208.8178022 |
| rs11245450 | A | G | 10 | 0.00126373 | 9.3994E-16 | 64.55269835 |
| rs112544217 | T | C | 2 | 0.00428767 | 3.79997E-08 | 30.23208266 |
| rs112552200 | A | G | 1 | 0.00162117 | 1E-16 | 68.92368189 |
| rs112560164 | A | G | 14 | 0.00157823 | 2.09991E-11 | 44.88795381 |
| rs1135641 | T | G | 14 | 0.00124088 | 3.29997E-09 | 34.9956608 |
| rs113619763 | T | A | 22 | 0.00256405 | 1.09999E-10 | 41.64264531 |
| rs11485595 | T | C | 1 | 0.0012366 | 0.000000025 | 31.0872526 |
| rs115179432 | G | A | 2 | 0.00239598 | 6.79986E-19 | 78.82992314 |
| rs11525873 | C | T | 7 | 0.00208434 | 3.40017E-11 | 43.94052517 |
| rs11590433 | G | A | 1 | 0.00129476 | 9.09913E-12 | 46.51226094 |
| rs11592463 | A | G | 10 | 0.00156137 | 1.89998E-09 | 36.08243632 |
| rs11593630 | T | C | 10 | 0.0012917 | 3.2E-10 | 39.57137544 |
| rs115946508 | A | C | 7 | 0.00197495 | 5.99998E-10 | 38.30707074 |
| rs116165844 | T | G | 20 | 0.0017978 | 1.29999E-08 | 32.28955142 |
| rs11618507 | T | G | 13 | 0.00148278 | 5.40008E-12 | 47.54783199 |
| rs11684531 | G | A | 2 | 0.00181397 | 5.80003E-10 | 38.39480108 |
| rs11695471 | A | T | 2 | 0.00131199 | 3.50026E-14 | 57.4402912 |
| rs11707168 | C | T | 3 | 0.00125205 | 1.09999E-08 | 32.6445598 |
| rs11709402 | G | A | 3 | 0.00138049 | 2.90001E-11 | 44.22550112 |
| rs11717749 | T | C | 3 | 0.00193259 | 2.39994E-14 | 58.15799628 |
| rs117342986 | T | C | 16 | 0.00404225 | 3.09999E-08 | 30.66593546 |
| rs117451679 | G | A | 12 | 0.00203595 | 5.39995E-09 | 34.02620196 |
| rs117543413 | T | C | 10 | 0.00475621 | 2.70023E-12 | 48.88624018 |
| rs1176315 | C | T | 13 | 0.00125094 | 9.90011E-10 | 37.33939363 |
| rs11794152 | G | A | 9 | 0.00125632 | 6.09958E-12 | 47.29102003 |
| rs11838262 | T | C | 12 | 0.00278703 | 2.19999E-09 | 35.76280575 |
| rs11880992 | A | G | 19 | 0.00125994 | 6.4998E-25 | 106.2653115 |
| rs11925245 | G | A | 3 | 0.00161289 | 1.09999E-08 | 32.7245654 |
| rs11997525 | A | T | 8 | 0.00165696 | 5.19996E-19 | 79.3589189 |
| rs12048049 | G | C | 1 | 0.00136008 | 7.19946E-20 | 83.25985522 |
| rs1205593 | C | T | 1 | 0.00144255 | 1.9002E-22 | 95.00624078 |
| rs12095997 | T | C | 1 | 0.00215755 | 1.29987E-18 | 77.49107375 |
| rs12211255 | A | C | 6 | 0.00202965 | 2.39994E-12 | 49.13010867 |
| rs12216497 | T | C | 6 | 0.00124398 | 5.10035E-19 | 79.38434976 |
| rs12255372 | T | G | 10 | 0.00136316 | 0.000000017 | 31.84964321 |
| rs1228024 | A | C | 11 | 0.00130628 | 5.50047E-13 | 52.01668334 |
| rs12344515 | T | C | 9 | 0.00144996 | 3.2E-10 | 39.52854986 |
| rs12354311 | C | A | 1 | 0.00134158 | 4.60002E-08 | 29.89234464 |
| rs12443634 | C | A | 16 | 0.00137972 | 3.69999E-10 | 39.26706582 |
| rs12545613 | T | C | 8 | 0.00181166 | 2.39999E-09 | 35.58154175 |
| rs1260326 | C | T | 2 | 0.00125765 | 9.70063E-48 | 210.6993115 |
| rs12657771 | A | G | 5 | 0.00124946 | 7.70016E-22 | 92.2412152 |
| rs12713004 | G | A | 2 | 0.00138043 | 1.69981E-32 | 140.9398266 |
| rs1271309 | G | A | 12 | 0.00167882 | 1.20005E-25 | 109.6078611 |
| rs12726084 | T | C | 1 | 0.00152606 | 6.4998E-17 | 69.81225227 |
| rs12731454 | G | A | 1 | 0.0013381 | 2.39994E-15 | 62.68080954 |
| rs1281973 | T | C | 6 | 0.00123957 | 2E-10 | 40.50662134 |
| rs12831185 | G | A | 12 | 0.0016451 | 1.59993E-12 | 49.93113416 |
| rs12879423 | G | A | 14 | 0.00133272 | 8.00018E-28 | 119.5283928 |
| rs12879453 | G | A | 14 | 0.00124041 | 1.10002E-11 | 46.2296066 |
| rs12906197 | T | C | 15 | 0.00126103 | 1.80011E-16 | 67.81154126 |
| rs1296328 | C | A | 4 | 0.00124882 | 8.10028E-12 | 46.75012781 |
| rs12980774 | C | T | 19 | 0.00149444 | 2.1E-09 | 35.90851888 |
| rs12995666 | C | T | 2 | 0.00163244 | 2.29985E-83 | 374.1566986 |
| rs13001657 | A | G | 2 | 0.00135398 | 9.8992E-13 | 50.86083764 |
| rs13026283 | T | C | 2 | 0.0012626 | 4.79999E-08 | 29.78880607 |
| rs13037630 | T | C | 20 | 0.00160305 | 1E-29 | 128.1603934 |
| rs13240065 | A | G | 7 | 0.00184388 | 2.09991E-26 | 113.0771125 |
| rs13283416 | G | T | 9 | 0.0012545 | 8.19974E-17 | 69.36785249 |
| rs13392079 | C | T | 2 | 0.00143487 | 1.79999E-09 | 36.15396217 |
| rs13392139 | G | A | 2 | 0.00250184 | 8.40001E-09 | 33.17484466 |
| rs13430869 | T | G | 2 | 0.00140837 | 2.99985E-19 | 80.46371643 |
| rs1351394 | C | T | 12 | 0.00123438 | 9.70063E-74 | 329.9815673 |
| rs1374370 | A | G | 2 | 0.0013379 | 5.10035E-13 | 52.17117077 |
| rs1394046 | T | A | 3 | 0.001434 | 2.19999E-10 | 40.24035499 |
| rs1403987 | G | A | 7 | 0.0012968 | 1.79999E-10 | 40.65832434 |
| rs1411297 | C | A | 20 | 0.00130527 | 5.19996E-14 | 56.64584209 |
| rs1412234 | C | T | 9 | 0.00132014 | 2.29985E-14 | 58.26929658 |
| rs143218848 | G | C | 13 | 0.0016628 | 1.7E-10 | 40.7845232 |
| rs143384 | G | A | 20 | 0.00125974 | 1.7989E-130 | 590.6373053 |
| rs145594985 | T | C | 7 | 0.00178678 | 4.60045E-11 | 43.3375636 |
| rs146851424 | C | A | 13 | 0.00429099 | 6.29941E-33 | 142.8647188 |
| rs1470571 | T | C | 3 | 0.00136822 | 6.59994E-09 | 33.64359484 |
| rs147107835 | T | C | 17 | 0.00985605 | 2.90001E-08 | 30.7475907 |
| rs147110934 | T | G | 19 | 0.00401803 | 7.39946E-13 | 51.43218944 |
| rs1472852 | A | C | 4 | 0.00168695 | 2.29985E-68 | 305.3061982 |
| rs1521624 | A | C | 15 | 0.00124642 | 1.10002E-11 | 46.18114164 |
| rs1528450 | C | T | 2 | 0.00127376 | 3.50002E-10 | 39.39016896 |
| rs1530179 | G | A | 11 | 0.00207434 | 2.1E-09 | 35.85644468 |
| rs1542224 | C | T | 2 | 0.00137184 | 4.00037E-16 | 66.23376035 |
| rs1573891 | C | G | 15 | 0.00170462 | 5.00035E-31 | 134.1942468 |
| rs1576981 | A | G | 13 | 0.00124118 | 4.30002E-08 | 29.99335593 |
| rs1582931 | A | G | 5 | 0.00124651 | 1.10002E-40 | 178.4301505 |
| rs1650465 | A | G | 5 | 0.00124003 | 5.1E-09 | 34.15172032 |
| rs16916881 | A | C | 8 | 0.00145438 | 2.19989E-14 | 58.36473363 |
| rs16942324 | A | C | 15 | 0.00381076 | 1.59993E-20 | 86.2209392 |
| rs16996657 | C | T | 20 | 0.00185838 | 7.59994E-09 | 33.3826746 |
| rs1701827 | G | A | 7 | 0.00140101 | 0.00000002 | 31.45035185 |
| rs17056859 | A | G | 3 | 0.00132602 | 2.59998E-09 | 35.44227595 |
| rs17069647 | A | T | 6 | 0.00211547 | 1.79999E-08 | 31.70756704 |
| rs17089329 | G | C | 8 | 0.00155134 | 3.90032E-13 | 52.67551626 |
| rs17115481 | A | G | 5 | 0.00139309 | 1.29999E-09 | 36.87282374 |
| rs17246129 | A | G | 2 | 0.00133997 | 2.09991E-18 | 76.55156265 |
| rs17277008 | C | T | 1 | 0.00132677 | 8.49963E-26 | 110.2866447 |
| rs173135 | T | C | 17 | 0.00195092 | 2.70023E-16 | 66.98516144 |
| rs17363646 | G | A | 1 | 0.00179543 | 1.59993E-11 | 45.3533152 |
| rs17443541 | C | T | 2 | 0.00158766 | 3.69999E-12 | 48.26628726 |
| rs1744769 | C | T | 20 | 0.00158195 | 4.49987E-13 | 52.42018125 |
| rs17556750 | A | C | 4 | 0.00136269 | 9.20026E-18 | 73.66828871 |
| rs17828687 | A | C | 8 | 0.00125369 | 1.39991E-14 | 59.19983481 |
| rs180963 | C | T | 6 | 0.00123552 | 1.80011E-11 | 45.18054599 |
| rs1813212 | G | A | 11 | 0.00124578 | 0.000000016 | 31.94021738 |
| rs1841738 | G | A | 4 | 0.00124204 | 2.90001E-19 | 80.5345985 |
| rs1889775 | G | T | 13 | 0.00228713 | 2.19999E-08 | 31.30455169 |
| rs1890951 | G | A | 10 | 0.0012792 | 2.30001E-08 | 31.19386664 |
| rs1924929 | G | A | 13 | 0.00148119 | 2.90001E-18 | 75.94450763 |
| rs1927635 | C | T | 9 | 0.00130704 | 2.19989E-12 | 49.29618316 |
| rs1949549 | C | T | 12 | 0.00137581 | 5.39995E-09 | 34.04907967 |
| rs1971955 | G | A | 12 | 0.00175244 | 2.09991E-12 | 49.36673326 |
| rs2005172 | C | A | 17 | 0.00130388 | 1.99986E-54 | 241.329653 |
| rs2024585 | A | G | 6 | 0.00150149 | 3.2E-10 | 39.52278796 |
| rs2101975 | G | A | 4 | 0.0012495 | 1.20005E-27 | 118.7219091 |
| rs2108746 | G | A | 22 | 0.00157094 | 3.50002E-10 | 39.37819346 |
| rs2140046 | C | T | 2 | 0.00128056 | 2.70023E-18 | 76.06995587 |
| rs2197563 | A | G | 2 | 0.00125611 | 8.80035E-15 | 60.15344702 |
| rs2225226 | T | C | 13 | 0.00150752 | 6.09958E-63 | 280.4211863 |
| rs2252720 | T | C | 20 | 0.0013304 | 2.19989E-20 | 85.63621551 |
| rs226250 | T | C | 1 | 0.00125779 | 2.90001E-11 | 44.21262963 |
| rs2265309 | C | T | 10 | 0.00123758 | 1.20005E-22 | 95.87719764 |
| rs2270894 | G | C | 3 | 0.00159127 | 1.20005E-19 | 82.30344804 |
| rs228280 | T | G | 17 | 0.00148092 | 3.2E-09 | 35.0537846 |
| rs2291256 | T | C | 12 | 0.0021836 | 2.19989E-12 | 49.32624191 |
| rs2293176 | A | G | 7 | 0.00130967 | 6.29999E-09 | 33.73948658 |
| rs2295363 | G | A | 1 | 0.00124064 | 4.79954E-15 | 61.35180892 |
| rs2295794 | T | C | 9 | 0.00125851 | 1.7E-09 | 36.26068269 |
| rs2296316 | C | T | 14 | 0.00124995 | 1.69981E-14 | 58.89867138 |
| rs2303083 | A | G | 16 | 0.00156489 | 9.70063E-16 | 64.49253124 |
| rs2307111 | C | T | 5 | 0.00126305 | 6.09958E-38 | 165.7948054 |
| rs2372604 | T | G | 2 | 0.00129408 | 4E-10 | 39.0991176 |
| rs2395792 | G | T | 6 | 0.00306237 | 5.79963E-19 | 79.13798217 |
| rs2437716 | C | T | 16 | 0.00246169 | 4.60002E-09 | 34.35386817 |
| rs244711 | T | C | 5 | 0.00142659 | 6.20012E-28 | 120.0443802 |
| rs252938 | T | C | 5 | 0.00130466 | 1.50003E-12 | 50.10499069 |
| rs2540034 | T | C | 16 | 0.00126834 | 1.69981E-25 | 108.8904114 |
| rs2569993 | C | T | 3 | 0.0013264 | 4.09996E-09 | 34.58236222 |
| rs2650965 | G | A | 20 | 0.00132138 | 1E-10 | 41.74221139 |
| rs271241 | A | G | 5 | 0.00137529 | 1.09999E-08 | 32.57018073 |
| rs2721938 | T | C | 8 | 0.00126387 | 2.90001E-19 | 80.5075513 |
| rs2733287 | C | A | 12 | 0.0012357 | 1.89998E-10 | 40.55227328 |
| rs2789366 | A | G | 1 | 0.0012939 | 6.4003E-11 | 42.68494938 |
| rs281385 | G | A | 19 | 0.00190097 | 1.99986E-11 | 45.00639144 |
| rs2833354 | A | G | 21 | 0.00206188 | 4.60002E-09 | 34.35371629 |
| rs285677 | C | T | 19 | 0.00132174 | 6.89922E-14 | 56.0859045 |
| rs28620532 | G | A | 9 | 0.0013018 | 2.39994E-34 | 149.3716584 |
| rs2866720 | T | C | 7 | 0.00127729 | 2.90001E-09 | 35.22110119 |
| rs2871392 | C | T | 2 | 0.0013151 | 4.09996E-08 | 30.09245341 |
| rs2875907 | G | A | 3 | 0.00129009 | 7.19946E-25 | 106.0384779 |
| rs2885697 | T | G | 1 | 0.00130345 | 6.4003E-44 | 193.2024286 |
| rs2900208 | A | C | 12 | 0.00129272 | 4.40048E-28 | 120.7020217 |
| rs2952615 | C | G | 5 | 0.00127075 | 1.80011E-19 | 81.4361551 |
| rs3099282 | G | A | 6 | 0.00151938 | 1.40001E-09 | 36.66951395 |
| rs310796 | T | G | 12 | 0.00132856 | 1.99986E-14 | 58.48814062 |
| rs3116201 | A | G | 2 | 0.00207514 | 2.99985E-17 | 71.33139591 |
| rs31210 | A | G | 5 | 0.00142256 | 2.39994E-16 | 67.2354809 |
| rs3131115 | T | C | 6 | 0.0012782 | 7.70016E-11 | 42.32672076 |
| rs332115 | A | G | 10 | 0.00138334 | 4.09996E-09 | 34.56624616 |
| rs34017457 | A | G | 16 | 0.00744006 | 7.19996E-10 | 37.97231504 |
| rs34118426 | G | A | 12 | 0.00134255 | 1.20005E-16 | 68.67669176 |
| rs34147411 | T | C | 16 | 0.00176722 | 4.19952E-13 | 52.53177685 |
| rs34373881 | A | G | 3 | 0.0013843 | 4.79999E-08 | 29.78164714 |
| rs343954 | C | T | 2 | 0.00164336 | 3.59998E-12 | 48.3240541 |
| rs34517439 | A | C | 1 | 0.00190608 | 6.29941E-70 | 312.4860453 |
| rs34776209 | T | C | 7 | 0.00143388 | 4.10015E-25 | 107.1693078 |
| rs34879158 | C | A | 20 | 0.00140724 | 1.10002E-43 | 192.1006558 |
| rs35251247 | A | G | 11 | 0.0013683 | 4.60045E-15 | 61.43269279 |
| rs35267052 | G | T | 5 | 0.00203317 | 5.19996E-12 | 47.62104379 |
| rs35309034 | A | C | 8 | 0.00147126 | 2.99985E-15 | 62.23771364 |
| rs35339719 | A | G | 4 | 0.00137599 | 2.30001E-09 | 35.72624716 |
| rs35506085 | A | G | 11 | 0.00160402 | 3.59998E-29 | 125.7023422 |
| rs35631456 | T | C | 17 | 0.00192962 | 1.89998E-10 | 40.56053182 |
| rs357486 | C | T | 3 | 0.00123725 | 4.79954E-15 | 61.34450327 |
| rs35756741 | T | C | 12 | 0.00214388 | 8.99995E-10 | 37.52553934 |
| rs35874463 | G | A | 15 | 0.00265194 | 5.89997E-09 | 33.85620213 |
| rs36000545 | G | A | 17 | 0.00128165 | 1E-31 | 137.3759945 |
| rs36089326 | T | A | 4 | 0.00131463 | 5.99998E-10 | 38.32420196 |
| rs3730071 | A | C | 12 | 0.00360541 | 2.30001E-08 | 31.23928819 |
| rs3740591 | T | C | 10 | 0.00125604 | 2.49977E-18 | 76.24307891 |
| rs3751866 | C | T | 16 | 0.00143626 | 4.39997E-08 | 29.97079541 |
| rs3778934 | C | A | 7 | 0.00130341 | 1.2E-09 | 36.94398678 |
| rs3782232 | A | G | 12 | 0.00240378 | 5.60015E-14 | 56.50519981 |
| rs380803 | C | T | 15 | 0.0013838 | 1.39991E-11 | 45.60211693 |
| rs3809570 | A | C | 15 | 0.00144598 | 1.9002E-21 | 90.48390979 |
| rs3810291 | A | G | 19 | 0.00132054 | 4.40048E-32 | 138.9904117 |
| rs3822742 | A | C | 5 | 0.00128073 | 9.3994E-20 | 82.73977168 |
| rs3843750 | G | C | 19 | 0.00131188 | 1.20005E-20 | 86.7229047 |
| rs3850625 | A | G | 1 | 0.0019057 | 5.50047E-23 | 97.47223206 |
| rs3853252 | A | G | 6 | 0.00124327 | 4.49987E-28 | 120.6627864 |
| rs3879448 | C | G | 1 | 0.00135637 | 3.69999E-10 | 39.28387615 |
| rs3925 | A | G | 8 | 0.00143887 | 0.000000001 | 37.27627494 |
| rs39328 | T | C | 7 | 0.00125156 | 8.69961E-14 | 55.64376095 |
| rs4073153 | G | A | 9 | 0.00125397 | 5.99998E-09 | 33.83179744 |
| rs41268896 | A | G | 6 | 0.00134692 | 3.90032E-36 | 157.5426426 |
| rs41271299 | T | C | 6 | 0.00279284 | 1.39991E-41 | 182.40336 |
| rs41311445 | C | A | 22 | 0.00210864 | 2.80027E-30 | 130.7527349 |
| rs4145824 | A | G | 14 | 0.00129755 | 5.19996E-10 | 38.58271478 |
| rs4240326 | G | A | 4 | 0.0012398 | 1.99986E-55 | 245.9189615 |
| rs4253755 | A | G | 22 | 0.00186451 | 1.09999E-08 | 32.67732116 |
| rs4282339 | A | G | 5 | 0.00151808 | 1.69981E-32 | 140.8576572 |
| rs4341996 | C | A | 20 | 0.00151567 | 1.40001E-08 | 32.22189756 |
| rs434841 | A | G | 6 | 0.001341 | 2.39999E-10 | 40.1213131 |
| rs4477562 | T | C | 13 | 0.00186436 | 1.59993E-17 | 72.56437931 |
| rs4671197 | C | T | 2 | 0.00133295 | 3.50002E-08 | 30.3927277 |
| rs4677151 | A | G | 3 | 0.00124023 | 9.09997E-10 | 37.51168657 |
| rs4725984 | C | T | 7 | 0.0012954 | 1.10002E-41 | 182.8946172 |
| rs4752689 | A | G | 10 | 0.00125335 | 1.50003E-17 | 72.65374195 |
| rs4788218 | C | T | 16 | 0.0012618 | 8.10028E-56 | 247.741812 |
| rs4852257 | G | T | 2 | 0.00125144 | 7.89951E-16 | 64.88625363 |
| rs4858940 | C | T | 3 | 0.00193722 | 5.19996E-16 | 65.70802835 |
| rs4899012 | C | G | 14 | 0.0012669 | 3.10027E-50 | 222.1391545 |
| rs4906203 | T | C | 14 | 0.00146154 | 4.70002E-08 | 29.84158049 |
| rs4922819 | G | A | 11 | 0.00135035 | 4.60045E-11 | 43.35700973 |
| rs4926542 | T | C | 1 | 0.00133097 | 1.20005E-12 | 50.44119005 |
| rs4952818 | T | C | 2 | 0.00123441 | 1.09999E-09 | 37.11813253 |
| rs4963120 | T | C | 11 | 0.00125788 | 4.20001E-08 | 30.04641328 |
| rs4974223 | T | C | 3 | 0.00209117 | 3.90032E-12 | 48.19400557 |
| rs5015933 | C | T | 9 | 0.00124202 | 1E-12 | 50.76123658 |
| rs5019542 | T | C | 6 | 0.00128159 | 0.000000015 | 32.00460975 |
| rs508347 | C | T | 7 | 0.00135186 | 4.90004E-17 | 70.38706805 |
| rs509035 | A | G | 3 | 0.00132636 | 3.29989E-34 | 148.69853 |
| rs55665822 | G | A | 5 | 0.00142181 | 6.89922E-13 | 51.57062367 |
| rs55674305 | A | G | 8 | 0.00134436 | 7.50067E-13 | 51.40035413 |
| rs55745410 | G | A | 1 | 0.0012931 | 5.30029E-28 | 120.3424779 |
| rs55758152 | A | G | 5 | 0.00133204 | 1.9002E-11 | 45.03429222 |
| rs55796651 | T | C | 8 | 0.00141509 | 1.5E-09 | 36.48831346 |
| rs55830103 | G | T | 20 | 0.00163778 | 3.19963E-11 | 44.05993423 |
| rs55831773 | T | C | 17 | 0.00157908 | 3.40017E-15 | 61.99512979 |
| rs56017587 | C | T | 6 | 0.00180286 | 5.30005E-09 | 34.07060902 |
| rs56130943 | C | A | 14 | 0.00150291 | 5.90065E-11 | 42.85622392 |
| rs57020561 | C | T | 6 | 0.00125014 | 1.69981E-12 | 49.78490274 |
| rs57126421 | G | A | 18 | 0.00146358 | 4.79999E-10 | 38.7458147 |
| rs5752989 | A | G | 22 | 0.00125258 | 2.19989E-15 | 62.87249958 |
| rs5753630 | G | A | 22 | 0.00125264 | 3.40001E-08 | 30.49091396 |
| rs5771118 | C | T | 22 | 0.00142417 | 0.000000016 | 31.88616661 |
| rs578366 | G | A | 6 | 0.00124729 | 4.10015E-16 | 66.21209033 |
| rs58164669 | T | A | 12 | 0.00147652 | 1.99986E-13 | 53.97251333 |
| rs58687622 | A | G | 7 | 0.00180101 | 4.60002E-10 | 38.82817512 |
| rs59360013 | T | C | 18 | 0.00309784 | 1.20005E-14 | 59.46079426 |
| rs59985551 | T | C | 2 | 0.00147063 | 1.50003E-20 | 86.32996761 |
| rs603321 | G | A | 6 | 0.00144103 | 8.00018E-19 | 78.49559377 |
| rs60804050 | A | G | 1 | 0.00140916 | 7.59976E-14 | 55.91625485 |
| rs6136938 | A | G | 20 | 0.00124876 | 2.09991E-13 | 53.90454162 |
| rs6142059 | C | T | 20 | 0.00124038 | 6.70039E-14 | 56.14314807 |
| rs61729527 | T | C | 8 | 0.00278814 | 5.10035E-13 | 52.16014845 |
| rs61749613 | G | A | 5 | 0.00309371 | 7.89951E-19 | 78.5347116 |
| rs61849823 | C | T | 10 | 0.00168192 | 5.60015E-11 | 42.96423999 |
| rs61941043 | T | A | 12 | 0.00639667 | 2.19989E-11 | 44.82782784 |
| rs62104476 | A | G | 19 | 0.00131773 | 5.19996E-23 | 97.56833331 |
| rs62136802 | T | A | 19 | 0.00133574 | 2.59998E-09 | 35.43250269 |
| rs62160072 | T | C | 2 | 0.00154631 | 7.69999E-09 | 33.35308808 |
| rs62325220 | C | G | 4 | 0.00173429 | 5.90065E-16 | 65.46297108 |
| rs62372052 | G | A | 5 | 0.0019785 | 1E-37 | 164.7716416 |
| rs62476192 | T | C | 7 | 0.00219811 | 1.80011E-11 | 45.15779346 |
| rs62515438 | G | T | 8 | 0.00147341 | 1.99986E-34 | 149.6840756 |
| rs62621197 | T | C | 19 | 0.00339702 | 6.4998E-16 | 65.26869714 |
| rs62621812 | A | G | 7 | 0.00450698 | 1.80011E-18 | 76.87159627 |
| rs6450346 | C | T | 5 | 0.00134919 | 8.99912E-16 | 64.64293962 |
| rs6500249 | G | A | 16 | 0.00140783 | 3.89996E-10 | 39.17438772 |
| rs6512577 | T | C | 20 | 0.00149517 | 4.20001E-09 | 34.52353321 |
| rs652112 | C | T | 1 | 0.00200601 | 8.00018E-18 | 73.94554455 |
| rs6570509 | T | G | 6 | 0.00136181 | 1.50003E-20 | 86.40215852 |
| rs6575340 | A | G | 14 | 0.00128882 | 4.49987E-11 | 43.40198772 |
| rs6587552 | G | A | 1 | 0.00146035 | 7.00003E-12 | 47.04080247 |
| rs663344 | C | G | 9 | 0.00163122 | 4.49987E-16 | 66.01699476 |
| rs6669139 | T | C | 1 | 0.00151881 | 6.70039E-19 | 78.84232355 |
| rs6690871 | G | A | 1 | 0.00126144 | 9.59997E-10 | 37.40170302 |
| rs67141907 | T | C | 13 | 0.00174325 | 4.30031E-11 | 43.48242092 |
| rs6719296 | A | G | 2 | 0.00123885 | 4.00037E-11 | 43.58994582 |
| rs6721191 | G | A | 2 | 0.00124688 | 7.19946E-11 | 42.46598976 |
| rs6733029 | C | T | 2 | 0.00126304 | 3.59998E-17 | 70.95778734 |
| rs67373773 | A | G | 1 | 0.00124122 | 3.79997E-09 | 34.71197819 |
| rs67551338 | T | C | 12 | 0.00259978 | 1.59993E-15 | 63.5341038 |
| rs676105 | C | T | 11 | 0.0013471 | 6.00067E-19 | 79.06880991 |
| rs6762578 | A | G | 3 | 0.00149134 | 5.10035E-22 | 93.04705409 |
| rs6764533 | A | G | 3 | 0.00128673 | 8.00018E-12 | 46.77748631 |
| rs6800021 | A | G | 3 | 0.00124892 | 8.80035E-32 | 137.631756 |
| rs6824633 | T | C | 4 | 0.00129314 | 1.79999E-08 | 31.65844025 |
| rs6870324 | G | C | 5 | 0.00139797 | 1.89998E-08 | 31.55569165 |
| rs6874142 | G | T | 5 | 0.00205734 | 5.10035E-13 | 52.14879293 |
| rs6898801 | G | A | 5 | 0.0013059 | 1.09999E-10 | 41.55238301 |
| rs6923431 | C | A | 6 | 0.00138255 | 2.59998E-08 | 30.98991179 |
| rs6940643 | G | A | 6 | 0.00126084 | 8.40001E-09 | 33.17953337 |
| rs6951209 | T | A | 7 | 0.0015466 | 1.10002E-11 | 46.18479342 |
| rs7023690 | A | C | 9 | 0.00124795 | 1.09999E-09 | 37.06589443 |
| rs7030157 | C | T | 9 | 0.00138009 | 3.10027E-15 | 62.19139892 |
| rs7033487 | C | T | 9 | 0.00155306 | 1.39991E-41 | 182.4167606 |
| rs7080472 | T | G | 10 | 0.00125292 | 1.80011E-18 | 76.94638436 |
| rs7129320 | A | G | 11 | 0.00166068 | 1.29987E-35 | 155.1874894 |
| rs7132908 | A | G | 12 | 0.00126964 | 6.09958E-31 | 133.7877292 |
| rs7134283 | A | G | 12 | 0.0013742 | 8.19974E-17 | 69.35149218 |
| rs71385734 | G | T | 16 | 0.00165643 | 5.30029E-42 | 184.3947668 |
| rs71647469 | T | C | 1 | 0.00577724 | 3.59998E-10 | 39.30488053 |
| rs7178905 | G | A | 15 | 0.00181784 | 0.000000005 | 34.19120143 |
| rs7204864 | T | C | 16 | 0.00176437 | 2.29985E-19 | 80.97725127 |
| rs7223535 | A | G | 17 | 0.00139246 | 3.40017E-58 | 258.6135915 |
| rs7229351 | A | G | 18 | 0.00128367 | 5.60015E-13 | 51.96701875 |
| rs723149 | G | A | 7 | 0.00124607 | 1.50003E-16 | 68.11974899 |
| rs7237942 | G | A | 18 | 0.00151582 | 3.10027E-60 | 268.0293144 |
| rs724016 | G | A | 3 | 0.00124064 | 5.9979E-103 | 464.1631019 |
| rs7245864 | T | C | 19 | 0.0012436 | 2.99999E-08 | 30.71785334 |
| rs7245985 | G | T | 19 | 0.0015285 | 2.19989E-14 | 58.37619803 |
| rs72656010 | C | T | 8 | 0.00183053 | 8.49963E-69 | 307.2914979 |
| rs72660086 | G | T | 1 | 0.00151184 | 2.49977E-13 | 53.5701142 |
| rs72697614 | A | C | 1 | 0.00133698 | 6.59994E-10 | 38.13208912 |
| rs72703409 | C | G | 4 | 0.00249599 | 2.90001E-08 | 30.79118531 |
| rs72755233 | A | G | 15 | 0.00196436 | 1.40001E-08 | 32.14704578 |
| rs72774845 | C | A | 16 | 0.00133897 | 1E-17 | 73.4516475 |
| rs72828807 | A | G | 17 | 0.00138599 | 2.59998E-08 | 31.01313169 |
| rs72856680 | T | C | 17 | 0.00206294 | 5.19996E-13 | 52.14571314 |
| rs73013411 | A | C | 6 | 0.00182926 | 2.70023E-11 | 44.35216605 |
| rs73052033 | C | T | 3 | 0.00159109 | 2.09991E-19 | 81.09833979 |
| rs7305516 | G | A | 12 | 0.00124296 | 9.3994E-18 | 73.63853505 |
| rs73175572 | G | A | 3 | 0.00197866 | 1.80011E-37 | 163.6570036 |
| rs7321045 | A | G | 13 | 0.00125323 | 2.60016E-12 | 49.00201082 |
| rs73213484 | T | A | 4 | 0.00177304 | 3.50026E-11 | 43.87949088 |
| rs734764 | C | G | 9 | 0.0015195 | 2.69998E-09 | 35.38648635 |
| rs73526129 | A | G | 9 | 0.00396761 | 3.40001E-08 | 30.44144852 |
| rs73601548 | T | C | 10 | 0.00194806 | 1.29987E-12 | 50.27504881 |
| rs73619441 | G | T | 20 | 0.0017652 | 2.99985E-12 | 48.69987182 |
| rs74048171 | A | C | 11 | 0.00141418 | 5.49997E-09 | 33.99962437 |
| rs7442885 | G | C | 5 | 0.00150674 | 7.10068E-28 | 119.7658692 |
| rs74598702 | T | C | 1 | 0.00212866 | 1.69981E-13 | 54.3790711 |
| rs74684058 | T | C | 2 | 0.00241693 | 1.29999E-10 | 41.33128041 |
| rs74841302 | A | G | 15 | 0.00477078 | 4.19952E-15 | 61.60498396 |
| rs74876583 | G | T | 7 | 0.00192329 | 1.80011E-17 | 72.37764759 |
| rs75044513 | C | T | 10 | 0.00206014 | 1.40001E-09 | 36.62337112 |
| rs7513326 | A | G | 1 | 0.00125264 | 0.000000017 | 31.79564741 |
| rs7535501 | T | C | 1 | 0.00268119 | 2.99999E-08 | 30.71601715 |
| rs75406471 | A | G | 10 | 0.00171234 | 2.39994E-15 | 62.72332147 |
| rs7542242 | T | C | 1 | 0.00132709 | 7.00003E-11 | 42.52753043 |
| rs75451531 | T | C | 3 | 0.00174317 | 9.79941E-12 | 46.36213312 |
| rs757833 | A | C | 7 | 0.00131609 | 9.90011E-09 | 32.85096468 |
| rs7581335 | T | A | 2 | 0.0017717 | 4.90004E-10 | 38.72791307 |
| rs758598 | G | A | 17 | 0.00131401 | 4.49987E-30 | 129.8313881 |
| rs76179188 | T | C | 2 | 0.00231019 | 2.19999E-08 | 31.29330644 |
| rs7619139 | A | T | 3 | 0.0012567 | 2.39994E-15 | 62.67191024 |
| rs7633464 | A | G | 3 | 0.00123421 | 5.50047E-15 | 61.05648217 |
| rs76513770 | C | T | 16 | 0.00185036 | 6.20012E-16 | 65.3570831 |
| rs7669124 | A | G | 4 | 0.00128364 | 4.10015E-12 | 48.06912345 |
| rs77079139 | A | G | 13 | 0.00185618 | 1.09999E-08 | 32.59310163 |
| rs77093479 | G | C | 17 | 0.00167959 | 3.50026E-11 | 43.89075319 |
| rs77165542 | T | C | 2 | 0.00336835 | 7.70016E-55 | 243.2418224 |
| rs773145 | C | G | 9 | 0.00125494 | 7.29995E-10 | 37.94952306 |
| rs7740107 | A | T | 6 | 0.00139759 | 6.4998E-87 | 390.4978 |
| rs7755185 | G | A | 6 | 0.00133258 | 2.1E-09 | 35.895347 |
| rs77603146 | A | G | 11 | 0.00197928 | 3.69999E-08 | 30.29258601 |
| rs7766034 | T | C | 6 | 0.00124975 | 1E-10 | 41.81801925 |
| rs7805694 | T | C | 7 | 0.00125638 | 0.000000016 | 31.94438638 |
| rs7824070 | T | C | 8 | 0.00132284 | 2.99985E-11 | 44.15701158 |
| rs78287937 | G | T | 11 | 0.00216113 | 6.4E-10 | 38.19878636 |
| rs78378222 | G | T | 17 | 0.00573729 | 2.49977E-40 | 176.700624 |
| rs7910211 | C | T | 10 | 0.00169333 | 4.90004E-09 | 34.21178772 |
| rs7949333 | T | C | 11 | 0.00124346 | 7.19946E-11 | 42.46249691 |
| rs7952436 | T | C | 11 | 0.00225288 | 2.99985E-46 | 203.8775006 |
| rs7961994 | T | A | 12 | 0.00126448 | 9.49948E-29 | 123.7508447 |
| rs798759 | G | A | 4 | 0.0012344 | 6.4998E-12 | 47.18278706 |
| rs8091287 | C | T | 18 | 0.00146572 | 2.59998E-08 | 31.00415145 |
| rs822551 | G | A | 7 | 0.00159657 | 2.99985E-12 | 48.71726454 |
| rs823118 | T | C | 1 | 0.00123604 | 1.39991E-23 | 100.116535 |
| rs843374 | T | A | 3 | 0.00125373 | 1.29987E-14 | 59.42333691 |
| rs875908 | G | C | 14 | 0.00128953 | 6.90001E-10 | 38.04507502 |
| rs903678 | A | G | 1 | 0.00130125 | 7.00003E-19 | 78.77662322 |
| rs903908 | C | T | 1 | 0.00123739 | 1.69981E-11 | 45.26000409 |
| rs9291926 | G | T | 5 | 0.00123758 | 7.8001E-19 | 78.56177076 |
| rs9327336 | C | T | 5 | 0.00129931 | 1.99986E-11 | 44.99642268 |
| rs9350850 | C | T | 6 | 0.00227754 | 9.30037E-22 | 91.86952401 |
| rs9372414 | T | C | 6 | 0.00129703 | 0.000000032 | 30.58886575 |
| rs9379084 | A | G | 6 | 0.00198614 | 1.50003E-16 | 68.13849394 |
| rs9513510 | C | G | 13 | 0.00134885 | 7.00003E-13 | 51.55311329 |
| rs9538162 | C | T | 13 | 0.00125539 | 2.90001E-10 | 39.75047419 |
| rs960024 | G | T | 1 | 0.0013488 | 1.40001E-08 | 32.24282954 |
| rs9634212 | A | C | 12 | 0.00149457 | 9.20026E-50 | 219.963172 |
| rs9636630 | G | A | 21 | 0.00146608 | 2.39999E-08 | 31.10096494 |
| rs9790159 | G | A | 3 | 0.0012548 | 4.60045E-17 | 70.48343806 |
| rs9800418 | C | T | 5 | 0.00144198 | 0.000000001 | 37.23955024 |
| rs9925273 | G | A | 16 | 0.00160828 | 6.20012E-11 | 42.74678846 |
| rs9951619 | G | T | 18 | 0.00147448 | 9.49948E-13 | 50.9351837 |
| rs9960619 | T | C | 18 | 0.00130344 | 4.90004E-10 | 38.70522977 |

**Table S9**. Genetic instruments used in the analyses for the association of Leg fat-free mass with Heart failure.

| SNP | EA | OA | chr | SE | *P* value | F |
| --- | --- | --- | --- | --- | --- | --- |
| rs10058393 | T | C | 5 | 0.00193822 | 1.09999E-09 | 37.07075912 |
| rs10163018 | T | C | 15 | 0.00134993 | 3.59998E-08 | 30.3399863 |
| rs10202845 | G | A | 2 | 0.00206842 | 2.80027E-15 | 62.42972463 |
| rs10211596 | A | G | 2 | 0.00130221 | 1.69981E-14 | 58.80144762 |
| rs10236214 | T | C | 7 | 0.00136413 | 6.59933E-45 | 197.7110628 |
| rs10269774 | A | G | 7 | 0.00138737 | 1E-64 | 288.5127653 |
| rs10401784 | A | C | 19 | 0.00135943 | 3.29989E-15 | 62.09662126 |
| rs10457469 | A | G | 6 | 0.00129978 | 1.69981E-25 | 108.8763111 |
| rs1047891 | A | C | 2 | 0.00139427 | 2.49977E-31 | 135.5847423 |
| rs1049193 | C | G | 12 | 0.00172933 | 1.59993E-18 | 77.08213091 |
| rs10511111 | C | T | 3 | 0.00140607 | 7.29962E-12 | 46.95388409 |
| rs1064213 | A | G | 2 | 0.00129837 | 4.60045E-18 | 75.04517623 |
| rs10775348 | G | A | 16 | 0.00142976 | 1.9002E-16 | 67.68312023 |
| rs1078043 | G | A | 12 | 0.00244319 | 2.30001E-10 | 40.17575302 |
| rs10780905 | A | G | 9 | 0.0013337 | 4.49997E-10 | 38.88673094 |
| rs10803955 | G | A | 2 | 0.00129545 | 1.10002E-13 | 55.20256569 |
| rs10835372 | C | T | 11 | 0.00134365 | 0.000000016 | 31.96832952 |
| rs10843397 | T | C | 12 | 0.00151653 | 5.49997E-09 | 34.0057409 |
| rs10845408 | T | C | 12 | 0.00136301 | 1E-14 | 59.89059491 |
| rs10858923 | T | A | 12 | 0.00145538 | 1E-10 | 41.81015937 |
| rs10861679 | C | T | 12 | 0.00142419 | 6.20012E-12 | 47.25060121 |
| rs10914462 | G | A | 1 | 0.00131278 | 2.29985E-12 | 49.18520186 |
| rs10920678 | G | A | 1 | 0.00130785 | 9.70063E-17 | 69.02263785 |
| rs10953083 | A | C | 7 | 0.00131394 | 8.50002E-09 | 33.16581399 |
| rs10958683 | G | C | 8 | 0.00154867 | 5.60015E-13 | 51.98865608 |
| rs10975935 | G | A | 9 | 0.00151824 | 2.30001E-09 | 35.6750175 |
| rs10979612 | C | T | 9 | 0.00250179 | 6.4003E-14 | 56.23066551 |
| rs10995366 | A | G | 10 | 0.00150878 | 8.60003E-10 | 37.61785366 |
| rs11014285 | A | G | 10 | 0.00177427 | 1.39991E-23 | 100.1601299 |
| rs11016737 | A | G | 10 | 0.00143066 | 9.49992E-10 | 37.42096234 |
| rs11030112 | A | G | 11 | 0.00139533 | 6.4003E-37 | 161.1482071 |
| rs11042725 | A | C | 11 | 0.0013002 | 1.20005E-14 | 59.49342292 |
| rs11049684 | T | C | 12 | 0.00142028 | 1.2E-09 | 36.95156921 |
| rs11052457 | T | A | 12 | 0.00340008 | 2.90001E-12 | 48.78587478 |
| rs11060406 | T | C | 12 | 0.00351248 | 1.39991E-14 | 59.16819477 |
| rs11065015 | T | C | 12 | 0.00407382 | 3.19963E-13 | 53.07130118 |
| rs1108548 | G | A | 1 | 0.00145302 | 2.09991E-22 | 94.82852658 |
| rs11097755 | C | T | 4 | 0.00130724 | 2.49977E-13 | 53.5345141 |
| rs11130827 | T | A | 3 | 0.00134262 | 2.19989E-11 | 44.75811506 |
| rs111598585 | T | C | 4 | 0.00160394 | 8.99995E-09 | 33.05565315 |
| rs111614550 | A | G | 3 | 0.00136863 | 2.69998E-08 | 30.91252102 |
| rs111640872 | C | G | 19 | 0.00139074 | 1.20005E-28 | 123.2385111 |
| rs11175891 | A | G | 12 | 0.00149575 | 9.79941E-13 | 50.88555723 |
| rs111768603 | T | G | 3 | 0.00207738 | 1.80011E-12 | 49.65480284 |
| rs111821073 | T | C | 9 | 0.00180545 | 1E-19 | 82.60151359 |
| rs11191515 | A | G | 10 | 0.00243215 | 3.19963E-23 | 98.55959013 |
| rs11205354 | A | C | 1 | 0.00131265 | 6.09958E-12 | 47.29612524 |
| rs112069922 | T | C | 4 | 0.00306454 | 2.49977E-17 | 71.66986505 |
| rs11243202 | C | T | 6 | 0.00130443 | 2.80027E-31 | 135.3332137 |
| rs11245450 | A | G | 10 | 0.001331 | 8.69961E-17 | 69.23840333 |
| rs112560164 | A | G | 14 | 0.00166264 | 2.19989E-16 | 67.44781369 |
| rs112685832 | A | C | 1 | 0.00206311 | 9.8992E-13 | 50.87371734 |
| rs112875651 | A | G | 8 | 0.00135067 | 4.70002E-25 | 106.9121122 |
| rs112957890 | G | A | 14 | 0.00149228 | 3.40017E-12 | 48.45961592 |
| rs113684968 | T | C | 2 | 0.00252118 | 4.49997E-09 | 34.38417238 |
| rs114949263 | C | T | 7 | 0.0020714 | 2.39999E-10 | 40.14923717 |
| rs11496125 | T | C | 7 | 0.00132286 | 6.20012E-15 | 60.84934012 |
| rs11516134 | C | T | 9 | 0.00156792 | 2.49977E-12 | 49.07824949 |
| rs115179432 | G | A | 2 | 0.002524 | 5.40008E-13 | 52.05245177 |
| rs11545482 | T | C | 2 | 0.00457771 | 1.89998E-10 | 40.57213361 |
| rs11610148 | C | G | 12 | 0.0018909 | 5.69994E-09 | 33.9300551 |
| rs11616283 | C | T | 13 | 0.00190845 | 9.09997E-09 | 33.02462343 |
| rs11627567 | T | C | 14 | 0.00159111 | 9.49948E-13 | 50.95514283 |
| rs11651809 | G | C | 17 | 0.00143893 | 2.70023E-18 | 76.09453135 |
| rs11695471 | A | T | 2 | 0.00138211 | 2.19989E-11 | 44.75604288 |
| rs11709402 | G | A | 3 | 0.00145362 | 1.80011E-16 | 67.81793638 |
| rs117169657 | T | C | 8 | 0.00190167 | 2.1E-10 | 40.34317545 |
| rs117206167 | T | C | 3 | 0.00263873 | 1.10002E-12 | 50.67574266 |
| rs11738728 | A | G | 5 | 0.00135757 | 5.69994E-09 | 33.92706162 |
| rs117451679 | G | A | 12 | 0.00214456 | 7.59994E-10 | 37.86183241 |
| rs117543413 | T | C | 10 | 0.00500932 | 3.40017E-16 | 66.54341262 |
| rs11761240 | C | T | 7 | 0.00140801 | 1.79999E-08 | 31.70140813 |
| rs1177765 | C | T | 5 | 0.00130199 | 6.70039E-14 | 56.15297891 |
| rs11785562 | A | G | 8 | 0.00164232 | 2.1E-09 | 35.89493283 |
| rs11794152 | G | A | 9 | 0.00132351 | 1.9002E-17 | 72.29322865 |
| rs118173451 | C | T | 22 | 0.0052568 | 4.30002E-08 | 30.01880063 |
| rs118187244 | A | G | 9 | 0.00339351 | 0.000000012 | 32.41644832 |
| rs11855017 | A | C | 15 | 0.00169276 | 4.10015E-13 | 52.59839722 |
| rs1187328 | A | G | 9 | 0.00164756 | 1.2E-09 | 36.92312686 |
| rs11880992 | A | G | 19 | 0.00132735 | 5.10035E-20 | 83.94251044 |
| rs11945720 | A | G | 4 | 0.00178113 | 2.59998E-08 | 30.96998455 |
| rs12041740 | A | G | 1 | 0.0014765 | 6.00067E-25 | 106.4048489 |
| rs12085666 | G | A | 1 | 0.00226852 | 1.10002E-19 | 82.40268449 |
| rs12099669 | A | G | 12 | 0.00141403 | 9.60064E-29 | 123.750146 |
| rs12123505 | G | C | 1 | 0.00144808 | 2.19999E-10 | 40.24809261 |
| rs12140153 | T | G | 1 | 0.00227459 | 5.00035E-17 | 70.31827332 |
| rs12147845 | T | C | 14 | 0.00203921 | 2.90001E-10 | 39.76713112 |
| rs1218822 | A | G | 13 | 0.00137906 | 3.19963E-13 | 53.07490069 |
| rs12238669 | G | A | 9 | 0.00130767 | 7.00003E-15 | 60.60728529 |
| rs12273579 | A | G | 11 | 0.00131847 | 3.79997E-08 | 30.22952139 |
| rs12364470 | G | T | 11 | 0.00175551 | 3.09999E-09 | 35.11847345 |
| rs1243872 | G | T | 9 | 0.0013087 | 8.99912E-12 | 46.54571646 |
| rs12519532 | A | G | 5 | 0.00144251 | 2.39994E-21 | 89.98617066 |
| rs12543207 | T | C | 8 | 0.00153775 | 1.2E-10 | 41.47150606 |
| rs1260326 | C | T | 2 | 0.00132487 | 2.80027E-37 | 162.7859883 |
| rs12655172 | T | C | 5 | 0.00211151 | 3.59998E-16 | 66.43325769 |
| rs1266876 | T | C | 6 | 0.00136168 | 6.89922E-14 | 56.10028482 |
| rs12713004 | G | A | 2 | 0.0014542 | 2.70023E-22 | 94.31906512 |
| rs12731187 | T | C | 1 | 0.00136616 | 1.79999E-08 | 31.69969578 |
| rs12731454 | G | A | 1 | 0.00140901 | 2.80027E-12 | 48.84144968 |
| rs12777893 | G | C | 10 | 0.00190621 | 3.19963E-12 | 48.54957861 |
| rs12879423 | G | A | 14 | 0.00140399 | 9.30037E-26 | 110.0945729 |
| rs12889702 | C | A | 14 | 0.00140853 | 6.89922E-14 | 56.10659085 |
| rs12906197 | T | C | 15 | 0.00132831 | 1.10002E-14 | 59.77808563 |
| rs1296328 | C | A | 4 | 0.00131487 | 1.69981E-13 | 54.35174811 |
| rs13014796 | A | G | 2 | 0.00173771 | 9.20005E-09 | 32.99838707 |
| rs13039956 | C | G | 20 | 0.00168708 | 4.90004E-27 | 115.9399 |
| rs13059004 | C | A | 3 | 0.00131848 | 0.000000017 | 31.86106214 |
| rs13180309 | G | A | 5 | 0.0013132 | 1.80011E-27 | 117.9552802 |
| rs13209685 | T | G | 6 | 0.00177698 | 4.30002E-10 | 38.95406963 |
| rs13211684 | C | T | 6 | 0.00134276 | 7.69999E-09 | 33.34598885 |
| rs13233916 | G | C | 7 | 0.0022812 | 1.7E-10 | 40.72814248 |
| rs13272451 | T | G | 8 | 0.00131337 | 8.60003E-16 | 64.72494504 |
| rs13282247 | T | C | 8 | 0.00142447 | 1.5E-09 | 36.53658353 |
| rs13430869 | T | G | 2 | 0.00148365 | 9.30037E-18 | 73.65090563 |
| rs1360285 | A | G | 9 | 0.00244404 | 1.89998E-09 | 36.0430236 |
| rs1369869 | T | C | 6 | 0.00131511 | 3.10027E-12 | 48.65611718 |
| rs1374370 | A | G | 2 | 0.00140938 | 1.09999E-10 | 41.65394638 |
| rs1376750 | T | C | 2 | 0.00185591 | 8.4004E-13 | 51.17524355 |
| rs139919928 | A | G | 4 | 0.00410685 | 1.79999E-09 | 36.21918648 |
| rs1412234 | C | T | 9 | 0.00139072 | 3.69999E-19 | 80.01443488 |
| rs143384 | G | A | 20 | 0.00132699 | 3.6983E-141 | 639.8136915 |
| rs1443536 | G | A | 4 | 0.00141314 | 4.79954E-15 | 61.32346001 |
| rs1443749 | T | C | 7 | 0.00134863 | 8.99912E-18 | 73.71128306 |
| rs146851424 | C | A | 13 | 0.00451918 | 8.80035E-38 | 165.0726697 |
| rs147448400 | A | C | 6 | 0.00272784 | 3.50026E-28 | 121.1731366 |
| rs1477890 | G | A | 4 | 0.00130176 | 8.69961E-14 | 55.65034384 |
| rs1517037 | T | C | 18 | 0.00166956 | 1.50003E-17 | 72.69095443 |
| rs1542224 | C | T | 2 | 0.00144515 | 7.39946E-22 | 92.30301084 |
| rs1557339 | A | C | 18 | 0.00151911 | 3.50002E-08 | 30.40577467 |
| rs1573891 | C | G | 15 | 0.00179556 | 1.39991E-27 | 118.3720635 |
| rs157577 | C | G | 5 | 0.00145529 | 1.50003E-23 | 99.98488331 |
| rs1657222 | A | G | 10 | 0.00133527 | 4.60045E-11 | 43.34523291 |
| rs16905901 | A | G | 9 | 0.00201716 | 4.90004E-08 | 29.73967784 |
| rs16996637 | T | C | 20 | 0.00196014 | 0.000000016 | 31.9254344 |
| rs17010957 | C | T | 4 | 0.00184397 | 6.20012E-12 | 47.25746955 |
| rs17024393 | C | T | 1 | 0.00409483 | 1.20005E-17 | 73.12966112 |
| rs17115481 | A | G | 5 | 0.0014668 | 6.19998E-10 | 38.24766416 |
| rs17157112 | G | T | 7 | 0.00130863 | 0.000000015 | 32.09431202 |
| rs17246129 | A | G | 2 | 0.00141159 | 1.09999E-10 | 41.59614432 |
| rs17361789 | G | T | 1 | 0.00140124 | 1.80011E-16 | 67.83977637 |
| rs17363646 | G | A | 1 | 0.00189056 | 1.99986E-11 | 44.98962529 |
| rs17491275 | G | T | 1 | 0.00178507 | 1.39991E-25 | 109.3561483 |
| rs17516329 | T | A | 1 | 0.00140754 | 2.30001E-09 | 35.7051121 |
| rs17551974 | A | C | 2 | 0.00166793 | 4.40048E-11 | 43.42763615 |
| rs1813212 | G | A | 11 | 0.00131171 | 8.30042E-13 | 51.20018846 |
| rs183041 | A | G | 5 | 0.00145769 | 3.19963E-11 | 44.04814329 |
| rs185350 | T | C | 19 | 0.00130767 | 8.40001E-09 | 33.1786642 |
| rs1856754 | T | A | 6 | 0.00148662 | 4E-10 | 39.12924863 |
| rs1864180 | G | A | 5 | 0.00130166 | 2.99999E-08 | 30.70976433 |
| rs1889431 | C | T | 10 | 0.00130076 | 1.20005E-11 | 46.02207354 |
| rs1910466 | C | T | 3 | 0.00130687 | 0.000000001 | 37.28315919 |
| rs1923766 | G | T | 1 | 0.00151526 | 2.99999E-08 | 30.73995025 |
| rs1924936 | A | T | 13 | 0.00155987 | 7.59976E-28 | 119.6418346 |
| rs198678 | T | G | 6 | 0.00135584 | 4.30002E-08 | 30.00685562 |
| rs2005172 | C | A | 17 | 0.00137344 | 1.10002E-50 | 224.1641921 |
| rs2007022 | A | C | 20 | 0.00154706 | 1.40001E-08 | 32.23838712 |
| rs2016469 | A | G | 3 | 0.0013542 | 2.30001E-10 | 40.2090096 |
| rs2045767 | G | T | 4 | 0.00132238 | 0.000000017 | 31.85915172 |
| rs2066295 | G | A | 6 | 0.00154476 | 2.49977E-34 | 149.2688042 |
| rs2102278 | G | A | 4 | 0.00139105 | 2.70023E-13 | 53.39012319 |
| rs2122823 | T | C | 7 | 0.00161346 | 2.59998E-09 | 35.4475267 |
| rs2140046 | C | T | 2 | 0.001349 | 8.19974E-14 | 55.76624104 |
| rs2225226 | T | C | 13 | 0.00158758 | 7.8001E-79 | 353.3664892 |
| rs2229840 | T | C | 12 | 0.00177433 | 2.60016E-31 | 135.441043 |
| rs2235699 | A | G | 1 | 0.00147897 | 5.00035E-11 | 43.18255632 |
| rs2237403 | T | C | 7 | 0.00137343 | 5.49997E-09 | 33.99869017 |
| rs224143 | A | G | 10 | 0.0013317 | 2.80027E-12 | 48.82753057 |
| rs2252720 | T | C | 20 | 0.00140141 | 2.80027E-14 | 57.90525701 |
| rs2252909 | T | C | 17 | 0.00139627 | 0.00000001 | 32.81527987 |
| rs2265139 | G | C | 6 | 0.00142889 | 9.8992E-24 | 100.8556321 |
| rs2267371 | G | T | 22 | 0.00138226 | 3.89996E-08 | 30.21586989 |
| rs2270894 | G | C | 3 | 0.00167563 | 1.9002E-26 | 113.3022861 |
| rs2276190 | A | G | 18 | 0.00145834 | 1.6E-09 | 36.37826674 |
| rs2281175 | C | T | 1 | 0.00138968 | 8.19974E-16 | 64.81149058 |
| rs2284932 | A | G | 2 | 0.00132798 | 3.40001E-09 | 34.96627857 |
| rs2287547 | C | T | 12 | 0.00173841 | 6.19998E-09 | 33.76771759 |
| rs2293576 | A | G | 11 | 0.00138197 | 1.50003E-17 | 72.72011761 |
| rs2296316 | C | T | 14 | 0.00131679 | 3.59998E-10 | 39.3117258 |
| rs2298200 | T | C | 1 | 0.00167521 | 1.2E-09 | 36.90199324 |
| rs2307111 | C | T | 5 | 0.00132986 | 2.80027E-38 | 167.3854487 |
| rs2369463 | C | T | 12 | 0.00174392 | 5.80003E-09 | 33.90768855 |
| rs2411453 | G | T | 16 | 0.00132916 | 2.19989E-34 | 149.4822478 |
| rs2455561 | G | T | 15 | 0.0013046 | 3.80014E-16 | 66.33893976 |
| rs2482357 | A | G | 9 | 0.00131324 | 4.60045E-15 | 61.44647849 |
| rs2524137 | T | C | 6 | 0.00141634 | 3.69999E-50 | 221.7856425 |
| rs2568958 | A | G | 1 | 0.00132347 | 1E-14 | 59.89639759 |
| rs2569993 | C | T | 3 | 0.00139669 | 2.90001E-11 | 44.24279957 |
| rs2615075 | G | A | 1 | 0.00134376 | 6.29941E-12 | 47.23094325 |
| rs2647268 | G | A | 4 | 0.00133471 | 9.79941E-32 | 137.4110696 |
| rs2676298 | T | C | 17 | 0.00186213 | 0.000000021 | 31.42241464 |
| rs2678204 | G | T | 1 | 0.00136897 | 1.80011E-19 | 81.4591395 |
| rs2699433 | T | C | 4 | 0.00185061 | 2.80027E-12 | 48.82034319 |
| rs2735559 | A | G | 3 | 0.00203875 | 2.09991E-16 | 67.54239686 |
| rs2737218 | C | T | 8 | 0.00160651 | 1.80011E-23 | 99.66912153 |
| rs2740761 | T | C | 7 | 0.00162009 | 3.29997E-08 | 30.50300353 |
| rs2744700 | T | C | 1 | 0.00131495 | 4.60045E-15 | 61.43331294 |
| rs2789370 | G | A | 1 | 0.00136241 | 1.59993E-12 | 49.867496 |
| rs28457693 | G | A | 9 | 0.00211432 | 3.10027E-34 | 148.8191819 |
| rs284662 | C | T | 19 | 0.00134232 | 1.40001E-10 | 41.13461326 |
| rs28605759 | A | G | 1 | 0.00130141 | 4.10015E-13 | 52.58564956 |
| rs28640931 | A | C | 17 | 0.0014034 | 0.000000016 | 31.93184086 |
| rs286501 | C | G | 11 | 0.00246777 | 9.20005E-09 | 33.00531065 |
| rs2866719 | T | C | 7 | 0.00135359 | 7.70016E-11 | 42.33364496 |
| rs2920891 | A | C | 2 | 0.00131106 | 6.80002E-09 | 33.59941853 |
| rs2952615 | C | G | 5 | 0.00133799 | 3.90032E-17 | 70.85014377 |
| rs2978360 | T | C | 18 | 0.00131011 | 3.59998E-09 | 34.80928846 |
| rs2979655 | G | T | 8 | 0.00176638 | 2.19999E-08 | 31.32812731 |
| rs30235 | T | C | 16 | 0.00132782 | 8.70001E-10 | 37.60592918 |
| rs310796 | T | G | 12 | 0.00139938 | 6.4998E-14 | 56.22052652 |
| rs3110496 | G | A | 17 | 0.0014032 | 3.79997E-09 | 34.73318097 |
| rs3116201 | A | G | 2 | 0.00218602 | 5.40008E-12 | 47.53655948 |
| rs332162 | C | A | 10 | 0.00162378 | 2.90001E-08 | 30.77106971 |
| rs34017457 | A | G | 16 | 0.0078328 | 1.29999E-10 | 41.29434103 |
| rs34055910 | G | A | 17 | 0.00134593 | 5.19996E-09 | 34.0945694 |
| rs34118426 | G | A | 12 | 0.00141413 | 1.10002E-13 | 55.2491916 |
| rs34517439 | A | C | 1 | 0.00200706 | 6.4998E-80 | 358.3440361 |
| rs34667744 | C | T | 8 | 0.0013089 | 0.000000032 | 30.61236686 |
| rs34693680 | T | C | 3 | 0.00191731 | 1.10002E-11 | 46.08402304 |
| rs34772064 | G | T | 4 | 0.00130728 | 7.79992E-09 | 33.31227386 |
| rs34776209 | T | C | 7 | 0.00151012 | 3.59998E-24 | 102.8381764 |
| rs34848742 | G | T | 4 | 0.00158935 | 2.90001E-19 | 80.52333836 |
| rs34879158 | C | A | 20 | 0.0014824 | 1.80011E-43 | 191.1291328 |
| rs35050648 | T | G | 19 | 0.00153545 | 0.000000017 | 31.781778 |
| rs35105141 | T | C | 16 | 0.00132808 | 2.39994E-60 | 268.5178037 |
| rs35506085 | A | G | 11 | 0.00168888 | 1.9002E-24 | 104.0922362 |
| rs357486 | C | T | 3 | 0.00130281 | 6.59933E-16 | 65.24293385 |
| rs357868 | T | G | 18 | 0.00132465 | 4.30031E-15 | 61.57270125 |
| rs35874463 | G | A | 15 | 0.00279337 | 4.20001E-08 | 30.03641279 |
| rs35990522 | A | T | 9 | 0.00247729 | 1.5E-10 | 41.04345749 |
| rs36000545 | G | A | 17 | 0.00135004 | 3.80014E-31 | 134.7101672 |
| rs36012032 | A | C | 3 | 0.00226206 | 8.00018E-16 | 64.86455728 |
| rs3730071 | A | C | 12 | 0.00379765 | 3.29989E-12 | 48.52247834 |
| rs3736984 | G | A | 6 | 0.0017193 | 1.99986E-11 | 44.99129945 |
| rs3744806 | G | C | 17 | 0.00142046 | 8.79995E-10 | 37.58518795 |
| rs3803575 | C | T | 16 | 0.0014072 | 1.59993E-17 | 72.57896941 |
| rs3809570 | A | C | 15 | 0.00152311 | 1.50003E-18 | 77.20020535 |
| rs3810291 | A | G | 19 | 0.0013912 | 1.20005E-37 | 164.5075446 |
| rs3822742 | A | C | 5 | 0.00134847 | 3.69999E-26 | 111.9518947 |
| rs3823674 | T | C | 7 | 0.00131737 | 1.99986E-15 | 63.04338543 |
| rs3827910 | G | A | 14 | 0.00153494 | 1.80011E-13 | 54.17575309 |
| rs3843750 | G | C | 19 | 0.00138209 | 8.19974E-20 | 82.99025572 |
| rs3853252 | A | G | 6 | 0.00131 | 2.80027E-28 | 121.6087025 |
| rs390801 | C | T | 4 | 0.00147924 | 9.3994E-12 | 46.45877903 |
| rs4073155 | A | G | 9 | 0.00137995 | 0.00000002 | 31.46291701 |
| rs4073717 | T | G | 5 | 0.00162107 | 4.60045E-23 | 97.82939928 |
| rs41311445 | C | A | 22 | 0.00222108 | 4.19952E-24 | 102.5745761 |
| rs4235012 | C | T | 4 | 0.00131378 | 1.69981E-12 | 49.78121029 |
| rs4240326 | G | A | 4 | 0.0013054 | 2.60016E-54 | 240.8416768 |
| rs4242244 | G | T | 5 | 0.00132033 | 3.40001E-09 | 34.92863445 |
| rs4282339 | A | G | 5 | 0.0015984 | 1E-23 | 100.7396007 |
| rs4392169 | T | A | 18 | 0.00158696 | 1.50003E-34 | 150.3210488 |
| rs4477562 | T | C | 13 | 0.00196325 | 1.59993E-21 | 90.77457261 |
| rs4525525 | T | G | 17 | 0.0014982 | 1.59993E-12 | 49.8794955 |
| rs4567604 | T | G | 13 | 0.00165906 | 8E-10 | 37.76751674 |
| rs4635681 | G | A | 3 | 0.00178775 | 2.99999E-09 | 35.20831658 |
| rs4648450 | A | C | 1 | 0.00130749 | 2.19999E-08 | 31.33555731 |
| rs4653016 | A | C | 1 | 0.00139665 | 8.00018E-23 | 96.70535777 |
| rs4682001 | C | T | 3 | 0.00219508 | 7.8001E-11 | 42.29490323 |
| rs4704496 | T | C | 5 | 0.00158671 | 3.29997E-08 | 30.5112615 |
| rs4727295 | A | G | 7 | 0.00172735 | 1.59993E-21 | 90.83805354 |
| rs4733724 | G | A | 8 | 0.00161498 | 2.09991E-15 | 62.97438332 |
| rs4776970 | T | A | 15 | 0.00135768 | 7.39946E-16 | 65.03247328 |
| rs4782286 | A | G | 16 | 0.0016002 | 4.70002E-15 | 61.37090194 |
| rs4808845 | G | A | 19 | 0.00133458 | 1.2E-09 | 36.96324385 |
| rs4819021 | C | T | 21 | 0.00131984 | 1.50003E-13 | 54.59107604 |
| rs4865956 | A | T | 5 | 0.0014179 | 4.70002E-11 | 43.28060461 |
| rs4899012 | C | G | 14 | 0.00133466 | 1.29987E-47 | 210.1029246 |
| rs4955853 | T | C | 3 | 0.00134135 | 8.10028E-12 | 46.73570097 |
| rs4973924 | A | G | 3 | 0.00130394 | 3.40017E-17 | 71.08281958 |
| rs4980826 | A | C | 12 | 0.00132791 | 4.30002E-08 | 30.02176078 |
| rs5017213 | C | T | 1 | 0.00135604 | 3.89996E-09 | 34.67711123 |
| rs505575 | C | T | 5 | 0.00138768 | 8.9002E-12 | 46.54722603 |
| rs508347 | C | T | 7 | 0.00142374 | 7.39997E-10 | 37.89950595 |
| rs512692 | T | A | 3 | 0.00144209 | 6.70039E-21 | 87.95572813 |
| rs529736 | C | A | 12 | 0.00174192 | 1.2E-09 | 36.94145761 |
| rs532499 | C | T | 13 | 0.00149006 | 2.80001E-08 | 30.85531627 |
| rs536007 | T | G | 11 | 0.00142087 | 4.90004E-19 | 79.45895459 |
| rs5396 | C | T | 3 | 0.00144995 | 2.09991E-11 | 44.85123945 |
| rs55758152 | A | G | 5 | 0.00140254 | 4.39997E-08 | 29.97869724 |
| rs55831773 | T | C | 17 | 0.00166341 | 6.29941E-20 | 83.52127716 |
| rs55854145 | C | A | 18 | 0.00287531 | 0.00000002 | 31.4530247 |
| rs56254146 | A | G | 2 | 0.00189448 | 2.19989E-15 | 62.84784901 |
| rs56304870 | A | T | 7 | 0.00254823 | 1.09999E-08 | 32.5750065 |
| rs56383938 | G | A | 7 | 0.00230341 | 3.19963E-12 | 48.58351627 |
| rs56676529 | A | C | 6 | 0.0019142 | 0.000000017 | 31.83914158 |
| rs57126421 | G | A | 18 | 0.00154136 | 2.19989E-13 | 53.78342373 |
| rs5752989 | A | G | 22 | 0.00131937 | 2.29985E-13 | 53.74198887 |
| rs5771118 | C | T | 22 | 0.00150014 | 1.09999E-10 | 41.62913598 |
| rs58584712 | A | G | 2 | 0.00158989 | 5.50047E-13 | 52.02758159 |
| rs59985551 | T | C | 2 | 0.00154922 | 6.89922E-33 | 142.6732654 |
| rs60223575 | C | T | 13 | 0.00141903 | 2.19999E-09 | 35.81622212 |
| rs6026578 | G | C | 20 | 0.00135375 | 6.4998E-14 | 56.21205626 |
| rs6084183 | C | T | 20 | 0.00131651 | 3.59998E-10 | 39.3084452 |
| rs6124249 | C | T | 20 | 0.00142574 | 2.39999E-10 | 40.07960774 |
| rs6142059 | C | T | 20 | 0.0013066 | 2.09991E-12 | 49.40865502 |
| rs61729527 | T | C | 8 | 0.00293686 | 6.59933E-16 | 65.24057903 |
| rs61878760 | A | G | 11 | 0.00237186 | 5.99998E-09 | 33.84381862 |
| rs61941043 | T | A | 12 | 0.00673795 | 3.79997E-10 | 39.2110773 |
| rs62064607 | A | C | 17 | 0.00346457 | 2.90001E-12 | 48.78650956 |
| rs62070645 | A | C | 17 | 0.00146953 | 1.50003E-52 | 232.7237419 |
| rs62378779 | T | C | 5 | 0.00143782 | 6.20012E-16 | 65.36863416 |
| rs62515438 | G | T | 8 | 0.00155197 | 1.59993E-28 | 122.7702895 |
| rs62621197 | T | C | 19 | 0.0035788 | 1.39991E-18 | 77.44963929 |
| rs62621400 | G | C | 15 | 0.00280065 | 1.39991E-11 | 45.60836473 |
| rs62621812 | A | G | 7 | 0.00474632 | 7.70016E-18 | 74.02723063 |
| rs6435043 | G | C | 2 | 0.00140256 | 3.89996E-08 | 30.20092398 |
| rs6437277 | G | A | 2 | 0.00154787 | 5.40008E-16 | 65.6513786 |
| rs6493780 | G | A | 15 | 0.00226891 | 1.20005E-11 | 46.01496944 |
| rs6505044 | C | A | 17 | 0.00130894 | 8.40001E-10 | 37.6744464 |
| rs6514066 | G | T | 20 | 0.00134766 | 9.29994E-09 | 32.97496359 |
| rs6570509 | T | G | 6 | 0.00143488 | 8.10028E-19 | 78.48062249 |
| rs6575340 | A | G | 14 | 0.00135774 | 1.59993E-13 | 54.44242974 |
| rs6585827 | A | G | 10 | 0.00130296 | 2.29985E-18 | 76.40678016 |
| rs6591 | T | C | 11 | 0.00131305 | 2.29985E-13 | 53.69042725 |
| rs66462629 | A | C | 11 | 0.00206554 | 2.19999E-08 | 31.28833635 |
| rs6669139 | T | C | 1 | 0.00159928 | 5.70033E-26 | 111.0822259 |
| rs6669189 | T | C | 1 | 0.00132676 | 3.19963E-13 | 53.09872 |
| rs67141907 | T | C | 13 | 0.0018358 | 0.00000002 | 31.49265115 |
| rs6743060 | A | C | 2 | 0.00172009 | 1.3002E-103 | 467.2422712 |
| rs67551338 | T | C | 12 | 0.00273823 | 2.09991E-19 | 81.1698848 |
| rs6761949 | C | T | 2 | 0.00163442 | 2.30001E-10 | 40.21792472 |
| rs6762578 | A | G | 3 | 0.0015704 | 1.20005E-21 | 91.40061307 |
| rs6800021 | A | G | 3 | 0.00131511 | 4.49987E-26 | 111.5302768 |
| rs68156080 | G | A | 10 | 0.00146698 | 1.99986E-11 | 44.95464009 |
| rs6840229 | A | G | 4 | 0.00130953 | 2.29985E-11 | 44.71538575 |
| rs6873192 | G | A | 5 | 0.00130118 | 7.70016E-17 | 69.48970988 |
| rs6874130 | C | G | 5 | 0.00158321 | 7.00003E-15 | 60.5955074 |
| rs6874142 | G | T | 5 | 0.00216621 | 4.49987E-14 | 56.9475458 |
| rs6888717 | A | G | 5 | 0.0016597 | 1.09999E-09 | 37.1103175 |
| rs6899378 | T | C | 6 | 0.00129749 | 4.60045E-11 | 43.32019592 |
| rs6902789 | A | G | 6 | 0.00135233 | 6.79986E-14 | 56.11171281 |
| rs695922 | G | A | 5 | 0.00177744 | 1.09999E-09 | 37.11339297 |
| rs7016759 | C | T | 8 | 0.00174568 | 2.39994E-14 | 58.13267565 |
| rs7033487 | C | T | 9 | 0.00163615 | 9.60064E-40 | 174.051589 |
| rs705953 | G | A | 6 | 0.00137649 | 4.19952E-11 | 43.52650035 |
| rs7107076 | T | C | 11 | 0.00148831 | 9.30037E-14 | 55.50941306 |
| rs7111235 | C | T | 11 | 0.00130879 | 1.5E-10 | 41.06866939 |
| rs7129320 | A | G | 11 | 0.00174857 | 8.60003E-35 | 151.3911563 |
| rs7132908 | A | G | 12 | 0.00133732 | 2.80027E-44 | 194.8338517 |
| rs7134283 | A | G | 12 | 0.00144743 | 1.29987E-14 | 59.4336037 |
| rs7145429 | T | C | 14 | 0.00329595 | 0.000000015 | 32.10131195 |
| rs7188009 | A | G | 16 | 0.00132835 | 1.89998E-09 | 36.03948837 |
| rs724016 | G | A | 3 | 0.0013064 | 4.00037E-100 | 451.1787022 |
| rs72656010 | C | T | 8 | 0.0019281 | 6.20012E-66 | 294.1489181 |
| rs72659403 | G | A | 4 | 0.00243005 | 4.79999E-09 | 34.28563798 |
| rs72697614 | A | C | 1 | 0.00140783 | 0.000000002 | 35.93703699 |
| rs72733810 | G | A | 9 | 0.00163661 | 0.000000016 | 31.87483177 |
| rs72828544 | G | C | 6 | 0.00165714 | 4.10015E-14 | 57.14011165 |
| rs73004967 | G | A | 19 | 0.00259166 | 3.10027E-17 | 71.30975705 |
| rs73013411 | A | C | 6 | 0.00192741 | 7.29962E-12 | 46.94483239 |
| rs73052033 | C | T | 3 | 0.00167545 | 2.09991E-26 | 113.0840467 |
| rs73175572 | G | A | 3 | 0.00208357 | 4.60045E-38 | 166.3771506 |
| rs73189390 | A | G | 21 | 0.00169437 | 9.40005E-09 | 32.96471819 |
| rs73601548 | T | C | 10 | 0.00205177 | 1.59993E-12 | 49.96413153 |
| rs73619441 | G | T | 20 | 0.00185936 | 4.49997E-10 | 38.87247802 |
| rs7372674 | A | C | 3 | 0.00135481 | 2.69998E-10 | 39.88206697 |
| rs738084 | G | A | 22 | 0.00165495 | 4.09996E-08 | 30.12308123 |
| rs742698 | C | T | 20 | 0.00142538 | 8.69961E-11 | 42.10247014 |
| rs74494415 | T | C | 18 | 0.00336278 | 1.59993E-13 | 54.41825093 |
| rs7487292 | G | T | 12 | 0.00131712 | 2.99985E-14 | 57.76678591 |
| rs74900203 | G | C | 6 | 0.00210922 | 1.29999E-10 | 41.24445381 |
| rs751894 | T | C | 18 | 0.00161645 | 2.19999E-08 | 31.29192723 |
| rs752070 | G | A | 2 | 0.00195499 | 2.99999E-10 | 39.66462991 |
| rs7527948 | G | A | 1 | 0.00209774 | 4.49997E-09 | 34.3867903 |
| rs7535501 | T | C | 1 | 0.00282304 | 1.40001E-08 | 32.24402406 |
| rs757608 | G | A | 17 | 0.00138452 | 4.10015E-17 | 70.74251871 |
| rs7619139 | A | T | 3 | 0.00132327 | 6.29941E-25 | 106.2990031 |
| rs76364830 | A | G | 8 | 0.00269453 | 3.29989E-18 | 75.70865694 |
| rs76513770 | C | T | 16 | 0.00194865 | 7.39946E-17 | 69.56295304 |
| rs76693355 | C | T | 11 | 0.00203348 | 1.99986E-15 | 63.10193939 |
| rs7671110 | T | C | 4 | 0.00177844 | 1.59993E-54 | 241.7702896 |
| rs76875574 | A | C | 15 | 0.0029232 | 9.90011E-09 | 32.85785392 |
| rs77165542 | T | C | 2 | 0.00354815 | 6.29941E-77 | 344.624643 |
| rs7740107 | A | T | 6 | 0.00147259 | 1.20005E-67 | 301.9507731 |
| rs7755185 | G | A | 6 | 0.0014041 | 3.69999E-09 | 34.79731118 |
| rs7780752 | C | T | 7 | 0.00135296 | 1.20005E-23 | 100.558073 |
| rs7781964 | A | G | 7 | 0.00167521 | 1.20005E-12 | 50.46518896 |
| rs77848106 | A | C | 1 | 0.00143157 | 1.89998E-10 | 40.55509159 |
| rs7793710 | G | C | 7 | 0.00144542 | 6.70039E-14 | 56.14264305 |
| rs7824070 | T | C | 8 | 0.00139333 | 1.2E-10 | 41.40877655 |
| rs7824350 | G | A | 8 | 0.0013301 | 1.80011E-11 | 45.12382901 |
| rs78378222 | G | T | 17 | 0.00604353 | 1.29987E-39 | 173.4136421 |
| rs7912286 | G | A | 10 | 0.0013422 | 1.80011E-11 | 45.21886117 |
| rs7916385 | T | C | 10 | 0.00198078 | 2.70023E-12 | 48.90491194 |
| rs7952436 | T | C | 11 | 0.00237211 | 9.60064E-33 | 142.0235816 |
| rs7959830 | T | G | 12 | 0.00132009 | 1.50003E-78 | 352.0686845 |
| rs7969505 | C | G | 12 | 0.0020596 | 2.49977E-11 | 44.49905649 |
| rs7977788 | A | G | 12 | 0.00156086 | 7.50067E-39 | 169.9812133 |
| rs7985813 | A | G | 13 | 0.00148336 | 0.000000016 | 31.97490176 |
| rs798759 | G | A | 4 | 0.00129982 | 9.20026E-12 | 46.49684535 |
| rs7994573 | C | T | 13 | 0.00153727 | 7.00003E-12 | 47.02772383 |
| rs8006178 | C | A | 14 | 0.00155563 | 0.00000004 | 30.13488631 |
| rs8007644 | A | G | 14 | 0.00134844 | 2.99999E-10 | 39.65177302 |
| rs8021250 | A | G | 14 | 0.00151955 | 1.09999E-08 | 32.64942975 |
| rs8023812 | G | A | 15 | 0.00178298 | 3.50002E-09 | 34.88163178 |
| rs8031196 | T | C | 15 | 0.0013126 | 1.20005E-15 | 64.1475785 |
| rs8073817 | T | G | 17 | 0.00161282 | 3.2E-09 | 35.06766034 |
| rs8074074 | T | C | 17 | 0.00151908 | 1.69981E-11 | 45.30345894 |
| rs821116 | A | G | 8 | 0.00141564 | 2.29985E-18 | 76.43090236 |
| rs823118 | T | C | 1 | 0.00130153 | 7.70016E-26 | 110.4814145 |
| rs8413 | C | T | 9 | 0.00131279 | 4.09996E-08 | 30.09581205 |
| rs843374 | T | A | 3 | 0.00132019 | 6.29941E-21 | 88.06851248 |
| rs867529 | C | G | 2 | 0.0014432 | 1.79999E-09 | 36.218429 |
| rs9295 | A | G | 2 | 0.00142544 | 2.49977E-18 | 76.27058829 |
| rs9327336 | C | T | 5 | 0.00136808 | 0.000000015 | 32.08756093 |
| rs9344126 | C | T | 6 | 0.00131209 | 8.19974E-12 | 46.72863238 |
| rs9480933 | G | C | 6 | 0.00136197 | 1.29987E-21 | 91.18548291 |
| rs9513143 | G | A | 13 | 0.00130755 | 2.49977E-13 | 53.54706963 |
| rs9540493 | G | A | 13 | 0.00131591 | 2.29985E-13 | 53.7037219 |
| rs9549099 | C | T | 13 | 0.0014822 | 1.10002E-11 | 46.09917095 |
| rs9588151 | T | C | 13 | 0.00140301 | 1.29999E-08 | 32.2951456 |
| rs9784689 | A | C | 5 | 0.00154297 | 4.40048E-24 | 102.4635128 |
| rs981938 | A | G | 2 | 0.00139775 | 1.80011E-36 | 159.0242343 |
| rs9888796 | C | T | 16 | 0.00149158 | 1.89998E-10 | 40.54953324 |
| rs9889839 | G | A | 17 | 0.00132169 | 0.000000021 | 31.44247227 |
| rs9915368 | G | A | 17 | 0.00206161 | 5.90065E-17 | 70.02543336 |
| rs9920235 | T | C | 15 | 0.00133795 | 8.99912E-12 | 46.5411637 |
| rs9957318 | G | A | 18 | 0.00137663 | 1.2E-10 | 41.5196714 |

**Table S10**. Genetic instruments used in the analyses for the association of Trunk fat-free mass with Heart failure.

| SNP | EA | OA | chr | SE | *P* value | F |
| --- | --- | --- | --- | --- | --- | --- |
| rs10020631 | A | G | 4 | 0.00143688 | 1.5E-09 | 36.58857295 |
| rs1005696 | G | T | 21 | 0.00126476 | 1.29999E-08 | 32.25910551 |
| rs10119967 | C | A | 9 | 0.00153816 | 3.90032E-30 | 130.0841863 |
| rs10128597 | A | G | 11 | 0.00139359 | 3.40001E-09 | 34.96443738 |
| rs10170082 | C | T | 2 | 0.00183283 | 4.49987E-20 | 84.18848197 |
| rs10203320 | C | T | 2 | 0.00132242 | 5.60015E-13 | 51.99742367 |
| rs1022523 | A | G | 1 | 0.00138521 | 2.09991E-31 | 135.9429976 |
| rs10226050 | G | A | 7 | 0.00124156 | 1.50003E-13 | 54.59593029 |
| rs10242866 | T | C | 7 | 0.00126305 | 5.40008E-14 | 56.56926334 |
| rs10269774 | A | G | 7 | 0.00131848 | 4.10015E-97 | 437.3385883 |
| rs1040617 | G | A | 14 | 0.00124982 | 8.9E-09 | 33.06226997 |
| rs10423120 | G | A | 19 | 0.00160138 | 5.79963E-12 | 47.38343857 |
| rs1047891 | A | C | 2 | 0.00132446 | 2.09991E-36 | 158.7467577 |
| rs10485622 | G | A | 20 | 0.00156664 | 1.59993E-28 | 122.7611495 |
| rs1049193 | C | G | 12 | 0.00164276 | 1.10002E-13 | 55.23016949 |
| rs10492145 | G | A | 12 | 0.00139036 | 3.40001E-08 | 30.49091189 |
| rs10498672 | G | C | 6 | 0.00161843 | 3.59998E-12 | 48.34125511 |
| rs1064213 | A | G | 2 | 0.00123336 | 3.69999E-19 | 80.02165286 |
| rs10748128 | T | G | 12 | 0.00130055 | 8.49963E-24 | 101.1520438 |
| rs10775348 | G | A | 16 | 0.00135971 | 1.10002E-22 | 96.07520426 |
| rs10822055 | A | T | 10 | 0.00146216 | 7.19946E-13 | 51.500275 |
| rs10835381 | G | A | 11 | 0.00127782 | 2.99985E-12 | 48.71282281 |
| rs10861681 | T | C | 12 | 0.00135302 | 2.99985E-13 | 53.17960388 |
| rs10870597 | G | A | 13 | 0.00146121 | 2.99985E-12 | 48.65692853 |
| rs10899736 | A | G | 7 | 0.00124619 | 2.70023E-14 | 57.97359892 |
| rs10931008 | C | T | 2 | 0.00130023 | 8.4004E-20 | 82.9459297 |
| rs10943915 | T | A | 6 | 0.00135573 | 7.29995E-10 | 37.92627547 |
| rs10948 | T | G | 19 | 0.0013126 | 1.9002E-25 | 108.6702209 |
| rs11001399 | C | A | 10 | 0.00125039 | 2.60016E-21 | 89.81131542 |
| rs11014285 | A | G | 10 | 0.0016866 | 1.10002E-31 | 137.1423467 |
| rs11021307 | T | C | 11 | 0.00125332 | 0.000000012 | 32.45177233 |
| rs11042725 | A | C | 11 | 0.00123635 | 8.80035E-26 | 110.2096632 |
| rs11065015 | T | C | 12 | 0.00387002 | 1.59993E-11 | 45.40484093 |
| rs111365325 | T | C | 5 | 0.00146721 | 4.49987E-23 | 97.87133397 |
| rs111391498 | G | A | 4 | 0.00289986 | 8.60003E-20 | 82.89961021 |
| rs11142705 | A | G | 9 | 0.00126149 | 6.4E-10 | 38.20731196 |
| rs111903923 | G | T | 2 | 0.00162531 | 6.09958E-11 | 42.77459862 |
| rs11198591 | A | G | 10 | 0.00128491 | 1.9002E-11 | 45.06854002 |
| rs11217863 | A | G | 11 | 0.00193373 | 7.10068E-12 | 47.00507013 |
| rs11237265 | T | C | 11 | 0.00130169 | 4.20001E-08 | 30.07351486 |
| rs11243202 | C | T | 6 | 0.00123841 | 8.30042E-58 | 256.8622165 |
| rs11245450 | A | G | 10 | 0.0012652 | 1E-12 | 50.81350421 |
| rs112544217 | T | C | 2 | 0.00429099 | 8.49963E-12 | 46.63947216 |
| rs112560164 | A | G | 14 | 0.0015804 | 1.69981E-17 | 72.42328779 |
| rs1126339 | A | G | 14 | 0.00123869 | 1.79999E-08 | 31.73932292 |
| rs11376 | T | C | 14 | 0.00124229 | 0.000000032 | 30.58344896 |
| rs113907898 | G | C | 16 | 0.0029522 | 2.90001E-08 | 30.78892823 |
| rs11485595 | T | C | 1 | 0.00123737 | 1.5E-10 | 40.96933129 |
| rs115179432 | G | A | 2 | 0.00239772 | 2.19989E-22 | 94.67601983 |
| rs11545482 | T | C | 2 | 0.00434858 | 5.79963E-13 | 51.93018945 |
| rs11588850 | G | A | 1 | 0.00164522 | 1.50003E-13 | 54.60692737 |
| rs11590433 | G | A | 1 | 0.00129559 | 8.10028E-15 | 60.31852543 |
| rs11618507 | T | G | 13 | 0.00148499 | 3.40017E-17 | 71.11525424 |
| rs11622992 | C | T | 14 | 0.00126764 | 2E-10 | 40.49355857 |
| rs11635080 | T | G | 15 | 0.0014276 | 1.80011E-13 | 54.1774779 |
| rs11647120 | G | A | 16 | 0.00156153 | 3.50026E-14 | 57.40983423 |
| rs11684531 | G | A | 2 | 0.00181525 | 1.50003E-11 | 45.59271779 |
| rs11695471 | A | T | 2 | 0.00131296 | 2.29985E-18 | 76.41861811 |
| rs11709402 | G | A | 3 | 0.0013819 | 2.69998E-09 | 35.37688592 |
| rs1171336 | G | A | 1 | 0.00123854 | 1.69981E-11 | 45.25254443 |
| rs117451679 | G | A | 12 | 0.00203712 | 0.000000016 | 31.9783943 |
| rs117543413 | T | C | 10 | 0.00476149 | 1.9002E-14 | 58.59289681 |
| rs1176314 | G | T | 13 | 0.00125268 | 1.40001E-09 | 36.6524169 |
| rs11779459 | T | C | 8 | 0.00129913 | 2.29985E-11 | 44.65605084 |
| rs11785562 | A | G | 8 | 0.00156112 | 1.59993E-15 | 63.5022527 |
| rs11794152 | G | A | 9 | 0.00125769 | 4.10015E-14 | 57.13416923 |
| rs1179721 | G | A | 2 | 0.00139768 | 4.30002E-09 | 34.50267976 |
| rs118173451 | C | T | 22 | 0.00499978 | 2.59998E-09 | 35.47872576 |
| rs11863963 | C | G | 16 | 0.00128155 | 0.00000001 | 32.82778968 |
| rs1187428 | C | A | 14 | 0.00135016 | 1.89998E-08 | 31.5934815 |
| rs11880992 | A | G | 19 | 0.00126159 | 2.60016E-32 | 140.0537607 |
| rs11937249 | T | G | 4 | 0.00128395 | 2.30001E-09 | 35.6828388 |
| rs11974610 | A | G | 7 | 0.00156879 | 1.29999E-09 | 36.77138044 |
| rs11997525 | A | T | 8 | 0.00165893 | 3.10027E-22 | 94.05116729 |
| rs12026358 | T | C | 1 | 0.00129427 | 2E-10 | 40.47380786 |
| rs12072845 | A | G | 1 | 0.0012615 | 1.9002E-30 | 131.5462617 |
| rs12095997 | T | C | 1 | 0.00215882 | 1.50003E-27 | 118.27099 |
| rs12106274 | C | G | 20 | 0.00131782 | 1.29987E-13 | 54.84439728 |
| rs12119249 | T | C | 1 | 0.00185651 | 4.49997E-10 | 38.90115758 |
| rs12140153 | T | G | 1 | 0.00216148 | 9.79941E-12 | 46.3667822 |
| rs12148418 | A | G | 15 | 0.00124136 | 1.80011E-19 | 81.46699843 |
| rs12156265 | A | G | 8 | 0.00126008 | 1.40001E-10 | 41.17042002 |
| rs12209223 | A | C | 6 | 0.00205213 | 1.99986E-18 | 76.64321486 |
| rs1228024 | A | C | 11 | 0.00130793 | 1.39991E-19 | 81.93341157 |
| rs12314162 | T | C | 12 | 0.00168173 | 5.60015E-32 | 138.5348303 |
| rs12344515 | T | C | 9 | 0.00145152 | 4E-10 | 39.1116019 |
| rs1242518 | T | C | 17 | 0.00141984 | 2.69998E-10 | 39.84498874 |
| rs12467963 | T | A | 2 | 0.00126692 | 7.79992E-09 | 33.32261558 |
| rs12509014 | T | C | 4 | 0.00151209 | 7.10068E-22 | 92.38152931 |
| rs12533655 | A | G | 7 | 0.00130237 | 8.00018E-12 | 46.77361789 |
| rs12552167 | T | C | 9 | 0.00137033 | 1E-10 | 41.82761917 |
| rs12572123 | A | G | 10 | 0.00140815 | 1.40001E-09 | 36.63621315 |
| rs1260326 | C | T | 2 | 0.00125855 | 5.30029E-55 | 244.0029922 |
| rs12656549 | T | C | 5 | 0.00131807 | 2.59998E-08 | 30.99608707 |
| rs12657771 | A | G | 5 | 0.00125053 | 6.20012E-26 | 110.9216435 |
| rs12670224 | A | G | 7 | 0.00142168 | 0.000000032 | 30.56308436 |
| rs12713004 | G | A | 2 | 0.00138136 | 1.80011E-42 | 186.5841766 |
| rs12744382 | C | T | 1 | 0.0021815 | 5.99998E-09 | 33.83649973 |
| rs12764498 | C | T | 10 | 0.00193265 | 2.60016E-16 | 67.06844269 |
| rs12821683 | C | G | 12 | 0.00181225 | 0.000000016 | 31.87629725 |
| rs12831751 | C | A | 12 | 0.00136896 | 7.10068E-12 | 47.00774336 |
| rs12981554 | G | A | 19 | 0.00125334 | 2.80027E-12 | 48.83482117 |
| rs13014796 | A | G | 2 | 0.00165066 | 7.8001E-14 | 55.84589035 |
| rs13155458 | A | G | 5 | 0.00135097 | 1.29999E-08 | 32.39573581 |
| rs13156484 | A | G | 5 | 0.00124946 | 2.19989E-49 | 218.2043438 |
| rs13220932 | T | C | 7 | 0.00230233 | 2.99985E-15 | 62.27825939 |
| rs13240065 | A | G | 7 | 0.00184569 | 8.9002E-24 | 101.0549487 |
| rs13245726 | G | T | 7 | 0.00196516 | 1.09999E-08 | 32.74626021 |
| rs13271368 | T | C | 8 | 0.00147439 | 5.50047E-24 | 102.0286473 |
| rs13283416 | G | T | 9 | 0.00125593 | 1.99986E-16 | 67.61756676 |
| rs13340461 | T | C | 6 | 0.00138364 | 1.20005E-27 | 118.7568152 |
| rs1341215 | A | G | 9 | 0.00179749 | 1.99986E-11 | 44.92442559 |
| rs13430869 | T | G | 2 | 0.00140932 | 1.50003E-23 | 100.0922643 |
| rs1351394 | C | T | 12 | 0.00123515 | 4.7973E-102 | 459.9994367 |
| rs1356164 | A | G | 2 | 0.00133072 | 3.89996E-09 | 34.69554542 |
| rs1363902 | T | C | 5 | 0.00129699 | 0.000000025 | 31.06696399 |
| rs1374370 | A | G | 2 | 0.00133892 | 5.10035E-15 | 61.23823447 |
| rs139030 | G | A | 22 | 0.0015051 | 2.99999E-09 | 35.18662817 |
| rs141866277 | T | A | 15 | 0.00406039 | 1.09999E-08 | 32.62568445 |
| rs143384 | G | A | 20 | 0.00126115 | 1.7989E-184 | 839.0258992 |
| rs1443750 | G | C | 7 | 0.00128264 | 3.40017E-17 | 71.09309569 |
| rs146851424 | C | A | 13 | 0.00429759 | 1.69981E-45 | 200.4359235 |
| rs147110934 | T | G | 19 | 0.00402318 | 4.70002E-16 | 65.91090258 |
| rs1472852 | A | C | 4 | 0.00168862 | 9.20026E-92 | 412.7493866 |
| rs147461490 | T | C | 14 | 0.00357003 | 1E-11 | 46.26398767 |
| rs1519480 | T | C | 11 | 0.00132307 | 3.50026E-31 | 134.8949158 |
| rs1521624 | A | C | 15 | 0.00124781 | 1.50003E-11 | 45.59250049 |
| rs1542224 | C | T | 2 | 0.00137277 | 4.60045E-18 | 75.03865226 |
| rs1573891 | C | G | 15 | 0.00170653 | 5.10035E-44 | 193.6229042 |
| rs1578992 | T | G | 1 | 0.00130531 | 6.4998E-13 | 51.69578162 |
| rs1591806 | G | A | 6 | 0.00124568 | 6.89922E-44 | 193.0370364 |
| rs1624064 | C | T | 6 | 0.00125096 | 2.70023E-13 | 53.45167293 |
| rs16844418 | C | T | 4 | 0.00176615 | 2.99985E-12 | 48.68620454 |
| rs1690789 | T | C | 1 | 0.00123243 | 9.79941E-16 | 64.47877724 |
| rs16916881 | A | C | 8 | 0.00145609 | 2.90001E-12 | 48.74621056 |
| rs16942324 | A | C | 15 | 0.00381466 | 3.90032E-26 | 111.8465408 |
| rs17011108 | C | T | 4 | 0.0013506 | 8.9E-09 | 33.06471387 |
| rs17096799 | T | A | 12 | 0.0037353 | 3.50002E-08 | 30.40081799 |
| rs17115481 | A | G | 5 | 0.00139428 | 6.69993E-10 | 38.1206254 |
| rs1717775 | G | T | 11 | 0.00147813 | 7.00003E-10 | 38.03157434 |
| rs17246129 | A | G | 2 | 0.00134087 | 2.70023E-27 | 117.1432051 |
| rs17277008 | C | T | 1 | 0.00132759 | 2.70023E-31 | 135.4046081 |
| rs17363646 | G | A | 1 | 0.00179653 | 1.39991E-12 | 50.23181693 |
| rs1741344 | T | C | 20 | 0.00128612 | 4.90004E-09 | 34.23549193 |
| rs17556750 | A | C | 4 | 0.00136407 | 1.10002E-17 | 73.24926375 |
| rs1805165 | A | C | 2 | 0.00137077 | 8.80035E-15 | 60.14657249 |
| rs1813212 | G | A | 11 | 0.00124733 | 2E-10 | 40.45409939 |
| rs1841738 | G | A | 4 | 0.00124331 | 3.90032E-20 | 84.45286401 |
| rs1927635 | C | T | 9 | 0.0013085 | 1.29987E-13 | 54.80649668 |
| rs1928850 | T | A | 9 | 0.00232045 | 4.60045E-11 | 43.34200369 |
| rs1951455 | C | T | 14 | 0.00137701 | 4.40048E-11 | 43.41761127 |
| rs1971955 | G | A | 12 | 0.00175349 | 1.29987E-16 | 68.41023542 |
| rs1986868 | G | A | 8 | 0.0013811 | 2.39994E-13 | 53.6359234 |
| rs2005172 | C | A | 17 | 0.00130498 | 6.4003E-78 | 349.1850311 |
| rs2015561 | G | A | 17 | 0.00125169 | 1.9002E-21 | 90.45268379 |
| rs2034923 | C | A | 12 | 0.00123647 | 4.00037E-11 | 43.59767149 |
| rs2035901 | G | A | 4 | 0.00124015 | 2.29985E-29 | 126.5554697 |
| rs204886 | C | T | 6 | 0.00122994 | 2.60016E-53 | 236.2449528 |
| rs2051815 | A | G | 19 | 0.00132968 | 2.80001E-08 | 30.84739451 |
| rs2055981 | C | T | 3 | 0.00129829 | 4.90004E-09 | 34.21099734 |
| rs2104449 | T | G | 1 | 0.00137154 | 6.70039E-14 | 56.14232595 |
| rs2140046 | C | T | 2 | 0.00128149 | 5.70033E-23 | 97.37675684 |
| rs2172839 | G | A | 3 | 0.00161582 | 3.50002E-10 | 39.3579921 |
| rs2197563 | A | G | 2 | 0.00125703 | 4.00037E-16 | 66.23468172 |
| rs2225226 | T | C | 13 | 0.00150971 | 7.29962E-89 | 399.4490906 |
| rs224048 | A | G | 10 | 0.00124136 | 9.90011E-09 | 32.8630691 |
| rs2240735 | T | C | 16 | 0.001424 | 7.00003E-27 | 115.2381142 |
| rs2252720 | T | C | 20 | 0.00133192 | 1.10002E-29 | 128.0296532 |
| rs2265309 | C | T | 10 | 0.00123901 | 2.80027E-27 | 117.0411702 |
| rs2270894 | G | C | 3 | 0.00159286 | 1.29987E-27 | 118.5149201 |
| rs2281175 | C | T | 1 | 0.00132061 | 1.39991E-17 | 72.83968015 |
| rs2291256 | T | C | 12 | 0.00218487 | 3.80014E-13 | 52.72118337 |
| rs2296316 | C | T | 14 | 0.00125158 | 8.60003E-21 | 87.45428264 |
| rs2307111 | C | T | 5 | 0.00126413 | 6.4998E-31 | 133.6547652 |
| rs236587 | C | T | 17 | 0.00142102 | 5.19996E-09 | 34.10250298 |
| rs2421992 | T | C | 1 | 0.0013624 | 0.000000017 | 31.83364823 |
| rs244711 | T | C | 5 | 0.00142777 | 2.49977E-42 | 185.9181155 |
| rs2457982 | A | G | 6 | 0.00138704 | 1.80011E-14 | 58.7847056 |
| rs2474901 | A | G | 6 | 0.0012912 | 4.20001E-08 | 30.0683034 |
| rs2504235 | G | A | 13 | 0.00129013 | 9.49992E-10 | 37.42892493 |
| rs2521349 | A | G | 17 | 0.00127625 | 1.79999E-10 | 40.69325295 |
| rs2530399 | C | G | 14 | 0.00130476 | 2.59998E-08 | 31.02154296 |
| rs254963 | G | A | 5 | 0.00125154 | 1.99986E-12 | 49.44431251 |
| rs2569400 | A | C | 19 | 0.00127081 | 1.10002E-14 | 59.68222372 |
| rs2578557 | T | C | 5 | 0.00128991 | 1.20005E-12 | 50.54008342 |
| rs25849 | G | C | 16 | 0.00137162 | 1.50003E-37 | 163.9905416 |
| rs2602713 | C | A | 19 | 0.00125995 | 4.30031E-24 | 102.4932258 |
| rs2647239 | G | A | 4 | 0.00125664 | 4.49987E-43 | 189.2901595 |
| rs2650965 | G | A | 20 | 0.00132284 | 1.50003E-11 | 45.53750041 |
| rs2651316 | A | G | 3 | 0.00143555 | 8.10009E-09 | 33.26241206 |
| rs2724616 | G | A | 12 | 0.00129312 | 2.39994E-33 | 144.7481854 |
| rs2768950 | G | A | 4 | 0.00138756 | 9.8992E-12 | 46.34593509 |
| rs2785078 | C | T | 10 | 0.00124964 | 4.49987E-12 | 47.88585953 |
| rs2803888 | A | C | 1 | 0.00125082 | 6.00067E-13 | 51.86090871 |
| rs282303 | A | C | 5 | 0.00216561 | 0.000000001 | 37.27413139 |
| rs284315 | G | A | 1 | 0.00123022 | 4.40048E-12 | 47.9168654 |
| rs28504597 | A | G | 22 | 0.00255743 | 8.69961E-12 | 46.59365048 |
| rs28701981 | C | T | 9 | 0.00130273 | 5.19996E-52 | 230.2869677 |
| rs2885697 | T | G | 1 | 0.00130427 | 1.20005E-56 | 251.6217552 |
| rs28929474 | T | C | 14 | 0.00445708 | 7.89951E-14 | 55.83983121 |
| rs291979 | A | G | 10 | 0.0014734 | 2.49977E-14 | 58.11294148 |
| rs2952615 | C | G | 5 | 0.00127183 | 2.19989E-20 | 85.63484512 |
| rs310796 | T | G | 12 | 0.00132941 | 5.19996E-16 | 65.70848647 |
| rs3110496 | G | A | 17 | 0.00133324 | 2.99985E-11 | 44.16400191 |
| rs3116201 | A | G | 2 | 0.00207665 | 1.9002E-20 | 85.85840886 |
| rs31210 | A | G | 5 | 0.0014238 | 1.99986E-22 | 94.91806481 |
| rs3218036 | A | G | 19 | 0.00132549 | 2.80027E-24 | 103.3670172 |
| rs332115 | A | G | 10 | 0.00138498 | 6.89922E-12 | 47.06838926 |
| rs34017457 | A | G | 16 | 0.00745146 | 2.80001E-10 | 39.82099033 |
| rs34118426 | G | A | 12 | 0.00134339 | 2.49977E-20 | 85.34701436 |
| rs34289700 | C | A | 1 | 0.00152356 | 2.80027E-11 | 44.30569683 |
| rs343952 | A | G | 2 | 0.00170726 | 2.90001E-10 | 39.72367583 |
| rs34478611 | A | G | 9 | 0.00144465 | 1.20005E-14 | 59.50974638 |
| rs34517439 | A | C | 1 | 0.00190726 | 2.80027E-75 | 337.0184918 |
| rs34552833 | G | A | 14 | 0.00146854 | 2.19999E-10 | 40.28187308 |
| rs34776209 | T | C | 7 | 0.00143517 | 1.10002E-36 | 160.0841971 |
| rs34879158 | C | A | 20 | 0.00140881 | 7.00003E-55 | 243.4300761 |
| rs35251247 | A | G | 11 | 0.00136993 | 5.30029E-12 | 47.57014747 |
| rs35268848 | A | C | 16 | 0.00659674 | 1.99986E-13 | 53.959814 |
| rs35506085 | A | G | 11 | 0.00160596 | 1.59993E-38 | 168.5052818 |
| rs35592623 | G | A | 8 | 0.00268112 | 9.3994E-11 | 41.95290968 |
| rs35658696 | G | A | 5 | 0.00276062 | 4.39997E-10 | 38.94542667 |
| rs35665085 | A | G | 22 | 0.00269971 | 8.19974E-12 | 46.70548929 |
| rs35710322 | C | T | 18 | 0.00124923 | 1.20005E-25 | 109.6118804 |
| rs357486 | C | T | 3 | 0.00123852 | 3.29989E-13 | 53.03795915 |
| rs35756741 | T | C | 12 | 0.00214527 | 1.50003E-15 | 63.59446714 |
| rs35811052 | G | A | 16 | 0.00141713 | 1E-15 | 64.34323627 |
| rs35874463 | G | A | 15 | 0.00265483 | 1.39991E-13 | 54.72799449 |
| rs35897671 | T | C | 5 | 0.00129994 | 7.89951E-13 | 51.30603189 |
| rs35915186 | C | T | 1 | 0.00150869 | 3.59998E-09 | 34.8538838 |
| rs36000545 | G | A | 17 | 0.00128273 | 1.20005E-36 | 159.8108475 |
| rs36089326 | T | A | 4 | 0.0013158 | 5.70033E-11 | 42.90757767 |
| rs3740591 | T | C | 10 | 0.0012575 | 1.59993E-21 | 90.78187477 |
| rs3751866 | C | T | 16 | 0.00143843 | 2.69998E-09 | 35.38323915 |
| rs3772051 | A | G | 2 | 0.00147383 | 0.00000001 | 32.79336568 |
| rs3782232 | A | G | 12 | 0.0024051 | 6.4003E-19 | 78.95199166 |
| rs3808424 | C | T | 8 | 0.00149801 | 5.19996E-23 | 97.56245086 |
| rs3809569 | G | A | 15 | 0.00144741 | 2.90001E-23 | 98.69985475 |
| rs3818416 | C | A | 13 | 0.00146384 | 4.30031E-21 | 88.82300193 |
| rs3822742 | A | C | 5 | 0.00128184 | 2.29985E-23 | 99.20740589 |
| rs3850625 | A | G | 1 | 0.00190688 | 2.19989E-21 | 90.15974946 |
| rs3853252 | A | G | 6 | 0.00124365 | 2.80027E-35 | 153.6292129 |
| rs3913369 | T | G | 3 | 0.00145042 | 4.90004E-13 | 52.22469251 |
| rs3925 | A | G | 8 | 0.00144056 | 1.20005E-13 | 55.01669263 |
| rs4076108 | T | A | 3 | 0.00144109 | 9.09913E-12 | 46.52211355 |
| rs41271299 | T | C | 6 | 0.00279359 | 7.89951E-61 | 270.7231625 |
| rs41311445 | C | A | 22 | 0.00211238 | 8.60003E-31 | 133.0955086 |
| rs4252548 | T | C | 19 | 0.00422923 | 7.00003E-18 | 74.22875083 |
| rs4282339 | A | G | 5 | 0.00151931 | 2.70023E-40 | 176.5798427 |
| rs4369779 | C | T | 18 | 0.00151775 | 3.59998E-81 | 364.1049843 |
| rs4373831 | G | A | 10 | 0.00205233 | 1.09999E-08 | 32.581266 |
| rs4439140 | A | G | 8 | 0.00128596 | 1.2E-10 | 41.42331158 |
| rs4477562 | T | C | 13 | 0.0018671 | 7.29962E-17 | 69.58674616 |
| rs4532931 | C | A | 10 | 0.00127523 | 7.10068E-12 | 47.00014605 |
| rs45474992 | T | C | 19 | 0.00335547 | 1.29987E-30 | 132.3541743 |
| rs45528934 | T | C | 14 | 0.00168053 | 6.79986E-24 | 101.5986938 |
| rs4635681 | G | A | 3 | 0.00169946 | 5.89997E-10 | 38.33826753 |
| rs4653175 | G | A | 1 | 0.00142623 | 2.39999E-08 | 31.11305979 |
| rs4677151 | A | G | 3 | 0.00124145 | 1.99986E-11 | 44.98163236 |
| rs4681994 | C | T | 3 | 0.00123774 | 1.20005E-19 | 82.30298296 |
| rs4711965 | A | T | 6 | 0.00164293 | 6.20012E-25 | 106.3374173 |
| rs4762962 | A | G | 12 | 0.00137398 | 2.99999E-10 | 39.65402335 |
| rs4788218 | C | T | 16 | 0.00126369 | 1.20005E-63 | 283.7044915 |
| rs4793982 | C | T | 17 | 0.00341566 | 3.89996E-10 | 39.16732671 |
| rs4794665 | G | A | 17 | 0.0012383 | 5.40008E-11 | 43.01328086 |
| rs4795318 | T | C | 17 | 0.00123998 | 5.30029E-14 | 56.62089762 |
| rs4845852 | T | C | 1 | 0.00140325 | 1.59993E-24 | 104.4918846 |
| rs485047 | C | G | 9 | 0.00137702 | 2.49977E-14 | 58.06780438 |
| rs485554 | C | G | 3 | 0.00133404 | 9.3994E-45 | 197.0074981 |
| rs4858940 | C | T | 3 | 0.00193909 | 1.99986E-14 | 58.57902002 |
| rs4864606 | A | G | 4 | 0.00132788 | 8.4004E-11 | 42.15812152 |
| rs4899012 | C | G | 14 | 0.00126856 | 1E-69 | 311.5145972 |
| rs4912537 | T | C | 3 | 0.00145008 | 1E-16 | 68.87667559 |
| rs4912650 | G | T | 5 | 0.00142655 | 6.20012E-11 | 42.74275011 |
| rs4952818 | T | C | 2 | 0.00123528 | 2.30001E-09 | 35.71834982 |
| rs4968799 | T | A | 17 | 0.00196129 | 1.59993E-22 | 95.3054742 |
| rs4973930 | C | A | 3 | 0.00123878 | 8.00018E-14 | 55.80805678 |
| rs4980067 | A | C | 10 | 0.00123703 | 4.30031E-20 | 84.27187459 |
| rs4980826 | A | C | 12 | 0.0012615 | 2.19999E-10 | 40.26206804 |
| rs498685 | C | T | 18 | 0.00126102 | 4.79954E-11 | 43.25131154 |
| rs5015933 | C | T | 9 | 0.00124338 | 3.90032E-14 | 57.2084225 |
| rs508347 | C | T | 7 | 0.00135313 | 1.39991E-22 | 95.57886206 |
| rs514328 | A | G | 19 | 0.00204265 | 2.1E-10 | 40.34222716 |
| rs553597 | A | T | 7 | 0.00125976 | 3.69999E-09 | 34.75322251 |
| rs55665822 | G | A | 5 | 0.00142302 | 1.10002E-16 | 68.69921746 |
| rs55674305 | A | G | 8 | 0.00134596 | 2.70023E-14 | 57.91486369 |
| rs55745410 | G | A | 1 | 0.00129394 | 8.69961E-26 | 110.2456181 |
| rs55758152 | A | G | 5 | 0.00133316 | 1.20005E-15 | 64.02664624 |
| rs55800172 | A | G | 1 | 0.00251822 | 7.50067E-13 | 51.40470096 |
| rs55831773 | T | C | 17 | 0.00158033 | 5.40008E-20 | 83.81841448 |
| rs56207600 | A | G | 11 | 0.00198087 | 2.30001E-09 | 35.67349355 |
| rs56311196 | T | C | 4 | 0.00177602 | 6.59994E-10 | 38.14158838 |
| rs57126421 | G | A | 18 | 0.00146572 | 1.69981E-11 | 45.31718731 |
| rs5742915 | C | T | 15 | 0.00124329 | 4.40048E-19 | 79.68917741 |
| rs57481308 | C | T | 14 | 0.0012798 | 6.4003E-13 | 51.70742664 |
| rs5752989 | A | G | 22 | 0.00125481 | 4.10015E-16 | 66.19794338 |
| rs5753630 | G | A | 22 | 0.00125489 | 8.4004E-11 | 42.17156342 |
| rs5754185 | G | C | 22 | 0.00175102 | 0.00000002 | 31.5113132 |
| rs578366 | G | A | 6 | 0.00124768 | 2.29985E-20 | 85.52928958 |
| rs58403320 | A | G | 9 | 0.00149304 | 5.90065E-13 | 51.88063165 |
| rs58416107 | A | G | 12 | 0.00136087 | 7.10068E-18 | 74.18663849 |
| rs59839808 | C | T | 18 | 0.00219762 | 4.39997E-08 | 29.97527871 |
| rs59985551 | T | C | 2 | 0.00147166 | 5.30029E-36 | 156.9123683 |
| rs6026578 | G | C | 20 | 0.0012866 | 1.39991E-13 | 54.69203727 |
| rs603321 | G | A | 6 | 0.00144145 | 4.70002E-18 | 75.02015004 |
| rs60804050 | A | G | 1 | 0.00141009 | 2.29985E-17 | 71.90603164 |
| rs6081869 | G | T | 20 | 0.00128995 | 1.59993E-19 | 81.72092723 |
| rs6117348 | T | C | 20 | 0.00130221 | 1.99986E-17 | 72.14188204 |
| rs6142059 | C | T | 20 | 0.0012418 | 4.00037E-14 | 57.14473631 |
| rs61482805 | G | C | 9 | 0.00131963 | 1.09999E-09 | 37.17928515 |
| rs61729527 | T | C | 8 | 0.00279134 | 1.9002E-16 | 67.71150689 |
| rs61749613 | G | A | 5 | 0.00309609 | 6.4003E-19 | 78.92910848 |
| rs61878760 | A | G | 11 | 0.00225525 | 9.20026E-12 | 46.49180847 |
| rs61884730 | T | C | 11 | 0.00145287 | 8.40001E-09 | 33.18575799 |
| rs62107261 | C | T | 2 | 0.00288315 | 2.80027E-61 | 272.8160033 |
| rs62160102 | G | C | 2 | 0.00159232 | 3.29997E-09 | 35.01997556 |
| rs62325220 | C | G | 4 | 0.00173608 | 2.49977E-17 | 71.7058003 |
| rs62338991 | C | T | 4 | 0.00203318 | 3.89996E-08 | 30.18624685 |
| rs62372052 | G | A | 5 | 0.00198011 | 1.69981E-47 | 209.5681396 |
| rs62439025 | C | G | 6 | 0.00172271 | 1.40001E-09 | 36.60955576 |
| rs62466118 | A | G | 7 | 0.00395479 | 5.10035E-11 | 43.15602824 |
| rs62476192 | T | C | 7 | 0.00220009 | 2.39994E-11 | 44.64729335 |
| rs62515437 | T | G | 8 | 0.00148156 | 2.09991E-42 | 186.2028574 |
| rs62621197 | T | C | 19 | 0.00340161 | 2.19989E-21 | 90.19585582 |
| rs62621812 | A | G | 7 | 0.00451151 | 5.90065E-21 | 88.20674153 |
| rs6448733 | G | A | 4 | 0.00130351 | 1.6E-10 | 40.94582167 |
| rs6450346 | C | T | 5 | 0.00135039 | 1.10002E-23 | 100.6497535 |
| rs6493780 | G | A | 15 | 0.00215634 | 5.39995E-10 | 38.52222986 |
| rs6500249 | G | A | 16 | 0.00140992 | 6.4E-10 | 38.20935971 |
| rs6502119 | T | C | 17 | 0.00128416 | 3.50026E-12 | 48.37437774 |
| rs6570509 | T | G | 6 | 0.0013622 | 2.29985E-28 | 122.0342075 |
| rs6575985 | T | C | 14 | 0.0013754 | 3.50026E-14 | 57.43400497 |
| rs6585827 | A | G | 10 | 0.00123852 | 2.60016E-19 | 80.73454591 |
| rs6603813 | G | T | 1 | 0.00141661 | 1.80011E-12 | 49.68676999 |
| rs663344 | C | G | 9 | 0.00163302 | 5.60015E-25 | 106.5331609 |
| rs66674044 | T | A | 16 | 0.00177078 | 1.39991E-20 | 86.4673762 |
| rs6680471 | T | C | 1 | 0.00123536 | 0.000000025 | 31.07140141 |
| rs6719296 | A | G | 2 | 0.00123971 | 8.30042E-14 | 55.73314821 |
| rs6744298 | G | T | 2 | 0.00124313 | 4.30002E-08 | 30.02244456 |
| rs67551338 | T | C | 12 | 0.00260133 | 5.60015E-24 | 102.0004257 |
| rs6762578 | A | G | 3 | 0.0014929 | 3.50026E-27 | 116.6295829 |
| rs6764533 | A | G | 3 | 0.00128801 | 1.09999E-08 | 32.72508665 |
| rs68119703 | T | C | 2 | 0.00124631 | 5.19996E-09 | 34.11609332 |
| rs6874142 | G | T | 5 | 0.00205909 | 4.90004E-20 | 84.00648466 |
| rs6900690 | A | G | 6 | 0.00123991 | 4.09996E-10 | 39.0814805 |
| rs6902789 | A | G | 6 | 0.00128387 | 1.10002E-17 | 73.2732373 |
| rs6919534 | A | G | 6 | 0.00176219 | 5.00035E-32 | 138.7470333 |
| rs6936615 | G | A | 6 | 0.00165312 | 5.49997E-10 | 38.50120224 |
| rs6940643 | G | A | 6 | 0.00126127 | 3.69999E-09 | 34.75675866 |
| rs695922 | G | A | 5 | 0.00168948 | 2.59998E-08 | 30.97537702 |
| rs6972291 | C | T | 7 | 0.00162622 | 1E-12 | 50.76985307 |
| rs6984820 | T | C | 8 | 0.00125561 | 1E-19 | 82.5471112 |
| rs7016990 | C | T | 8 | 0.00132704 | 0.000000015 | 31.99998411 |
| rs7033487 | C | T | 9 | 0.00155475 | 1.59993E-55 | 246.3531899 |
| rs705953 | G | A | 6 | 0.00130682 | 2.99985E-13 | 53.19411902 |
| rs7080472 | T | G | 10 | 0.00125436 | 6.79986E-25 | 106.1555696 |
| rs708723 | T | C | 1 | 0.00123647 | 2.39994E-24 | 103.6313363 |
| rs7129320 | A | G | 11 | 0.00166266 | 1.29987E-45 | 200.9373517 |
| rs71301804 | A | C | 3 | 0.00209693 | 1.59993E-20 | 86.22632436 |
| rs7132908 | A | G | 12 | 0.00127047 | 3.69999E-25 | 107.3725098 |
| rs7134283 | A | G | 12 | 0.00137499 | 5.40008E-18 | 74.71353633 |
| rs71385734 | G | T | 16 | 0.0016589 | 5.70033E-46 | 202.5950422 |
| rs71423263 | G | T | 2 | 0.00177007 | 2.19999E-09 | 35.76162569 |
| rs71564907 | C | A | 7 | 0.00147864 | 2.39999E-08 | 31.1259763 |
| rs7188009 | A | G | 16 | 0.00126326 | 1.10002E-12 | 50.64592665 |
| rs7196459 | G | T | 16 | 0.00230541 | 2.30001E-08 | 31.2139599 |
| rs7223535 | A | G | 17 | 0.00139364 | 4.00037E-78 | 350.1203703 |
| rs7229351 | A | G | 18 | 0.00128559 | 1.69981E-16 | 67.95322541 |
| rs7245985 | G | T | 19 | 0.00153048 | 6.79986E-14 | 56.13636762 |
| rs7262524 | T | C | 20 | 0.00141198 | 1.79999E-10 | 40.70636647 |
| rs726547 | A | G | 15 | 0.0029611 | 4.19952E-14 | 57.05041946 |
| rs72656010 | C | T | 8 | 0.00183267 | 1.20005E-97 | 439.725112 |
| rs72660086 | G | T | 1 | 0.0015128 | 1.9002E-16 | 67.6704169 |
| rs72664840 | T | C | 14 | 0.00161723 | 5.60015E-12 | 47.45749061 |
| rs72703414 | G | A | 4 | 0.00250088 | 0.00000002 | 31.52085575 |
| rs72755233 | A | G | 15 | 0.00196653 | 2.80027E-15 | 62.42215672 |
| rs73052033 | C | T | 3 | 0.00159279 | 8.69961E-16 | 64.70691818 |
| rs73158212 | G | A | 7 | 0.00150062 | 4.79999E-08 | 29.81323589 |
| rs73175572 | G | A | 3 | 0.00198061 | 4.79954E-45 | 198.3290313 |
| rs73180805 | G | A | 12 | 0.00170339 | 8.19974E-15 | 60.27769087 |
| rs7318451 | T | C | 13 | 0.00166078 | 4.19952E-13 | 52.5471785 |
| rs7321045 | A | G | 13 | 0.00125505 | 0.000000021 | 31.43170023 |
| rs74048171 | A | C | 11 | 0.00141585 | 4.10015E-12 | 48.0946193 |
| rs744205 | A | G | 11 | 0.00124439 | 4.79954E-16 | 65.86203096 |
| rs74565893 | T | C | 11 | 0.00556292 | 2.39999E-10 | 40.11173349 |
| rs747680 | C | T | 20 | 0.00123789 | 9.90011E-09 | 32.86133003 |
| rs74841302 | A | G | 15 | 0.00477657 | 1.20005E-16 | 68.57226978 |
| rs7513326 | A | G | 1 | 0.00125345 | 0.000000012 | 32.48722612 |
| rs7514705 | C | T | 1 | 0.00124449 | 5.19996E-09 | 34.10671124 |
| rs7518221 | C | T | 1 | 0.00129022 | 1.80011E-14 | 58.78007543 |
| rs7520575 | T | A | 1 | 0.00131128 | 4.49987E-12 | 47.88019833 |
| rs752070 | G | A | 2 | 0.00185702 | 7.29995E-09 | 33.44092995 |
| rs7528419 | G | A | 1 | 0.00148128 | 1.09999E-10 | 41.56413838 |
| rs7542242 | T | C | 1 | 0.00132795 | 8.80035E-15 | 60.14408667 |
| rs7549783 | T | C | 1 | 0.00158956 | 1.09999E-09 | 37.22476832 |
| rs7559547 | T | C | 2 | 0.00162068 | 5.60015E-77 | 344.8465591 |
| rs7569435 | A | C | 2 | 0.00143601 | 9.69996E-10 | 37.38444357 |
| rs7610673 | G | A | 3 | 0.00129991 | 1.29999E-08 | 32.34119753 |
| rs7619139 | A | T | 3 | 0.00125796 | 8.60003E-17 | 69.26437542 |
| rs7633464 | A | G | 3 | 0.00123544 | 3.59998E-16 | 66.4470953 |
| rs76364830 | A | G | 8 | 0.00256122 | 6.79986E-18 | 74.265254 |
| rs76513770 | C | T | 16 | 0.00185307 | 1.59993E-16 | 68.04177719 |
| rs76733024 | G | A | 2 | 0.00250176 | 4.60002E-09 | 34.36858756 |
| rs7679463 | C | A | 4 | 0.0012435 | 0.00000001 | 32.84253135 |
| rs7689420 | C | T | 4 | 0.00164735 | 1.10002E-66 | 297.5125013 |
| rs7708460 | A | G | 5 | 0.00124857 | 1.6E-10 | 40.89085055 |
| rs77093479 | G | C | 17 | 0.00168097 | 2.80027E-11 | 44.30204837 |
| rs77159542 | G | A | 1 | 0.00248567 | 0.000000012 | 32.50367316 |
| rs77169818 | T | A | 18 | 0.0030903 | 2.09991E-15 | 62.94609569 |
| rs7728690 | T | C | 5 | 0.00126815 | 5.19996E-23 | 97.56731762 |
| rs7731023 | G | A | 5 | 0.00124805 | 1.29987E-13 | 54.77838222 |
| rs7740107 | A | T | 6 | 0.0013981 | 2.3988E-107 | 484.3368013 |
| rs7755185 | G | A | 6 | 0.00133297 | 1.29999E-10 | 41.24985704 |
| rs7755251 | G | A | 6 | 0.00125778 | 1.09999E-09 | 37.09971945 |
| rs77820836 | G | A | 7 | 0.00178937 | 3.29997E-10 | 39.47741129 |
| rs77848106 | A | C | 1 | 0.00136043 | 1.7E-10 | 40.78000104 |
| rs7794796 | T | C | 7 | 0.00132173 | 7.00003E-52 | 229.675902 |
| rs7808966 | C | A | 7 | 0.00127041 | 2.19999E-09 | 35.74645128 |
| rs7815955 | T | A | 8 | 0.00153795 | 4.90004E-27 | 115.9211811 |
| rs7824350 | G | A | 8 | 0.00126428 | 2.39999E-09 | 35.61284141 |
| rs78378222 | G | T | 17 | 0.00574202 | 2.19989E-47 | 209.075437 |
| rs7843128 | C | T | 8 | 0.00129335 | 1.9002E-11 | 45.05161658 |
| rs7873683 | C | T | 9 | 0.00125668 | 3.2E-09 | 35.07763408 |
| rs7909735 | T | G | 10 | 0.00141014 | 0.000000012 | 32.46697998 |
| rs7910211 | C | T | 10 | 0.00169534 | 4E-10 | 39.1017719 |
| rs7952436 | T | C | 11 | 0.00225551 | 4.10015E-55 | 244.5267247 |
| rs7961994 | T | A | 12 | 0.00126528 | 2.80027E-37 | 162.7536127 |
| rs79815595 | T | A | 3 | 0.00173632 | 4.30002E-09 | 34.50493367 |
| rs8002779 | A | G | 13 | 0.00126259 | 9.60064E-12 | 46.41636821 |
| rs8019890 | A | C | 14 | 0.0012601 | 1.10002E-20 | 87.01879723 |
| rs8091287 | C | T | 18 | 0.00146794 | 3.2E-09 | 35.03765085 |
| rs8130733 | G | A | 21 | 0.00147835 | 2.30001E-10 | 40.18909022 |
| rs822552 | G | C | 7 | 0.00140387 | 1.99986E-18 | 76.72884942 |
| rs834363 | T | G | 6 | 0.00196956 | 4.70002E-23 | 97.76140623 |
| rs836510 | T | C | 7 | 0.00158036 | 1E-10 | 41.76900571 |
| rs855206 | C | A | 12 | 0.0020711 | 1.20005E-16 | 68.60471921 |
| rs876122 | G | A | 6 | 0.00188893 | 3.69999E-08 | 30.30992334 |
| rs8904 | A | G | 14 | 0.00128692 | 4.49987E-11 | 43.36275511 |
| rs894360 | C | T | 8 | 0.00128853 | 5.70033E-53 | 234.6791066 |
| rs903678 | A | G | 1 | 0.00130205 | 1.50003E-17 | 72.72963445 |
| rs9291926 | G | T | 5 | 0.00123862 | 4.30031E-24 | 102.5102708 |
| rs9295 | A | G | 2 | 0.00135406 | 6.00067E-12 | 47.32187308 |
| rs9327336 | C | T | 5 | 0.00130044 | 4.60045E-12 | 47.83378917 |
| rs9350850 | C | T | 6 | 0.00227817 | 2.60016E-28 | 121.7392004 |
| rs9379084 | A | G | 6 | 0.00198682 | 1.20005E-17 | 73.08777465 |
| rs946197 | C | G | 1 | 0.00146278 | 1.29987E-23 | 100.2435203 |
| rs9465505 | A | G | 6 | 0.00124006 | 1.39991E-11 | 45.73025287 |
| rs9472487 | T | G | 6 | 0.00125415 | 4.90004E-10 | 38.70969917 |
| rs9479012 | G | A | 6 | 0.00210668 | 5.39995E-09 | 34.0501178 |
| rs9480933 | G | C | 6 | 0.00129305 | 5.40008E-24 | 102.0630435 |
| rs9491200 | G | A | 6 | 0.00166498 | 1.29999E-08 | 32.26822429 |
| rs9513510 | C | G | 13 | 0.0013508 | 2.49977E-17 | 71.68101804 |
| rs9540493 | G | A | 13 | 0.00125136 | 6.89922E-12 | 47.06048152 |
| rs9594693 | A | G | 13 | 0.00168292 | 1.2E-10 | 41.46131879 |
| rs9634212 | A | C | 12 | 0.00149544 | 2.80027E-65 | 291.1141879 |
| rs968821 | C | G | 9 | 0.00130575 | 1E-17 | 73.42813178 |
| rs9800418 | C | T | 5 | 0.00144316 | 3.59998E-08 | 30.33138314 |
| rs9810734 | T | A | 3 | 0.00138274 | 2.39999E-09 | 35.65819115 |
| rs981938 | A | G | 2 | 0.00132775 | 1.10002E-38 | 169.1635492 |
| rs983399 | T | C | 7 | 0.0012383 | 6.20012E-23 | 97.2142174 |
| rs9838614 | G | T | 3 | 0.00126688 | 6.00067E-22 | 92.73911353 |
| rs9853018 | T | C | 3 | 0.00124163 | 1.4997E-136 | 618.6716183 |
| rs9884540 | G | T | 4 | 0.00127434 | 2.59998E-09 | 35.4870328 |
| rs9892365 | G | A | 17 | 0.00131765 | 1.20005E-43 | 191.9685801 |
| rs9925273 | G | A | 16 | 0.00161072 | 1.50003E-11 | 45.52764028 |
| rs9951619 | G | T | 18 | 0.0014767 | 1.59993E-15 | 63.5457402 |
| rs9960148 | T | G | 18 | 0.00127241 | 9.09997E-10 | 37.51233972 |
| rs9960619 | T | C | 18 | 0.00130543 | 4.60045E-15 | 61.4107059 |

**Table S11**. Genetic instruments used in the analyses for the association of Whole fat-free mass with Heart failure.

| SNP | EA | OA | chr | SE | *P* value | F |
| --- | --- | --- | --- | --- | --- | --- |
| rs10020631 | A | G | 4 | 0.00144209 | 8.19993E-09 | 33.23859232 |
| rs1008158 | G | A | 9 | 0.0013173 | 1.09999E-10 | 41.58482595 |
| rs10119967 | C | A | 9 | 0.00154444 | 5.90065E-24 | 101.8839034 |
| rs10172196 | A | G | 2 | 0.00134675 | 1.29987E-15 | 63.84683391 |
| rs10188231 | G | C | 2 | 0.0015909 | 2.69998E-10 | 39.87578356 |
| rs10202845 | G | A | 2 | 0.00197265 | 4.30031E-19 | 79.7234937 |
| rs10222924 | G | A | 4 | 0.00135692 | 1.99986E-12 | 49.49825341 |
| rs1022523 | A | G | 1 | 0.00139049 | 4.40048E-29 | 125.2984703 |
| rs10248298 | A | C | 7 | 0.00128664 | 8.49963E-19 | 78.37665597 |
| rs10269774 | A | G | 7 | 0.00132369 | 4.70002E-87 | 391.1471463 |
| rs10282707 | T | C | 7 | 0.00126782 | 3.59998E-10 | 39.3024024 |
| rs10283100 | G | A | 8 | 0.00271462 | 8.69961E-25 | 105.6617868 |
| rs10401784 | A | C | 19 | 0.00129734 | 1.29987E-14 | 59.45727739 |
| rs10412300 | G | C | 19 | 0.00136884 | 3.29997E-08 | 30.49551383 |
| rs10434434 | C | A | 4 | 0.00175181 | 2.39994E-15 | 62.73421983 |
| rs1043801 | A | G | 21 | 0.00267794 | 1.79999E-08 | 31.70193662 |
| rs1047891 | A | C | 2 | 0.0013297 | 6.00067E-37 | 161.2558091 |
| rs1049193 | C | G | 12 | 0.00164953 | 4.60045E-16 | 65.95720746 |
| rs10498672 | G | C | 6 | 0.00162526 | 1.6E-10 | 40.85953672 |
| rs10511111 | C | T | 3 | 0.00134157 | 7.89951E-11 | 42.28445547 |
| rs1064213 | A | G | 2 | 0.00123828 | 1.80011E-20 | 85.96500977 |
| rs10748128 | T | G | 12 | 0.00130585 | 3.19963E-21 | 89.44609694 |
| rs10770705 | C | A | 12 | 0.00131169 | 2.80001E-10 | 39.77979892 |
| rs10775348 | G | A | 16 | 0.00136464 | 1.99986E-21 | 90.38538313 |
| rs10775406 | G | A | 17 | 0.00145435 | 4.70002E-11 | 43.28623889 |
| rs10777860 | A | G | 12 | 0.00124243 | 7.8001E-18 | 73.99602605 |
| rs10780905 | A | G | 9 | 0.00127253 | 8.4004E-15 | 60.24220464 |
| rs10803955 | G | A | 2 | 0.00123547 | 6.4003E-19 | 78.94536883 |
| rs10843397 | T | C | 12 | 0.00144658 | 7.59976E-11 | 42.3554799 |
| rs10861678 | A | G | 12 | 0.00140468 | 6.20012E-14 | 56.30767916 |
| rs10870597 | G | A | 13 | 0.00146679 | 1E-12 | 50.80761502 |
| rs10881583 | C | T | 9 | 0.0014564 | 3.40001E-10 | 39.43303605 |
| rs10899736 | A | G | 7 | 0.00125107 | 4.40048E-16 | 66.06572288 |
| rs10908289 | A | G | 1 | 0.0016163 | 7.49998E-09 | 33.41187603 |
| rs10956488 | G | A | 8 | 0.00172779 | 4.49987E-16 | 66.01474328 |
| rs10979612 | C | T | 9 | 0.00238714 | 4.90004E-15 | 61.28393329 |
| rs10995366 | A | G | 10 | 0.00143974 | 1.10002E-11 | 46.07632995 |
| rs11001399 | C | A | 10 | 0.00125526 | 5.19996E-19 | 79.36018409 |
| rs11014285 | A | G | 10 | 0.00169316 | 1.10002E-29 | 128.0705893 |
| rs11021307 | T | C | 11 | 0.00125805 | 3.59998E-08 | 30.36005182 |
| rs11030112 | A | G | 11 | 0.00133184 | 3.59998E-36 | 157.7213416 |
| rs11042725 | A | C | 11 | 0.00124104 | 3.10027E-21 | 89.48796556 |
| rs11049684 | T | C | 12 | 0.00135474 | 4.79954E-15 | 61.35035675 |
| rs11052457 | T | A | 12 | 0.00324324 | 2.09991E-12 | 49.35815879 |
| rs11071182 | G | A | 15 | 0.00185999 | 6.29941E-13 | 51.74460084 |
| rs111365325 | T | C | 5 | 0.00147276 | 9.60064E-23 | 96.34569603 |
| rs111391498 | G | A | 4 | 0.00291033 | 6.70039E-19 | 78.84139998 |
| rs11142700 | C | T | 9 | 0.00126675 | 3.29997E-10 | 39.48689992 |
| rs111598585 | T | C | 4 | 0.00153055 | 0.00000002 | 31.46773472 |
| rs11160601 | T | C | 14 | 0.00216547 | 6.4998E-13 | 51.67731348 |
| rs111640872 | C | G | 19 | 0.00132728 | 1.10002E-27 | 118.9027841 |
| rs11198591 | A | G | 10 | 0.00128997 | 1.2E-09 | 37.0050817 |
| rs11205354 | A | C | 1 | 0.00125214 | 3.69999E-10 | 39.25505747 |
| rs11243202 | C | T | 6 | 0.00124355 | 6.29941E-49 | 216.1518282 |
| rs11245450 | A | G | 10 | 0.00127016 | 7.00003E-16 | 65.14661563 |
| rs112544217 | T | C | 2 | 0.00430743 | 4.79999E-10 | 38.76608488 |
| rs112560164 | A | G | 14 | 0.00158669 | 1.29987E-16 | 68.48021711 |
| rs112957890 | G | A | 14 | 0.00142409 | 2.09991E-11 | 44.83503702 |
| rs113619763 | T | A | 22 | 0.00257877 | 8.00018E-11 | 42.25652856 |
| rs113978196 | A | G | 15 | 0.0014337 | 1.79999E-08 | 31.74701922 |
| rs114949263 | C | T | 7 | 0.00197625 | 2.49977E-11 | 44.53489734 |
| rs115179432 | G | A | 2 | 0.00240719 | 4.49987E-20 | 84.20068802 |
| rs11545482 | T | C | 2 | 0.00436594 | 4.40048E-13 | 52.45340663 |
| rs11611651 | A | G | 12 | 0.00219189 | 4.30031E-14 | 57.03853878 |
| rs116165844 | T | G | 20 | 0.00180724 | 4.30002E-09 | 34.47509926 |
| rs116337081 | T | C | 2 | 0.00242956 | 3.59998E-09 | 34.82989649 |
| rs116817990 | G | A | 1 | 0.0034489 | 5.69994E-09 | 33.94298075 |
| rs11684531 | G | A | 2 | 0.00182251 | 6.59994E-10 | 38.14282852 |
| rs1168759 | A | T | 12 | 0.00417011 | 1.40001E-08 | 32.12355057 |
| rs11695471 | A | T | 2 | 0.00131813 | 8.30042E-17 | 69.33679997 |
| rs11709402 | G | A | 3 | 0.00138691 | 7.10068E-13 | 51.52979152 |
| rs11712872 | A | G | 3 | 0.00193812 | 4.30031E-18 | 75.16800174 |
| rs117451679 | G | A | 12 | 0.00204565 | 5E-10 | 38.66077806 |
| rs117543413 | T | C | 10 | 0.00477995 | 1.20005E-15 | 64.03146863 |
| rs1176314 | G | T | 13 | 0.00125747 | 3.59998E-09 | 34.85080386 |
| rs11785562 | A | G | 8 | 0.00156717 | 2.70023E-14 | 57.94547737 |
| rs11794152 | G | A | 9 | 0.00126284 | 2.39994E-16 | 67.25833833 |
| rs1179905 | G | A | 6 | 0.00157064 | 6.29941E-11 | 42.72426448 |
| rs118173451 | C | T | 22 | 0.00501933 | 6.29999E-09 | 33.7394168 |
| rs1184893 | A | G | 12 | 0.00150699 | 2.09991E-13 | 53.88235481 |
| rs11855017 | A | C | 15 | 0.00161542 | 4.79954E-16 | 65.86796165 |
| rs11880992 | A | G | 19 | 0.00126679 | 4.60045E-28 | 120.6114713 |
| rs11925245 | G | A | 3 | 0.00162039 | 0.000000001 | 37.31767546 |
| rs11937249 | T | G | 4 | 0.00128883 | 5.1E-10 | 38.65324901 |
| rs11997525 | A | T | 8 | 0.0016654 | 3.90032E-20 | 84.45029496 |
| rs12041740 | A | G | 1 | 0.00140844 | 2.09991E-25 | 108.4485248 |
| rs12047986 | G | A | 1 | 0.00123837 | 5.1E-10 | 38.64148785 |
| rs12070699 | T | C | 1 | 0.00124653 | 1.50003E-13 | 54.53094943 |
| rs12072845 | A | G | 1 | 0.00126632 | 2.80027E-28 | 121.6467967 |
| rs12095997 | T | C | 1 | 0.00216716 | 2.70023E-25 | 107.9847765 |
| rs12140153 | T | G | 1 | 0.00216969 | 3.80014E-15 | 61.80015366 |
| rs12156265 | A | G | 8 | 0.00126504 | 1.09999E-10 | 41.64689051 |
| rs1218826 | G | A | 13 | 0.00132355 | 5.00035E-12 | 47.69214412 |
| rs12209223 | A | C | 6 | 0.00206082 | 2.60016E-13 | 53.52556628 |
| rs1228024 | A | C | 11 | 0.00131283 | 2.09991E-16 | 67.52003899 |
| rs12375196 | A | C | 7 | 0.00126298 | 6.4998E-14 | 56.21496927 |
| rs12427047 | T | C | 12 | 0.00144602 | 2.09991E-11 | 44.90923997 |
| rs1243872 | G | T | 9 | 0.00124871 | 2.29985E-11 | 44.72492568 |
| rs12525009 | T | C | 6 | 0.00125353 | 2.99999E-10 | 39.69986125 |
| rs12533548 | G | A | 7 | 0.00130715 | 4.60002E-10 | 38.8271567 |
| rs12553221 | A | G | 9 | 0.00129666 | 2.19999E-08 | 31.31090597 |
| rs1260326 | C | T | 2 | 0.00126353 | 1.10002E-50 | 224.1875368 |
| rs12657771 | A | G | 5 | 0.0012553 | 9.3994E-27 | 114.653546 |
| rs12658452 | G | T | 5 | 0.00210744 | 4.30002E-08 | 29.98791971 |
| rs12713004 | G | A | 2 | 0.00138687 | 3.90032E-35 | 152.9815475 |
| rs12729817 | G | A | 1 | 0.00123705 | 2.19989E-11 | 44.76167585 |
| rs12764498 | C | T | 10 | 0.00194006 | 1.59993E-15 | 63.56298891 |
| rs12795042 | C | A | 11 | 0.00129746 | 2.30001E-08 | 31.26247971 |
| rs12820906 | G | A | 12 | 0.00144104 | 3.90032E-16 | 66.29275523 |
| rs12879423 | G | A | 14 | 0.00133985 | 3.40017E-26 | 112.0910176 |
| rs12888955 | A | G | 14 | 0.00130873 | 1.40001E-08 | 32.1508141 |
| rs12889702 | C | A | 14 | 0.00134415 | 6.4003E-12 | 47.20691305 |
| rs12906197 | T | C | 15 | 0.0012676 | 9.3994E-18 | 73.62847585 |
| rs1296328 | C | A | 4 | 0.00125464 | 4.49987E-12 | 47.87450044 |
| rs13014796 | A | G | 2 | 0.00165731 | 6.20012E-13 | 51.78152364 |
| rs13043303 | A | G | 20 | 0.00160988 | 6.20012E-31 | 133.7354009 |
| rs13140382 | A | G | 4 | 0.0013707 | 4.70002E-08 | 29.85310518 |
| rs13240065 | A | G | 7 | 0.0018528 | 2.90001E-22 | 94.16372774 |
| rs13259194 | C | T | 8 | 0.00154838 | 1.5E-09 | 36.53845598 |
| rs13271368 | T | C | 8 | 0.00148017 | 1.99986E-23 | 99.43599751 |
| rs13272451 | T | G | 8 | 0.00125324 | 8.80035E-14 | 55.61731371 |
| rs13392079 | C | T | 2 | 0.00144153 | 3.79997E-09 | 34.7236367 |
| rs13430869 | T | G | 2 | 0.00141494 | 1.39991E-21 | 91.08405946 |
| rs1351394 | C | T | 12 | 0.00124023 | 5.30029E-96 | 432.2213609 |
| rs1374370 | A | G | 2 | 0.00134413 | 3.50026E-14 | 57.44506098 |
| rs1407031 | T | C | 20 | 0.00127585 | 1.2E-10 | 41.41936597 |
| rs1412234 | C | T | 9 | 0.00132697 | 2.60016E-16 | 67.0491399 |
| rs1413816 | T | C | 6 | 0.00129419 | 3.80014E-12 | 48.23936226 |
| rs143384 | G | A | 20 | 0.00126635 | 1.4997E-171 | 779.566439 |
| rs145594985 | T | C | 7 | 0.00179542 | 1.20005E-11 | 45.89556489 |
| rs146851424 | C | A | 13 | 0.0043141 | 1.59993E-43 | 191.3419792 |
| rs147110934 | T | G | 19 | 0.00403968 | 5.60015E-15 | 61.03360911 |
| rs148662000 | T | G | 1 | 0.00254491 | 2.99999E-09 | 35.1746113 |
| rs1521624 | A | C | 15 | 0.00125288 | 4.00037E-13 | 52.65535912 |
| rs1542224 | C | T | 2 | 0.00137825 | 1.99986E-20 | 85.79470431 |
| rs1573891 | C | G | 15 | 0.00171347 | 7.50067E-38 | 165.4047555 |
| rs1582931 | A | G | 5 | 0.00125234 | 8.69961E-51 | 224.655178 |
| rs1657222 | A | G | 10 | 0.00127422 | 1.7E-10 | 40.75324677 |
| rs16844418 | C | T | 4 | 0.00177258 | 1.9002E-12 | 49.61035796 |
| rs16916881 | A | C | 8 | 0.00146179 | 9.20026E-13 | 51.01691795 |
| rs16942324 | A | C | 15 | 0.00383077 | 2.19989E-22 | 94.67243214 |
| rs17011108 | C | T | 4 | 0.00135557 | 2.30001E-08 | 31.18836506 |
| rs17112250 | G | A | 10 | 0.00284368 | 0.000000012 | 32.45602953 |
| rs17115481 | A | G | 5 | 0.00139958 | 1.29999E-10 | 41.37376235 |
| rs17246129 | A | G | 2 | 0.00134623 | 1.39991E-20 | 86.49913286 |
| rs17277008 | C | T | 1 | 0.00133266 | 4.60045E-27 | 116.0810351 |
| rs17363646 | G | A | 1 | 0.00180347 | 5.50047E-12 | 47.50260478 |
| rs17400325 | C | T | 2 | 0.00309727 | 3.80014E-12 | 48.2454026 |
| rs17556750 | A | C | 4 | 0.00136906 | 3.69999E-18 | 75.49539106 |
| rs177252 | G | A | 5 | 0.0013462 | 9.30037E-13 | 50.98641033 |
| rs17828687 | A | C | 8 | 0.0012601 | 6.79986E-13 | 51.5891242 |
| rs1805165 | A | C | 2 | 0.00137619 | 2.49977E-13 | 53.60356333 |
| rs1813212 | G | A | 11 | 0.00125205 | 6.70039E-12 | 47.09946279 |
| rs182224 | T | C | 5 | 0.00217186 | 4.60002E-09 | 34.35093532 |
| rs1841738 | G | A | 4 | 0.00124786 | 3.19963E-19 | 80.31282869 |
| rs1910466 | C | T | 3 | 0.0012469 | 9.40005E-10 | 37.453438 |
| rs1924929 | G | A | 13 | 0.00148903 | 7.59976E-25 | 105.9322208 |
| rs1927635 | C | T | 9 | 0.00131385 | 5.00035E-12 | 47.68654861 |
| rs1928850 | T | A | 9 | 0.00232983 | 0.000000001 | 37.32338696 |
| rs2005172 | C | A | 17 | 0.00131036 | 1E-66 | 297.6938944 |
| rs2015561 | G | A | 17 | 0.0012569 | 1.9002E-20 | 85.92902064 |
| rs2071286 | T | C | 6 | 0.00159773 | 4.19952E-39 | 171.120648 |
| rs2077218 | A | G | 10 | 0.00146371 | 2.29985E-22 | 94.5931695 |
| rs2101975 | G | A | 4 | 0.00125533 | 7.39946E-39 | 169.9999852 |
| rs2104449 | T | G | 1 | 0.00137676 | 9.3994E-15 | 60.01083544 |
| rs2122823 | T | C | 7 | 0.00153938 | 1.99986E-11 | 44.9618782 |
| rs213997 | A | G | 6 | 0.00132866 | 5.99998E-09 | 33.82868767 |
| rs2140046 | C | T | 2 | 0.00128653 | 8.19974E-20 | 83.01351027 |
| rs2197563 | A | G | 2 | 0.00126196 | 3.80014E-16 | 66.31180081 |
| rs2225226 | T | C | 13 | 0.00151548 | 3.10027E-86 | 387.3687249 |
| rs2229840 | T | C | 12 | 0.00169246 | 1.50003E-33 | 145.7149657 |
| rs224048 | A | G | 10 | 0.00124616 | 3.79997E-09 | 34.71987481 |
| rs2240735 | T | C | 16 | 0.00142919 | 3.29989E-30 | 130.4331839 |
| rs2252720 | T | C | 20 | 0.00133737 | 4.60045E-24 | 102.3889096 |
| rs2252909 | T | C | 17 | 0.00133213 | 0.000000021 | 31.396264 |
| rs2265309 | C | T | 10 | 0.00124386 | 1.50003E-23 | 99.97105993 |
| rs2270894 | G | C | 3 | 0.00159875 | 1E-27 | 119.0313415 |
| rs2274116 | T | C | 9 | 0.00131768 | 8.30004E-10 | 37.68911453 |
| rs2281175 | C | T | 1 | 0.00132557 | 6.29941E-18 | 74.41741848 |
| rs2287547 | C | T | 12 | 0.00165819 | 0.000000012 | 32.50950721 |
| rs2293176 | A | G | 7 | 0.00131602 | 2.80027E-11 | 44.28899365 |
| rs2296316 | C | T | 14 | 0.00125664 | 1.20005E-16 | 68.55735631 |
| rs2305758 | T | C | 19 | 0.00138215 | 4.30002E-09 | 34.47736936 |
| rs2307111 | C | T | 5 | 0.00126894 | 2.09991E-38 | 167.9296872 |
| rs236587 | C | T | 17 | 0.001427 | 6.19998E-09 | 33.77157339 |
| rs237738 | C | T | 20 | 0.00162928 | 7.29995E-09 | 33.45561082 |
| rs240113 | A | G | 6 | 0.00124317 | 1.09999E-10 | 41.66056231 |
| rs2427320 | G | A | 20 | 0.00147567 | 0.000000025 | 31.0964925 |
| rs244711 | T | C | 5 | 0.00143325 | 1.20005E-31 | 136.9516396 |
| rs247008 | G | A | 5 | 0.0013272 | 3.59998E-21 | 89.1695432 |
| rs2521349 | A | G | 17 | 0.00128156 | 1.6E-09 | 36.42457231 |
| rs2524137 | T | C | 6 | 0.00135026 | 2.29985E-52 | 231.8703473 |
| rs254963 | G | A | 5 | 0.00125629 | 6.59933E-12 | 47.13619422 |
| rs2568958 | A | G | 1 | 0.00126246 | 5.50047E-14 | 56.54588259 |
| rs2578557 | T | C | 5 | 0.00129484 | 2.90001E-13 | 53.30418555 |
| rs25849 | G | C | 16 | 0.00137667 | 1.29987E-34 | 150.5111749 |
| rs2615075 | G | A | 1 | 0.00128182 | 1.99986E-14 | 58.53022295 |
| rs2678204 | G | T | 1 | 0.00130586 | 2.39994E-20 | 85.43497454 |
| rs2721938 | T | C | 8 | 0.00127027 | 1.29987E-21 | 91.23740982 |
| rs2726036 | C | A | 16 | 0.00127188 | 8.00018E-21 | 87.61406267 |
| rs2740761 | T | C | 7 | 0.00154571 | 0.000000017 | 31.83204851 |
| rs2763265 | G | A | 6 | 0.00145332 | 1.29999E-08 | 32.39246123 |
| rs2785078 | C | T | 10 | 0.00125453 | 1.40001E-08 | 32.21188824 |
| rs2789366 | A | G | 1 | 0.00129964 | 1.59993E-12 | 49.98060469 |
| rs281385 | G | A | 19 | 0.00191124 | 1.7E-10 | 40.78581668 |
| rs284315 | G | A | 1 | 0.00123489 | 4.90004E-10 | 38.71494088 |
| rs284662 | C | T | 19 | 0.001281 | 2.19989E-13 | 53.82931237 |
| rs28620532 | G | A | 9 | 0.00130855 | 6.4003E-44 | 193.1899241 |
| rs2866720 | T | C | 7 | 0.00128349 | 5.19996E-11 | 43.08703333 |
| rs2885697 | T | G | 1 | 0.00130925 | 5.10035E-47 | 207.3753984 |
| rs2897968 | A | G | 12 | 0.00127253 | 1.39991E-34 | 150.4016884 |
| rs2900208 | A | C | 12 | 0.00129881 | 4.90004E-27 | 115.9417643 |
| rs291979 | A | G | 10 | 0.0014791 | 1.80011E-12 | 49.6486065 |
| rs2920974 | C | A | 2 | 0.00125144 | 1.09999E-08 | 32.68095995 |
| rs2952615 | C | G | 5 | 0.00127671 | 8.9002E-21 | 87.39264459 |
| rs2979649 | G | A | 8 | 0.00164465 | 3.59998E-09 | 34.80788347 |
| rs2993531 | C | A | 1 | 0.00125604 | 2.80027E-13 | 53.31904029 |
| rs310796 | T | G | 12 | 0.00133483 | 2.09991E-16 | 67.4957221 |
| rs3110496 | G | A | 17 | 0.00133876 | 5.00035E-11 | 43.18950788 |
| rs3116201 | A | G | 2 | 0.00208476 | 7.10068E-18 | 74.1983298 |
| rs332162 | C | A | 10 | 0.00154953 | 5.80003E-10 | 38.37204145 |
| rs338361 | C | T | 15 | 0.00136821 | 3.90032E-12 | 48.15084497 |
| rs33967909 | A | G | 5 | 0.00150689 | 2.19989E-17 | 71.99453072 |
| rs34017457 | A | G | 16 | 0.00747734 | 6.89922E-11 | 42.55094431 |
| rs34055910 | G | A | 17 | 0.00128412 | 5.1E-09 | 34.15736702 |
| rs34227797 | C | G | 4 | 0.00134082 | 9.29994E-10 | 37.46902202 |
| rs34517439 | A | C | 1 | 0.00191453 | 3.29989E-84 | 378.0829215 |
| rs34693680 | T | C | 3 | 0.00182938 | 3.29989E-15 | 62.10547586 |
| rs34776209 | T | C | 7 | 0.00144079 | 1.50003E-32 | 141.1132066 |
| rs34825238 | T | G | 12 | 0.00144272 | 7.10003E-10 | 37.97946467 |
| rs34831515 | T | C | 19 | 0.00147768 | 1.29999E-08 | 32.33707253 |
| rs34848742 | G | T | 4 | 0.00151652 | 2.29985E-22 | 94.65571102 |
| rs34879158 | C | A | 20 | 0.00141463 | 2.19989E-52 | 231.9762651 |
| rs35050648 | T | G | 19 | 0.00146534 | 0.000000016 | 31.97942867 |
| rs35251247 | A | G | 11 | 0.00137513 | 3.80014E-15 | 61.77706449 |
| rs35506085 | A | G | 11 | 0.00161208 | 8.10028E-35 | 151.5009027 |
| rs35612982 | C | T | 6 | 0.00161745 | 1.09999E-08 | 32.66718425 |
| rs35665085 | A | G | 22 | 0.00271032 | 9.20005E-10 | 37.47852693 |
| rs357486 | C | T | 3 | 0.00124304 | 6.70039E-16 | 65.22189243 |
| rs35756741 | T | C | 12 | 0.00215406 | 1.59993E-13 | 54.43861968 |
| rs35874463 | G | A | 15 | 0.00266581 | 9.70063E-12 | 46.39415695 |
| rs35897671 | T | C | 5 | 0.00130487 | 4.49987E-22 | 93.28681625 |
| rs36000545 | G | A | 17 | 0.00128804 | 8.19974E-38 | 165.2259914 |
| rs3730071 | A | C | 12 | 0.00362243 | 3.2E-10 | 39.56693143 |
| rs3734254 | T | C | 6 | 0.00152073 | 4.70002E-27 | 116.026017 |
| rs3740591 | T | C | 10 | 0.0012624 | 1.69981E-19 | 81.51841239 |
| rs3751866 | C | T | 16 | 0.00144366 | 1.89998E-08 | 31.63713787 |
| rs3772051 | A | G | 2 | 0.00147954 | 5.89997E-09 | 33.85145417 |
| rs3782232 | A | G | 12 | 0.00241514 | 4.40048E-17 | 70.59356031 |
| rs3783256 | C | T | 13 | 0.00130766 | 6.70039E-13 | 51.64195083 |
| rs3809569 | G | A | 15 | 0.00145343 | 2.09991E-22 | 94.78843023 |
| rs3810291 | A | G | 19 | 0.00132769 | 2.39994E-37 | 163.0805697 |
| rs3822742 | A | C | 5 | 0.00128672 | 1.59993E-25 | 109.0137747 |
| rs3827910 | G | A | 14 | 0.00146479 | 1.9002E-13 | 54.1017448 |
| rs3843750 | G | C | 19 | 0.001319 | 7.19946E-24 | 101.4945497 |
| rs3853252 | A | G | 6 | 0.00124887 | 1.69981E-34 | 149.9829116 |
| rs3925 | A | G | 8 | 0.00144625 | 1.20005E-13 | 55.03511044 |
| rs41271299 | T | C | 6 | 0.00280542 | 2.29985E-50 | 222.7163275 |
| rs41311445 | C | A | 22 | 0.00212069 | 1.59993E-30 | 131.9157896 |
| rs4132132 | C | T | 4 | 0.00125989 | 4.70002E-12 | 47.79462188 |
| rs41458449 | C | G | 2 | 0.0017678 | 1.89998E-09 | 36.078785 |
| rs4240326 | G | A | 4 | 0.0012456 | 3.69999E-65 | 290.5962894 |
| rs4268495 | C | G | 11 | 0.00128273 | 2.19989E-11 | 44.74521395 |
| rs4282339 | A | G | 5 | 0.00152518 | 2.39994E-35 | 153.9123649 |
| rs434072 | G | T | 3 | 0.00137154 | 4.09996E-08 | 30.11997248 |
| rs4369779 | C | T | 18 | 0.0015236 | 2.60016E-65 | 291.3299595 |
| rs4439140 | A | G | 8 | 0.00129099 | 4.70002E-11 | 43.30410476 |
| rs4477562 | T | C | 13 | 0.00187416 | 1.9002E-20 | 85.85315385 |
| rs4504126 | C | A | 3 | 0.00379875 | 4.30031E-11 | 43.45182637 |
| rs4525525 | T | G | 17 | 0.00142939 | 5.90065E-11 | 42.86241817 |
| rs4567604 | T | G | 13 | 0.00158373 | 0.000000005 | 34.18202411 |
| rs4635681 | G | A | 3 | 0.00170574 | 1.09999E-10 | 41.56981287 |
| rs4648626 | A | C | 1 | 0.00124402 | 9.09913E-12 | 46.50575139 |
| rs4676442 | C | T | 2 | 0.00129244 | 9.09997E-09 | 33.01935927 |
| rs4788218 | C | T | 16 | 0.00126829 | 3.59998E-66 | 295.2086936 |
| rs4795318 | T | C | 17 | 0.00124512 | 7.10068E-13 | 51.50741257 |
| rs4800670 | C | G | 18 | 0.00128248 | 2.30001E-08 | 31.25687738 |
| rs4819021 | C | T | 21 | 0.0012602 | 6.4E-10 | 38.1995013 |
| rs485554 | C | G | 3 | 0.0013389 | 1.80011E-37 | 163.7019814 |
| rs4858940 | C | T | 3 | 0.00194633 | 1.20005E-16 | 68.61275852 |
| rs4865956 | A | T | 5 | 0.00135296 | 7.29962E-19 | 78.69453399 |
| rs4899012 | C | G | 14 | 0.00127368 | 1.10002E-63 | 283.8005911 |
| rs490535 | T | C | 20 | 0.00131354 | 0.000000016 | 31.91001869 |
| rs4906203 | T | C | 14 | 0.00146932 | 2.59998E-10 | 39.97223601 |
| rs4926542 | T | C | 1 | 0.00133689 | 2.39994E-13 | 53.6747023 |
| rs4968799 | T | A | 17 | 0.00196938 | 7.29962E-21 | 87.77986204 |
| rs4974223 | T | C | 3 | 0.00210098 | 1E-14 | 59.85614417 |
| rs4980067 | A | C | 10 | 0.00124184 | 1.80011E-19 | 81.40548591 |
| rs4980826 | A | C | 12 | 0.00126664 | 9.29994E-10 | 37.4644815 |
| rs498685 | C | T | 18 | 0.00126587 | 2.19999E-08 | 31.31443337 |
| rs5017213 | C | T | 1 | 0.00129352 | 6.80002E-10 | 38.08122268 |
| rs505575 | C | T | 5 | 0.00132413 | 2.60016E-11 | 44.43615427 |
| rs508347 | C | T | 7 | 0.0013584 | 7.8001E-18 | 74.00001719 |
| rs536007 | T | G | 11 | 0.00135621 | 3.80014E-22 | 93.60802556 |
| rs55665822 | G | A | 5 | 0.00142848 | 3.10027E-16 | 66.72966601 |
| rs55674305 | A | G | 8 | 0.0013512 | 3.10027E-16 | 66.75247956 |
| rs55727637 | T | C | 16 | 0.00134922 | 6.09958E-20 | 83.59555412 |
| rs55745410 | G | A | 1 | 0.00129888 | 2.99985E-25 | 107.768785 |
| rs55758152 | A | G | 5 | 0.00133828 | 2.80027E-13 | 53.3109047 |
| rs55766788 | T | C | 17 | 0.001965 | 5.30029E-11 | 43.06164732 |
| rs55800172 | A | G | 1 | 0.00252769 | 5.70033E-13 | 51.96216788 |
| rs55831773 | T | C | 17 | 0.00158701 | 4.10015E-20 | 84.36900737 |
| rs55854145 | C | A | 18 | 0.0027447 | 2.39999E-08 | 31.13507449 |
| rs56130943 | C | A | 14 | 0.00151098 | 2.90001E-09 | 35.27887326 |
| rs56141370 | T | C | 9 | 0.00195101 | 1E-10 | 41.7433579 |
| rs56207600 | A | G | 11 | 0.00198839 | 9.59997E-10 | 37.41411048 |
| rs57126421 | G | A | 18 | 0.00147137 | 3.80014E-13 | 52.76933278 |
| rs5742915 | C | T | 15 | 0.0012484 | 3.19963E-14 | 57.62675428 |
| rs5752989 | A | G | 22 | 0.00125977 | 2.90001E-15 | 62.31818893 |
| rs5753630 | G | A | 22 | 0.00125982 | 6.69993E-09 | 33.63396004 |
| rs5771118 | C | T | 22 | 0.00143238 | 1.79999E-09 | 36.21798071 |
| rs578366 | G | A | 6 | 0.00125287 | 3.50026E-16 | 66.48378533 |
| rs59985551 | T | C | 2 | 0.00147745 | 8.60003E-36 | 155.962052 |
| rs6026578 | G | C | 20 | 0.0012919 | 6.79986E-14 | 56.11365575 |
| rs603321 | G | A | 6 | 0.00144747 | 1.59993E-15 | 63.52821349 |
| rs60385590 | A | C | 3 | 0.00143357 | 1E-10 | 41.7657444 |
| rs60804050 | A | G | 1 | 0.00141541 | 1.50003E-12 | 50.11236713 |
| rs6081869 | G | T | 20 | 0.00129529 | 4.40048E-15 | 61.50224123 |
| rs6085658 | T | C | 20 | 0.00127705 | 1.59993E-11 | 45.35366718 |
| rs6142059 | C | T | 20 | 0.00124689 | 1.10002E-14 | 59.75550751 |
| rs61729527 | T | C | 8 | 0.00280238 | 1.9002E-17 | 72.28136603 |
| rs61749613 | G | A | 5 | 0.00310812 | 1.29987E-15 | 63.85666246 |
| rs61878760 | A | G | 11 | 0.00226393 | 2.80027E-11 | 44.29164626 |
| rs62372052 | G | A | 5 | 0.00198772 | 6.79986E-41 | 179.3127031 |
| rs62439025 | C | G | 6 | 0.00172995 | 1.79999E-10 | 40.64186872 |
| rs62460525 | A | C | 7 | 0.00243085 | 0.000000017 | 31.82030155 |
| rs62466118 | A | G | 7 | 0.00397074 | 1.40001E-10 | 41.11799145 |
| rs62476192 | T | C | 7 | 0.00220867 | 2.19989E-12 | 49.32450421 |
| rs62515437 | T | G | 8 | 0.00148739 | 1.39991E-38 | 168.6825325 |
| rs62621197 | T | C | 19 | 0.0034154 | 1.39991E-21 | 91.01095197 |
| rs62621400 | G | C | 15 | 0.00267267 | 9.79941E-14 | 55.41115583 |
| rs62621812 | A | G | 7 | 0.00452815 | 5.90065E-21 | 88.20350475 |
| rs6493534 | C | T | 15 | 0.00126923 | 3.59998E-08 | 30.35413286 |
| rs6514066 | G | T | 20 | 0.00128608 | 0.000000025 | 31.0749379 |
| rs6563808 | C | T | 13 | 0.00141464 | 1.7E-09 | 36.33294974 |
| rs6570509 | T | G | 6 | 0.00136794 | 3.90032E-25 | 107.2620798 |
| rs6575340 | A | G | 14 | 0.00129572 | 3.59998E-10 | 39.29640652 |
| rs6585827 | A | G | 10 | 0.00124341 | 3.80014E-20 | 84.51172025 |
| rs6591 | T | C | 11 | 0.00125332 | 1.89998E-10 | 40.55760505 |
| rs663344 | C | G | 9 | 0.00163963 | 3.69999E-20 | 84.57721309 |
| rs6665399 | C | T | 1 | 0.00124146 | 4.90004E-11 | 43.2060595 |
| rs6669189 | T | C | 1 | 0.00126558 | 2.49977E-11 | 44.50609763 |
| rs6680471 | T | C | 1 | 0.00124005 | 3.40001E-09 | 34.92404431 |
| rs6681795 | G | A | 1 | 0.00147749 | 1.59993E-24 | 104.4795599 |
| rs6693481 | C | T | 1 | 0.00134714 | 8.60003E-10 | 37.61651513 |
| rs67141907 | T | C | 13 | 0.00175247 | 1.7E-10 | 40.81982535 |
| rs6719296 | A | G | 2 | 0.00124466 | 1.10002E-12 | 50.57115152 |
| rs6721191 | G | A | 2 | 0.00125273 | 0.000000002 | 36.00057475 |
| rs6743060 | A | C | 2 | 0.00164044 | 1.10002E-93 | 421.5426459 |
| rs6743107 | T | C | 2 | 0.00138466 | 1.29999E-08 | 32.2872733 |
| rs67551338 | T | C | 12 | 0.00261202 | 1.39991E-22 | 95.56113745 |
| rs6762578 | A | G | 3 | 0.00149834 | 8.9002E-26 | 110.1971679 |
| rs6764533 | A | G | 3 | 0.00129282 | 3.79997E-10 | 39.19270183 |
| rs6781248 | A | G | 3 | 0.00124552 | 1.29999E-09 | 36.81014674 |
| rs6800021 | A | G | 3 | 0.00125477 | 1.59993E-27 | 118.1213863 |
| rs68119703 | T | C | 2 | 0.00125123 | 0.000000012 | 32.47468616 |
| rs6874142 | G | T | 5 | 0.00206704 | 1.39991E-18 | 77.45914198 |
| rs6900690 | A | G | 6 | 0.00124509 | 1.09999E-10 | 41.60083712 |
| rs6902789 | A | G | 6 | 0.00128924 | 1.10002E-16 | 68.80900927 |
| rs6946415 | G | A | 7 | 0.00128554 | 6.29941E-49 | 216.1302984 |
| rs6946419 | C | T | 7 | 0.00128435 | 7.19996E-10 | 37.97365336 |
| rs695922 | G | A | 5 | 0.00169607 | 1.7E-09 | 36.34595444 |
| rs696343 | G | A | 12 | 0.00194468 | 9.09997E-09 | 33.01810401 |
| rs6972291 | C | T | 7 | 0.00163261 | 7.70016E-12 | 46.84467203 |
| rs6984820 | T | C | 8 | 0.00126049 | 8.49963E-16 | 64.74446696 |
| rs7033487 | C | T | 9 | 0.00156114 | 4.49987E-51 | 225.9888238 |
| rs703593 | G | A | 12 | 0.00124074 | 4.10015E-20 | 84.38203865 |
| rs705953 | G | A | 6 | 0.00131226 | 1.10002E-12 | 50.64795317 |
| rs708723 | T | C | 1 | 0.00124122 | 8.4004E-27 | 114.863505 |
| rs7095768 | T | G | 10 | 0.00140856 | 2.69998E-08 | 30.88918213 |
| rs7129320 | A | G | 11 | 0.00166904 | 1.10002E-43 | 192.0581744 |
| rs7132908 | A | G | 12 | 0.00127564 | 2.90001E-35 | 153.5863223 |
| rs7134283 | A | G | 12 | 0.00138066 | 1.20005E-17 | 73.19488696 |
| rs71385734 | G | T | 16 | 0.00166494 | 1.20005E-43 | 191.9453191 |
| rs71393968 | A | G | 16 | 0.00297504 | 6.89922E-13 | 51.58335812 |
| rs71423263 | G | T | 2 | 0.00177706 | 1.50003E-11 | 45.53245954 |
| rs7145429 | T | C | 14 | 0.00314537 | 4.40048E-11 | 43.44658825 |
| rs7178905 | G | A | 15 | 0.00182735 | 3.59998E-09 | 34.84122872 |
| rs7188009 | A | G | 16 | 0.00126786 | 7.00003E-12 | 47.02920115 |
| rs7223535 | A | G | 17 | 0.00139944 | 1E-71 | 320.7176677 |
| rs7229520 | A | G | 18 | 0.00131762 | 1.99986E-15 | 63.01705458 |
| rs7245985 | G | T | 19 | 0.00153676 | 1.50003E-12 | 50.10280708 |
| rs72656010 | C | T | 8 | 0.0018399 | 2.80027E-88 | 396.7369391 |
| rs72703409 | C | G | 4 | 0.0025077 | 0.000000002 | 35.94307791 |
| rs7280982 | A | G | 21 | 0.00150596 | 4.20001E-09 | 34.52089178 |
| rs73004967 | G | A | 19 | 0.0024734 | 1.29987E-11 | 45.79230104 |
| rs7301341 | C | T | 12 | 0.00132363 | 9.30037E-15 | 60.04455543 |
| rs73013411 | A | C | 6 | 0.00183747 | 8.9002E-13 | 51.0656223 |
| rs73052033 | C | T | 3 | 0.00159865 | 5.19996E-22 | 92.99249239 |
| rs7312646 | C | A | 12 | 0.00126518 | 9.20026E-17 | 69.13302494 |
| rs73158212 | G | A | 7 | 0.00150656 | 0.000000025 | 31.0644042 |
| rs73175572 | G | A | 3 | 0.00198793 | 6.09958E-45 | 197.8718376 |
| rs73189390 | A | G | 21 | 0.00161779 | 2.30001E-08 | 31.25787175 |
| rs7321045 | A | G | 13 | 0.00125985 | 1.29987E-11 | 45.82780054 |
| rs73213484 | T | A | 4 | 0.00178131 | 1.50003E-11 | 45.47279354 |
| rs73601548 | T | C | 10 | 0.00195798 | 4.79954E-13 | 52.28696673 |
| rs73619441 | G | T | 20 | 0.00177444 | 1.50003E-12 | 50.07506037 |
| rs74048171 | A | C | 11 | 0.00142123 | 3.19963E-13 | 53.09213485 |
| rs744205 | A | G | 11 | 0.00124912 | 1E-17 | 73.52095963 |
| rs74494415 | T | C | 18 | 0.00321022 | 4.60045E-15 | 61.43129285 |
| rs7485647 | A | G | 12 | 0.00171023 | 2.90001E-08 | 30.80232801 |
| rs7513326 | A | G | 1 | 0.00125821 | 0.000000025 | 31.04757265 |
| rs752070 | G | A | 2 | 0.00186451 | 8.19993E-10 | 37.70756048 |
| rs7535501 | T | C | 1 | 0.00269284 | 3.29997E-09 | 34.99843453 |
| rs7549783 | T | C | 1 | 0.00159561 | 1.29999E-08 | 32.34350823 |
| rs7619139 | A | T | 3 | 0.00126254 | 3.40017E-21 | 89.29497034 |
| rs76364830 | A | G | 8 | 0.00257118 | 2.39994E-18 | 76.35895952 |
| rs7647657 | G | A | 3 | 0.00127681 | 9.09913E-19 | 78.2522528 |
| rs76513770 | C | T | 16 | 0.00185994 | 5.10035E-18 | 74.83011802 |
| rs76693355 | C | T | 11 | 0.00194095 | 8.99912E-14 | 55.57332051 |
| rs7671110 | T | C | 4 | 0.00169681 | 2.80027E-78 | 350.8029368 |
| rs7683836 | A | G | 4 | 0.00125172 | 3.40001E-08 | 30.4596901 |
| rs76875574 | A | C | 15 | 0.00278961 | 2.69998E-08 | 30.94018743 |
| rs77093479 | G | C | 17 | 0.00168799 | 6.90001E-10 | 38.0521002 |
| rs77165542 | T | C | 2 | 0.00338396 | 6.59933E-67 | 298.6001484 |
| rs7728690 | T | C | 5 | 0.00127302 | 5.00035E-18 | 74.86160277 |
| rs7731023 | G | A | 5 | 0.00125278 | 7.8001E-12 | 46.81863578 |
| rs77344209 | T | C | 3 | 0.00144504 | 1.20005E-11 | 46.0039603 |
| rs7740107 | A | T | 6 | 0.00140391 | 1.10002E-95 | 430.7063968 |
| rs7755185 | G | A | 6 | 0.00133858 | 1E-10 | 41.82946315 |
| rs7780752 | C | T | 7 | 0.00129087 | 5.40008E-22 | 92.92979715 |
| rs77848106 | A | C | 1 | 0.00136556 | 3.90032E-11 | 43.67437006 |
| rs78378222 | G | T | 17 | 0.00576633 | 7.39946E-46 | 202.0530072 |
| rs7843128 | C | T | 8 | 0.00129837 | 2E-10 | 40.49807782 |
| rs78751935 | C | T | 5 | 0.00349984 | 2.99999E-10 | 39.68354877 |
| rs7910211 | C | T | 10 | 0.00170194 | 4.79999E-09 | 34.27401631 |
| rs7952436 | T | C | 11 | 0.00226417 | 6.70039E-50 | 220.6022019 |
| rs7962636 | C | T | 12 | 0.00130234 | 5.49997E-10 | 38.47801852 |
| rs798759 | G | A | 4 | 0.0012402 | 6.70039E-15 | 60.67621101 |
| rs8002779 | A | G | 13 | 0.00126739 | 1.5E-09 | 36.49041136 |
| rs8007644 | A | G | 14 | 0.00128683 | 3.40001E-10 | 39.40997888 |
| rs8074074 | T | C | 17 | 0.00144929 | 6.79986E-11 | 42.58672591 |
| rs8088739 | C | T | 18 | 0.00144789 | 1.29999E-08 | 32.28982568 |
| rs8091374 | A | G | 18 | 0.00172549 | 1.09999E-08 | 32.7327038 |
| rs822549 | C | T | 7 | 0.00140293 | 1.39991E-17 | 72.89115942 |
| rs843374 | T | A | 3 | 0.00125963 | 5.40008E-19 | 79.26784929 |
| rs892511 | G | C | 1 | 0.00378949 | 3.69999E-12 | 48.29459386 |
| rs894360 | C | T | 8 | 0.00129354 | 5.70033E-49 | 216.3346715 |
| rs9291926 | G | T | 5 | 0.00124336 | 6.09958E-22 | 92.70349356 |
| rs9327336 | C | T | 5 | 0.00130542 | 3.19963E-11 | 44.05970874 |
| rs933048 | C | T | 10 | 0.00128332 | 2.39999E-08 | 31.10140244 |
| rs9350850 | C | T | 6 | 0.00228783 | 1.50003E-24 | 104.5599057 |
| rs9369308 | G | A | 6 | 0.00183186 | 9.3994E-24 | 100.9641769 |
| rs9372837 | A | G | 6 | 0.00125109 | 6.79986E-34 | 147.2994092 |
| rs9379084 | A | G | 6 | 0.00199505 | 2.70023E-14 | 57.91491891 |
| rs943190 | T | C | 10 | 0.00126937 | 3.09999E-10 | 39.62073556 |
| rs9465505 | A | G | 6 | 0.00124524 | 4.60045E-11 | 43.34672791 |
| rs9480933 | G | C | 6 | 0.00129843 | 9.30037E-24 | 100.9743047 |
| rs9491201 | T | C | 6 | 0.00167258 | 9.49992E-09 | 32.9432129 |
| rs9513510 | C | G | 13 | 0.00135596 | 1E-15 | 64.41507834 |
| rs9540493 | G | A | 13 | 0.00125617 | 1.50003E-12 | 50.09457642 |
| rs9634212 | A | C | 12 | 0.0015016 | 4.00037E-56 | 249.156638 |
| rs9654451 | T | C | 5 | 0.0018522 | 1.40001E-09 | 36.71030467 |
| rs968821 | C | G | 9 | 0.00131107 | 2.09991E-17 | 72.06346766 |
| rs9800418 | C | T | 5 | 0.00144872 | 7.90005E-09 | 33.30258375 |
| rs9853018 | T | C | 3 | 0.00124617 | 6.2951E-128 | 578.966616 |
| rs9884540 | G | T | 4 | 0.00127923 | 4.09996E-10 | 39.05558697 |
| rs9892365 | G | A | 17 | 0.00132317 | 8.9002E-33 | 142.1839554 |
| rs9898189 | G | C | 17 | 0.00134891 | 2.1E-09 | 35.91891354 |
| rs9925273 | G | A | 16 | 0.00161653 | 2.70023E-13 | 53.39238723 |
| rs9938120 | T | C | 16 | 0.00179345 | 9.3994E-25 | 105.5275197 |
| rs9951619 | G | T | 18 | 0.00148235 | 4.00037E-17 | 70.77844925 |
| rs9960619 | T | C | 18 | 0.00131041 | 8.69961E-14 | 55.65161594 |

**Table S12** The results of the horizontal pleiotropy analysis.

| Exposure | Outcome | Egger_intercept | P |
| --- | --- | --- | --- |
| BMI | Heart Failure | -0.0002746246 | 0.8984722 |
| WC | Heart Failure | 0.0002083227 | 0.9364172 |
| WHR | Heart Failure | 0.006506332 | 0.7024938 |
| Arm fat mass (right) | Heart Failure | 0.0001423331 | 0.9495062 |
| Leg fat mass (right) | Heart Failure | 9.769553e-06 | 0.9968003 |
| Trunk fat mass | Heart Failure | 0.002273001 | 0.3340587 |
| Whole-body fat mass | Heart Failure | 0.0002694136 | 0.906924 |
| Arm fat-free mass (right) | Heart Failure | 0.002155827 | 0.2038111 |
| Leg fat-free mass (right) | Heart Failure | 0.0002875231 | 0.865134 |
| Trunk fat-free mass | Heart Failure | 0.0002259047 | 0.879509 |
| Whole-body fat-free mass | Heart Failure | 0.001390055 | 0.3710016 |

Abbreviations: BMI: body mass index; WC: waist circumference; WHR: waist-to-hip ratio.

**Table S13** The results of the weighted median analysis.

| Exposure | Outcome | HR (95 % CI) | P |
| --- | --- | --- | --- |
| BMI | Heart Failure | 1.55 (1.38 - 1.74) | 1.20E-13 |
| WC | Heart Failure | 1.66 (1.44 - 1.92) | 5.29E-12 |
| WHR | Heart Failure | 1.24 (0.92 - 1.68) | 0.15 |
| Arm fat mass (right) | Heart Failure | 1.50 (1.33 - 1.69) | 5.68E-11 |
| Leg fat mass (right) | Heart Failure | 1.63 (1.40 - 1.90) | 2.00E-10 |
| Trunk fat mass | Heart Failure | 1.35 (1.20 - 1.51) | 3.68E-07 |
| Whole-body fat mass | Heart Failure | 1.38 (1.22 - 1.57) | 4.54E-07 |
| Arm fat-free mass (right) | Heart Failure | 1.37 (1.16 - 1.61) | 1.50E-04 |
| Leg fat-free mass (right) | Heart Failure | 1.40 (1.20 - 1.64) | 2.00E-05 |
| Trunk fat-free mass | Heart Failure | 1.30 (1.13 - 1.49) | 2.11E-04 |
| Whole-body fat-free mass | Heart Failure | 1.31 (1.14 - 1.52) | 2.26E-04 |

Abbreviations: BMI: body mass index; WC: waist circumference; WHR: waist-to-hip ratio; HR:Hazard ratio; CI: Confidence interval.

**Table S14** Multivariate MR of Whole body fat-free mass, Whole body fat mass and Heart Failure.

| Exposure | Outcome | HR (95 % CI) | P |
| --- | --- | --- | --- |
| Whole body fat-free mass | Heart Failure | 1.07 (0.93 - 1.26) | 0.33 |
| Whole body fat mass | Heart Failure | 1.66 (1.44 - 1.91) | 1.92E-12 |

Abbreviations: HR:Hazard ratio; CI: Confidence interval.

**Table S15** Multivariate MR of Whole body fat-free mass, BMI and Heart Failure.

| Exposure | Outcome | HR (95 % CI) | P |
| --- | --- | --- | --- |
| Whole body fat-free mass | Heart Failure | 1.26 (1.12 - 1.42) | 1.17E-04 |
| BMI | Heart Failure | 1.55 (1.39 - 1.73) | 1.92E-12 |

Abbreviations: BMI:Body mass index; HR:Hazard ratio; CI: Confidence interval.
